# Comparison of Prophylactic Intravenous Antibiotic Regimens After Endoprosthetic Reconstruction for Lower Extremity Bone Tumors: A Randomized Clinical Trial

**DOI:** 10.1001/jamaoncol.2021.6628

**Published:** 2022-01-06

**Authors:** The Prophylactic Antibiotic Regimens in Tumor Surgery (PARITY) Investigators, Michelle Ghert, Patricia Schneider, Gordon Guyatt, Lehana Thabane, Roberto Vélez, Timothy O’Shea, R. Lor Randall, Robert Turcotte, David Wilson, Jay S. Wunder, André Mathias Baptista, Edward Y. Cheng, Yee-Cheen Doung, Peter C. Ferguson, Victoria Giglio, James Hayden, Diane Heels-Ansdell, Shah Alam Khan, Venkatesan Sampath Kumar, Paula McKay, Benjamin Miller, Michiel van de Sande, Juan P. Zumárraga, Mohit Bhandari

**Affiliations:** 1 Division of Orthopaedic Surgery, Department of Surgery, McMaster University, Hamilton, Ontario, Canada; 2 Department of Medicine, McMaster University, Hamilton, Ontario, Canada; 3 Department of Health Research Methods, Evidence and Impact, McMaster University, Hamilton, Ontario, Canada; 4 Hospital Vall d’Hebron, Vall d’Hebron Institut de Recerca, Barcelona, Spain; 5 Department of Orthopaedic Surgery, University of California Davis Health, Sacramento; 6 Division of Orthopaedic Surgery, Department of Surgery, McGill University, Montreal, Quebec, Canada; 7 University of Toronto Musculoskeletal Oncology Unit, Mount Sinai Hospital, Division of Orthopaedic Surgery, Department of Surgery, University of Toronto, Toronto, Ontario, Canada; 8 Instituto de Ortopedia e Traumatologia do Hospital das Clínicas da Universidade de São Paulo, São Paulo, Brazil; 9 Department of Orthopaedic Surgery, University of Minnesota, Minneapolis; 10 Department of Orthopaedics and Rehabilitation, Oregon Health & Science University, Portland; 11 Department of Orthopaedics, All India Institute of Medical Sciences, Delhi, India; 12 Department of Orthopaedics and Rehabilitation, University of Iowa, Iowa City; 13 Department of Orthopaedic Surgery, Leids Universitair Medisch Centrum, Leiden, the Netherlands

## Abstract

**Importance:**

The use of perioperative, prophylactic, intravenous antibiotics is standard practice to reduce the risk of surgical site infection after oncologic resection and complex endoprosthetic reconstruction for lower extremity bone tumors. However, evidence guiding the duration of prophylactic treatment remains limited.

**Objective:**

To assess the effect of a 5-day regimen of postoperative, prophylactic, intravenous antibiotics compared with a 1-day regimen on the rate of surgical site infections within 1 year after surgery.

**Design, Setting, and Participants:**

This randomized clinical superiority trial was performed at 48 clinical sites in 12 countries from January 1, 2013, to October 29, 2019. The trial included patients with a primary bone tumor or a soft tissue sarcoma that had invaded the femur or tibia or oligometastatic bone disease of the femur or tibia with expected survival of at least 1 year who required surgical management by excision and endoprosthetic reconstruction. A total of 611 patients were enrolled, and 7 were excluded for ineligibility.

**Interventions:**

A 1- or 5-day regimen of postoperative prophylactic intravenous cephalosporin (cefazolin or cefuroxime) that began within 8 hours after skin closure and was administered every 8 hours thereafter. Those randomized to the 1-day regimen received identical saline doses every 8 hours for the remaining 4 days; patients, care providers, and outcomes assessors were blinded to treatment regimen.

**Main Outcomes and Measures:**

The primary outcome in this superiority trial was a surgical site infection (superficial incisional, deep incisional, or organ space) classified according to the criteria established by the Centers for Disease Control and Prevention within 1 year after surgery. Secondary outcomes included antibiotic-related complications, unplanned additional operations, oncologic and functional outcomes, and mortality.

**Results:**

Of the 604 patients included in the final analysis (mean [SD] age, 41.2 [21.9] years; 361 [59.8%] male; 114 [18.9%] Asian, 43 [7.1%] Black, 34 [5.6%] Hispanic, 15 [2.5%] Indigenous, 384 [63.8%] White, and 12 [2.0%] other), 293 were randomized to a 5-day regimen and 311 to a 1-day regimen. A surgical site infection occurred in 44 patients (15.0%) allocated to the 5-day regimen and in 52 patients (16.7%) allocated to the 1-day regimen (hazard ratio, 0.93; 95% CI, 0.62-1.40; *P* = .73). Antibiotic-related complications occurred in 15 patients (5.1%) in the 5-day regimen and in 5 patients (1.6%) allocated to the 1-day regimen (hazard ratio, 3.24; 95% CI, 1.17-8.98; *P* = .02). Other secondary outcomes did not differ significantly between treatment groups.

**Conclusions and Relevance:**

This randomized clinical trial did not confirm the superiority of a 5-day regimen of postoperative intravenous antibiotics over a 1-day regimen in preventing surgical site infections after surgery for lower extremity bone tumors that required an endoprosthesis. The 5-day regimen group had significantly more antibiotic-related complications.

**Trial Registration:**

ClinicalTrials.gov Identifier: NCT01479283

## Key Points

**Question:**

Can a 5-day regimen of postoperative, prophylactic, intravenous antibiotics reduce the rate of surgical site infections in patients with a lower extremity bone tumor undergoing complex endoprosthetic reconstruction compared with a 1-day regimen?

**Findings:**

In this randomized clinical trial including 604 patients in the primary analysis, the 5-day regimen did not reduce the rate of surgical site infection compared with the 1-day regimen, although it resulted in a higher rate of antibiotic-related complications, notably *Clostridioides difficile*–associated colitis.

**Meaning:**

The results of this study suggest that prolonging use of postoperative antibiotics beyond 1 day does not reduce the rate of surgical site infection but increases the risk of clinically significant antibiotic-related complications.

## Teaser

This randomized clinical trial of patients with a primary bone tumor or a soft tissue sarcoma compares the effects of a 5-day vs 1-day regimen of postoperative, prophylactic, intravenous antibiotics on surgical site infections within 1 year after surgery.

## Introduction

Operations to remove malignant tumors of the femur or tibia involve the resection of the affected bone and surrounding soft tissue followed by complex reconstruction of the limb. The most common reconstruction involves the use of modular metallic and polyethylene endoprostheses to replace surgically resected bones and joints. Because of the length and intricacies of these procedures and the medical complexity of these patients, the risk of a surgical site infection is high.^1,2,3^ Attempts to eradicate the organism often fail and result in the eventual need for amputation in nearly 50% of patients, thereby strongly affecting patient function and quality of life and delaying the administration of adjuvant cancer therapies.^1,2,3,4^

Strategies to minimize surgical site infections in this population include the administration of perioperative intravenous antibiotics. The most effective antibiotic regimen to prevent surgical site infections remains uncertain and current clinical practice is highly varied, particularly with respect to antibiotic duration. Approximately 2 of every 3 surgeons prescribe prolonged courses well beyond the 24-hour recommended duration for standard total joint replacement operations.^5,6,7,8^ However, overuse of antibiotics can lead to antibiotic-related complications and antibiotic resistance; antibiotic stewardship, therefore, remains a salient issue.^9^ We conducted the Prophylactic Antibiotic Regimens in Tumor Surgery (PARITY) trial to inform the effect of a 5-day regimen of postoperative, prophylactic, intravenous antibiotics compared with a 1-day regimen on the prevention of surgical site infections and on antibiotic adverse effects in patients requiring surgical resection and endoprosthetic reconstruction for a lower extremity bone tumor.

## Methods

### Trial Design and Oversight

This intention-to-treat study was an investigator-initiated, international, blinded (patients, surgeons, outcomes assessors, and data analysts), parallel, superiority randomized clinical trial. The rationale, design, and methods of the trial have been previously published.^10^ Written informed consent was required to participate in the study, and all data were deidentified. Supplement 1 (the Trial Protocol) and Supplement 2 provide information on eligibility criteria, interventions, blinding, follow-up, outcomes definitions, and statistical analysis. The Hamilton Integrated Research Ethics Board as well as the relevant local ethics committee at each participating site approved the trial protocol and its amendments before local study initiation. In addition, the following regulatory bodies also approved the trial protocol and its amendments: Health Canada, the Brazilian National Health Surveillance Agency, the European Medicines Agency (including the competent authorities in Austria and Spain), the Indian Council of Medical Research, and the Republic of South Africa’s Department of Health. The PARITY Data and Safety Monitoring Board, composed of 2 orthopedic oncologists and 1 statistician who were independent of the study team, reviewed the trial outcomes. This study followed the Consolidated Standards of Reporting Trials (CONSORT) reporting guideline.

### Patients

From January 1, 2013, to October 29, 2019, investigators at 48 clinical sites across Canada, the US, Argentina, Australia, Austria, Brazil, Egypt, India, the Netherlands, Singapore, South Africa, and Spain recruited patients for the study. Eligible patients included all individuals 12 years or older with a primary bone tumor or a soft tissue sarcoma that had invaded the femur or tibia or oligometastatic bone disease of the femur or tibia with expected survival of at least 1 year who required surgical management by excision and endoprosthetic reconstruction. Patients with previous infections at the surgical site or who were known to be colonized with methicillin-resistant *Staphylococcus aureus* or vancomycin-resistant *Enterococcus* were excluded. A total of 895 patients were screened for eligibility, and 7 patients were adjudicated to be ineligible at the time of randomization; thus, of the 611 patients randomized, 604 were included in the final analyses. The final 1-year assessments were completed in March 2021. Details on the eligibility criteria are available in the eAppendix in Supplement 2.

### Trial Interventions and Procedures

Surgical procedures were performed according to the standard practices at each clinical site. All patients received standardized preoperative and intraoperative prophylactic intravenous antibiotics. Patients were randomly allocated perioperatively in a 1-to-1 ratio to receive a 1- or 5-day postoperative prophylactic regimen of an intravenous cephalosporin (cefazolin or cefuroxime); they were blinded to the treatment regimen. Those randomized to the 1-day regimen received identical saline (placebo) doses for the remaining 4 days. Randomization, stratified according to tumor location (femur or tibia) and clinical site, was centralized through an internet-based, computer-generated platform that concealed allocation and used randomly permuted blocks of 2 or 4. An unblinded member of the local investigational pharmacy performed the randomization. Patients began their randomly allocated, postoperative, prophylactic antibiotic regimen within 8 hours after skin closure, and the doses were intravenously administered every 8 hours. Clinical sites used their own inventory to prepare the study antibiotics or placebo. Preparation, blinding of study antibiotics or placebo, and storage and administration of the study antibiotics were conducted as per local procedures established at each clinical site and the relevant manufacturers’ labels. Further details on the intravenous antibiotic regimens are available in the eAppendix in Supplement 2.

### Outcome Measures

The primary outcome was the development of a surgical site infection (superficial incisional, deep incisional, or organ space [deep prosthetic infection]) within 1 year of the date of surgery. Surgical site infections were classified according to the criteria established by the Centers for Disease Control and Prevention.^11^ Secondary outcomes included antibiotic-related complications, unplanned additional operations, death, and oncologic and functional outcomes within 1 year after surgical resection and endoprosthetic reconstruction. Validated functional assessments included the Musculoskeletal Tumor Society 1987 (MSTS-87) (range, 0-35, with higher scores indicating better function) and 1993 (MSTS-93) (range, 0-100, with higher scores indicating better function) scores and the Toronto Extremity Salvage Score (TESS) (range, 0-100, with higher scores indicating better function).^12,13,14^

Patients were assessed for study events by their treating surgeon at 2 and 6 weeks, 3, 6, and 9 months, and 1 year postoperatively. The functional assessments were completed before surgery and at the 1-year follow-up visit. The blinded Central Adjudication Committee adjudicated all primary and key secondary outcome events identified during the 1-year study follow-up as well as all instances when eligibility was in doubt.

### Statistical Analysis

The justification for the PARITY trial sample size has been previously published.^10,15^ At the trial’s onset, we calculated that the definitive sample size would require a total of 920 patients based on a between-group comparison of deep prosthetic infection. The sample size was calculated as a noninferiority trial under the assumption of an overall 10% event rate with an absolute difference of 5% in the risk of deep surgical site infection within 1 year to define noninferiority. After initiating enrollment and then transitioning from the vanguard to the definitive phase of the trial, we expanded the trial’s primary outcome from deep to any surgical site infection and changed the study’s design to a superiority trial to increase the expected event rate and feasibility without compromising clinical importance. The expanded definition of the primary outcome resulted in an overall vanguard phase event rate of 14%.^16^ Therefore, with a presumed 50% or greater reduction in the relative risk of deep surgical site infection within 1 year and with a 2-sided α of .05 and study power of 80%, we planned the definitive trial’s sample size to include 300 patients per arm, for a total of 600 patients.

When conducting the final analyses, we adhered to the published statistical analysis plan.^15^ Briefly, for the primary analysis, we used a Cox proportional hazards regression model with time from surgery to the surgical site infection as the primary outcome. The analysis included all patients in the groups to which they were randomly allocated. Postoperative antibiotic duration (treatment group) was the independent variable, and the Cox proportional hazards regression included tumor location and clinical site as stratification variables. Patients who did not experience the primary outcome were censored at 1 year or at the time of last study visit. We tested the proportional hazards assumption of the Cox proportional hazards regression model by examining Schoenfeld residuals. We also performed several sensitivity analyses for the primary outcome, including a competing risks analysis that accounted for deaths and amputation as competing risks. We identified the following 5 subgroups a priori, which we analyzed in the primary model to assess for possible effect modification: tumor type, tumor location, sex, age, and preoperative chemotherapy.

We assessed the effect of postoperative antibiotic duration on the secondary outcomes using Cox proportional hazards regressions with treatment group as the independent variable and tumor location and clinical site as stratification variables. We also estimated the effect of postoperative antibiotic duration on functional outcomes at 1 year using multiple linear regression models that included treatment group, tumor location, clinical site, and baseline score as independent variables. We used multiple imputation to address missing functional outcome data.

The results are presented as hazard ratios (HRs) for time-to-event outcomes and mean difference for continuous outcomes, with corresponding 95% CIs and associated 2-sided *P* values. No adjustments were made for multiple testing. Kaplan-Meier curves were constructed for the primary outcome. These analyses were first completed using blinded treatment groups by the data analyst (D.H.-A.). Interpretations for the effect of antibiotic duration were developed and documented based on blinded group A vs B. The randomization code was then broken, the correct a priori interpretation selected, and the manuscript drafted. All analyses were conducted using SAS software, version 9.4 (SAS Institute Inc).

## Results

### Study Patients

**Figure 1.**
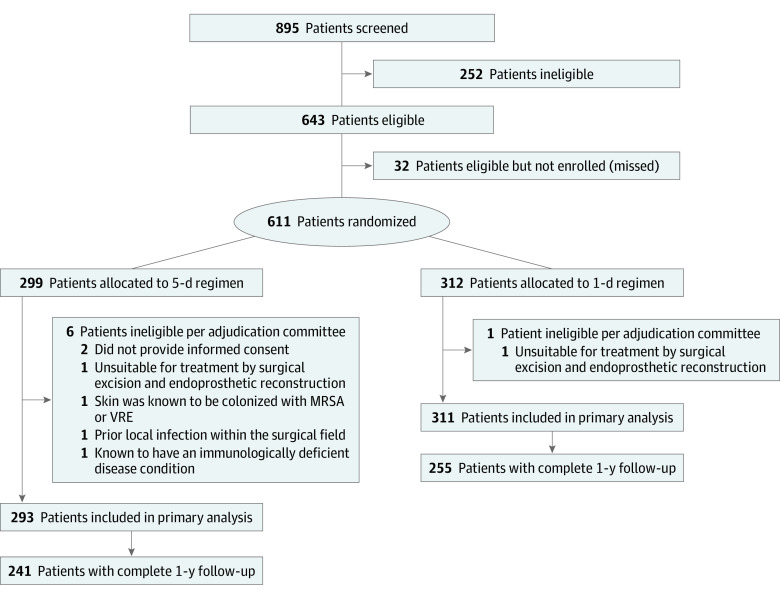
Patient Flow Diagram MRSA indicates methicillin-resistant *Staphylococcus aureus*; VRE, vancomycin-resistant *Enterococcus.*

Of the 604 patients included in the final analysis (mean [SD] age, 41.2 [21.9] years; 361 [59.8%] male; 114 [18.9%] Asian, 43 [7.1%] Black, 34 [5.6%] Hispanic, 15 [2.5%] Indigenous, 384 [63.8%] White, and 12 [2.0%] other [3 Middle Eastern, 5 mixed race, 2 North African, 1 Polynesian, and 1 Turkish]), 293 were randomized to a 5-day regimen and 311 to a 1-day regimen. Of the 527 patients alive at 1 year, 496 (94%) had 1-year follow-up data available. Figure 1 and the eAppendix (Section 5.0) and eTables 1 and 2 in Supplement 2 provide details regarding patient flow and the reasons for exclusion.

Table 1 and eTables 3 to 5 in Supplement 2 present patient characteristics and demonstrate similar characteristics in the 2 groups. The most common tumor type was a primary bone tumor located predominantly in the femur. A total of 290 patients (48.0%) received preoperative chemotherapy; 22 (3.6%) received preoperative radiotherapy. There was an imbalance between treatment groups, with 129 of 293 patients (44.0%) allocated to the 5-day regimen having received preoperative chemotherapy compared with 161 of 311 patients (51.8%) allocated to the 1-day regimen.

**Table 1.** Baseline Characteristics of the Study Patients^a^

Characteristic	5-d Regimen (n = 293)	1-d Regimen (n = 311)	Total (n = 604)
Patient demographic characteristics and tumor details			
Age, mean (SD), y	42.6 (21.7)	39.9 (22.0)	41.2 (21.9)
Sex			
Male	178 (60.8)	183 (58.8)	361 (59.8)
Female	115 (39.2)	128 (41.2)	243 (40.2)
Race and ethnicity			
Asian	54 (18.4)	60 (19.4)	114 (18.9)
Black	21 (7.2)	22 (7.1)	43 (7.1)
Hispanic	14 (4.8)	20 (6.5)	34 (5.6)
Indigenous	4 (1.4)	11 (3.6)	15 (2.5)
White	194 (66.2)	190 (61.5)	384 (63.8)
Other^b^	6 (2.0)	6 (1.9)	12 (2.0)
Unknown	0	2	2
Systemic metastases			
No	244 (83.3)	255 (82.0)	499 (82.6)
Yes	49 (16.7)	56 (18.0)	105 (17.4)
Other cancer treatment modalities			
No	157 (53.6)	138 (44.4)	295 (48.8)
Yes	136 (46.4)	173 (55.6)	309 (51.2)
Preoperative chemotherapy	129 (44.0)	161 (51.8)	290 (48.0)
Preoperative radiation	10 (3.4)	12 (3.9)	22 (3.6)
Other	7 (2.4)	7 (2.3)	14 (2.3)
Location of tumor			
Tibia	53 (18.1)	55 (17.7)	108 (17.9)
Femur	240 (81.9)	256 (82.3)	496 (82.1)
Type of tumor			
Bone tumor	237 (80.9)	249 (80.1)	486 (80.5)
Soft tissue sarcoma	28 (9.6)	34 (10.9)	62 (10.3)
Oligometastatic bone disease	28 (9.6)	28 (9.0)	56 (9.3)
Neutropenia at time of surgery^c^			
No. of patients	275	286	561
No	231 (84.0)	234 (81.8)	465 (82.9)
Yes	44 (16.0)	52 (18.2)	96 (17.1)
Surgical and perioperative management details			
Length of procedure, median (Q1-Q3), min	270 (206-377)	270 (200-377)	270 (205-377)
Antibiotic or silver-coated prosthesis			
No. of patients	292	311	603
No	276 (94.5)	295 (94.9)	571 (94.7)
Yes	16 (5.5)	16 (5.1)	32 (5.3)
Antibiotic	6 (2.1)	6 (1.9)	12 (2.0)
Silver-coated prosthesis	10 (3.4)	10 (3.2)	20 (3.3)
Suction drain used			
No. of patients	293	310	603
No	63 (21.5)	74 (23.9)	137 (22.7)
Yes	230 (78.5)	236 (76.1)	466 (77.3)

Abbreviation: Q1-Q3, quartile 1 to quartile 3.

^a^ Data are presented as number (percentage) of patients unless otherwise indicated.

^b^ Other ethnicity includes Middle Eastern (n = 3), mixed race (n = 5), North African (n = 2), Polynesian (n = 1), and Turkish (n = 1).

^c^ Absolute neutrophil count of 1500/μL or less (to convert to ×10^9^/L, multiply by 0.001).

### Adherence to the Allocated Intervention

Two patients (0.6%) who had originally been allocated to a 1-day regimen received a 5-day regimen; no patients originally allocated to a 5-day regimen crossed over to a 1-day regimen. Protocol deviations primarily resulted from inpatient hospital discharge earlier than 5 days postoperatively and were similar between groups. Of those allocated to the 5-day regimen, 248 patients (84.6%) received all but the final 3 doses of intravenous infusions as did 256 patients (82.6%) allocated to the 1-day regimen. eTable 6 in Supplement 2 provides details regarding the administration of the antibiotics.

### Primary Outcome

**Figure 2.**
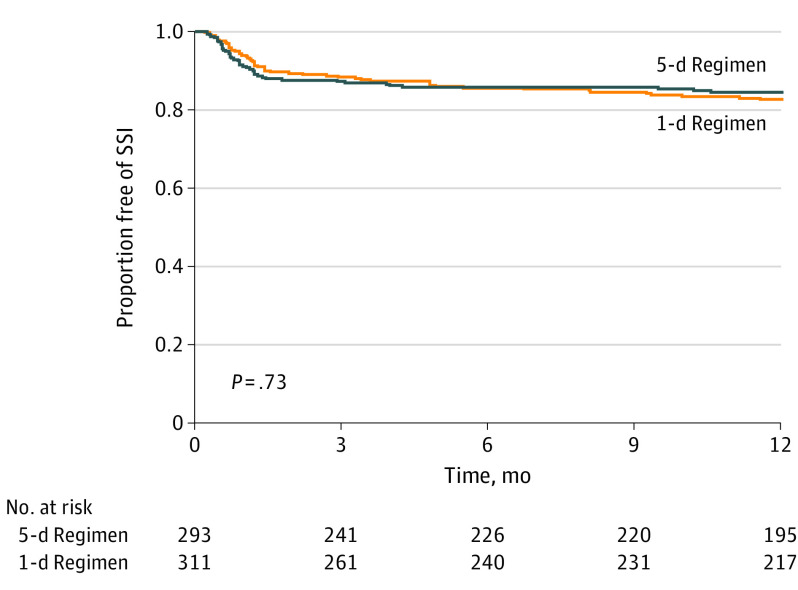
Kaplan-Meier Estimates of the Duration of Primary Outcome-Free Survival at the Time of Final Assessment SSI indicates surgical site infection.

A surgical site infection occurred within 1 year in 44 of 293 patients (15.0%) allocated to the 5-day regimen and in 52 of 311 patients (16.7%) allocated to the 1-day regimen (HR, 0.93; 95% CI, 0.62-1.40; *P* = .73) (Figure 2 and Table 2). The Schoenfeld residuals demonstrate that the assumption of proportional hazards for the primary outcome was not violated. Table 2 presents the incidence of superficial incisional, deep incisional, and organ space surgical site infections for each group. The most common causative organisms of surgical site infections were *S aureus* and coagulase-negative staphylococci.

**Table 2.** Study Outcomes by Treatment Group (Primary and Secondary)

Study end point	5-d Regimen (n = 293)	1-d Regimen (n = 311)	HR (95% CI)	*P* value
**Primary outcome**				
Any surgical site infection	44 (15.0)	52 (16.7)	0.93 (0.62-1.40)	.73
Superficial incisional	13 (4.4)	12 (3.9)	NR	NR
Deep incisional	3 (1.0)	8 (2.6)	NR	NR
Organ or space	28 (9.6)	34 (10.9)	0.97 (0.59-1.62)	.92
**Secondary outcomes**				
Any antibiotic-related complications	15 (5.1)	5 (1.6)	3.24 (1.17-8.98)	.02
*Clostridioides difficile*–associated colitis	11 (3.8)	4 (1.3)	NR	NR
Opportunistic fungal infection	0	1 (0.3)	NR	NR
Oral candidiasis	1 (0.3)	0	NR	NR
Diarrhea (unrelated to *C difficile*) that required intervention	3 (1.0)	0	NR	NR
Any unplanned additional operation	75 (25.6)	80 (25.7)	1.06 (0.77-1.46)	.72
Any oncologic events	85 (29.0)	89 (28.6)	1.02 (0.75-1.39)	.90
Local recurrence	15 (5.1)	22 (7.1)	0.78 (0.40-1.51)	.46
Distant metastases	69 (23.5)	79 (25.4)	0.90 (0.65-1.25)	.53
Other oncologic event	7 (2.4)	8 (2.6)	NR	NR
All-cause mortality	37 (12.6)	40 (12.9)	1.01 (0.64-1.58)	.98
Death from disease progression	29 (9.9)	29 (9.3)	1.08 (0.64-1.81)	.78

Abbreviations: HR, hazard ratio; NR, not reported (these study end points did not meet the threshold set in the Statistical Analysis Plan for the minimum number of events required to conduct a statistical comparison).

**Figure 3.**
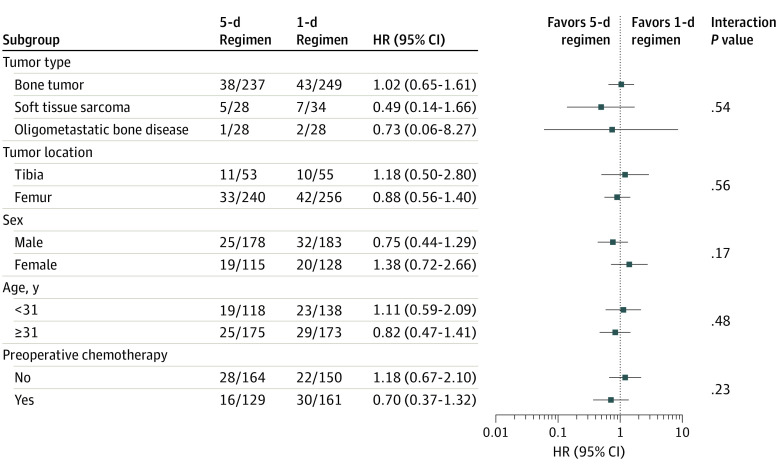
Forest Plot for Subgroup Analyses HR indicates hazard ratio.

The results were similar in the sensitivity analyses that accounted for deaths and amputation as competing risks. Adjusted analyses yielded similar results to those in the primary analysis. Subgroup analyses did not show any effect modification, including when adjusted for preoperative chemotherapy. Figure 3 and eTables 7 and 8 in Supplement 2 provide details regarding the sensitivity and subgroup analyses.

### Secondary Outcomes

#### Antibiotic-Related Complications, Unplanned Additional Operations, Oncologic Events, and Mortality

Study-related antibiotic-related complications occurred in 15 patients (5.1%) allocated to the 5-day regimen and in 5 patients (1.6%) allocated to the 1-day regimen (HR, 3.24; 95% CI, 1.17-8.98; *P* = .02) (Table 2). The most common antibiotic-related complication was *Clostridioides difficile*–associated colitis (Table 2). Unplanned additional operations occurred in 75 patients (25.6%) allocated to the 5-day regimen and in 80 patients (25.7%) allocated to the 1-day regimen (HR, 1.06; 95% CI, 0.77-1.46) (eTable 9 in Supplement 2). The most common types of additional operations were irrigation and debridement and implant exchange, most of which were to treat a deep incisional or organ space surgical site infection. Oncologic events (HR, 1.02; 95% CI, 0.75-1.39) and mortality (HR, 1.01; 95% CI, 0.64-1.58) proved similar between the treatment groups (Table 2 and eTable 9 in Supplement 2).

#### Functional Outcomes

Surgeon-reported function was similar between the treatment groups as measured by the MSTS-87 scores (mean difference, −0.49; *P* = .41) and MSTS-93 scores (mean difference, −1.89; *P* = .34) as was patient-reported function between the 2 groups as measured by the TESS (mean difference, 0.10; *P* = .96) (eTable 10 in Supplement 2).

## Discussion

The PARITY randomized clinical trial failed to demonstrate a benefit of a 5-day prophylactic antibiotic regimen of intravenous cephalosporins compared with a 1-day regimen in reducing surgical site infections after surgical resection and endoprosthetic reconstruction for a lower extremity bone tumor. However, 3 times as many patients allocated to the 5-day regimen experienced serious antibiotic-related complications.

Our overall primary event rate is higher than previously published studies in the field.^5,17^ In a meta-analysis^5^ of retrospective data, the surgical site infection rate after lower extremity endoprosthetic reconstruction was 10% (95% CI, 8%-11%). This meta-analysis^5^ suggested that antibiotic prophylaxis for longer than 24 hours postoperatively decreases the risk of infection, but the biases of observational research leave only low-quality evidence. Our event rate may be higher than previously reported because of our broader definition of a surgical site infection and the careful prospective collection of study event data.

Perioperative antibiotic prophylaxis is considered essential in minimizing surgical site infections in total joint arthroplasty, in which the infection rate is much lower.^18,19,20,21,22^ A meta-analysis^23^ of randomized clinical trials that compared only preoperative antibiotics with both preoperative and postoperative antibiotics in joint replacement surgery did not show efficacy of postoperative antibiotic prophylaxis. Because there is no evidence of additional benefit to a lengthened course, the Surgical Infection Prevention Project recommends the discontinuation of use of prophylactic antibiotics 24 hours postoperatively for all surgical cases.^24^ However, lower limb oncologic reconstructions are unique in their complexity and the immunocompromised state of the affected patient population; therefore, these recommendations may not be applicable in this particular setting. The second International Consensus Meeting on Musculoskeletal Infection recently identified whether prolonging use of postoperative antibiotics would prevent surgical site infections in patients with bone tumors undergoing endoprosthetic reconstruction as 1 of the most critical orthopedic oncology clinical questions.^25^ Their recommendation acknowledged that although more than half of orthopedic oncologists prescribe prophylactic antibiotics for longer than 24 hours postoperatively, there is insufficient evidence to support this practice.^25^

The overuse of antibiotics is a major public health concern associated with increased health care costs from antibiotic-related complications and antibiotic resistance.^26^ The increase in antibiotic-resistant organisms, particularly in a nosocomial setting, is outpacing the development of new antimicrobial agents. Antibiotic use often results in antibiotic-associated diarrhea, not infrequently caused by *C difficile*. Although antibiotic-associated diarrhea is generally mild and self-limiting, gut infection with *C difficile* is universally severe and may lead to toxic megacolon, organ failure, or even death.^27^ Our study found a significant increase in antibiotic-related complications in the 5-day regimen group, with most complications reported to be *C difficile*–associated colitis. To avoid this complication, hospital antibiotic stewardship programs in the UK have restricted the use of high-risk antibiotics, including cephalosporins, with a subsequent clear reduction of nosocomial *C difficile* infections.^28,29,30^

### Strengths and Limitations

Our trial has several strengths. Safeguards against potential bias included concealed randomization and blinding of treatment allocation from patients, caregivers, outcomes assessors, and data analysts. The diagnosis of surgical site infection was independently adjudicated using well-established definitions, thus ensuring objective decision-making and minimizing outcome assessment bias. By documenting our interpretations based on blinded results before breaking the randomization code, we safeguarded against interpretation bias.^31^ After accounting for the nearly 10% mortality rate, true loss to follow-up was only 5%. Close clinical surveillance and rigorous study monitoring procedures resulted in this relatively small loss to follow-up. Our study’s broad eligibility criteria and conduct in many health care systems strengthens its generalizability. Several of the study outcomes are of unequivocal importance to both patients and health care systems. Finally, answering clinical questions in rare conditions requires an immense international, collaborative effort. To our knowledge, this trial was the first-ever collaborative, interventional trial initiated and led by orthopedic oncologists.

Our trial also has limitations. The large number of participating clinical sites, combined with the rarity of bone tumors, resulted in low enrollment at some sites. More patients in the shorter duration group received preoperative chemotherapy, which may have increased their risk of a surgical site infection. However, our subgroup and adjusted analyses yielded results similar to our primary analysis. Protocol deviations, particularly doses not administered because of early hospital discharge, were not uncommon. Although the deviations were balanced between groups, they would have affected the 5-day regimen group more than the 1-day regimen. However, the discontinuation of antibiotics at hospital discharge is standard procedure; thus, our results are applicable to actual clinical practice. The impact of the postoperative prophylactic antibiotic regimens on late infections that present after the 1-year postsurgery timepoint cannot be inferred from this study; however, these infections are not considered surgical site infections according to the definitions established by the Centers for Disease Control and Prevention. The diagnosis of a surgical site infection is challenging because of the complexities of surgery and wound healing, a concern ameliorated by the rigor and blinding of the adjudication process. Finally, the lack of a statistically significant difference observed may be the result of insufficient study power. However, the observed absolute difference between the 2 treatment groups was small, and the increased sample size needed to show statistical significance would likely not be considered necessary by surgeons in the field.

## Conclusions

In this randomized clinical trial, the 5-day regimen did not reduce the rate of surgical site infection compared with the 1-day regimen. Although the results of this randomized clinical trial do not definitively exclude an important benefit of postoperative prophylactic antibiotic therapy longer than 24 hours, the significantly higher risk of antibiotic-related complications in the 5-day regimen represents a critical finding. Clinicians should consider the uncertainty of the benefits and the relative confidence in findings of harm to make an informed decision on antibiotic duration after surgical resection and endoprosthetic reconstruction for lower extremity bone tumors.

## Notes

Supplement 1.
Trial Protocol

Supplement 2. **eAppendix.** Supplemental Methods **eTable 1.** Reasons for Exclusion Prior to Randomization **eTable 2.** Reasons for Exclusion After Adjudication Committee Review **eTable 3.** Patient Demographics and Baseline Details **eTable 4.** Tumor Details **eTable 5.** Surgical and Perioperative Management Details **eTable 6.** Prophylactic Antibiotic Administration Details **eTable 7.** Sensitivity Analyses **eTable 8.** Subgroup Analyses **eTable 9.** Study Outcomes by Treatment Group (Primary and Secondary) **eTable 10.** Functional and Quality of Life Outcomes by Treatment Group

Supplement 3. **Nonauthor Collaborators.** The PARITY Investigators

Supplement 4.
Data Sharing Statement
