# Supplementary material for: Comparison of Prophylactic Intravenous Antibiotic Regimens After Endoprosthetic Reconstruction for Lower Extremity Bone Tumors: A Randomized Clinical Trial
Source: JAMA Oncol. 2022 Jan 6;8(3):345–53. doi: 10.1001/jamaoncol.2021.6628 (PMC8739829; doi:10.1001/jamaoncol.2021.6628)
Supplement: Supplement 1. — Trial Protocol [file jamaoncol-e216628-s001.pdf]

This supplement contains the following items:

1. Initial protocol (Version 1.0), final protocol (Version 6.0 [cefazolin] / 6.1 [cefuroxime]), summary of changes; and
2. The statistical analysis plan (Version 1.0) [no amendments].

Protocol Version 1.0

(Follows)

PARITY

Prophylactic Antibiotic Regimens in Tumor Surgery (PARITY): A Multi-Center Randomized Controlled Study Comparing Alternative Antibiotic Regimens in Patients Undergoing Tumor Resections with Endoprosthetic Replacements

Methods Centre:

Center for Evidence-Based Orthopaedics (CEO)
Master University
293 Wellington Street North
Suite 110
Hamilton, Ontario L8L 8E7
Tel: 905-527-4322 x44490
Fax: 905-523-8781
Email: racanoa@mcmaster.ca

Funding Sponsors:

Orthopaedic Research & Education Foundation / Muskuloskeletal Tumor Society (OREF/MSTS) (*applied*)
PSI (*applied*)
Society of Surgical Oncology (*application in process*)

Date: 20-December-2011

Version: 1.0

STEERING COMMITTEE:

CHAIR:<br>Michelle Ghert, MD, FRCSC<br>McMaster University<br>Juravinski Hospital and Cancer Centre<br>Department of Surgery<br>699 Concession Street<br>Hamilton, ON L8V 5C2<br>Tel: 905-387-9495 ext. 64089<br>Fax: 905-575-6343<br>Email: michelle.ghert@jcc.hhsc.ca	Mohit Bhandari, MD, MSc, FRCSC<br>McMaster University<br>Department of Surgery<br>Department of Clinical Epidemiology & Biostatistics<br>293 Wellington Street North, Suite 110<br>Hamilton, ON L8L 8E7<br>Tel: 905-527-4322 ext.44490<br>Fax: 905-523-8781<br>Email: bhandam@mcmaster.ca
Ginger Holt, MD<br>Vanderbilt University Medical Center<br>Department of Orthopaedics and Rehabilitation<br>1215 21st Avenue South, Room 4200<br>Nashville, TN, USA 37232-8828<br>Tel: 615-936-5363<br>Fax: 615-936-2667<br>Email: ginger.e.holt@Vanderbilt.Edu	Benjamin Deheshi, MD, MSc, FRCSC<br>McMaster University<br>Juravinski Hospital and Cancer Centre<br>Department of Surgery<br>711 Concession Street<br>Hamilton, ON<br>Tel: 905-521-2100 ext. 43962<br>Fax: 905-381-7062<br>Email: deheshi@jcc.hhsc.ca
Peter Ferguson, MD, MSc, FRCSC<br>University of Toronto<br>Mount Sinai Hospital<br>600 University Avenue, Suite 476G<br>Toronto, ON M5G 1X5<br>Tel: 416-586-4800 ext. 8687<br>Fax: 416-586-8397<br>Email: pferguson@mtsinai.on.ca	Jay Wunder, MD, MSc, FRCSC<br>University of Toronto<br>Mount Sinai Hospital<br>600 University Avenue, Suite 476E<br>Toronto, ON M5G 1X5<br>Tel: 416-586-4800 ext. 5995<br>Fax: 416-586-8397<br>Email: wunder@lunenfeld.ca
Gordon Guyatt, MD, MSc<br>McMaster University<br>Department of Clinical Epidemiology & Biostatistics<br>1200 Main Street West, Rm. 2C12<br>Hamilton, ON L8N 3Z5<br>Tel: 905-525-9140 ext.95287<br>Fax: 905-524-3841<br>Email: guyatt@mcmaster.ca	Stephen Walter, PhD<br>McMaster University<br>Department of Clinical Epidemiology & Biostatistics<br>1200 Main Street West, Rm. 2C16<br>Hamilton, ON L8N 3Z5<br>Tel: 905-525-9140 ext.22338<br>Fax: 905-529-3012<br>Email: walter@mcmaster.ca

TABLE OF CONTENTS

List of Abbreviations v

Study Summary 1

1 Introduction 2

1.1 Background *2*

1.2 Preclinical Data *2*

1.2.1 Best Evidence for Infection Rates 2

1.2.2 Lack of consensus in Antibiotic Regimens and Global Interest in a Randomized Trial 4

1.2.3 Complications of Antibiotic Overuse 5

2 Study Objectives 6

2.1 Primary Questions *6*

2.2 Secondary Questions *6*

3 Study Design 6

All eligible patients who consent to the trial 7

3.1 Rationale for Design *8*

3.2 Primary Study Endpoints *8*

3.3 Secondary Study Endpoints *9*

4 Subject Selection and Withdrawal 9

4.1 Inclusion Criteria *9*

4.2 Exclusion Criteria *9*

4.3 Subject Recruitment and Screening *10*

4.4 Early Withdrawal of Subjects *11*

4.4.1 When and How to Withdraw Subjects 11

4.4.2 Data Collection and Follow-up for Withdrawn Subjects 11

5 Study Interventions 11

5.1 Randomization Methods *11*

5.2 Antibiotic Regimens *12*

5.3 Blinding *12*

6 Study Procedures 12

6.1 Patient Screening and Consent 12
6.2 Randomization 12
6.3 Surgical Treatment 13
6.4 2 Week Follow-up 13
6.5 4 Week Follow-up 13
6.6 3 Month Follow-up 13
6.7 6 Month Follow-up 14
6.8 9 Month Follow-up 14
6.9 1 Year Follow-up 14
6.10 Maximization of Follow-up 14
6.11 Minimization of Crossovers of Surgical Interventions 15
6.12 Adjudication Requirements 15

7 Statistical Plan 16
7.1 Sample Size Determination 16
7.2 Statistical Methods 18

8 Safety and Adverse Events 18
8.1 Definitions 18
8.1.1 Adverse Event 18
8.1.2 Serious Adverse Event 18
8.1.3 Unanticipated Problems Resulting in Risk to Subjects or Others 19
8.2 Reporting of Serious Adverse Events and Unanticipated Problems Resulting in Risk to Subjects or Others 19
8.2.2 Investigator Reporting: Notifying the Methods Center 19
8.2.3 Site Investigator — IRB/REB Reporting 20
8.2.4 Data Monitoring Committee (DMC) 20

9 Data Handling and Record Keeping 20
9.1 Confidentiality 20
9.2 Case Report Forms 20

10 Ethical Considerations 21

11 References 21

List of Abbreviations

Abbreviations are listed in alphabetical order

AE: adverse event
CAC: Central Adjudication Committee
CDC: Center for Disease and Control
CRF: case report form
DMC: Data Monitoring Committee
FDA: Food and Drug Administration
GCP: Good Clinical Practice
HIPAA: Health Insurance Portability and Accountability Act
IRB: Institutional Review Board
MSTS: Musculoskeletal Tumor Society questionnaire
PHI: protected health information
RCT: randomized controlled trial
REB: Research Ethics Board
SAE: serious adverse event
SSI: Surgical Site Infection
TESS: Toronto Extremity Salvage Score

Study Summary

Title	Prophylactic Antibiotic Regimens in Tumor Surgery (PARITY): A Multi-Center Randomized Controlled Study Comparing Alternative Antibiotics Regimens in Patients Undergoing Tumor Resections with Endoprosthetic Replacements
Short Title	PARITY
Methodology	Multi-center, Blinded, Randomized Trial
Study Duration	July 2012 to June 2014
Study Center(s)	Multi-Center
Primary Study Question	In patients undergoing surgical excision and endoprosthetic reconstruction of a lower extremity bone tumor, is there any difference in the effect of postoperative antibiotic regimens (24 hours vs. 5 days) on infection rate outcomes?
Number of Subjects	100
Diagnosis and Main Inclusion Criteria	Primary malignant or aggressive benign bone tumors of the lower extremities requiring surgical excision and endoprosthetic reconstruction
Study Product, Dose, Route, Regimen	Antibiotic regimens: intravenous cephalosporin for 24 hours and 5 days

1 Introduction

This document is a protocol for a multi-center, blinded, randomized controlled trial, using a parallel two-arm design, to investigate whether postoperative antibiotic regimens (24 hours vs. 5 days) will decrease the rate of infection among patients being surgically treated for a lower extremity bone tumor. The rationale for this study is fuelled by: 1) increased infection rate outcomes in bone tumor surgery compared to general arthroplasty; 2) a lack of consensus among Orthopaedic Oncologists regarding the most effective prophylactic antibiotic regimen; 3) a lack of randomized controlled trial (RCT) evidence; and 4) extensive investigator support for the proposed trial.

1.1 Background

Limb salvage surgery is the standard of care in the management of sarcoma of the long-bones. Advances in chemotherapeutic regimens and imaging techniques allow for wide resection and functional reconstruction in the 95% of patients. The most common type of long-bone reconstruction involves the use of a tumor prosthesis, or endoprostheses. Due to the complexity and length of surgical resection and reconstruction, as well as the immunocompromised nature of patients treated with chemotherapy, the risk for infection remains high.[1, 2] Deep infection following endoprosthetic reconstruction is a devastating complication that requires staged revision surgery and long-term intravenous antibiotics.[3] The risk for subsequent infection remains high, as does the risk for ultimate amputation.[1, 4, 5] However, the most effective antibiotic regimen in preventing postoperative deep infections remains controversial, and the current state of practice varies widely, particularly with respect to antibiotic duration. Moreover, patients' quality of life and function following infection are dramatically impacted, as are health care costs.[6] Strategies to optimize prevention of infection and quality of life, while mitigating health care costs are needed.

1.2 Preclinical Data

1.2.1 Best Evidence for Infection Rates

We performed a systematic review comparing the infection rate outcomes reported following the surgical treatment of primary long bone tumors (malignant and benign aggressive) by excision and endoprosthetic reconstruction. A systematic literature search was conducted of the Medline, EMBASE, and all EBM Reviews (including Cochrane) databases. The proceedings for past American Society for Clinical Oncology (ASCO) Annual Meetings were also searched. The search was limited to articles published in the English language, and no restrictions were placed on dates of publication.

Our initial search generated 3889 titles. All titles and relevant abstracts were independently screened by two reviewers in order to minimize bias and ensure that

studies were not overlooked. Based on this first screen, only those titles and abstracts that discussed the use of endoprosthetic reconstructions in the treatment of a long bone sarcomas (malignant or aggressive benign) were selected. Studies that reported the use of allografts or allograft-prosthesis composites were excluded. From the full-text articles selected, only those papers that examined primary lesions of the lower extremities in skeletally mature patients were considered for further review. Papers that included soft-tissue sarcomas were excluded, as were those that included metastatic lesions, recurrent lesions, or lesions that had received prior surgical treatment. The reported infection rates were then extracted from the remaining papers and compared. Data extraction and assessment of data quality was performed independently by both reviewers. Differences were reconciled by mutual agreement, or by a third party.

Of the 3889 titles, we identified 50 eligible papers which are listed below in **Table 1**. The deep infection rates ranged from 0% to 22.2% with a weighted mean of 9.0% (95% confidence interval: 7.4% to 10.7%). Those papers that reported antibiotic regimens varied significantly from ‘intra-operative dosing only’ to ‘greater than 72 hours’.[7-9]

Table 1: Deep Infection Rates Reported by Systemati Review

Study	Year	Number	Deep infection rate
Lee et al.	1990	17	0.0%
Roberts et al.	1991	133	5.3%
Horowitz et al.	1991	12	25.0%
Eckardt et al.	1991	68	1.5%
Shih et al.	1993	61	6.6%
Morris et al.	1995	31	3.2%
Malawer et al.	1995	51	19.6%
Zehr et al.	1996	17	5.9%
Abudu et al.	1996	16	0.0%
Abudu et al.	1999	5	20.0%
Lee et al.	1999	6	16.7%
Grimer et al.	1999	151	18.5%
Kawai et al.	1999	32	6.3%
Kabukcuoglu et al.	1999	54	1.9%
Natarajan et al.	2000	6	16.7%
Ilyas et al.	2000	15	13.3%
Ilyas et al.	2001	48	8.3%
Donati et al.	2001	25	4.2%
Wunder et al.	2001	64	6.3%
Sewell et al.	2001	18	5.6%
Sokolov	2002	38	10.5%
Lee et al.	2002	145	11.0%

Ilyas et al.	2002	15	6.7%
Bickels et al.	2002	110	5.5%
Antract et al.	2002	9	22.2%
Griffin et al.	2005	99	10.1%
Natarajan et al.	2005	246	6.9%
Jeys et al.	2005	1036	11.9%
Sharma et al.	2006	77	7.8%
Farid et al.	2006	52	3.8%
Orlic et al.	2006	82	4.9%
Ahlmann et al.	2006	211	5.2%
Gosheger et al.	2006	250	12.0%
Sharma et al.	2007	112	9.8%
Myers et al.	2007	194	19.6%
Sim et al.	2007	50	12.00%
Finstein et al.	2007	62	4.80%
Myers et al.	2007	335	9.6%
Akahane et al.	2007	11	9.1%
Gitelis et al.	2008	80	2.5%
Guo et al.	2008	104	6.7%
Jeys et al.	2008	530	12.8%
Sewell et al.	2009	22	0.0%
Natarajan et al.	2009	17	11.8%
Shekkeris et al.	2009	6	16.7%
Chandrasekar et al.	2009	100	2.0%
Lee et al.	2009	256	9.8%
Morii et al.	2010	82	12.2%
Hanna et al.	2010	23	5.6%
Hardes et al.	2010	125	12.80%
Li et al.	2011	49	2.0%

1.2.2 Lack of consensus in Antibiotic Regimens and Global Interest in a Randomized Trial

We conducted a survey addressing the practices of Orthopaedic Oncologists registered with the Muscuoloskeletal Tumor Society (MSTS) and the Canadian Orthopaedic Oncology Association (CANOOS). From this survey, we concluded that there is currently a lack of guidelines for the prescription of prophylactic antibiotics in Musculoskeletal Tumor Surgery, which has left Orthopaedic Oncologists with varying opinions and practices.[10] Of the 97 surgeons who received the questionnaire, 72 responded (75% response rate (% CI: 65.5, 82.5%)). While almost all respondents agreed antibiotic regimens were important in reducing the risk of infection, respondents varied considerably in their choices of antibiotic regimens and dosages. Although 73%

(95% CI: 61, 82%) of respondents prescribe a first generation cephalosporin, one in four favours additional coverage with an aminoglycoside and/or Vancomycin. One in three surgeons (95% CI: 25, 48%) believes antibiotics should be discontinued after 24 hours (as recommended by the AAOS for total joint arthroplasty[11]) but 40% (95% CI: 30, 53%) continue antibiotics until the suction drain is removed.

Given the ongoing uncertainty in evidence to guide best practices, 90% (95% CI: 81, 95%) of respondents agreed that they would change their practice if a large randomized controlled trial showed clear benefit of an antibiotic drug regimen different from what they are currently using. Further support for a clinical trial was observed by an overwhelming surgeon interest (87%; 95% CI: 77, 93%) in participating in a multi-center randomized controlled study.

1.2.3 Complications of Antibiotic Overuse

Antibiotic resistance is an increasingly clinically relevant issue both in surgical and infectious disease literature. The Canadian Antibiotic Resistance Alliance (CARA) publishes statistics intended for use by infectious disease physicians and other medical and surgical specialists.[12] Our systematic review shows that the most common infective pathogen was *staph aureus*. The 2009 Canadian antibiotogram shows that 100% of MSSA (*methicillin sensitive staphylococcus aureus*) is susceptible to cefazolin (Ancef).[12] However, the prevalence of MRSA versus MSSA varies by institution and patient population. Zhanel et al. shows that MRSA comprised 27.0% of all *S. aureus* isolates (68.8% were health care associated [HA-MRSA] and 27.6% were community associated [CA-MRSA]).[12] One hundred percent of both community-associated and health care- associated MRSA showed susceptibility to vancomycin and varying susceptibilities to other antimicrobials. Furthermore, prevalence of antibiotic resistance is increasing in Canada. Data from the Canadian Nosocomial Infection Surveillance Program show that the incidence of MRSA as a proportion of all *S. Aureus* has increased from 1% in 1995 to 8% by 2000 and 27% in 2008 as mentioned above.[12]

The vast majority of prosthetic infections are due to gram positive bacteria, and Ancef also exhibits gram negative coverage. Notably, the CANWARD 2009 antibiotogram shows 37.6% of *E. coli* and 47.6% of *Klebsiella pneumonia* are susceptible to cefazolin.[12] Based on an expert panel of six Orthopaedic Oncologists and three Infectious Disease specialists who were consulted in preparation for this study, it was determined that the ideal study would be a non-inferiority trial comparing the efficacy of 2g of Ancef given intravenously every 8 hours for either 24 hours (short duration) or 5 days or until discharge from acute care (long duration). Despite the fact that 11% of respondents in the PARITY Survey responded that they prescribe an aminoglycoside, the Infectious Disease experts on our panel agreed that this type of gram negative coverage does not add more gram negative coverage to that already provided by Ancef.

In addition, our PARITY survey indicated that there is significant concern in the community regarding nephrotoxicity and ototoxicity associated with aminoglycosides.[10]

Antibiotic misuse and overuse in terms of spectrum and duration respectively are considered the main factors in development of antibiotic resistance.[13] When threatened, bacteria evolve to survive, the main mechanisms being genetic mutation, expression of latent resistance genes, or acquisition of genes with resistance determinants.[13] If the antibiotic resistance profile outruns the development of new antibiotics, we are left defenseless against prosthetic infections, which will significantly impact our ability to salvage infected tumor prosthesis and therefore adversely affect patient morbidity and mortality. In addition to the medium to long term effects of development of antibiotic resistance, a long course of antibiotics itself is not benign. Complications can vary from an inconvenience to a fatality. Possible complications include the development of *clostridium difficile* diarrhea and toxic megacolon, opportunistic fungal infections, catheter related infections, and seizures.[14-18]

2 Study Objectives

The objective of this study is to determine the effects of postoperative antibiotic regimens (24 hours vs. 5 days) on infection rate outcomes of lower extremity tumor surgery. This objective will be carried out by answering the following questions:

2.1 Primary Questions

In patients undergoing surgical excision and endoprosthetic reconstruction of a lower extremity bone tumor, is there any difference in the effect of postoperative antibiotic regimens (24 hours vs. 5 days) on infection rate outcomes?

2.2 Secondary Questions

In patients surgically treated for bone tumors of the lower extremities followed by limb reconstruction using an endoprosthesis, what is the impact of the postoperative antibiotic regimen (24 hours vs. 5 days) on the development of antibiotic-related complications (ie: gastrointestinal infections, fungal infections, etc.) and on patient functional outcome and quality of life after one year?

3 Study Design

This study is a multi-center, blinded, randomized controlled trial, using a parallel two-arm design to investigate whether postoperative antibiotic regimens (24 hours vs. 5 days) will decrease the rate of infection among patients being surgically treated for a lower extremity bone tumor.

Patients are randomized using a 24 hour toll-free computerized randomization system that allows random variable block sizes to one of two treatment arms (24 hours or 5 days). The randomization is stratified by: 1) center, 2) location of tumor (femur vs. tibia), and 3) perioperative chemotherapy (yes or no). The period of patient enrolment is approximately 1 year and the enrolled patients will be followed for 1 year after surgery. We will assess infection rates within 12 months after initial surgery across both study arms. Patients, outcome adjudicators and data analysts will be blinded. We will measure function and quality of life at 2 weeks, 4 weeks, 3 months, 6 months, 9 months, and 1 year. The schematic procedure is shown in **Figure 1**.

Figure 1: Trial Conduct Procedure

Patient Recruitment, Randomization and Surgical Interventions

Identification of Patients	Direct referral-within center	Data Collected
	↓	
Assessment of Patient Eligibility	Study explanation<br>History-review eligibility criteria, and other relevant medical conditions<br>Physical Examination<br>Radiographs	Screening Form
	Informed Consent, if eligible	Informed Consent
	↓ All eligible patients who consent to the trial	
Randomization	24 hour web-based or telephone<br>Eligibility criteria reviewed again<br>Key patient information recorded<br>Randomization issued to patient	Baseline Form<br>Randomization Form
	↓	
Surgery	Either short or long arm<br>Surgical protocols will be followed	Surgical Form
	↓	

Follow Up Schedule

2 Weeks	Assessment of outcome events	Follow-Up Form<br>MSTS, TESS
4 Weeks	Assessment of outcome events	Follow-Up Form<br>MSTS, TESS

3 Months	Assessment of outcome events	Follow-Up Form<br>MSTS, TESS
6 Months	Assessment of outcome events	Follow-Up Form<br>MSTS, TESS
9 Months	Assessment of outcome events	Follow-Up Form<br>MSTS, TESS
1 Year	Assessment of outcome events	Follow-Up Form<br>MSTS, TESS

**Follow Up Forms include AEs, SAEs, infections, reoperations, protocol deviations or wound healing problems, and other appropriate forms*.

3.1 Rationale for Design

Deep infection following endoprosthetic limb reconstruction for sarcoma of the long bones is a devastating complication. We conducted a survey and a systematic review and found that there are no current best practice guidelines for antibiotic prophylaxis in tumor surgery and that Orthopaedic Oncologists would be interested in enrolling patients in research to inform the development of such guidelines.[10] These findings provide a strong rationale for undertaking a randomized control trial to determine the effects of postoperative antibiotic regimens on infection rate outcomes following bone tumor surgeries of the lower extremities. Implications of this trial may include both fewer endoprosthetic infections as well as fewer antibiotic related complications.

3.2 Primary Study Endpoints

The primary study endpoint is the development of a deep surgical site infection (SSI) within 12 months following the initial surgery to treat a primary bone tumor of the lower extremities. Patients will be monitored regularly by the treating physician at 2 weeks, 4 weeks, 3 months, 6 months, 9 months, and 1 year.

Deep infections will be classified according to the criteria established by the Center for Disease Control (CDC).[19] The CDC defines a deep SSI as infection occurring within the 30 days following the operative procedure or within 1 year if an implant is in place and the infection appears to be related to the operative procedure. Infection can involve any part of the body, but excludes the skin incision, fascia, or muscle layers, that is opened or manipulated during the operative procedure. The patient must also present with at least one of the following:

- purulent drainage from a drain that is placed through a stab wound into the organ/space

- organisms isolated from an aseptically obtained culture of fluid or tissue in the organ/space
- an abscess or other evidence of infection involving the organ/space that is found on direct examination, during reoperation, or by histopathologic or radiologic examination
- diagnosis of an organ/space SSI by a surgeon or attending physician.

A blinded Central Adjudication Committee (CAC) will judge whether the primary study endpoint has occurred. The CAC will be comprised of 3-4 orthopaedic surgeons and 1 infectious disease specialist, all of whom are independent from the study's investigative team.

3.3 Secondary Study Endpoints

The secondary study endpoints include patients' functional outcome and quality of life, as well as antibiotic-related complications. Questionnaires will be used to assess both functional outcome and quality of life at each of the above-noted follow-up time points. Questionnaires include the Musculoskeletal Tumor Society functional score (MSTS) and the Toronto Extremity Salvage Score (TESS). The MSTS and TESS surveys are based on the commonly accepted functional scoring systems in Orthopaedic Oncology publications.[20-22] Antibiotic-related complications, such as gastrointestinal infections, fungal infections, etc., will also be recorded on patient case report forms (CRF). The blinded CAC will adjudicate all reported secondary endpoints.

4 Subject Selection and Withdrawal

4.1 Inclusion Criteria

Patients who satisfy all of the eligibility criteria outlined below are to be included in the PARITY study:

1) Men and women of skeletal maturity (16 years of age or older);
2) Primary bone malignancies or benign aggressive tumors of the lower extremity;
3) Treatment by excision and endoprosthetic reconstruction;
4) Preoperative chemotherapy (non-compulsory);
5) Provision of informed consent

4.2 Exclusion Criteria

Patients who meet any of the following criteria are not to be included in the PARITY study:

1) Methicillin-resistant Staphylococcus Aureus (MRSA), or Vancomycin Resistant Enterococcus (VRE) colonization*;
2) Allergy to study antibiotics [Ancef® (cefazolin)];
3) Skeletal immaturity**;
4) Upper extremity endoprosthetic reconstruction;
5) Prior surgery in the affected limb (excluding a biopsy);
6) Revision surgery or prior infection in the limb***
7) Enrolled in a competing study

** unable to safely randomize antibiotics in these patients; ** endoprosthetic reconstruction generally not utilized in skeletally immature patients; *** higher risk of infection (vs. baseline) in patients undergoing revision or with prior infection*

4.3 Subject Recruitment and Screening

Each clinical site will have a locally responsible investigator who will oversee the administration of the trial at the local level. The treating physicians at each site will identify potentially eligible patients upon presentation with a lower extremity bone sarcoma. A resident or a delegate will be responsible for obtaining informed consent. All patients who meet the inclusion criteria will be registered and failure to randomize patients will be documented. All patients will be screened for eligibility and documented as: 1) eligible and included, 2) eligible and missed, and 3) excluded. The CAC will adjudicate all situations where eligibility is in doubt. The research coordinator will be responsible for completing the relevant case report forms and screening logs, conducting follow-up visits with each patient, and ensuring completed forms are scanned into the electronic Data Management System (iDataFax). **Figure 1** outlines this process.

Upon receiving their respective Research Ethics Board (REB) approval, participating Orthopaedic Oncologists at each center will be educated on the process of patient enrolment for our study. Access credentials to an internet based randomization website will be provided along with a consent package including a general form for patient demographics, tumor grade and stage, neoadjuvant treatment and proposed adjuvant treatment. Data on the skin prep used, the type and lot of prosthetic, the usage of antibiotic cement, and operative time will also be collected. At the time of procedure consent, patients meeting inclusion criteria will be introduced to the study and consent or refusal obtained. Prior to the surgeon filling out the preoperative orders for antibiotics, the internet based randomization program will be utilized to determine the antibiotic duration. For patients who are allocated to the long-term antibiotic group, discharge will be defined as the date of discharge from the Orthopaedic surgery acute floor to final destinations of home, rehabilitation, or a medical unit for a non-Orthopaedic, non-infection related surgical complication.

4.4 Early Withdrawal of Subjects

4.4.1 When and How to Withdraw Subjects

We will only withdraw patients for the following scenarios:

- If patients withdraw consent for participation or
- If patients are deemed loss to follow-up after all exhaustive measures have been taken to locate the patient.

We will document the reasons for patient withdrawal from the trial. We will not withdraw patients if the study protocol was not adhered (e.g., occurrence of protocol deviations, missed follow-up visits, etc.).

4.4.2 Data Collection and Follow-up for Withdrawn Subjects

To maximize the integrity of the data, all possible attempts will be made to collect as much data as possible and to reduce loss to follow-up (Section 6.9). If a patient wishes to withdraw their consent from the study, the following strategies will be used to reduce the demands of the study and help to retain the subject:

- Ask the patient if you can still collect clinical data from their medical and hospital charts; and
- Ask the patient if you may contact them by telephone to ask about the primary and secondary outcomes.

Patients should not be deemed lost to follow-up until the 12 month visit is due and all attempts to contact the patient have been exhausted.

5 Study Interventions

5.1 Randomization Methods

The patients will be randomized to either short-term duration or long-term duration antibiotics. We will conceal allocation for our study using a centralized 24-hour computerized randomization system. Patients will be the unit of randomization. Randomization will occur in random permuted blocks with varying block sizes of two or four. Based upon our international survey of surgeons and current evidence, randomization will be stratified for the following variables: 1) location of tumor (femur vs. tibia), 2) center and 3) perioperative chemotherapy (yes/no).

5.2 Antibiotic Regimens

Patients will either be randomized to either the short arm antibiotic regimen or the long arm antibiotic regimen. Patients randomized to the short arm regimen will receive 2g of intravenous Ancef® (cefazolin) postoperatively every 8 hours for 24 hours, followed by intravenous saline for an additional 4 days. Conversely, patients randomized to the long arm regimen will receive 2g of intravenous Ancef® (cefazolin) postoperatively every 8 hours over a 5 day period. All patients will also receive 2g of intravenous Ancef® (cefazolin) preoperatively and every 3-4 hours intraoperatively. For prophylaxis, no other antibiotics will be administered.

5.3 Blinding

Patients, surgeons and data analysts will be blinded to the antibiotic regimen. Members of the CAC will also be blinded to the study treatment, as will the nurse(s) administering treatment. The pharmacy technician preparing the solutions will not be blinded however. Patients randomized to short-term antibiotics will receive 4 days of 'sham' antibiotics with saline replacing the Ancef dose.

6 Study Procedures

Completed forms recording patient status should be sent electronically to iDataFax promptly via Electronic Data Capture, once each of the defined follow up visits are completed. Completed forms for patient screening, randomization, and surgical interventions should be as soon as they are completed. It is anticipated that completed forms will be sent within seven days. See **Figure 1** for Study Follow-up Timeline.

6.1 Patient Screening and Consent

Research Coordinators and/or Investigators (or their designees) (as permitted by local regulations) should screen all patients attending weekly sarcoma clinics who are possible candidates for lower extremity wide resection and endoprosthetic reconstruction. The Screening Form should be completed, and patient consent should be obtained using local IRB/REB approved Informed Consent Form to participate the trial.

6.2 Randomization

Patients should be randomized after the patient eligibility is established and the patient consent is obtained. Both study consent and operative consent will be obtained at the pre-operative clinic visit, 1-2 weeks before the anticipated date of surgery. At this time, the Randomization and Baseline Characteristics Forms should be completed. Randomization will then occur, either before or during surgery, prior to case completion. Randomization will be carried out by the pharmacy technician.

6.3 Surgical Treatment

The surgical management of the tumor will take place as is standard for the participating surgeon. Tumor Characteristics Form, Surgical Report Form, Peri-operative Form, and Antibiotics Log should be completed. Only antibiotics that are prescribed for the randomized tumor are to be recorded on the Antibiotics Log. Patients should be assessed for any adverse events and protocol deviations.

6.4 2 Week Follow-up

The 2 week follow-up visit should occur in person either in clinic or at the hospital (if prior to discharge). The 2 Week Follow-up Form should be completed. Additionally, MSTS and TESS Forms should be completed. Patients should be assessed for any AEs, SAEs, infections (surgical site and other), protocol deviations, wound healing and oncologic outcomes such as local recurrence and metastases. The appropriate forms to record these events should be completed as necessary. A Missed Follow up Form should be completed if the patient misses the follow up visit. An Early Withdrawal Form should only be completed if the patient withdraws their consent.

6.5 4 Week Follow-up

The 4 week follow-up visit should occur in person either in clinic or at the hospital (if prior to discharge). The 4 Week Follow-up Form should be completed. Additionally, MSTS and TESS Forms should be completed. Patients should be assessed for any AEs, SAEs, infections (surgical site and other), protocol deviations, wound healing and oncologic outcomes such as local recurrence and metastases. The appropriate forms to record these events should be completed as necessary. A Missed Follow up Form should be completed if the patient misses the follow up visit. An Early Withdrawal Form should only be completed if the patient withdraws their consent.

6.6 3 Month Follow-up

The 3 month follow-up visit should occur in person either in clinic or at the hospital (if prior to discharge). The 3 Month Follow-up Form should be completed. Additionally, MSTS and TESS Forms should be completed. Patients should be assessed for any AEs, SAEs, infections (surgical site and other), protocol deviations, wound healing and oncologic outcomes such as local recurrence and metastases. The appropriate forms to record these events should be completed as necessary. A Missed Follow up Form should be completed if the patient misses the follow up visit. An Early Withdrawal Form should only be completed if the patient withdraws their consent.

6.7 6 Month Follow-up

The 6 month follow-up visit should occur in person either in clinic or at the hospital (if prior to discharge). The 6 Month Follow-up Form should be completed. Additionally, MSTS and TESS Forms should be completed. Patients should be assessed for any AEs, SAEs, infections (surgical site and other), protocol deviations, wound healing and oncologic outcomes such as local recurrence and metastases. The appropriate forms to record these events should be completed as necessary. A Missed Follow up Form should be completed if the patient misses the follow up visit. An Early Withdrawal Form should only be completed if the patient withdraws their consent.

6.8 9 Month Follow-up

The 6 month follow-up visit should occur in person either in clinic or at the hospital (if prior to discharge). The 6 Month Follow-up Form should be completed. Additionally, MSTS and TESS Forms should be completed. Patients should be assessed for any AEs, SAEs, infections (surgical site and other), protocol deviations, wound healing and oncologic outcomes such as local recurrence and metastases. The appropriate forms to record these events should be completed as necessary. A Missed Follow up Form should be completed if the patient misses the follow up visit. An Early Withdrawal Form should only be completed if the patient withdraws their consent.

6.9 1 Year Follow-up

The 1 year follow-up visit should occur in person either in clinic or at the hospital (if prior to discharge). The 1 Year Follow-up Form should be completed. Additionally, MSTS and TESS forms should be completed. Patients should be assessed for any AEs, SAEs, infections (surgical site and other), protocol deviations, wound healing and oncologic outcomes such as local recurrence and metastases. The appropriate forms to record these events should be completed as necessary. A Missed Follow up Form should be completed if the patient misses the follow up visit. An Early Withdrawal Form should only be completed if the patient withdraws their consent.

6.10 Maximization of Follow-up

It is extremely important to maintain patients follow up in the trial to ensure the completeness and integrity of the data. We will implement several procedures to limit loss of follow up, as described in **Table 2** below.[23]

Table 2: Strategies to Limit Loss to Follow-Up

1) Individuals should be excluded if they are likely to present problems with follow-up (refer to exclusion criteria).
2) At the time of randomization, as well as their own address and telephone number, each patient should

provide the name and address of their primary care physician, and the name, address and phone number of three people at different addresses with whom the patient does not live with who are likely to be aware of the patient's whereabouts. The research coordinator should confirm that these numbers are accurate prior to the patient's discharge from hospital.
3) Whenever possible, participants should be given information on open extremity fractures, their complications and the potential treatment effects, expectations for personal benefit from study participation, and be encouraged for adherence with follow-up visits and research protocols.
4) The Study Coordinator should remind patients of upcoming clinic visits.
5) Study coordinator should contact patients no less than once every three months to maintain contact and obtain information about any planned change in residence.
6) If a patient refuses to return for a follow-up assessment, study surgeons and coordinator should determine his/her status with regard to revision surgery or any secondary outcome by phone contact with the patient or the patient's family physician.

6.11 Minimization of Crossovers of Surgical Interventions

Crossovers are extremely unlikely between the short- and long-duration antibiotic regimens as patients will be blinded and acute infections are unlikely to occur in the first 5 days after surgery**.** Any patients who do crossover will be analyzed in the group to which they were originally allocated, maintaining the 'intention to treat' approach we plan for the analysis. Our standardization of management protocols will limit co-intervention, and we will document the use of drugs that affect antibiotic metabolism, and major additional procedures that patients undergo while in the hospital or other site infections (urinary tract, Port or PICC line). Research coordinators will record all medications and therapy used concurrently in included patients on the CRFs.

6.12 Adjudication Requirements

The CAC will adjudicate the following:

- All situations where eligibility is in doubt;
- Review reports of infection;
- Determine if implant is stable radiographically
- Decide if infection (meeting study criteria) has occurred

The CAC will be blinded to allocation. A web-based (password protected) adjudication process will occur using the Global Adjudicator™ platform, outlined in **Figure 2**.

Figure 2: Adjudication Process for Central Outcomes Adjudication Committee (CAC)

1. The Study Coordinator or Research Assistant at the Methods Center will retrieve from each site:
    - X-rays (initial, post-operative and just prior to the revision surgery)
    - Relevant chart notes (operative report, chart notes leading up to revision surgery, and any other relevant laboratory data such as wound cultures).

↓

2. All information will be edited to remove any patient identifiers by the Study Coordinator or Research Assistant at Methods Center. Chart notes will be re-typed for clarity.

↓

3. X-rays will be uploaded to the website as JPeg files and chart notes will be posted as PDF files. Each patient will have a unique identification number on the website. Additional DataFax forms will also be converted to PDF files and posted for each patient.

↓

4. Each CAC member will independently adjudicate all posted cases. They will log all answers on specific data forms developed, tested and utilized for the study.

↓

5. After all CAC members have completed the web-based adjudication, a consensus table with each member's results will be prepared by the research coordinator at the methods center.

↓

6. Following each adjudication batch posting, CAC members will communicate via conference call (or in person if coinciding with a scheduled meeting) and discuss all disagreements. The chair of the CAC will run each session.

↓

7. A consensus will be achieved for all cases. Occasionally, the CAC may request additional information from a site to achieve consensus. In situations where a consensus cannot be achieved, a majority vote will be taken.

7 Statistical Plan

7.1 Sample Size Determination

Our choice of sample size is based upon pairwise comparisons for the primary outcome (deep infection) of long- vs. short-term antibiotics. We hypothesize that short-term antibiotics will have similar or lower rates of infection (primary outcome) and less

antibiotic related complications (secondary outcome) at 12 months compared with long-term antibiotics. We have chosen alpha levels of 0.05 for the primary and 0.01 for the secondary outcomes. We will evaluate 3 secondary outcomes, but because they are likely to be correlated, the Bonferroni correction would be excessively conservative.

For the primary outcome, we will power the study for a non-inferiority design (i.e. short-term antibiotics is similar or better than long-term antibiotics with respect to deep infection rates at 12 months). The logic of the non-inferiority trial is that we anticipate that short-term antibiotics will be superior in terms reduced antibiotic-related complications, and that as a result it would be the regimen of choice unless it proves inferior in terms of infection. Estimates for infection rates with endoprosthetic reconstruction have ranged from 0-22% with a weighted mean of 9% (95% confidence interval: 7.4% to 10.7%). We have set an upper threshold (i.e. margin of non-inferiority) of an absolute difference of 5% to define non-inferiority: up to a 5% higher infection rate with short-term antibiotics will be considered non-inferior to long-term antibiotics. This upper threshold was determined by the PARITY Survey responses indicating that infection rates within 5% of each other would not be considered different.[10]

Our power table suggests acceptable study power for our non-inferiority design can be achieved with 457 patients per study arm (total: 914 patients), assuming a 10% baseline risk of infection, a 5% non-inferiority margin, an alpha=0.05, and an assumed study power of 80% (Beta=0.20). (**Table 3)** Adjustments for potential loss to follow up, errors in eligibility, and study drop outs, would require an estimated sample of 1042 patients (521 patients per arm). Thus, a study of 100 patients, representing approximately 10% of the anticipated definitive study sample size, represents a sufficient number to adequately determine study feasibility and compliance with study procedures.

Table 3: Sample Sizes Per Group for 80% power, α=0.05.

	**Control Infection Rate**			
**Absolute acceptable difference**	**10%**	**13%**	**15%**	**20%**
**2%**	2795	3505	3948	4949
**3%**	1249	1562	1758	2201
**4%**	708	882	992	1240
**5%**	**457**	567	637	794
**6%**	321	396	444	553
**7%**	239	293	328	407

**10%**	122	147	163	201

7.2 Statistical Methods

The success of our study will relate directly to our objectives and measures of outcome. We will consider our study a success if the following criteria are met: 1) 100 patients recruited by 12 months, 2) 95 of 100 patients (95%) achieving follow up at 1 year for infections rates, 3) 90 of 100 patients (90%) achieving follow up for secondary outcomes (complications and functional scores), 4) At least 90% case report form completeness with no outstanding queries at 1 year, 5) 4 or fewer errors in randomization across the 100 enrolled patients, 6) At least 90 of 100 patients (90%) adherence to the protocol, and 7) At least 70 of 100 patients (70%) compliance in each of the following: perioperative management, adherence to follow up schedule, and avoidance of study crossovers.

The primary analysis will follow the intention-to-treat principle and compare the proportions of infection between the postoperative antibiotic duration arms (24 hours vs. 5 days duration), using Fisher's Exact Tests. We will quantify the treatment effect with an absolute difference in rate of infection, with the associated 95% confidence interval and p-value. We will also conduct a multiple regression model to determine if total operative time, tumor location, chemotherapy regimen, diabetes, smoking or other factors are related to infection rates. We plan to conduct subgroup analyses for infection rates within each type of tumor (Ewing's, Osteosarcoma, Chondrosarcoma, Giant Cell Tumor) and tumor location (proximal femur, distal femur and proximal tibia). However, due to inadequate sample size and power to conduct this analysis, the results will be used solely for generating hypotheses for future investigations.[24]

8 Safety and Adverse Events

8.1 Definitions

8.1.1 Adverse Event

An ***adverse event*** (AE) is any symptom, sign, illness or experience that develops or worsens in severity during the course of the study

8.1.2 Serious Adverse Event

Adverse events are classified as serious or non-serious. A ***serious adverse event*** is any AE that is:

- fatal,

- life-threatening,
- requires or prolongs hospital stay,
- results in persistent or significant disability or incapacity,
- a congenital anomaly or birth defect, or
- an important medical event.

8.1.3 Unanticipated Problems Resulting in Risk to Subjects or Others

Any incident, experience, or outcome that meets all of the following criteria:

- unexpected in nature, severity, or frequency (i.e. not described in study-related documents such as the IRB-approved protocol or consent form, the investigators brochure, etc),
- related or possibly related to participation in the research (i.e. possibly related means there is a reasonable possibility that the incident experience, or outcome may have been caused by the procedures involved in the research),
- suggests that the research places subjects or others at greater risk of harm (including physical, psychological, economic, or social harm).

Unanticipated problems resulting in risk to volunteers or others encompass more than what one usually thinks of as adverse events. “Problems involving risk” may not necessarily result in harm. For example, misplacing a volunteer’s study records containing identifiable private information introduces the risk of breach of confidentiality. Confidentiality may or may not be breached, but either way this would be a reportable event. Risks to others must also be reported. For example, an unexpected outburst during questionnaire administration by a volunteer that puts study staff at risk would be a reportable event.

8.2 Reporting of Serious Adverse Events and Unanticipated Problems Resulting in Risk to Subjects or Others

All serious adverse events and unanticipated problems resulting in risk to subjects or others are to be reported to the Methods Center immediately.

8.2.2 Investigator Reporting: Notifying the Methods Center

Any SAEs must be reported to the Methods Center by completing the SAE Form and submitting it to iDataFax. The investigator will keep a copy of this SAE form on file at the study site. The SAE form should include of a written narrative and any other information that will assist the understanding of the event. Significant new information on ongoing serious adverse events should be provided promptly to the Methods Center by updating the SAE form.

Unanticipated problems resulting in risk to subjects or others are to be reported to the Methods Center by either fax or email.

8.2.3 Site Investigator – IRB/REB Reporting

Investigators are responsible for reporting AEs, SAEs, and unanticipated problems resulting in risk to subjects or others to their local IRB/REB. Investigators are responsible for complying with their local IRB's/REB's reporting requirements. Copies of each report and documentation of IRB/REB notification and receipt will be kept in the investigator's study file.

8.2.4 Data Monitoring Committee (DMC)

The DMC will be established at the onset of the trial to monitor the trial and review the study bi-annual progress report.[25, 26] The Committee members will be independent of the trial, free of conflicts with any of the investigative team and will consist of a clinical trial methodologist, a statistician and Orthopaedic Surgeons. The terms of reference and functions are derived from the principles established by the Data and Safety Monitoring Boards: Lessons, Ethics, Statistics (DAMOCLES) Study Group charter.

9 Data Handling and Record Keeping

9.1 Confidentiality

Information about study subjects will be kept confidential and managed according to the requirements of the Health Insurance Portability and Accountability Act of 1996 (HIPAA). Those regulations require a signed subject authorization informing the subject of the following:

- What protected health information (PHI) will be collected from subjects in this study,
- Who will have access to that information and why,
- Who will use or disclose that information, and
- The rights of a research subject to revoke their authorization for use of their PHI.

In the event that a subject revokes authorization to collect or use PHI, the investigator, by regulation, retains the ability to use all information collected prior to the revocation of subject authorization. For subjects that have revoked authorization to collect or use PHI, attempts should be made to obtain permission to collect at least vital status (i.e. that the subject is alive) at the end of their scheduled study period.

9.2 Case Report Forms

The CRFs are the primary data collection instrument for the study. All data requested on the CRF must be recorded. All missing data must be explained. If a space on the CRF is left blank because the procedure was not done or the question was not asked, write "N/D". If the item is not applicable to the individual case, write "N/A". Sites will

receive an iDataFax manual which includes detailed instructions for entering data using iDataFax.

10 Ethical Considerations

This study is to be conducted according to US and international standards of Good Clinical Practice (FDA Title 21 part 312 and International Conference on Harmonization guidelines), applicable government regulations, and Institutional research policies and procedures.

This protocol and any amendments will be submitted to a properly constituted independent REB or IRB, in agreement with local legal prescriptions, for formal approval of the study conduct. The decision of the REB /IRB concerning the conduct of the study will be made in writing to the investigator and a copy of this decision will be provided to the Methods Center before commencement of this study.

All subjects for this study will be provided a consent form describing this study and providing sufficient information for subjects to make an informed decision about their participation in this study. The consent form will be submitted with the protocol for review and approval by the REB /IRB for the study. The formal consent of a subject, using the REB /IRB-approved consent form, must be obtained before that subject undergoes any study procedure. The consent form must be signed by the subject or legally authorized representative, and the investigator-designated research professional obtaining the consent.

11 References

1. Jeys L, Grimer R The long-term risks of infection and amputation with limb salvage surgery using endoprostheses. *Recent Results Cancer Res* 2009;179:75-84.:75-84.

2. Morii T, Yabe H, Morioka H, Beppu Y, Chuman H, Kawai A, Takeda K, Kikuta K, Hosaka S, Yazawa Y, Takeuchi K, Anazawa U, Mochizuki K, Satomi K Postoperative deep infection in tumor endoprosthesis reconstruction around the knee. *J Orthop Sci* 2010;15:331-9.

3. Grimer RJ, Belthur M, Chandrasekar C, Carter SR, Tillman RM Two-stage revision for infected endoprostheses used in tumor surgery. *Clin Orthop Relat Res* 2002:193-203.

4. Jeys LM, Grimer RJ, Carter SR, Tillman RM Risk of amputation following limb salvage surgery with endoprosthetic replacement, in a consecutive series of 1261 patients. *Int Orthop* 2003;27:160-3.

5. Wirganowicz PZ, Eckardt JJ, Dorey FJ, Eilber FR, Kabo JM Etiology and results of tumor endoprosthesis revision surgery in 64 patients. *Clin Orthop Relat Res* 1999:64-74.

6. Akahane T, Shimizu T, Isobe K, Yoshimura Y, Fujioka F, Kato H Evaluation of postoperative general quality of life for patients with osteosarcoma around the knee joint. *J Pediatr Orthop B* 2007;16:269-72.

7. Abudu A, Carter SR, Grimer RJ The outcome and functional results of diaphyseal endoprostheses after tumour excision. *J Bone Joint Surg* 1996; 78B:652-657.

8. Abudu A, Grimer RJ, Tillman RM, Carter SR Endoprosthetic replacement of the distal tibia and ankle joint for aggressive bone tumours. *Int Orthop* 1999;23:291-294.

9. Grimer RJ, Carter SR, Tillman RM, Sneath RS, Walker PS, Unwin PS, Shewell PC Endoprosthetic replacement of the proximal tibia. *J Bone Jointt Surg* 1999;81B:488-494.

10. Hasan K, Racano A, Deheshi B, Farrokhyar F, Wunder J, Ferguson P, Holt G, Schwartz H, Petrisor B, Bhandari M, Ghert M Prophylactic antibiotic regimens in tumor surgery (PARITY) survey. 2011. *Manuscript submitted for publication*.

11. Bratzler DW, Houck PM Antimicrobial prophylaxis for surgery: an advisory statement from the National Surgical Infection Prevention Project. *Clin Infect Dis* 2004;38:1706-15.

12. Zhanel GG, Adam HJ, Low DE, Blondeau J, DeCorby M, Karlowsky JA, Weshnoweski B, Vashisht R, Wierzbowski A, Hoban DJ Antimicrobial susceptibility of 15,644 pathogens from Canadian hospitals: results of the CANWARD 2007-2009 study. *Diagn Microbiol Infect Dis* 2011;69:291-306.

13. Costelloe C, Metcalfe C, Lovering A, Mant D, Hay AD Effect of antibiotic prescribing in primary care on antimicrobial resistance in individual patients: systematic review and meta-analysis. *BMJ* 2010;340:c2096. doi: 10.1136/bmj.c2096.:c2096.

14. Bergogne-Berezin E Treatment and prevention of antibiotic associated diarrhea. *Int J Antimicrob Agents* 2000;16:521-6.

15. Weiss K, Bergeron L, Bernatchez H, Goyette M, Savoie M, Thirion D Clostridium difficile-associated diarrhoea rates and global antibiotic consumption in five Quebec institutions from 2001 to 2004. *Int J Antimicrob Agents* 2007;30:309-14.

16. Shigemura K, Tanaka K, Adachi M, Yamashita M, Arakawa S, Fujisawa M Chronological change of antibiotic use and antibiotic resistance in Escherichia coli causing urinary tract infections. *J Infect Chemother* 2011.

17. Hueston WJ, Dickerson L Antibiotic resistance and the need for the rational use of antibiotics. *J Med Liban* 2001;49:246-56.

18. Christiaens TC, Digranes A, Baerheim A The relation between sale of antimicrobial drugs and antibiotic resistance in uropathogens in general practice. *Scand J Prim Health Care* 2002;20:45-9.

19. Horan TC, Gaynes RP, Martone WJ, Jarvis WR, Emori TG CDC definitions of nosocomial surgical site infections, 1992: a modification of CDC definitions of surgical wound infections. *Am J Infect Control* 1992;20:271-274.

20. Davis AM, Wright JG, Williams JI, Bombardier C, Griffin A, Bell RS Development of a measure of physical function for patients with bone and soft tissue sarcoma. *Qual Life Res* 1996;5:508-16.

21. Schreiber D, Bell RS, Wunder JS, O'Sullivan B, Turcotte R, Masri BA, Davis AM Evaluating function and health related quality of life in patients treated for extremity soft tissue sarcoma. *Qual Life Res* 2006;15:1439-46.

22. Tunn PU, Pomraenke D, Goerling U, Hohenberger P Functional outcome after endoprosthetic limb-salvage therapy of primary bone tumours--a comparative analysis using the MSTS score, the TESS and the RNL index. *Int Orthop* 2008;32:619-25.

23. Sprague S, Leece P, Bhandari M, Tornetta P, Schemitsch E, Swiontkowski M. Limiting loss to follow-up in a multicenter randomized trial in orthopaedic surgery. *Control Clin Trials* 2003; 24: 719-725.

24. Assmann SF, Pocock SJ, Enos LE, Kasten LE Subgroup analysis and other (mis)uses of baseline data in clinical trials. *Lancet* 2000;355:1064-9.

25. Friedman L The NHLBI model: a 25 year history. *Stat Med* 1993;12:425-31.

26. Slutsky AS, Lavery JV Data safety and monitoring boards. *N Engl J Med* 2004;350:1143-7.

Protocol Version 6.0 (Cefazolin)

(Follows)

Prophylactic Antibiotic Regimens in Tumor Surgery (PARITY): A Multi-Center Randomized Controlled Study Comparing Alternative Antibiotic Regimens in Patients Undergoing Tumor Resections with Endoprosthetic Replacements

Methods Center: Center for Evidence-Based Orthopaedics (CEO)
McMaster University
293 Wellington Street North
Suite 110
Hamilton, Ontario L8L 8E7

Funding Sponsors: Orthopaedic Research & Education Foundation / Musculoskeletal Tumor Society (OREF/MSTS)
Physicians' Services Incorporated (PSI)
Canadian Cancer Society Research Institute (CCSRI)
Canadian Institutes of Health Research (CIHR)

TABLE OF CONTENTS

List of Abbreviations iv

Study Summary 1

1 Introduction 2

1.1 Background 2

1.2 Preclinical Data 2

1.2.1 Best Evidence for Infection Rates 2

1.2.2 Lack of Consensus in Antibiotic Regimens and Global Interest in a Randomized Trial 4

1.2.3 Complications of Antibiotic Overuse 4

2 Study Objectives 6

2.1 Primary Questions 6

2.2 Secondary Questions 6

3 Study Design 6

3.1 Published Survey Results Show 8

3.2 Primary Study Endpoints 8

3.3 Secondary Study Endpoints 9

4 Subject Selection and Withdrawal 9

4.1 Inclusion Criteria 9

4.2 Exclusion Criteria 9

4.3 Subject Recruitment and Screening 10

4.4 Early Withdrawal of Subjects 11

4.4.1 When and How to Withdraw Subjects 11

4.4.2 Data Collection and Follow-up for Withdrawn Subjects 11

5 Study Interventions 11

5.1 Allocation for the Study 11

5.2 Antibiotic Regimens 12

5.3 Blinding 12

5.3.1 Unblinding Procedure 13

6 Study Procedures 13

6.1 Patient Screening and Consent 13
6.2 Randomization 14
6.3 Surgical Treatment 14
6.4 2-Week Follow-Up 14
6.5 6-Week Follow-Up 15
6.6 3-Month Follow-Up 15
6.7 6-Month Follow-Up 15
6.8 9-Month Follow-Up 15
6.9 1-Year Follow-Up 16
6.10 Maximization of Follow-up 16
6.11 Minimization of Crossovers of Surgical Interventions 17
6.12 Adjudication Requirements 17

7 Statistical Plan 19
7.1 Sample Size Determination 19
7.2 Statistical Methods 19

8 Safety and Adverse Events 22
8.1 Definitions 22
8.1.1 Adverse Event 22
8.1.2 Serious Adverse Event 22
8.1.3 Unanticipated Problems Resulting in Risk to Subjects or Others 22
8.2 Reporting of Adverse Events, Serious Adverse Events, and Unanticipated Problems Resulting in Risk to Subjects or Others 23
8.2.1 Investigator Reporting: Notifying the Methods Center 23
8.2.2 Site Investigator – IRB/REB Reporting 23
8.2.3 Data Safety Monitoring Board (DSMB) 24

9 Data Handling and Record Keeping 24
9.1 Confidentiality 24
9.2 Case Report Forms 24

10 Ethical Considerations 24

11 References 26

List of Abbreviations

Abbreviations are listed in alphabetical order

AE: Adverse Event
CAC: Central Adjudication Committee
CDC: Centers for Disease Control and Prevention
CRF: Case Report Form
DSMB: Data Safety Monitoring Board
FDA: Food and Drug Administration
GCP: Good Clinical Practice
HIPAA: Health Insurance Portability and Accountability Act
IRB: Institutional Review Board
MSTS: Musculoskeletal Tumor Society Questionnaire
PHI: Protected Health Information
PI: Principal Investigator
RCT: Randomized Controlled Trial
REB: Research Ethics Board
SAE: Serious Adverse Event
SSI: Surgical Site Infection
TESS: Toronto Extremity Salvage Score

Study Summary

Title	Prophylactic Antibiotic Regimens in Tumor Surgery (PARITY): A Multi-Center Randomized Controlled Study Comparing Alternative Antibiotics Regimens in Patients Undergoing Tumor Resections with Endoprosthetic Replacements
Short Title	PARITY
Methodology	Multi-Center, Blinded, Randomized Trial
Study Duration	December 2012 to March 2021
Study Center(s)	Multi-Center
Primary Study Question	In patients undergoing surgical excision and endoprosthetic reconstruction of the femur or tibia for a tumor, is a long-term (5 days) post-operative antibiotic regimen more effective at decreasing the rate of infection when compared to a short-term (24 hours) post-operative antibiotic regimen?
Number of Subjects	600
Diagnosis and Main Inclusion Criteria	Primary malignant or benign aggressive bone tumors of the femur or tibia, soft-tissue sarcomas of the lower extremity which have invaded the femur or tibia, or oligometastatic bone disease of the femur or tibia that requires surgical excision and endoprosthetic reconstruction
Study Product, Dose, Route, Regimen	Antibiotic regimens: intravenous cefazolin for 24 hours and 5 days

1 Introduction

This document is a protocol for a multi-center, blinded, randomized controlled trial, using a parallel two-arm design, to investigate whether a long-term (5 days) post-operative antibiotic regimen will decrease the rate of infection among patients being surgically treated for a tumor in the femur or tibia when compared to a short-term (24 hours) post-operative antibiotic regimen. The rationale for this study is fuelled by: 1) increased infection rate outcomes in bone tumor surgery compared to general arthroplasty; 2) a lack of consensus among Orthopaedic Oncologists regarding the most effective prophylactic antibiotic regimen; 3) a lack of randomized controlled trial (RCT) evidence; and 4) extensive investigator support for the proposed trial.

1.1 Background

Limb salvage surgery is the standard of care in the management of sarcoma of the long-bones. Advances in chemotherapeutic regimens and imaging techniques allow for wide resection and functional reconstruction in the 95% of patients. The most common type of long-bone reconstruction involves the use of a tumor prosthesis, or endoprostheses. Due to the complexity and length of surgical resection and reconstruction, as well as the immunocompromised nature of patients treated with chemotherapy, the risk for infection remains high.[1, 2] Infection following endoprosthetic reconstruction is a devastating complication that requires staged revision surgery and long-term intravenous antibiotics.[3] The risk for recurrent infection remains high, as does the risk for ultimate amputation.[1, 4, 5] However, the most effective antibiotic regimen in preventing post-operative infections remains controversial, and the current state of practice varies widely, particularly with respect to antibiotic duration. Moreover, patients' quality of life and function following infection are dramatically impacted, as are health care costs.[6] Strategies to optimize prevention of infection and quality of life, while mitigating health care costs are needed.

1.2 Preclinical Data

1.2.1 Best Evidence for Infection Rates

A systematic review was performed comparing the infection rate outcomes reported following the surgical treatment of primary long-bone tumors (malignant and benign aggressive) by excision and endoprosthetic reconstruction. The literature search was conducted of the Medline, EMBASE, and all EBM Reviews (including Cochrane) databases, as well as the proceedings for past American Society for Clinical Oncology (ASCO) Annual Meetings. The initial search generated 3898 titles. Of the 3898 titles, 48 eligible papers were identified and are listed below in **Table 1**. The deep infection rates ranged from 0% to 25.0% with a weighted mean of 9.5% (95% confidence interval:

8.1% to 11.0%). Those papers that reported antibiotic regimens varied significantly from ‘intra-operative dosing only’ to ‘greater than 72 hours’.[7-9]

Table 1: Deep Infection Rates Reported by Systematic Review

Study	Year	Number	Deep infection rate
Lee et al.	1990	17	0.0%
Roberts et al.	1991	133	5.3%
Horowitz et al.	1991	12	25.0%
Eckardt et al.	1991	68	1.5%
Shih et al.	1993	61	6.6%
Morris et al.	1995	31	3.2%
Malawer et al.	1995	51	19.6%
Zehr et al.	1996	17	5.9%
Abudu et al.	1996	16	0.0%
Abudu et al.	1999	5	20.0%
Lee et al.	1999	6	16.7%
Grimer et al.	1999	151	18.5%
Kawai et al.	1999	32	6.3%
Kabukcuoglu et al.	1999	54	1.9%
Natarajan et al.	2000	6	16.7%
Ilyas et al.	2000	15	13.3%
Ilyas et al.	2001	48	8.3%
Donati et al.	2001	25	4.2%
Wunder et al.	2001	64	6.3%
Sokolov	2002	38	10.5%
Ilyas et al.	2002	15	6.7%
Bickels et al.	2002	110	5.5%
Anract et al.	2002	9	22.2%
Griffin et al.	2005	99	10.1%
Natarajan et al.	2005	246	6.9%
Jeys et al.	2005	1036	11.9%
Sharma et al.	2006	77	7.8%
Farid et al.	2006	52	3.8%
Orlic et al.	2006	82	4.9%
Gosheger et al.	2006	250	12.0%
Sharma et al.	2007	112	9.8%
Myers et al.	2007	194	19.6%
Sim et al.	2007	50	12.00%
Finstein et al.	2007	62	4.80%
Myers et al.	2007	335	9.6%
Akahane et al.	2007	11	9.1%
Gitelis et al.	2008	80	2.5%

**Guo et al.**	2008	104	6.7%
**Jeys et al.**	2008	530	12.8%
**Sewell et al.**	2009	22	0.0%
**Natarajan et al.**	2009	17	11.8%
**Shekkeris et al.**	2009	6	16.7%
**Chandrasekar et al.**	2009	100	2.0%
**Lee et al.**	2009	256	9.8%
**Morii et al.**	2010	82	12.2%
**Hanna et al.**	2010	23	5.6%
**Hardes et al.**	2010	125	12.80%
**Li et al.**	2011	49	2.0%
**Sewell et al.**	2011	14	7.1%

1.2.2 Lack of Consensus in Antibiotic Regimens and Global Interest in a Randomized Trial

A survey was published addressing the practices of Orthopaedic Oncologists registered with the Musculoskeletal Tumor Society (MSTS) and the Canadian Orthopaedic Oncology Association (CANOOS). From this survey, it was concluded that there is currently a lack of guidelines for the prescription of prophylactic antibiotics in Musculoskeletal Tumor Surgery, which has left Orthopaedic Oncologists with varying opinions and practices.[10] Of the 97 surgeons who received the questionnaire, 72 responded (75% response rate (95% CI: 65.5, 82.5%)). While almost all respondents agreed antibiotic regimens were important in reducing the risk of infection, respondents varied considerably in their choices of antibiotic regimens and dosages. Although 73% (95% CI: 61, 82%) of respondents prescribe a first generation cephalosporin, one in four favours additional coverage with an aminoglycoside and/or Vancomycin. One in three surgeons (95% CI: 25, 48%) believes antibiotics should be discontinued after 24 hours (as recommended by the AAOS for total joint arthroplasty[11]) but 40% (95% CI: 30, 53%) continue antibiotics until the suction drain is removed.

Given the ongoing uncertainty in evidence to guide best practices, 90% (95% CI: 81, 95%) of respondents agreed that they would change their practice if a large randomized controlled trial showed clear benefit of an antibiotic drug regimen different from what they are currently using. Further support for a clinical trial was observed by an overwhelming surgeon interest (87%; 95% CI: 77, 93%) in participating in a multi-center randomized controlled study.

1.2.3 Complications of Antibiotic Overuse

Antibiotic resistance is an increasingly clinically relevant issue both in surgical and infectious disease literature. The Canadian Antibiotic Resistance Alliance (CARA) publishes statistics intended for use by infectious disease physicians and other medical

and surgical specialists.[12] Our systematic review shows that the most common infective pathogen was *staph aureus*. The 2009 Canadian antibiotogram shows that 100% of MSSA (*methicillin sensitive staphylococcus aureus*) is susceptible to cefazolin (Ancef).[12] However, the prevalence of MRSA, versus MSSA, varies by institution and patient population. Zhanel et al. shows that MRSA comprised 27.0% of all *S. aureus* isolates (68.8% were health care associated [HA-MRSA] and 27.6% were community associated [CA-MRSA]).[12] One hundred percent of both community-associated and health care- associated MRSA showed susceptibility to vancomycin and varying susceptibilities to other antimicrobials. Furthermore, prevalence of antibiotic resistance is increasing in Canada. Data from the Canadian Nosocomial Infection Surveillance Program show that the incidence of MRSA as a proportion of all *S. Aureus* has increased from 1% in 1995 to 8% by 2000 and 27% in 2008 as mentioned above.[12]

The vast majority of prosthetic infections are due to gram positive bacteria, and cefazolin also exhibits gram negative coverage. Notably, the CANWARD 2009 antibiotogram shows 37.6% of *E. coli* and 47.6% of *Klebsiella pneumonia* are susceptible to cefazolin.[12] Based on an expert panel of six Orthopaedic Oncologists and three Infectious Disease specialists who were consulted in preparation for this study, it was determined that the ideal study would be a superiority trial to assess whether 2g of cefazolin given intravenously every 8 hours for 5 days or until discharge from acute care (i.e., long-duration) is more effective than that given intravenously every 8 hours for 24 hours (i.e., short-duration). Despite the fact that 11% of respondents in the PARITY Survey responded that they prescribe an aminoglycoside, the Infectious Disease experts on our panel agreed that this type of gram negative coverage does not add more gram negative coverage to that already provided by cefazolin. In addition, our PARITY survey indicated that there is significant concern in the community regarding nephrotoxicity and ototoxicity associated with aminoglycosides.[10]

Antibiotic misuse and overuse in terms of spectrum and duration respectively are considered the main factors in development of antibiotic resistance.[13] When threatened, bacteria evolve to survive, the main mechanisms being genetic mutation, expression of latent resistance genes, or acquisition of genes with resistance determinants.[13] If the antibiotic resistance profile outruns the development of new antibiotics, we are left defenseless against prosthetic infections, which will significantly impact our ability to salvage infected tumor prosthesis and therefore adversely affect patient morbidity and mortality. In addition to the medium to long-term effects of development of antibiotic resistance, a long course of antibiotics itself is not benign. Complications can vary from an inconvenience to a fatality. Possible complications include the development of *clostridium difficile* diarrhea and toxic megacolon, opportunistic fungal infections, catheter related infections, and seizures.[14-18]

2 Study Objectives

The objective of this study is to determine whether long-term (5 days) post-operative antibiotics will decrease the rate of infection following lower extremity tumor surgery, when compared to short-term (24 hours) post-operative antibiotics. This objective will be carried out by answering the following questions:

2.1 Primary Questions

In patients undergoing surgical excision and endoprosthetic reconstruction of a tumor in the femur or tibia, do long-term (5 days) prophylactic antibiotics lead to decreased rates of post-operative surgical site infections over 12 months?

2.2 Secondary Questions

In patients surgically treated for tumors in the femur or tibia followed by limb reconstruction using an endoprosthesis, what is the impact of the post-operative antibiotic regimen (24 hours vs. 5 days) on: the development of antibiotic-related complications (i.e., gastrointestinal infections, fungal infections, etc.), patient functional outcomes and quality of life, the rate of re-operations, oncologic recurrence and/or metastases, and mortality after one year?

3 Study Design

This study is a multi-center, blinded, randomized controlled trial, using a parallel two-arm design to investigate whether long-term post-operative antibiotic regimens (5 days) will decrease the rate of infection among patients being surgically treated for a tumor in the femur or tibia, when compared to short-term post-operative antibiotics (24 hours).

Patients will be randomized using a 24-hour computerized randomization system that allows random variable block sizes to one of two treatment arms (24 hours or 5 days). The randomization is stratified by: 1) center and 2) location of tumor (femur vs. tibia). The patients will be followed for 1 year after surgery. We will assess infection rates within 12 months after initial surgery across both study arms. Patients, outcome adjudicators and data analysts will be blinded. We will measure function and quality of life pre-operatively, and at 3 months, 6 months, and 1 year post-operatively. The schematic procedure is shown in **Figure 1**.

Figure 1: Trial Conduct Procedure

Patient Recruitment, Randomization and Surgical Interventions

Identification of Patients	Direct referral-within center	Data Collected
	↓	
Assessment of Patient Eligibility	Study explanation<br>History-review eligibility criteria, and other relevant medical conditions<br>Physical Examination<br>Radiographs	Screening Form
	Informed Consent, if eligible	Informed Consent<br>MSTS-87, MSTS-93, TESS (baseline)
	↓ All eligible patients who consent to the trial	
Randomization	24 hour web-based or telephone<br>Eligibility criteria reviewed again<br>Key patient information recorded<br>Randomization issued to patient	Baseline Form<br>Randomization Form
	↓	
Surgery	Either short- or long-arm<br>Surgical protocols will be followed	Surgical Form
	↓	
Follow Up Schedule		
2 Weeks	Assessment of outcome events	Follow-Up Form
6 weeks	Assessment of outcome events	Follow-Up Form
3 Months	Assessment of outcome events	Follow-Up Form<br>MSTS-87, MSTS-93, TESS
6 Months	Assessment of outcome events	Follow-Up Form<br>MSTS-87, MSTS-93, TESS
9 Months	Assessment of outcome events	Follow-Up Form
1 Year	Assessment of outcome events	Follow-Up Form<br>MSTS-87, MSTS-93, TESS

**Follow-Up Forms include AEs, SAEs, infections, reoperations, protocol deviations or wound healing problems, and other appropriate forms*.

3.1 Published Survey Results Show

Infection following endoprosthetic limb reconstruction for sarcoma of the long-bones is a devastating complication. A conducted survey and systematic review show that there are no current best practice guidelines for antibiotic prophylaxis in tumor surgery and that Orthopaedic Oncologists would be interested in enrolling patients in research to inform the development of such guidelines.[10] These findings provide a strong rationale for undertaking a randomized control trial to determine the effects of post-operative antibiotic regimens on infection rate outcomes following bone tumor surgeries of the lower extremities. Implications of this trial may include both fewer endoprosthetic infections as well as fewer antibiotic related complications.

3.2 Primary Study Endpoints

The primary study endpoint is the development of a surgical site infection (SSI) within 12 months following the initial surgery to treat a tumor of the femur or tibia. Patients will be monitored regularly by the treating physician at 2 weeks, 6 weeks, 3 months, 6 months, 9 months, and 1 year.

Surgical site infections will be classified according to the criteria established by the Center for Disease Control (CDC).[19] The CDC defines a SSI as infection occurring within the 30 days following the operative procedure or within 1 year if an implant is in place and the infection appears to be related to the operative procedure. Infection can involve any part of the body, but excludes the skin incision, fascia, or muscle layers, that is opened or manipulated during the operative procedure. The patient must also present with at least one of the following:

- purulent drainage from the superficial/deep/organ space incision;
- organisms isolated from an aseptically obtained culture of fluid or tissue from the superficial/deep/organ space incision;
- superficial/deep/organ space incision that is deliberately opened by a surgeon, attending physician or other designee and is culture positive or not cultured *and* the patient has at least one of the following signs or symptoms: pain or tenderness; localized swelling; redness; or heat; or
- diagnosis of a superficial/deep/organ space incisional SSI by a surgeon or attending physician.

A blinded Central Adjudication Committee (CAC) will judge whether the primary study endpoint has occurred. The CAC will be comprised of 3 orthopaedic surgeons and 1 infectious disease specialist.

3.3 Secondary Study Endpoints

The secondary study endpoints include patients' functional outcome and quality of life, rate of re-operation, antibiotic-related complications, oncologic recurrence and/or metastases, and mortality. Questionnaires will be used to assess both functional outcome and quality of life prior to surgery, as well as at the 3 month, 6 month, and 1 year follow-up time points, as noted in **Figure 1**. Questionnaires include the Musculoskeletal Tumor Society functional score (MSTS) (1987 and 1993 versions) (clinician administered) and the Toronto Extremity Salvage Score (TESS) (patient administered). The MSTS-87, MSTS-93, and TESS surveys are based on the commonly accepted functional scoring systems in Orthopaedic Oncology publications.[20-22] Antibiotic-related complications, such as gastrointestinal infections, fungal infections, etc., will also be recorded on patient case report forms (CRFs), as will the number of re-operations and the mortality rate of study participants.

4 Subject Selection and Withdrawal

4.1 Inclusion Criteria

Patients who satisfy all of the eligibility criteria outlined below are to be included in the PARITY study:

1) Males and females 12 years of age or older;
2) Primary bone malignancies or benign aggressive tumors of the femur or tibia, soft-tissue sarcomas which have invaded the femur or tibia, or oligometastatic bone disease of the femur or tibia in a patient expected to live at least one year post-operatively;
3) Treatment by excision and endoprosthetic reconstruction of the femur or tibia*; and
4) Provision of informed consent.

* *Expandable prostheses are acceptable.*

4.2 Exclusion Criteria

Patients who meet any of the following criteria are not to be included in the PARITY study:

1) Current known Methicillin-resistant Staphylococcus Aureus (MRSA), or Vancomycin Resistant Enterococcus (VRE) skin colonization*;
2) Documented anaphylaxis or angioedema to penicillin or the study antibiotics [cefazolin, or equivalent gram-positive coverage (i.e., cefuroxime)];

3) Current surgical procedure is a revision surgery for implant failure or infection;
4) Prior local infection within the surgical field of the limb**;
5) Current known immunologically-deficient disease conditions (not including recent chemotherapy)***;
6) Known renal insufficiency with estimated creatinine clearance (eGRF) of less than 54 mL/min;
7) Reconstruction to include a structural allograft;
8) Likely problems, in the judgement of the investigator, with maintaining follow-up; and
9) Enrolled or previously randomized in a competing study.

** unable to safely randomize antibiotics in these patients; ** higher risk of infection (vs. baseline) in patients undergoing revision or with prior infection; *** acquired immunodeficiency conditions (ie. HIV, prior splenectomy) or inherited immunodeficiency diseases (ie. Agammaglobulinemia or Severe Combined Immunodeficiency Disorder).*

4.3 Subject Recruitment and Screening

Each clinical site will have a locally responsible investigator who will oversee the administration of the trial at the local level. The treating physicians at each site will identify potentially eligible patients upon presentation with a tumor of the femur or tibia. A resident or a delegate will be responsible for obtaining informed consent. All patients who meet the inclusion criteria will be registered and failure to randomize patients will be documented. All patients will be screened for eligibility and documented as: 1) eligible and included, 2) eligible and missed, and 3) excluded. The CAC will adjudicate all situations where eligibility is in doubt. The Research Coordinator will be responsible for completing the relevant case report forms and screening logs, conducting follow-up visits with each patient, and ensuring completed forms are scanned into the electronic Data Management System (iDataFax). **Figure 1** outlines this process.

Upon receiving their respective Research Ethics Board (REB) approval, participating Orthopaedic Oncologists at each center will be educated on the process of patient enrolment for our study. Access credentials to an internet based randomization website will be provided along with a consent package including a general form for patient demographics, tumor grade and stage, neoadjuvant treatment and proposed adjuvant treatment. At the time of procedure consent, patients meeting inclusion criteria will be introduced to the study and consent or refusal obtained. Data on the skin prep used, the type and lot of prosthetic, the usage of antibiotic cement, and operative time will also be collected. Prior to the surgeon filling out the pre-operative orders for antibiotics, the internet based randomization program will be utilized to determine the antibiotic duration. For patients who are allocated to the long-term antibiotic group, discharge will

be defined as the date of discharge from the Orthopaedic surgery acute floor to final destinations of home, rehabilitation, or a medical unit for a non-Orthopaedic, non-infection related surgical complication.

4.4 Early Withdrawal of Subjects

4.4.1 When and How to Withdraw Subjects

Patients will only be withdrawn for the following scenarios:

- If patients withdraw consent for participation; or
- If patients are deemed loss to follow-up after all exhaustive measures have been taken to locate the patient.

The reasons for patient withdrawal from the trial will be documented. Patients will not be withdrawn if the study protocol was not adhered (e.g., occurrence of protocol deviations, missed follow-up visits, etc.).

4.4.2 Data Collection and Follow-up for Withdrawn Subjects

To maximize the integrity of the data, all possible attempts will be made to collect as much data as possible and to reduce loss to follow-up (Section 6.9). If a patient wishes to withdraw their consent from the study, the following strategies will be used to reduce the demands of the study and help to retain the subject:

- Ask the patient if you can still collect clinical data from their medical and hospital charts; and
- Ask the patient if you may contact them by telephone to ask about the primary and secondary outcomes.

Patients should not be deemed lost to follow-up until the 12 month visit is due and all attempts to contact the patient have been exhausted.

5 Study Interventions

5.1 Allocation for the Study

The patients will be randomized to either short-term duration or long-term duration antibiotics. Allocation for our study will be concealed using a centralized 24-hour computerized randomization system. Patients will be the unit of randomization. Randomization will occur in random permuted blocks with varying block sizes of two or four based on tumor location (i.e., tibia or femur). Based upon the international survey of surgeons and current evidence, randomization will be stratified for the following variables: 1) location of tumor (femur vs. tibia) and 2) center.

5.2 Antibiotic Regimens

Pre-Operative Antibiotic Regimens

Adult patients will receive 2g of intravenous cefazolin pre-operatively (within 60 minutes of the procedure). Pediatric patients (less than 18 years old) will receive a weight-based dose of intravenous cefazolin based on 100mg/kg/day (33mg/kg/dose) with a maximum single dose of 2g pre-operatively within 60 minutes of the procedure. No other antibiotics will be administered pre-operatively.

Intra-Operative Antibiotic Regimens

Adult patients will receive 2g of intravenous cefazolin every 3-4 hours intra-operatively. Pediatric patients (less than 18 years old) will receive a weight-based dose of intravenous cefazolin based on 100mg/kg/day (33mg/kg/dose) with a maximum single dose of 2g intra-operatively every 3-4 hours. No other antibiotics will be administered intra-operatively.

Post-Operative Antibiotic Regimens

Patients will either be randomized to either the short-arm antibiotic regimen or the long-arm antibiotic regimen.

Adult patients randomized to the short-arm regimen will receive 2g of intravenous cefazolin post-operatively every 8 hours for 24 hours, followed by intravenous saline for an additional 4 days, or until hospital discharge if acute care stay is less than 5 days. Conversely, adult patients randomized to the long-arm regimen will receive 2g of intravenous cefazolin post-operatively every 8 hours over 5 days (maximum), or until hospital discharge if acute care stay is less than 5 days. No other antibiotics will be administered post-operatively.

Pediatric patients (less than 18 years old) randomized to the short-arm regimen will receive intravenous cefazolin based on 100mg/kg/day (33mg/kg/dose) every 8 hours for 24 hours (with a maximum single dose of 2g) followed by intravenous saline for 4 additional days or until hospital discharge if acute care stay is less than 5 days. Conversely, pediatric patients (less than 18 years old) randomized to the long-arm regimen will receive intravenous cefazolin based on 100mg/kg/day (33mg/kg/day) every 8 hours for 5 days (with a maximum single dose of 2g) or until hospital discharge if acute care stay is less than 5 days.

5.3 Blinding

Patients, surgeons and data analysts will be blinded to the antibiotic regimen. Members of the CAC will also be blinded to the study treatment, as will the nurse(s) administering

treatment. The pharmacy designate preparing the solutions will not be blinded however. Patients randomized to short-term antibiotics will receive 4 days of ‘sham’ antibiotics with saline replacing the cefazolin dose. An unblinding procedure will be followed in cases where a patient has an allergic reaction and the surgeon needs to know if the patient received the PARITY antibiotic in order to inform treatment, or if a patient needs to be started on a drug that has the potential to interfere or interact with the PARITY antibiotic. If an AE or SAE occurs within the first 5 days after surgery and the patient requires surgical intervention, the PARITY antibiotics should be stopped and the patient treated per standard of care, and a Protocol Deviation From completed.

5.3.1 Unblinding Procedure

The surgeon will contact either the site pharmacist or the designated Methods Center Research Coordinator and request to be unblinded. The request will be discussed with the Principal Investigator (or one of the Co-Principal Investigators if the PI is not available), and the PI or Co-PI will determine if unblinding is appropriate. The designated Methods Center Research Coordinator will unblind the surgeon by phone. When unblinding occurs, only the surgeon and any medical staff directly involved in the patient’s care should be unblinded (at no time should the site Research Coordinator be unblinded). The designated Methods Center Research Coordinator will complete the PARITY Unblinding Form.

6 Study Procedures

Completed forms recording patient status should be sent electronically to iDataFax promptly via Electronic Data Capture, once each of the defined follow up visits are completed. Completed forms for patient screening, randomization, and surgical interventions should be as soon as they are completed. It is anticipated that completed forms will be sent within seven days. See **Figure 1** for Study Follow-up Timeline.

6.1 Patient Screening and Consent

Research Coordinators and/or Investigators (or their designees) (as permitted by local regulations) should screen all patients attending weekly orthopaedic oncology clinics who are possible candidates for wide resection and endoprosthetic reconstruction of the femur or tibia. The Screening Form should be completed, and patient consent should be obtained using local IRB/REB approved Informed Consent Form in order to participate in the trial. The MSTS-87, MSTS-93, and TESS questionnaires should also be administered to consenting patients at this time, so as to capture patient functionality and quality of life prior to treatment.

The consent form must explicitly state the following possible risks associated with the study drug as listed below:

- stomach cramps;
- nausea and/or vomiting;
- oral candidiasis (oral thrush);
- sore and itchy vagina and/or discharge (vaginal thrush);
- unusual bleeding or bruising;
- difficulty breathing;
- sore mouth and/or throat;
- allergic reactions (itching, drug fever, skin rash, anaphylaxis);
- anemia and/or low blood counts;
- mild or severe skin reactions;
- mild or severe diarrhea; and
- liver or kidney toxicity.

6.2 Randomization

Patients should be randomized after the patient eligibility is established and the patient consent is obtained. Both study consent and operative consent will be obtained at the pre-operative clinic visit, 1-2 weeks before the anticipated date of surgery. At this time, the Randomization and Baseline Characteristics Forms should be completed. Randomization should occur during surgery, prior to case completion, but may occur up to 24 hours after case completion. Randomization will be carried out by the pharmacy designate once the surgical incision has been made. Pharmacy staff will be notified of upcoming study participants both at the time of consent and on the morning of surgery and the assigned antibiotic or placebo solutions will be prepared and shrouded or reconstituted in identical intravenous fluid bags to ensure blinding.

6.3 Surgical Treatment

The surgical management of the tumor will take place as is standard for the participating surgeon. This typically involves resection of the segment of bone affected by tumor with a 2-3 cm bone margin and replacement with a tumor endoprosthesis. A Tumor Characteristics Form, Surgical Report Form, Peri-Operative Form, and Antibiotics Log will be completed at the time of surgery. Patients will be assessed for any adverse events and protocol deviations.

6.4 2-Week Follow-Up

The 2 week follow-up visit should occur in person either in clinic or at the hospital (if prior to discharge). The 2 Week Follow-Up Form should be completed. Patients should be assessed for any AEs, SAEs, infections (surgical site and other), protocol deviations, wound healing and oncologic outcomes such as local recurrence and metastases. The appropriate forms to record these events should be completed as necessary. A Missed

Follow up Form should be completed if the patient misses the follow up visit. An Early Withdrawal Form should only be completed if the patient withdraws their consent.

6.5 6-Week Follow-Up

The 6 week follow-up visit should occur in person either in clinic or at the hospital (if prior to discharge). The 6 week Follow-Up Form should be completed. Patients should be assessed for any AEs, SAEs, infections (surgical site and other), re-operations, protocol deviations, wound healing and oncologic outcomes such as local recurrence and metastases. The appropriate forms to record these events should be completed as necessary. A Missed Follow up Form should be completed if the patient misses the follow up visit. An Early Withdrawal Form should only be completed if the patient withdraws their consent.

6.6 3-Month Follow-Up

The 3 month follow-up visit should occur in person either in clinic or at the hospital (if prior to discharge). The 3 Month Follow-Up Form should be completed. Additionally, MSTS-87, MSTS-93, and TESS Forms should be completed, by the treating physician and patient respectively. Patients should be assessed for any AEs, SAEs, infections (surgical site and other), re-operations, protocol deviations, wound healing and oncologic outcomes such as local recurrence and metastases. The appropriate forms to record these events should be completed as necessary. A Missed Follow up Form should be completed if the patient misses the follow up visit. An Early Withdrawal Form should only be completed if the patient withdraws their consent.

6.7 6-Month Follow-Up

The 6 month follow-up visit should occur in person either in clinic or at the hospital (if prior to discharge). The 6 Month Follow-Up Form should be completed. Additionally, MSTS-87, MSTS-93, and TESS Forms should be completed, by the treating physician and patient respectively. Patients should be assessed for any AEs, SAEs, infections (surgical site and other), re-operations, protocol deviations, wound healing and oncologic outcomes such as local recurrence and metastases. The appropriate forms to record these events should be completed as necessary. A Missed Follow up Form should be completed if the patient misses the follow up visit. An Early Withdrawal Form should only be completed if the patient withdraws their consent.

6.8 9-Month Follow-Up

The 9 month follow-up visit should occur in person either in clinic or at the hospital (if prior to discharge). The 9 Month Follow-Up Form should be completed. Patients should be assessed for any AEs, SAEs, infections (surgical site and other), re-operations, protocol deviations, wound healing and oncologic outcomes such as local recurrence

and metastases. The appropriate forms to record these events should be completed as necessary. A Missed Follow up Form should be completed if the patient misses the follow up visit. An Early Withdrawal Form should only be completed if the patient withdraws their consent.

6.9 1-Year Follow-Up

The 1 year follow-up visit should occur in person either in clinic or at the hospital (if prior to discharge). The 1 Year Follow-Up Form should be completed. Additionally, MSTS-87, MSTS-93, and TESS forms should be completed, by the treating physician and patient respectively. Patients should be assessed for any AEs, SAEs, infections (surgical site and other), re-operations, protocol deviations, wound healing and oncologic outcomes such as local recurrence and metastases. The appropriate forms to record these events should be completed as necessary. A Missed Follow up Form should be completed if the patient misses the follow up visit. An Early Withdrawal Form should only be completed if the patient withdraws their consent.

6.10 Maximization of Follow-up

It is extremely important to maintain patients' follow-up in the trial to ensure the completeness and integrity of the data. We will implement several procedures to limit loss of follow up, as described in **Table 2** below.[23]

Table 2: Strategies to Limit Loss to Follow-Up

1) Individuals should be excluded if they are likely to present problems with follow-up (refer to exclusion criteria).
2) At the time of randomization, as well as their own address and telephone number, each patient should provide the name and address of their primary care physician, and the name, address and phone number of three people at different addresses with whom the patient does not live with who are likely to be aware of the patient's whereabouts. The Research Coordinator should confirm that these numbers are accurate prior to the patient's discharge from hospital.
3) Whenever possible, participants should be given information on endoprosthetic replacements, their complications and the potential treatment effects, expectations for personal benefit from study participation, and be encouraged for adherence with follow-up visits and research protocols.
4) The Study Coordinator should remind patients of upcoming clinic visits.
5) The Study Coordinator should contact patients no less than once every three months to maintain contact and obtain information about any planned change in residence.
6) If a patient refuses to return for a follow-up assessment, study personnel should determine his/her status with regard to revision surgery or any secondary outcome by phone contact with the patient or the patient's family physician.

6.11 Minimization of Crossovers of Surgical Interventions

Crossovers are extremely unlikely between the short- and long-duration antibiotic regimens as patients will be blinded and acute infections are unlikely to occur in the first 5 days after surgery**.** Any patients who do crossover will be analyzed in the group to which they were originally allocated, maintaining the 'intention to treat' approach we plan for the analysis. Our standardization of management protocols will limit co-intervention, and we will document the use of drugs that affect antibiotic metabolism, and major additional procedures that patients undergo while in the hospital or other site infections (urinary tract, Port or PICC line). Research Coordinators will record all medications and therapy used concurrently in included patients on the CRFs.

6.12 Adjudication Requirements

The CAC will adjudicate the following:

- Case eligibility;
- Surgical site infections;
- Antibiotic-related complications;
- Unplanned revision surgery; and
- Mortality.

The CAC will be blinded to allocation. A web-based (password protected) adjudication process will occur using the Global Adjudicator™ platform, outlined in **Figure 2**.

Figure 2: Adjudication Process for Central Outcomes Adjudication Committee (CAC)

1. The Study Coordinator or Research Assistant at the Methods Center will retrieve from each site:
   - X-rays (initial, post-operative and just prior to the revision surgery)
   - Relevant chart notes (operative report, chart notes leading up to revision surgery, and any other relevant laboratory data such as wound cultures).

↓

2. All information will be edited to remove any patient identifiers by the Study Coordinator or Research Assistant at Methods Center. Chart notes will be re-typed for clarity.

↓

3. X-rays will be uploaded to the website as JPEG files and chart notes will be posted as PDF files. Each patient will have a unique identification number on the website. Additional iDataFax forms will also be converted to PDF files and posted for each patient.

↓

4. Each CAC member will independently adjudicate all posted cases. They will log all answers on specific data forms developed, tested and utilized for the study.

↓

5. After all CAC members have completed the web-based adjudication, a consensus table with each member's results will be prepared by the Research Coordinator at the Methods Center.

↓

6. Following each adjudication batch posting, CAC members will communicate via conference call (or in person if coinciding with a scheduled meeting) and discuss all disagreements. The Chair of the CAC will run each session.

↓

7. A consensus will be achieved for all cases. Occasionally, the CAC may request additional information from a site to achieve consensus. In situations where a consensus cannot be achieved, a majority vote will be taken.

7 Statistical Plan

7.1 Sample Size Determination

The determination of sample size is based upon pairwise comparisons for the primary outcome (surgical site infection within 12 months) of long-term vs. short-term antibiotics. The hypothesis is that long-term antibiotics will result in lower rates of surgical site infections (primary outcome). All tests will be two-sided and alpha levels will be set to 0.05 for the primary outcome and to 0.01 for the secondary outcomes.

For the primary outcome, the study will be powered for a superiority design (i.e. it is anticipated that long-term antibiotics are better than short-term antibiotics with respect to surgical site infection rates at 12 months). Although estimates for infection rates with endoprosthetic reconstruction have ranged from 0-25% in the literature (with a weighted mean of 9.5% (95% confidence interval: 8.1% to 11.0%)), the surgical site infection rate identified to date in the PARITY pilot phase is **14%.** Further, our PARITY survey demonstrated that a 50% relative risk reduction would be considered clinically important. Therefore, this trial will be powered to detect an absolute difference of 7% between the treatment arms.

Acceptable study power will be achieved with 300 patients per study arm (total 600 patients), assuming a 14% baseline risk of infection, a 7% absolute difference, an alpha of 0.05, and an assumed study power of 80% (Beta=0.20) (**Table 3**). Our pilot data demonstrate that losses to follow-up, dropouts, and crossovers are negligible in this population and adjustments for their occurrence are not indicated.

Table 3: Sample Sizes Per Group for 80% power, α=0.05.

		Rate in Control Group (24 hours)					
		**7%**	**9%**	**10%**	**13%**	**14%**	**15%**
**Rate in Experimental Group (5 days)**	**5%**	2213	638	435	200	166	141
	**6%**	9540	1209	721	275	220	181
	**7%**	-	2888	1356	392	**300**	239
	**8%**	-	12208	3213	589	426	325
	**9%**	-	-	13495	960	638	460
	**10%**	-	-	-	1774	1035	686

7.2 Statistical Methods

The results of patient demographics and baseline characteristics will be summarized using descriptive summary measures: expressed as mean (standard deviation) or median (interquartile range) for continuous variables and number (percent) for categorical variables. The analysis and reporting of the results of the clinical outcomes

will follow the CONSORT guidelines (www.consort-statement.org). Infection rates and secondary outcomes will undergo an intention-to-treat analysis.

Primary outcome: The primary analysis will be a Cox proportional hazards analysis stratified by tumor location (tibia or femur) and study center, with time to surgical site infection as the outcome. The proportional hazards assumption of the Cox model will be assessed. Estimates of treatment effects will be reported as hazard ratios (HR) with corresponding 95% CI and associated p-values. Kaplan- Meier curves will be constructed.

Secondary analysis: The following secondary analysis will be performed based on the primary outcome as follows: to adjust for a potential residual baseline imbalance, a Cox regression model will be conducted including the following factors as covariates: total operative time, tumor location, chemotherapy regimen, diabetes, and radiation treatment. The results will be reported as HR (95% CI) and associated p-value. Kaplan-Meier curves will be constructed.

Secondary outcomes: The study will estimate the effect of long-term antibiotics versus short-term antibiotics on patient functional outcomes and quality of life (TESS, MSTS-87 and MSTS-93) at follow-up with linear regression models, unadjusted and adjusted for limb replacement (tibia or femur) and center. The hypothesis is that long-term antibiotics will result in improved patient functional outcomes.

The effect of long-term antibiotics versus short-term antibiotics on rates of antibiotic-related complications at follow-up will be explored using descriptive statistics. The hypothesis is that long-term antibiotics will result in more antibiotic-related complications. These include Clostridium difficile associated colitis and life threatening toxic megacolon, opportunistic fungal infections, indwelling-catheter related sepsis, and seizures.

The effect of long-term antibiotics versus short-term antibiotics on rates of oncologic events and mortality at follow-up will be explored using descriptive statistics.

Subgroup analyses: Subgroup analyses will also be conducted for infection rates within each type of tumor (Ewing's, Osteosarcoma, Chondrosarcoma, Giant Cell Tumor) and tumor location (proximal femur, distal femur and proximal tibia). However, due to inadequate sample size and power to conduct this analysis, these results will be used solely for generating hypotheses for future investigations.[24]

Interim analysis: We will not conduct an interim analysis, as trials stopped early for benefit are at risk for systematically overestimating treatment effects.

Table 4 below summarizes the primary and secondary objectives, hypotheses, measures and planned analyses.

Table 4: Statistical Analysis Plan Summary

Primary Objective	
**Objective**	To determine if long-term antibiotics result in decreased surgical site infection rates compared to short-term antibiotics
**Outcome**	Time to surgical site infection within one year
**Statistical Hypothesis**	Null hypothesis: there is no difference in infection rates between the two treatment arms.<br>Alternative hypothesis: there is a difference in infection rates between the two treatment arms.
**Analysis**	**Primary:** Stratified Cox proportional hazards regression analysis for primary outcome, reported as hazard ratios.<br>**Secondary:**<br>To adjust for a potential residual baseline imbalance, a Cox regression model will be conducted including total operative time, tumor location, chemotherapy regimen, diabetes and radiation as covariates.
**Measure**	CDC Criteria for Surgical Site Infection
Secondary Objectives	
**1. Functional outcomes**	
**Objective**	To determine if long-term or short-term antibiotics affect patient functional outcomes
**Outcome**	Changes in patient functional outcomes and quality of life within one year
**Statistical Hypothesis**	Null hypothesis: There is no difference in functional outcomes between the two treatment arms.<br>Alternative hypothesis: There is a difference in functional outcomes between the two treatment arms.
**Analysis**	Linear regression models, unadjusted and adjusted using the following covariates:<br>1. tumor location (tibia vs. femur)<br>2. center
**Measure**	The following patient Case Report Forms:<br>- TESS<br>- MSTS-87<br>- MSTS-93
**2. Antibiotic related complications**	
**Objective**	To determine whether long-term or short-term antibiotics affect patient antibiotic related adverse events
**Outcome**	Changes in antibiotic related adverse events experienced by patients within one year
**Analysis**	Descriptive statistics
**Measure**	Documented adverse events (via patient Case Report Forms)

8 Safety and Adverse Events

8.1 Definitions

8.1.1 Adverse Event

An ***adverse event*** (AE) is any symptom, sign, illness or experience that develops or worsens in severity during the course of the study.

The following are expected possible event and therefore are NOT considered ***adverse events***:

Surgical Events	Post-Op Events
Bleeding requiring red blood cell transfusion	Drain falls out
Bleeding requiring platelet transfusion	Wound drainage
Fracture requiring repair	Wound breakdown
Implant breakage	
Inter-operative vascular bypass	
Nerve damage	
Nerve repair	
Nerve transplant	
Positive margin	
Tumor spillage	
Unplanned flap reconstruction	
Unplanned skin graft	
Vascular damage	

8.1.2 Serious Adverse Event

Adverse events are classified as serious or non-serious. A ***serious adverse event*** is any AE that is:

- fatal;
- life-threatening;
- requires or prolongs hospital stay;
- results in persistent or significant disability or incapacity;
- a congenital anomaly or birth defect; or
- an important medical event.

8.1.3 Unanticipated Problems Resulting in Risk to Subjects or Others

Any incident, experience, or outcome that meets <u>all</u> of the following criteria:

- unexpected in nature, severity, or frequency (i.e. not described in study-related documents such as the IRB-approved protocol or consent form, the investigators brochure, etc.);
- related or possibly related to participation in the research (i.e. possibly related means there is a reasonable possibility that the incident experience, or outcome may have been caused by the procedures involved in the research); and
- suggests that the research places subjects or others <u>at greater risk of harm</u> (including physical, psychological, economic, or social harm).

Unanticipated problems resulting in risk to volunteers or others encompass more than what one usually thinks of as adverse events. “Problems involving risk” may not necessarily result in harm. For example, misplacing a volunteer’s study records containing identifiable private information introduces the risk of breach of confidentiality. Confidentiality may or may not be breached, but either way this would be a reportable event. Risks to others must also be reported. For example, an unexpected outburst during questionnaire administration by a volunteer that puts study staff at risk would be a reportable event.

8.2 Reporting of Adverse Events, Serious Adverse Events, and Unanticipated Problems Resulting in Risk to Subjects or Others

All adverse events, serious adverse events, and unanticipated problems resulting in risk to subjects or others are to be reported to the Methods Center immediately.

8.2.1 Investigator Reporting: Notifying the Methods Center

Any SAEs must be reported to the Methods Center by completing the Adverse Events Form and indicating that the adverse event was serious, then submitting it to iDataFax. The investigator will keep a copy of this form on file at the study site. Significant new information on ongoing serious adverse events should be provided promptly to the Methods Center by updating the AE form.

Unanticipated problems resulting in risk to subjects or others are to be reported to the Methods Center by either fax or email.

8.2.2 Site Investigator ─ IRB/REB Reporting

Investigators are responsible for reporting AEs, SAEs, and unanticipated problems resulting in risk to subjects or others to their local IRB/REB. Investigators are responsible for complying with their local IRB’s/REB’s reporting requirements. Copies of each report and documentation of IRB/REB notification and receipt will be kept in the investigator’s study file.

8.2.3 Data Safety Monitoring Board (DSMB)

The DSMB will monitor the trial, review quarterly quality control and safety reports, and meet annually.[25, 26] The Committee members will be independent of the trial, free of conflicts with any of the investigative team and will consist of a clinical trial methodologist, a statistician and Orthopaedic Surgeons. The terms of reference and functions are derived from the principles established by the Data and Safety Monitoring Boards: Lessons, Ethics, Statistics (DAMOCLES) Study Group charter.

9 Data Handling and Record Keeping

9.1 Confidentiality

Information about study subjects will be kept confidential and managed according to the requirements of the Health Insurance Portability and Accountability Act of 1996 (HIPAA). Those regulations require a signed subject authorization informing the subject of the following:

- What protected health information (PHI) will be collected from subjects in this study;
- Who will have access to that information and why;
- Who will use or disclose that information; and
- The rights of a research subject to revoke their authorization for use of their PHI.

In the event that a subject revokes authorization to collect or use PHI, the investigator, by regulation, retains the ability to use all information collected prior to the revocation of subject authorization. For subjects that have revoked authorization to collect or use PHI, attempts should be made to obtain permission to collect at least vital status (i.e. that the subject is alive) at the end of their scheduled study period.

9.2 Case Report Forms

The CRFs are the primary data collection instrument for the study. All data requested on the CRF must be recorded. All missing data must be explained. If a space on the CRF is left blank because the procedure was not done or the question was not asked, write “N/D”. If the item is not applicable to the individual case, write “N/A”. Sites will receive an iDataFax Manual which includes detailed instructions for entering data using iDataFax.

10 Ethical Considerations

This study is to be conducted according to US and international standards of Good Clinical Practice (FDA Title 21 part 312 and International Conference on Harmonization

guidelines), applicable government regulations, and institutional research policies and procedures.

This protocol and any amendments will be submitted to a properly constituted independent REB or IRB, in agreement with local legal prescriptions, for formal approval of the study conduct. The decision of the REB /IRB concerning the conduct of the study will be made in writing to the investigator and a copy of this decision will be provided to the Methods Center before commencement of this study.

All subjects for this study will be provided a consent form describing this study and providing sufficient information for subjects to make an informed decision about their participation in this study. The consent form will be submitted with the protocol for review and approval by the REB /IRB for the study. The formal consent of a subject, using the REB /IRB-approved consent form, must be obtained before that subject undergoes any study procedure. The consent form must be signed by the subject or legally authorized representative, and the investigator-designated research professional obtaining the consent.

11 References

1. Jeys L, Grimer R The long-term risks of infection and amputation with limb salvage surgery using endoprostheses. *Recent Results Cancer Res* 2009;179:75-84.:75-84.
2. Morii T, Yabe H, Morioka H, Beppu Y, Chuman H, Kawai A, Takeda K, Kikuta K, Hosaka S, Yazawa Y, Takeuchi K, Anazawa U, Mochizuki K, Satomi K Postoperative deep infection in tumor endoprosthesis reconstruction around the knee. *J Orthop Sci* 2010;15:331-9.
3. Grimer RJ, Belthur M, Chandrasekar C, Carter SR, Tillman RM Two-stage revision for infected endoprostheses used in tumor surgery. *Clin Orthop Relat Res* 2002:193-203.
4. Jeys LM, Grimer RJ, Carter SR, Tillman RM Risk of amputation following limb salvage surgery with endoprosthetic replacement, in a consecutive series of 1261 patients. *Int Orthop* 2003;27:160-3.
5. Wirganowicz PZ, Eckardt JJ, Dorey FJ, Eilber FR, Kabo JM Etiology and results of tumor endoprosthesis revision surgery in 64 patients. *Clin Orthop Relat Res* 1999:64-74.
6. Akahane T, Shimizu T, Isobe K, Yoshimura Y, Fujioka F, Kato H Evaluation of postoperative general quality of life for patients with osteosarcoma around the knee joint. *J Pediatr Orthop B* 2007;16:269-72.
7. Abudu A, Carter SR, Grimer RJ The outcome and functional results of diaphyseal endoprostheses after tumour excision. *J Bone Joint Surg* 1996; 78B:652-657.
8. Abudu A, Grimer RJ, Tillman RM, Carter SR Endoprosthetic replacement of the distal tibia and ankle joint for aggressive bone tumours. *Int Orthop* 1999;23:291-294.
9. Grimer RJ, Carter SR, Tillman RM, Sneath RS, Walker PS, Unwin PS, Shewell PC Endoprosthetic replacement of the proximal tibia. *J Bone Jointt Surg* 1999;81B:488-494.
10. Hasan K, Racano A, Deheshi B, Farrokhyar F, Wunder J, Ferguson P, Holt G, Schwartz H, Petrisor B, Bhandari M, Ghert M Prophylactic antibiotic regimens in tumor surgery (PARITY) survey. 2011. *Manuscript submitted for publication*.
11. Bratzler DW, Houck PM Antimicrobial prophylaxis for surgery: an advisory statement from the National Surgical Infection Prevention Project. *Clin Infect Dis* 2004;38:1706-15.
12. Zhanel GG, Adam HJ, Low DE, Blondeau J, DeCorby M, Karlowsky JA, Weshnoweski B, Vashisht R, Wierzbowski A, Hoban DJ Antimicrobial susceptibility of 15,644 pathogens from Canadian hospitals: results of the CANWARD 2007-2009 study. *Diagn Microbiol Infect Dis* 2011;69:291-306.

13. Costelloe C, Metcalfe C, Lovering A, Mant D, Hay AD Effect of antibiotic prescribing in primary care on antimicrobial resistance in individual patients: systematic review and meta-analysis. *BMJ* 2010;340:c2096. doi: 10.1136/bmj.c2096.:c2096.

14. Bergogne-Berezin E Treatment and prevention of antibiotic associated diarrhea. *Int J Antimicrob Agents* 2000;16:521-6.

15. Weiss K, Bergeron L, Bernatchez H, Goyette M, Savoie M, Thirion D Clostridium difficile-associated diarrhoea rates and global antibiotic consumption in five Quebec institutions from 2001 to 2004. *Int J Antimicrob Agents* 2007;30:309-14.

16. Shigemura K, Tanaka K, Adachi M, Yamashita M, Arakawa S, Fujisawa M Chronological change of antibiotic use and antibiotic resistance in Escherichia coli causing urinary tract infections. *J Infect Chemother* 2011.

17. Hueston WJ, Dickerson L Antibiotic resistance and the need for the rational use of antibiotics. *J Med Liban* 2001;49:246-56.

18. Christiaens TC, Digranes A, Baerheim A The relation between sale of antimicrobial drugs and antibiotic resistance in uropathogens in general practice. *Scand J Prim Health Care* 2002;20:45-9.

19. Horan TC, Gaynes RP, Martone WJ, Jarvis WR, Emori TG CDC definitions of nosocomial surgical site infections, 1992: a modification of CDC definitions of surgical wound infections. *Am J Infect Control* 1992;20:271-274.

20. Davis AM, Wright JG, Williams JI, Bombardier C, Griffin A, Bell RS Development of a measure of physical function for patients with bone and soft tissue sarcoma. *Qual Life Res* 1996;5:508-16.

21. Schreiber D, Bell RS, Wunder JS, O'Sullivan B, Turcotte R, Masri BA, Davis AM Evaluating function and health related quality of life in patients treated for extremity soft tissue sarcoma. *Qual Life Res* 2006;15:1439-46.

22. Tunn PU, Pomraenke D, Goerling U, Hohenberger P Functional outcome after endoprosthetic limb-salvage therapy of primary bone tumours--a comparative analysis using the MSTS score, the TESS and the RNL index. *Int Orthop* 2008;32:619-25.

23. Sprague S, Leece P, Bhandari M, Tornetta P, Schemitsch E, Swiontkowski M. Limiting loss to follow-up in a multicenter randomized trial in orthopaedic surgery. *Control Clin Trials* 2003; 24: 719-725.

24. Assmann SF, Pocock SJ, Enos LE, Kasten LE Subgroup analysis and other (mis)uses of baseline data in clinical trials. *Lancet* 2000;355:1064-9.

25. Friedman L The NHLBI model: a 25 year history. *Stat Med* 1993;12:425-31.

26. Slutsky AS, Lavery JV Data safety and monitoring boards. *N Engl J Med* 2004;350:1143-7.

Protocol Version 6.1 (Cefuroxime)

(Follows)

PARITY | PROPHYLACTIC ANTIBIOTIC REGIMENS IN TUMOR SURGERY

Prophylactic Antibiotic Regimens in Tumor Surgery (PARITY): A Multi-Center Randomized Controlled Study Comparing Alternative Antibiotic Regimens in Patients Undergoing Tumor Resections with Endoprosthetic Replacements

Methods Center: Center for Evidence-Based Orthopaedics (CEO)
McMaster University
293 Wellington Street North
Suite 110
Hamilton, Ontario L8L 8E7

Funding Sponsors: Orthopaedic Research & Education Foundation / Musculoskeletal Tumor Society (OREF/MSTS)
Physicians’ Services Incorporated (PSI)
Canadian Cancer Society Research Institute (CCSRI)
Canadian Institutes of Health Research (CIHR)

TABLE OF CONTENTS

List of Abbreviations ..... **iv**

Study Summary **1**

1 Introduction **2**

1.1 Background ***2***

1.2 Preclinical Data ***2***

1.2.1 Best Evidence for Infection Rates 2

1.2.2 Lack of Consensus in Antibiotic Regimens and Global Interest in a 4

1.2.3 Complications of Antibiotic Overuse 4

2 Study Objectives **6**

2.1 Primary Questions ***6***

2.2 Secondary Questions ***6***

3 Study Design **6**

3.1 Published Survey Results Show ***8***

3.2 Primary Study Endpoints ***8***

3.3 Secondary Study Endpoints ***9***

4 Subject Selection and Withdrawal **9**

4.1 Inclusion Criteria ***9***

4.2 Exclusion Criteria ***9***

4.3 Subject Recruitment and Screening ***10***

4.4 Early Withdrawal of Subjects ***11***

4.4.1 When and How to Withdraw Subjects 11

4.4.2 Data Collection and Follow-Up for Withdrawn Subjects 11

5 Study Interventions **11**

5.1 Allocation for the Study ***11***

5.2 Antibiotic Regimens ***12***

5.3 Blinding ***13***

5.3.1 Unblinding Procedure 13

6 Study Procedures **13**

6.1 Patient Screening and Consent ... 13
6.2 Randomization ... 14
6.3 Surgical Treatment ... 14
6.4 2-Week Follow-Up ... 14
6.5 6-Week Follow-Up ... 15
6.6 3-Month Follow-Up ... 15
6.7 6-Month Follow-Up ... 15
6.8 9-Month Follow-Up ... 15
6.9 1-Year Follow-Up ... 16
6.10 Maximization of Follow-Up ... 16
6.11 Minimization of Crossovers of Surgical Interventions ... 17
6.12 Adjudication Requirements ... 17
7 Statistical Plan ... 19
7.1 Sample Size Determination ... 19
7.2 Statistical Methods ... 19
8 Safety and Adverse Events ... 22
8.1 Definitions ... 22
8.1.1 Adverse Event ... 22
8.1.2 Serious Adverse Event ... 22
8.1.3 Unanticipated Problems Resulting in Risk to Subjects or Others ... 22
8.2 Reporting of Adverse Events, Serious Adverse Events, and ... 23
8.2.1 Investigator Reporting: Notifying the Methods Center ... 23
8.2.2 Site Investigator – IRB/REB Reporting ... 23
8.2.3 Data Safety Monitoring Board (DSMB) ... 24
9 Data Handling and Record Keeping ... 24
9.1 Confidentiality ... 24
9.2 Case Report Forms ... 24
10 Ethical Considerations ... 24
11 References ... 26

List of Abbreviations

Abbreviations are listed in alphabetical order

AE: Adverse Event
CAC: Central Adjudication Committee
CDC: Centers for Disease Control and Prevention
CRF: Case Report Form
DSMB: Data Safety Monitoring Board
FDA: Food and Drug Administration
GCP: Good Clinical Practice
HIPAA: Health Insurance Portability and Accountability Act
IRB: Institutional Review Board
MSTS: Musculoskeletal Tumor Society Questionnaire
PHI: Protected Health Information
PI: Principal Investigator
RCT: Randomized Controlled Trial
REB: Research Ethics Board
SAE: Serious Adverse Event
SSI: Surgical Site Infection
TESS: Toronto Extremity Salvage Score

Study Summary

Title	Prophylactic Antibiotic Regimens in Tumor Surgery (PARITY): A Multi-Center Randomized Controlled Study Comparing Alternative Antibiotics Regimens in Patients Undergoing Tumor Resections with Endoprosthetic Replacements
Short Title	PARITY
Methodology	Multi-Center, Blinded, Randomized Trial
Study Duration	December 2012 to March 2021
Study Center(s)	Multi-Center
Primary Study Question	In patients undergoing surgical excision and endoprosthetic reconstruction of the femur or tibia, is a long-term (5 days) post-operative antibiotic regimen more effective at decreasing the rate of infection when compared to a short-term (24 hours) post-operative antibiotic regimen?
Number of Subjects	600
Diagnosis and Main Inclusion Criteria	Primary malignant or benign aggressive bone tumors of the femur or tibia, or soft-tissue sarcomas of the lower extremity which have invaded the femur or tibia, or oligometastatic bone disease of the femur or tibia that requires surgical excision and endoprosthetic reconstruction
Study Product, Dose, Route, Regimen	Antibiotic regimens: intravenous cefuroxime for 24 hours and 5 days

1 Introduction

This document is a protocol for a multi-center, blinded, randomized controlled trial, using a parallel two-arm design, to investigate whether a long-term (5 days) post-operative antibiotic regimen will decrease the rate of infection among patients being surgically treated for a tumor in the femur or tibia when compared to a short-term (24 hours) post-operative antibiotic regimen. The rationale for this study is fuelled by: 1) increased infection rate outcomes in bone tumor surgery compared to general arthroplasty; 2) a lack of consensus among Orthopaedic Oncologists regarding the most effective prophylactic antibiotic regimen; 3) a lack of randomized controlled trial (RCT) evidence; and 4) extensive investigator support for the proposed trial.

1.1 Background

Limb salvage surgery is the standard of care in the management of sarcoma of the long-bones. Advances in chemotherapeutic regimens and imaging techniques allow for wide resection and functional reconstruction in the 95% of patients. The most common type of long-bone reconstruction involves the use of a tumor prosthesis, or endoprostheses. Due to the complexity and length of surgical resection and reconstruction, as well as the immunocompromised nature of patients treated with chemotherapy, the risk for infection remains high.[1, 2] Infection following endoprosthetic reconstruction is a devastating complication that requires staged revision surgery and long-term intravenous antibiotics.[3] The risk for recurrent infection remains high, as does the risk for ultimate amputation.[1, 4, 5] However, the most effective antibiotic regimen in preventing post-operative infections remains controversial, and the current state of practice varies widely, particularly with respect to antibiotic duration. Moreover, patients' quality of life and function following infection are dramatically impacted, as are health care costs.[6] Strategies to optimize prevention of infection and quality of life, while mitigating health care costs are needed.

1.2 Preclinical Data

1.2.1 Best Evidence for Infection Rates

A systematic review was performed comparing the infection rate outcomes reported following the surgical treatment of primary long-bone tumors (malignant and benign aggressive) by excision and endoprosthetic reconstruction. The literature search was conducted of the Medline, EMBASE, and all EBM Reviews (including Cochrane) databases, as well as the proceedings for past American Society for Clinical Oncology (ASCO) Annual Meetings. The initial search generated 3898 titles. Of the 3898 titles, 48 eligible papers were identified and are listed below in **Table 1**. The deep infection rates ranged from 0% to 25.0% with a weighted mean of 9.5% (95% confidence interval:

8.1% to 11.0%). Those papers that reported antibiotic regimens varied significantly from 'intra-operative dosing only' to 'greater than 72 hours'.[7-9]

Table 1: Deep Infection Rates Reported by Systematic Review

Study	Year	Number	Deep infection rate
Lee et al.	1990	17	0.0%
Roberts et al.	1991	133	5.3%
Horowitz et al.	1991	12	25.0%
Eckardt et al.	1991	68	1.5%
Shih et al.	1993	61	6.6%
Morris et al.	1995	31	3.2%
Malawer et al.	1995	51	19.6%
Zehr et al.	1996	17	5.9%
Abudu et al.	1996	16	0.0%
Abudu et al.	1999	5	20.0%
Lee et al.	1999	6	16.7%
Grimer et al.	1999	151	18.5%
Kawai et al.	1999	32	6.3%
Kabukcuoglu et al.	1999	54	1.9%
Natarajan et al.	2000	6	16.7%
Ilyas et al.	2000	15	13.3%
Ilyas et al.	2001	48	8.3%
Donati et al.	2001	25	4.2%
Wunder et al.	2001	64	6.3%
Sokolov	2002	38	10.5%
Ilyas et al.	2002	15	6.7%
Bickels et al.	2002	110	5.5%
Anract et al.	2002	9	22.2%
Griffin et al.	2005	99	10.1%
Natarajan et al.	2005	246	6.9%
Jeys et al.	2005	1036	11.9%
Sharma et al.	2006	77	7.8%
Farid et al.	2006	52	3.8%
Orlic et al.	2006	82	4.9%
Gosheger et al.	2006	250	12.0%
Sharma et al.	2007	112	9.8%
Myers et al.	2007	194	19.6%
Sim et al.	2007	50	12.00%
Finstein et al.	2007	62	4.80%
Myers et al.	2007	335	9.6%
Akahane et al.	2007	11	9.1%
Gitelis et al.	2008	80	2.5%

**Guo et al.**	2008	104	6.7%
**Jeys et al.**	2008	530	12.8%
**Sewell et al.**	2009	22	0.0%
**Natarajan et al.**	2009	17	11.8%
**Shekkeris et al.**	2009	6	16.7%
**Chandrasekar et al.**	2009	100	2.0%
**Lee et al.**	2009	256	9.8%
**Morii et al.**	2010	82	12.2%
**Hanna et al.**	2010	23	5.6%
**Hardes et al.**	2010	125	12.80%
**Li et al.**	2011	49	2.0%
**Sewell et al.**	2011	14	7.1%

1.2.2 Lack of Consensus in Antibiotic Regimens and Global Interest in a Randomized Trial

A survey was published addressing the practices of Orthopaedic Oncologists registered with the Musculoskeletal Tumor Society (MSTS) and the Canadian Orthopaedic Oncology Association (CANOOS). From this survey, it was concluded that there is currently a lack of guidelines for the prescription of prophylactic antibiotics in Musculoskeletal Tumor Surgery, which has left Orthopaedic Oncologists with varying opinions and practices.[10] Of the 97 surgeons who received the questionnaire, 72 responded (75% response rate (95% CI: 65.5, 82.5%)). While almost all respondents agreed antibiotic regimens were important in reducing the risk of infection, respondents varied considerably in their choices of antibiotic regimens and dosages. Although 73% (95% CI: 61, 82%) of respondents prescribe a first generation cephalosporin, one in four favours additional coverage with an aminoglycoside and/or Vancomycin. One in three surgeons (95% CI: 25, 48%) believes antibiotics should be discontinued after 24 hours (as recommended by the AAOS for total joint arthroplasty[11]) but 40% (95% CI: 30, 53%) continue antibiotics until the suction drain is removed.

Given the ongoing uncertainty in evidence to guide best practices, 90% (95% CI: 81, 95%) of respondents agreed that they would change their practice if a large randomized controlled trial showed clear benefit of an antibiotic drug regimen different from what they are currently using. Further support for a clinical trial was observed by an overwhelming surgeon interest (87%; 95% CI: 77, 93%) in participating in a multi-center randomized controlled study.

1.2.3 Complications of Antibiotic Overuse

Antibiotic resistance is an increasingly clinically relevant issue both in surgical and infectious disease literature. The Canadian Antibiotic Resistance Alliance (CARA) publishes statistics intended for use by infectious disease physicians and other medical

and surgical specialists.[12] Our systematic review shows that the most common infective pathogen was *staph aureus*. The 2009 Canadian antibiotogram shows that 100% of MSSA (*methicillin sensitive staphylococcus aureus*) is susceptible to cefazolin (Ancef).[12] However, the prevalence of MRSA, versus MSSA, varies by institution and patient population. Zhanel et al. shows that MRSA comprised 27.0% of all *S. aureus* isolates (68.8% were health care associated [HA-MRSA] and 27.6% were community associated [CA-MRSA]).[12] One hundred percent of both community-associated and health care- associated MRSA showed susceptibility to vancomycin and varying susceptibilities to other antimicrobials. Furthermore, prevalence of antibiotic resistance is increasing in Canada. Data from the Canadian Nosocomial Infection Surveillance Program show that the incidence of MRSA as a proportion of all *S. Aureus* has increased from 1% in 1995 to 8% by 2000 and 27% in 2008 as mentioned above.[12]

The vast majority of prosthetic infections are due to gram positive bacteria, and cefazolin also exhibits gram negative coverage. Notably, the CANWARD 2009 antibiotogram shows 37.6% of *E. coli* and 47.6% of *Klebsiella pneumonia* are susceptible to cefazolin.[12] Based on an expert panel of six Orthopaedic Oncologists and three Infectious Disease specialists who were consulted in preparation for this study, it was determined that the ideal study would be a superiority trial to assess whether 2g of cefazolin, or equivalent gram-positive coverage (i.e., 1.5g of cefuroxime, a second-generation cephalosporin) in centers where cefazolin is not routinely used or is unavailable, given intravenously every 8 hours for 5 days or until discharge from acute care (i.e., long-duration) is more effective than that given intravenously every 8 hours for 24 hours (i.e., short-duration). Despite the fact that 11% of respondents in the PARITY Survey responded that they prescribe an aminoglycoside, the Infectious Disease experts on our panel agreed that this type of gram negative coverage does not add more gram negative coverage to that already provided by cefazolin. In addition, our PARITY survey indicated that there is significant concern in the community regarding nephrotoxicity and ototoxicity associated with aminoglycosides.[10]

Antibiotic misuse and overuse in terms of spectrum and duration respectively are considered the main factors in development of antibiotic resistance.[13] When threatened, bacteria evolve to survive, the main mechanisms being genetic mutation, expression of latent resistance genes, or acquisition of genes with resistance determinants.[13] If the antibiotic resistance profile outruns the development of new antibiotics, we are left defenseless against prosthetic infections, which will significantly impact our ability to salvage infected tumor prosthesis and therefore adversely affect patient morbidity and mortality. In addition to the medium to long-term effects of development of antibiotic resistance, a long course of antibiotics itself is not benign. Complications can vary from an inconvenience to a fatality. Possible complications include the development of

clostridium difficile diarrhea and toxic megacolon, opportunistic fungal infections, catheter related infections, and seizures.[14-18]

2 Study Objectives

The objective of this study is to determine whether long-term (5 days) post-operative antibiotics will decrease the rate of infection following lower extremity tumor surgery, when compared to short-term (24 hours) post-operative antibiotics. This objective will be carried out by answering the following questions:

2.1 Primary Questions

In patients undergoing surgical excision and endoprosthetic reconstruction of a tumor in the femur or tibia, do long-term (5 days) prophylactic antibiotics lead to decreased rates of post-operative surgical site infections over 12 months?

2.2 Secondary Questions

In patients surgically treated for tumors in the femur or tibia followed by limb reconstruction using an endoprosthesis, what is the impact of the post-operative antibiotic regimen (24 hours vs. 5 days) on: the development of antibiotic-related complications (i.e., gastrointestinal infections, fungal infections, etc.), patient functional outcome and quality of life, the rate of re-operations, oncologic recurrence and/or metastases, and mortality after one year?

3 Study Design

This study is a multi-center, blinded, randomized controlled trial, using a parallel two-arm design to investigate whether long-term post-operative antibiotic regimens (5 days) will decrease the rate of infection among patients being surgically treated for a tumor in the femur or tibia, when compared to short-term post-operative antibiotics (24 hours).

Patients will be randomized using a 24-hour computerized randomization system that allows random variable block sizes to one of two treatment arms (24 hours or 5 days). The randomization is stratified by: 1) center and 2) location of tumor (femur vs. tibia). The patients will be followed for 1 year after surgery. We will assess infection rates within 12 months after initial surgery across both study arms. Patients, outcome adjudicators and data analysts will be blinded. We will measure function and quality of life pre-operatively, and at 3 months, 6 months, and 1 year post-operatively. The schematic procedure is shown in **Figure 1**.

Figure 1: Trial Conduct Procedure

Patient Recruitment, Randomization and Surgical Interventions

Identification of Patients	Direct referral-within center	Data Collected
Assessment of Patient Eligibility	Study explanation<br>History-review eligibility criteria, and other relevant medical conditions<br>Physical Examination<br>Radiographs	Screening Form
	Informed Consent, if eligible	Informed Consent<br>MSTS-87, MSTS-93, TESS (baseline)
	All eligible patients who consent to the trial	
Randomization	24 hour web-based system<br>Eligibility criteria reviewed again<br>Key patient information recorded<br>Randomization issued to patient	Baseline Form<br>Randomization Form
Surgery	Either short- or long-arm<br>Surgical protocols will be followed	Surgical Form
Follow Up Schedule		
2 Weeks	Assessment of outcome events	Follow-Up Form
6 weeks	Assessment of outcome events	Follow-Up Form
3 Months	Assessment of outcome events	Follow-Up Form<br>MSTS-87, MSTS-93, TESS
6 Months	Assessment of outcome events	Follow-Up Form<br>MSTS-87, MSTS-93, TESS
9 Months	Assessment of outcome events	Follow-Up Form
1 Year	Assessment of outcome events	Follow-Up Form<br>MSTS-87, MSTS-93, TESS

**Follow-Up Forms include AEs, SAEs, infections, reoperations, protocol deviations or wound healing problems, and other appropriate forms*.

3.1 Published Survey Results Show

Infection following endoprosthetic limb reconstruction for sarcoma of the long-bones is a devastating complication. A conducted survey and systematic review show that there are no current best practice guidelines for antibiotic prophylaxis in tumor surgery and that Orthopaedic Oncologists would be interested in enrolling patients in research to inform the development of such guidelines.[10] These findings provide a strong rationale for undertaking a randomized control trial to determine the effects of post-operative antibiotic regimens on infection rate outcomes following bone tumor surgeries of the lower extremities. Implications of this trial may include both fewer endoprosthetic infections as well as fewer antibiotic related complications.

3.2 Primary Study Endpoints

The primary study endpoint is the development of a surgical site infection (SSI) within 12 months following the initial surgery to treat a tumor of the femur or tibia. Patients will be monitored regularly by the treating physician at 2 weeks, 6 weeks, 3 months, 6 months, 9 months, and 1 year.

Surgical site infections will be classified according to the criteria established by the Center for Disease Control (CDC).[19] The CDC defines a SSI as infection occurring within the 30 days following the operative procedure or within 1 year if an implant is in place and the infection appears to be related to the operative procedure. Infection can involve any part of the body, but excludes the skin incision, fascia, or muscle layers, that is opened or manipulated during the operative procedure. The patient must also present with at least one of the following:

- purulent drainage from the superficial/deep/organ space incision;
- organisms isolated from an aseptically obtained culture of fluid or tissue from the superficial/deep/organ space incision;
- superficial/deep/organ space incision that is deliberately opened by a surgeon, attending physician or other designee and is culture positive or not cultured *and* the patient has at least one of the following signs or symptoms: pain or tenderness; localized swelling; redness; or heat; or
- diagnosis of a superficial/deep/organ space incisional SSI by a surgeon or attending physician.

A blinded Central Adjudication Committee (CAC) will judge whether the primary study endpoint has occurred. The CAC will be comprised of 3 orthopaedic surgeons and 1 infectious disease specialist.

3.3 Secondary Study Endpoints

The secondary study endpoints include patients' functional outcome and quality of life, rate of re-operation, antibiotic-related complications, oncologic recurrence and/or metastases, and mortality. Questionnaires will be used to assess both functional outcome and quality of life prior to surgery, as well as at the 3 month, 6 month, and 1 year follow-up time points, as noted in **Figure 1**. Questionnaires include the Musculoskeletal Tumor Society functional score (MSTS) (1987 and 1993 versions) (clinician administered) and the Toronto Extremity Salvage Score (TESS) (patient administered). The MSTS-87, MSTS-93, and TESS surveys are based on the commonly accepted functional scoring systems in Orthopaedic Oncology publications.[20-22] Antibiotic-related complications, such as gastrointestinal infections, fungal infections, etc., will also be recorded on patient case report forms (CRFs), as will the number of re-operations and the mortality rate of study participants.

4 Subject Selection and Withdrawal

4.1 Inclusion Criteria

Patients who satisfy all of the eligibility criteria outlined below are to be included in the PARITY study:

1) Males and females 12 years of age or older;
2) Primary bone malignancies or benign aggressive tumors of the femur or tibia, soft-tissue sarcomas which have invaded the femur or tibia, or oligometastatic bone disease of the femur or tibia in a patient expected to live at least one year post-operatively;
3) Treatment by excision and endoprosthetic reconstruction of the femur or tibia*; and
4) Provision of informed consent.

* *Expandable prostheses are acceptable.*

4.2 Exclusion Criteria

Patients who meet any of the following criteria are not to be included in the PARITY study:

1) Current known Methicillin-resistant Staphylococcus Aureus (MRSA), or Vancomycin Resistant Enterococcus (VRE) skin colonization*;
2) Documented anaphylaxis or angioedema to penicillin or the study antibiotics [cefazolin, or equivalent gram-positive coverage (i.e., cefuroxime)]

3) Current surgical procedure is a revision surgery for implant failure or infection;
4) Prior local infection within the surgical field of the limb**;
5) Current known immunologically-deficient disease conditions (not including recent chemotherapy)***;
6) Known renal insufficiency with estimated creatinine clearance (eGRF) of less than 54 mL/min;
7) Reconstruction to include a structural allograft;
8) Likely problems, in the judgement of the investigator, with maintaining follow-up;
9) Enrolled or previously randomized in a competing study; and
10) Patients who weigh less than or equal to 45kg.

** unable to safely randomize antibiotics in these patients; ** higher risk of infection (vs. baseline) in patients undergoing revision or with prior infection; *** acquired immunodeficiency conditions (ie. HIV, prior splenectomy) or inherited immunodeficiency diseases (ie. Agammaglobulinemia or Severe Combined Immunodeficiency Disorder).*

4.3 Subject Recruitment and Screening

Each clinical site will have a locally responsible investigator who will oversee the administration of the trial at the local level. The treating physicians at each site will identify potentially eligible patients upon presentation with a tumor of the femur or tibia. A resident or a delegate will be responsible for obtaining informed consent. All patients who meet the inclusion criteria will be registered and failure to randomize patients will be documented. All patients will be screened for eligibility and documented as: 1) eligible and included, 2) eligible and missed, and 3) excluded. The CAC will adjudicate all situations where eligibility is in doubt. The Research Coordinator will be responsible for completing the relevant case report forms and screening logs, conducting follow-up visits with each patient, and ensuring completed forms are scanned into the electronic Data Management System (iDataFax). **Figure 1** outlines this process.

Upon receiving their respective Research Ethics Board (REB) approval, participating Orthopaedic Oncologists at each center will be educated on the process of patient enrolment for our study. Access credentials to an internet based randomization website will be provided along with a consent package including a general form for patient demographics, tumor grade and stage, neoadjuvant treatment and proposed adjuvant treatment. At the time of procedure consent, patients meeting inclusion criteria will be introduced to the study and consent or refusal obtained. Data on the skin prep used, the type and lot of prosthetic, the usage of antibiotic cement, and operative time will also be collected. Prior to the surgeon filling out the pre-operative orders for antibiotics, the internet based randomization program will be utilized to determine the antibiotic

duration. For patients who are allocated to the long-term antibiotic group, discharge will be defined as the date of discharge from the Orthopaedic surgery acute floor to final destinations of home, rehabilitation, or a medical unit for a non-Orthopaedic, non-infection related surgical complication.

4.4 Early Withdrawal of Subjects

4.4.1 When and How to Withdraw Subjects

Patients will only be withdrawn for the following scenarios:

- If patients withdraw consent for participation; or
- If patients are deemed loss to follow-up after all exhaustive measures have been taken to locate the patient.

The reasons for patient withdrawal from the trial will be documented. Patients will not be withdrawn if the study protocol was not adhered (e.g., occurrence of protocol deviations, missed follow-up visits, etc.).

4.4.2 Data Collection and Follow-Up for Withdrawn Subjects

To maximize the integrity of the data, all possible attempts will be made to collect as much data as possible and to reduce loss to follow-up (Section 6.9). If a patient wishes to withdraw their consent from the study, the following strategies will be used to reduce the demands of the study and help to retain the subject:

- Ask the patient if you can still collect clinical data from their medical and hospital charts; and
- Ask the patient if you may contact them by telephone to ask about the primary and secondary outcomes.

Patients should not be deemed lost to follow-up until the 12 month visit is due and all attempts to contact the patient have been exhausted.

5 Study Interventions

5.1 Allocation for the Study

The patients will be randomized to either short-term duration or long-term duration antibiotics. Allocation for our study will be concealed using a centralized 24-hour computerized randomization system. Patients will be the unit of randomization. Randomization will occur in random permuted blocks with varying block sizes of two or four based on tumor location (i.e., tibia or femur). Based upon the international survey

of surgeons and current evidence, randomization will be stratified for the following variables: 1) location of tumor (femur vs. tibia) and 2) center.

5.2 Antibiotic Regimens

Pre-Operative Antibiotic Regimens

Adult patients will receive 1.5g of intravenous cefuroxime pre-operatively (within 60 minutes of the procedure). Pediatric patients (less than 18 years old) will receive a weight-based dose of intravenous cefuroxime based on 50mg/kg/day (16mg/kg/dose) with a maximum single dose of 1.5g pre-operatively within 60 minutes of the procedure. No other antibiotics will be administered pre-operatively.

Intra-Operative Antibiotic Regimens

Adult patients will receive 1.5g of intravenous cefuroxime every 3-4 hours intra-operatively. Pediatric patients (less than 18 years old) will receive a weight-based dose of intravenous cefuroxime based on 50mg/kg/day (16mg/kg/dose) with a maximum single dose of 1.5g intra-operatively every 3-4 hours. No other antibiotics will be administered intra-operatively.

Post-Operative Antibiotic Regimens

Patients will either be randomized to either the short-arm antibiotic regimen or the long-arm antibiotic regimen.

Adult patients randomized to the short-arm regimen will receive 1.5g of intravenous cefuroxime post-operatively every 8 hours for 24 hours, followed by intravenous saline for an additional 4 days, or until hospital discharge if acute care stay is less than 5 days. Conversely, adult patients randomized to the long-arm regimen will receive 1.5g of intravenous cefuroxime post-operatively every 8 hours over 5 days (maximum), or until hospital discharge if acute care stay is less than 5 days. No other antibiotics will be administered post-operatively.

Pediatric patients (less than 18 years old) randomized to the short-arm regimen will receive intravenous cefuroxime based on 50mg/kg/day (16mg/kg/dose) every 8 hours for 24 hours (with a maximum single dose of 1.5g) followed by intravenous saline for 4 additional days or until hospital discharge if acute care stay is less than 5 days. Conversely, pediatric patients (less than 18 years old) randomized to the long-arm regimen will receive intravenous cefuroxime based on 50mg/kg/day (16mg/kg/dose) every 8 hours for 5 days (with a maximum single dose of 1.5g) or until hospital discharge if acute care stay is less than 5 days.

5.3 Blinding

Patients, surgeons and data analysts will be blinded to the antibiotic regimen. Members of the CAC will also be blinded to the study treatment, as will the nurse(s) administering treatment. The pharmacy designate preparing the solutions will not be blinded however. Patients randomized to short-term antibiotics will receive 4 days of ‘sham’ antibiotics with saline replacing the cefazolin dose. An unblinding procedure will be followed in cases where a patient has an allergic reaction and the surgeon needs to know if the patient received the PARITY antibiotic in order to inform treatment, or if a patient needs to be started on a drug that has the potential to interfere or interact with the PARITY antibiotic. If an AE or SAE occurs within the first 5 days after surgery and the patient requires surgical intervention, the PARITY antibiotics should be stopped and the patient treated per standard of care, and a Protocol Deviation From completed.

5.3.1 Unblinding Procedure

The surgeon will contact either the site pharmacist or the designated Methods Center Research Coordinator and request to be unblinded. The request will be discussed with the Principal Investigator (or one of the Co-Principal Investigators if the PI is not available), and the PI or Co-PI will determine if unblinding is appropriate. The designated Methods Center Research Coordinator will unblind the surgeon by phone. When unblinding occurs, only the surgeon and any medical staff directly involved in the patient’s care should be unblinded (at no time should the site Research Coordinator be unblinded). The designated Methods Centre Research Coordinator will complete the PARITY Unblinding Form.

6 Study Procedures

Completed forms recording patient status should be sent electronically to iDataFax promptly via Electronic Data Capture, once each of the defined follow up visits are completed. Completed forms for patient screening, randomization, and surgical interventions should be as soon as they are completed. It is anticipated that completed forms will be sent within seven days. See **Figure 1** for Study Follow-up Timeline.

6.1 Patient Screening and Consent

Research Coordinators and/or Investigators (or their designees) (as permitted by local regulations) should screen all patients attending weekly orthopaedic oncology clinics who are possible candidates for resection and endoprosthetic reconstruction of the femur or tibia. The Screening Form should be completed, and patient consent should be obtained using local IRB/REB approved Informed Consent Form in order to participate in the trial. The MSTS-87, MSTS-93, and TESS questionnaires should also be administered to consenting patients at this time, so as to capture patient functionality and quality of life prior to treatment.

The consent form must explicitly state the following possible risks associated with the study drug as listed below:

- stomach cramps;
- nausea and/or vomiting;
- oral candidiasis (oral thrush);
- sore and itchy vagina and/or discharge (vaginal thrush);
- unusual bleeding or bruising;
- difficulty breathing;
- sore mouth and/or throat;
- allergic reactions (itching, drug fever, skin rash, anaphylaxis);
- anemia and/or low blood counts;
- mild or severe skin reactions;
- mild or severe diarrhea; and
- liver or kidney toxicity.

6.2 Randomization

Patients should be randomized after the patient eligibility is established and the patient consent is obtained. Both study consent and operative consent will be obtained at the pre-operative clinic visit, 1-2 weeks before the anticipated date of surgery. At this time, the Randomization and Baseline Characteristics Forms should be completed. Randomization should occur during surgery, prior to case completion, but may occur up to 24 hours after case completion. Randomization will be carried out by the pharmacy designate once the surgical incision has been made. Pharmacy staff will be notified of upcoming study participants both at the time of consent and on the morning of surgery and the assigned antibiotic or placebo solutions will be prepared and shrouded or reconstituted in identical intravenous fluid bags to ensure blinding.

6.3 Surgical Treatment

The surgical management of the tumor will take place as is standard for the participating surgeon. This typically involves resection of the segment of bone affected by tumor with a 2-3 cm bone margin and replacement with a tumor endoprosthesis. A Tumor Characteristics Form, Surgical Report Form, Peri-Operative Form, and Antibiotics Log will be completed at the time of surgery. Patients will be assessed for any adverse events and protocol deviations.

6.4 2-Week Follow-Up

The 2 week follow-up visit should occur in person either in clinic or at the hospital (if prior to discharge). The 2 Week Follow-Up Form should be completed. Patients should be assessed for any AEs, SAEs, infections (surgical site and other), protocol deviations,

wound healing and oncologic outcomes such as local recurrence and metastases. The appropriate forms to record these events should be completed as necessary. A Missed Follow up Form should be completed if the patient misses the follow up visit. An Early Withdrawal Form should only be completed if the patient withdraws their consent.

6.5 6-Week Follow-Up

The 6 week follow-up visit should occur in person either in clinic or at the hospital (if prior to discharge). The 6 week Follow-Up Form should be completed. Patients should be assessed for any AEs, SAEs, infections (surgical site and other), re-operations, protocol deviations, wound healing and oncologic outcomes such as local recurrence and metastases. The appropriate forms to record these events should be completed as necessary. A Missed Follow up Form should be completed if the patient misses the follow up visit. An Early Withdrawal Form should only be completed if the patient withdraws their consent.

6.6 3-Month Follow-Up

The 3 month follow-up visit should occur in person either in clinic or at the hospital (if prior to discharge). The 3 Month Follow-Up Form should be completed. Additionally, MSTS-87, MSTS-93, and TESS Forms should be completed, by the treating physician and patient respectively. Patients should be assessed for any AEs, SAEs, infections (surgical site and other), re-operations, protocol deviations, wound healing and oncologic outcomes such as local recurrence and metastases. The appropriate forms to record these events should be completed as necessary. A Missed Follow up Form should be completed if the patient misses the follow up visit. An Early Withdrawal Form should only be completed if the patient withdraws their consent.

6.7 6-Month Follow-Up

The 6 month follow-up visit should occur in person either in clinic or at the hospital (if prior to discharge). The 6 Month Follow-Up Form should be completed. Additionally, MSTS-87, MSTS-93, and TESS Forms should be completed, by the treating physician and patient respectively. Patients should be assessed for any AEs, SAEs, infections (surgical site and other), re-operations, protocol deviations, wound healing and oncologic outcomes such as local recurrence and metastases. The appropriate forms to record these events should be completed as necessary. A Missed Follow up Form should be completed if the patient misses the follow up visit. An Early Withdrawal Form should only be completed if the patient withdraws their consent.

6.8 9-Month Follow-Up

The 9 month follow-up visit should occur in person either in clinic or at the hospital (if prior to discharge). The 9 Month Follow-Up Form should be completed. Patients should

be assessed for any AEs, SAEs, infections (surgical site and other), re-operations, protocol deviations, wound healing and oncologic outcomes such as local recurrence and metastases. The appropriate forms to record these events should be completed as necessary. A Missed Follow up Form should be completed if the patient misses the follow up visit. An Early Withdrawal Form should only be completed if the patient withdraws their consent.

6.9 1-Year Follow-Up

The 1 year follow-up visit should occur in person either in clinic or at the hospital (if prior to discharge). The 1 Year Follow-Up Form should be completed. Additionally, MSTS-87, MSTS-93, and TESS forms should be completed, by the treating physician and patient respectively. Patients should be assessed for any AEs, SAEs, infections (surgical site and other), re-operations, protocol deviations, wound healing and oncologic outcomes such as local recurrence and metastases. The appropriate forms to record these events should be completed as necessary. A Missed Follow up Form should be completed if the patient misses the follow up visit. An Early Withdrawal Form should only be completed if the patient withdraws their consent.

6.10 Maximization of Follow-Up

It is extremely important to maintain patients' follow-up in the trial to ensure the completeness and integrity of the data. We will implement several procedures to limit loss of follow up, as described in **Table 2** below.[23]

Table 2: Strategies to Limit Loss to Follow-Up

1) Individuals should be excluded if they are likely to present problems with follow-up (refer to exclusion criteria).
2) At the time of randomization, as well as their own address and telephone number, each patient should provide the name and address of their primary care physician, and the name, address and phone number of three people at different addresses with whom the patient does not live with who are likely to be aware of the patient's whereabouts. The Research Coordinator should confirm that these numbers are accurate prior to the patient's discharge from hospital.
3) Whenever possible, participants should be given information on endoprosthetic replacements, their complications and the potential treatment effects, expectations for personal benefit from study participation, and be encouraged for adherence with follow-up visits and research protocols.
4) The Study Coordinator should remind patients of upcoming clinic visits.
5) The Study Coordinator should contact patients no less than once every three months to maintain contact and obtain information about any planned change in residence.
6) If a patient refuses to return for a follow-up assessment, study personnel should determine his/her

status with regard to revision surgery or any secondary outcome by phone contact with the patient or the patient's family physician.

6.11 Minimization of Crossovers of Surgical Interventions

Crossovers are extremely unlikely between the short- and long-duration antibiotic regimens as patients will be blinded and acute infections are unlikely to occur in the first 5 days after surgery**.** Any patients who do crossover will be analyzed in the group to which they were originally allocated, maintaining the 'intention to treat' approach we plan for the analysis. Our standardization of management protocols will limit co-intervention, and we will document the use of drugs that affect antibiotic metabolism, and major additional procedures that patients undergo while in the hospital or other site infections (urinary tract, Port or PICC line). Research Coordinators will record all medications and therapy used concurrently in included patients on the CRFs.

6.12 Adjudication Requirements

The CAC will adjudicate the following:

- Case eligibility;
- Surgical site infections;
- Antibiotic-related complications;
- Unplanned revision surgery; and
- Mortality.

The CAC will be blinded to allocation. A web-based (password protected) adjudication process will occur using the Global Adjudicator^{TM} platform, outlined in **Figure 2**.

Figure 2: Adjudication Process for Central Outcomes Adjudication Committee (CAC)

1. The Study Coordinator or Research Assistant at the Methods Center will retrieve from each site:
   - X-rays (initial, post-operative and just prior to the revision surgery)
   - Relevant chart notes (operative report, chart notes leading up to revision surgery, and any other relevant laboratory data such as wound cultures).

↓

2. All information will be edited to remove any patient identifiers by the Study Coordinator or Research Assistant at Methods Center. Chart notes will be re-typed for clarity.

↓

3. X-rays will be uploaded to the website as JPEG files and chart notes will be posted as PDF files. Each patient will have a unique identification number on the website. Additional iDataFax forms will also be converted to PDF files and posted for each patient.

↓

4. Each CAC member will independently adjudicate all posted cases. They will log all answers on specific data forms developed, tested and utilized for the study.

↓

5. After all CAC members have completed the web-based adjudication, a consensus table with each member's results will be prepared by the Research Coordinator at the Methods Center.

↓

6. Following each adjudication batch posting, CAC members will communicate via conference call (or in person if coinciding with a scheduled meeting) and discuss all disagreements. The Chair of the CAC will run each session.

↓

7. A consensus will be achieved for all cases. Occasionally, the CAC may request additional information from a site to achieve consensus. In situations where a consensus cannot be achieved, a majority vote will be taken.

7 Statistical Plan

7.1 Sample Size Determination

The determination of sample size is based upon pairwise comparisons for the primary outcome (surgical site infection within 12 months) of long-term vs. short-term antibiotics. The hypothesis is that long-term antibiotics will result in lower rates of surgical site infections (primary outcome). All tests will be two-sided and alpha levels will be set to 0.05 for the primary outcome and to 0.01 for the secondary outcomes.

For the primary outcome, the study will be powered for a superiority design (i.e. it is anticipated that long-term antibiotics are better than short-term antibiotics with respect to surgical site infection rates at 12 months). Although estimates for infection rates with endoprosthetic reconstruction have ranged from 0-25% in the literature (with a weighted mean of 9.5% (95% confidence interval: 8.1% to 11.0%)), the surgical site infection rate identified to date in the PARITY pilot phase is **14%.** Further, our PARITY survey demonstrated that a 50% relative risk reduction would be considered clinically important. Therefore, this trial will be powered to detect an absolute difference of 7% between the treatment arms

Acceptable study power will be achieved with 300 patients per study arm (total 600 patients), assuming a 14% baseline risk of infection, a 7% absolute difference, an alpha of 0.05, and an assumed study power of 80% (Beta=0.20) (**Table 3**). Our pilot data demonstrate that losses to follow-up, dropouts, and crossovers are negligible in this population and adjustments for their occurrence are not indicated.

Table 3: Sample Sizes Per Group for 80% power, α=0.05.

		Rate in Control Group (24 hours)					
		**7%**	**9%**	**10%**	**13%**	**14%**	**15%**
**Rate in Experimental Group (5 days)**	**5%**	2213	638	435	200	166	141
	**6%**	9540	1209	721	275	220	181
	**7%**	-	2888	1356	392	**300**	239
	**8%**	-	12208	3213	589	426	325
	**9%**	-	-	13495	960	638	460
	**10%**	-	-	-	1774	1035	686

7.2 Statistical Methods

The results of patient demographics and baseline characteristics will be summarized using descriptive summary measures: expressed as mean (standard deviation) or median (interquartile range) for continuous variables and number (percent) for categorical variables. The analysis and reporting of the results of the clinical outcomes

will follow the CONSORT guidelines (www.consort-statement.org). Infection rates and secondary outcomes will undergo an intention-to-treat analysis.

Primary outcome: The primary analysis will be a Cox proportional hazards analysis stratified by tumor location (tibia or femur) and study center, with time to surgical site infection as the outcome. The proportional hazards assumption of the Cox model will be assessed. Estimates of treatment effects will be reported as hazard ratios (HR) with corresponding 95% CI and associated p-values. Kaplan- Meier curves will be constructed.

Secondary analysis: The following secondary analysis will be performed based on the primary outcome as follows: to adjust for a potential residual baseline imbalance, a Cox regression model will be conducted including the following factors as covariates: total operative time, tumor location, chemotherapy regimen, diabetes, and radiation treatment. The results will be reported as HR (95% CI) and associated p-value. Kaplan-Meier curves will be constructed.

Secondary outcomes: The study will estimate the effect of long-term antibiotics versus short-term antibiotics on patient functional outcomes and quality of life (TESS, MSTS-87 and MSTS-93) at follow-up with linear regression models, unadjusted and adjusted for limb replacement (tibia or femur) and center. The hypothesis is that long-term antibiotics will result in improved patient functional outcomes.

The effect of long-term antibiotics versus short-term antibiotics on rates of antibiotic-related complications at follow-up will be explored using descriptive statistics. The hypothesis is that long-term antibiotics will result in more antibiotic-related complications. These include Clostridium difficile associated colitis and life threatening toxic megacolon, opportunistic fungal infections, indwelling-catheter related sepsis, and seizures.

The effect of long-term antibiotics versus short-term antibiotics on rates of oncologic events and mortality at follow-up will be explored using descriptive statistics.

Subgroup analyses: Subgroup analyses will also be conducted for infection rates within each type of tumor (Ewing's, Osteosarcoma, Chondrosarcoma, Giant Cell Tumor) and tumor location (proximal femur, distal femur and proximal tibia). However, due to inadequate sample size and power to conduct this analysis, these results will be used solely for generating hypotheses for future investigations.[24]

Interim analysis: We will not conduct an interim analysis, as trials stopped early for benefit are at risk for systematically overestimating treatment effects.

Table 4 below summarizes the primary and secondary objectives, hypotheses, measures and planned analyses.

Table 4: Statistical Analysis Plan Summary

Primary Objective	
**Objective**	To determine if long-term antibiotics result in decreased surgical site infection rates compared to short-term antibiotics
**Outcome**	Time to surgical site infection within one year
**Statistical Hypothesis**	Null hypothesis: there is no difference in infection rates between the two treatment arms.<br>Alternative hypothesis: there is a difference in infection rates between the two treatment arms.
**Analysis**	**Primary:** Stratified Cox proportional hazards regression analysis for primary outcome, reported as hazard ratios.<br>**Secondary:**<br>To adjust for a potential residual baseline imbalance, a Cox regression model will be conducted including total operative time, tumor location, chemotherapy regimen, diabetes and radiation as covariates.
**Measure**	CDC Criteria for Surgical Site Infection
Secondary Objectives	
**1. Functional outcomes**	
**Objective**	To determine if long-term or short-term antibiotics affect patient functional outcomes
**Outcome**	Changes in patient functional outcomes and quality of life within one year
**Statistical Hypothesis**	Null hypothesis: There is no difference in functional outcomes between the two treatment arms.<br>Alternative hypothesis: There is a difference in functional outcomes between the two treatment arms.
**Analysis**	Linear regression models, unadjusted and adjusted using the following covariates:<br>1. tumor location (tibia vs. femur)<br>2. center
**Measure**	The following patient Case Report Forms:<br>- TESS<br>- MSTS-87<br>- MSTS-93
**2. Antibiotic related complications**	
**Objective**	To determine whether long-term or short-term antibiotics affect patient antibiotic related adverse events
**Outcome**	Changes in antibiotic related adverse events experienced by patients within one year
**Analysis**	Descriptive statistics
**Measure**	Documented adverse events (via patient Case Report Forms)

8 Safety and Adverse Events

8.1 Definitions

8.1.1 Adverse Event

An ***adverse event*** (AE) is any symptom, sign, illness or experience that develops or worsens in severity during the course of the study.

The following are expected possible event and therefore are NOT considered ***adverse events***:

Surgical Events	Post-Op Events
Bleeding requiring red blood cell transfusion	Drain falls out
Bleeding requiring platelet transfusion	Wound drainage
Fracture requiring repair	Wound breakdown
Implant breakage	
Inter-operative vascular bypass	
Nerve damage	
Nerve repair	
Nerve transplant	
Positive margin	
Tumor spillage	
Unplanned flap reconstruction	
Unplanned skin graft	
Vascular damage	

8.1.2 Serious Adverse Event

Adverse events are classified as serious or non-serious. A ***serious adverse event*** is any AE that is:

- fatal;
- life-threatening;
- requires or prolongs hospital stay;
- results in persistent or significant disability or incapacity;
- a congenital anomaly or birth defect; or
- an important medical event.

8.1.3 Unanticipated Problems Resulting in Risk to Subjects or Others

Any incident, experience, or outcome that meets all of the following criteria:

- unexpected in nature, severity, or frequency (i.e. not described in study-related documents such as the IRB-approved protocol or consent form, the investigators brochure, etc.);
- related or possibly related to participation in the research (i.e. possibly related means there is a reasonable possibility that the incident experience, or outcome may have been caused by the procedures involved in the research); and
- suggests that the research places subjects or others at greater risk of harm (including physical, psychological, economic, or social harm).

Unanticipated problems resulting in risk to volunteers or others encompass more than what one usually thinks of as adverse events. “Problems involving risk” may not necessarily result in harm. For example, misplacing a volunteer’s study records containing identifiable private information introduces the risk of breach of confidentiality. Confidentiality may or may not be breached, but either way this would be a reportable event. Risks to others must also be reported. For example, an unexpected outburst during questionnaire administration by a volunteer that puts study staff at risk would be a reportable event.

8.2 Reporting of Adverse Events, Serious Adverse Events, and Unanticipated Problems Resulting in Risk to Subjects or Others

All adverse events, serious adverse events, and unanticipated problems resulting in risk to subjects or others are to be reported to the Methods Center immediately.

8.2.1 Investigator Reporting: Notifying the Methods Center

Any SAEs must be reported to the Methods Center by completing the Adverse Events Form and indicating that the adverse event was serious, then submitting it to iDataFax. The investigator will keep a copy of this form on file at the study site. Significant new information on ongoing serious adverse events should be provided promptly to the Methods Center by updating the AE form.

Unanticipated problems resulting in risk to subjects or others are to be reported to the Methods Center by either fax or email.

8.2.2 Site Investigator ─ IRB/REB Reporting

Investigators are responsible for reporting AEs, SAEs, and unanticipated problems resulting in risk to subjects or others to their local IRB/REB. Investigators are responsible for complying with their local IRB’s/REB’s reporting requirements. Copies of each report and documentation of IRB/REB notification and receipt will be kept in the investigator’s study file.

8.2.3 Data Safety Monitoring Board (DSMB)

The DSMB will monitor the trial, review quarterly quality control and safety reports, and meet annually.[25, 26] The Committee members will be independent of the trial, free of conflicts with any of the investigative team and will consist of a clinical trial methodologist, a statistician and Orthopaedic Surgeons. The terms of reference and functions are derived from the principles established by the Data and Safety Monitoring Boards: Lessons, Ethics, Statistics (DAMOCLES) Study Group charter.

9 Data Handling and Record Keeping

9.1 Confidentiality

Information about study subjects will be kept confidential and managed according to the requirements of the Health Insurance Portability and Accountability Act of 1996 (HIPAA). Those regulations require a signed subject authorization informing the subject of the following:

- What protected health information (PHI) will be collected from subjects in this study;
- Who will have access to that information and why;
- Who will use or disclose that information; and
- The rights of a research subject to revoke their authorization for use of their PHI.

In the event that a subject revokes authorization to collect or use PHI, the investigator, by regulation, retains the ability to use all information collected prior to the revocation of subject authorization. For subjects that have revoked authorization to collect or use PHI, attempts should be made to obtain permission to collect at least vital status (i.e. that the subject is alive) at the end of their scheduled study period.

9.2 Case Report Forms

The CRFs are the primary data collection instrument for the study. All data requested on the CRF must be recorded. All missing data must be explained. If a space on the CRF is left blank because the procedure was not done or the question was not asked, write “N/D”. If the item is not applicable to the individual case, write “N/A”. Sites will receive an iDataFax Manual which includes detailed instructions for entering data using iDataFax.

10 Ethical Considerations

This study is to be conducted according to US and international standards of Good Clinical Practice (FDA Title 21 part 312 and International Conference on Harmonization

guidelines), applicable government regulations, and institutional research policies and procedures.

This protocol and any amendments will be submitted to a properly constituted independent REB or IRB, in agreement with local legal prescriptions, for formal approval of the study conduct. The decision of the REB /IRB concerning the conduct of the study will be made in writing to the investigator and a copy of this decision will be provided to the Methods Center before commencement of this study.

All subjects for this study will be provided a consent form describing this study and providing sufficient information for subjects to make an informed decision about their participation in this study. The consent form will be submitted with the protocol for review and approval by the REB /IRB for the study. The formal consent of a subject, using the REB /IRB-approved consent form, must be obtained before that subject undergoes any study procedure. The consent form must be signed by the subject or legally authorized representative, and the investigator-designated research professional obtaining the consent.

11 References

1. Jeys L, Grimer R The long-term risks of infection and amputation with limb salvage surgery using endoprostheses. *Recent Results Cancer Res* 2009;179:75-84.:75-84.

2. Morii T, Yabe H, Morioka H, Beppu Y, Chuman H, Kawai A, Takeda K, Kikuta K, Hosaka S, Yazawa Y, Takeuchi K, Anazawa U, Mochizuki K, Satomi K Postoperative deep infection in tumor endoprosthesis reconstruction around the knee. *J Orthop Sci* 2010;15:331-9.

3. Grimer RJ, Belthur M, Chandrasekar C, Carter SR, Tillman RM Two-stage revision for infected endoprostheses used in tumor surgery. *Clin Orthop Relat Res* 2002:193-203.

4. Jeys LM, Grimer RJ, Carter SR, Tillman RM Risk of amputation following limb salvage surgery with endoprosthetic replacement, in a consecutive series of 1261 patients. *Int Orthop* 2003;27:160-3.

5. Wirganowicz PZ, Eckardt JJ, Dorey FJ, Eilber FR, Kabo JM Etiology and results of tumor endoprosthesis revision surgery in 64 patients. *Clin Orthop Relat Res* 1999:64-74.

6. Akahane T, Shimizu T, Isobe K, Yoshimura Y, Fujioka F, Kato H Evaluation of postoperative general quality of life for patients with osteosarcoma around the knee joint. *J Pediatr Orthop B* 2007;16:269-72.

7. Abudu A, Carter SR, Grimer RJ The outcome and functional results of diaphyseal endoprostheses after tumour excision. *J Bone Joint Surg* 1996; 78B:652-657.

8. Abudu A, Grimer RJ, Tillman RM, Carter SR Endoprosthetic replacement of the distal tibia and ankle joint for aggressive bone tumours. *Int Orthop* 1999;23:291-294.

9. Grimer RJ, Carter SR, Tillman RM, Sneath RS, Walker PS, Unwin PS, Shewell PC Endoprosthetic replacement of the proximal tibia. *J Bone Jointt Surg* 1999;81B:488-494.

10. Hasan K, Racano A, Deheshi B, Farrokhyar F, Wunder J, Ferguson P, Holt G, Schwartz H, Petrisor B, Bhandari M, Ghert M Prophylactic antibiotic regimens in tumor surgery (PARITY) survey. 2011. *Manuscript submitted for publication*.

11. Bratzler DW, Houck PM Antimicrobial prophylaxis for surgery: an advisory statement from the National Surgical Infection Prevention Project. *Clin Infect Dis* 2004;38:1706-15.

12. Zhanel GG, Adam HJ, Low DE, Blondeau J, DeCorby M, Karlowsky JA, Weshnoweski B, Vashisht R, Wierzbowski A, Hoban DJ Antimicrobial susceptibility of 15,644 pathogens from Canadian hospitals: results of the CANWARD 2007-2009 study. *Diagn Microbiol Infect Dis* 2011;69:291-306.

13. Costelloe C, Metcalfe C, Lovering A, Mant D, Hay AD Effect of antibiotic prescribing in primary care on antimicrobial resistance in individual patients: systematic review and meta-analysis. *BMJ* 2010;340:c2096. doi: 10.1136/bmj.c2096.:c2096.

14. Bergogne-Berezin E Treatment and prevention of antibiotic associated diarrhea. *Int J Antimicrob Agents* 2000;16:521-6.

15. Weiss K, Bergeron L, Bernatchez H, Goyette M, Savoie M, Thirion D Clostridium difficile-associated diarrhoea rates and global antibiotic consumption in five Quebec institutions from 2001 to 2004. *Int J Antimicrob Agents* 2007;30:309-14.

16. Shigemura K, Tanaka K, Adachi M, Yamashita M, Arakawa S, Fujisawa M Chronological change of antibiotic use and antibiotic resistance in Escherichia coli causing urinary tract infections. *J Infect Chemother* 2011.

17. Hueston WJ, Dickerson L Antibiotic resistance and the need for the rational use of antibiotics. *J Med Liban* 2001;49:246-56.

18. Christiaens TC, Digranes A, Baerheim A The relation between sale of antimicrobial drugs and antibiotic resistance in uropathogens in general practice. *Scand J Prim Health Care* 2002;20:45-9.

19. Horan TC, Gaynes RP, Martone WJ, Jarvis WR, Emori TG CDC definitions of nosocomial surgical site infections, 1992: a modification of CDC definitions of surgical wound infections. *Am J Infect Control* 1992;20:271-274.

20. Davis AM, Wright JG, Williams JI, Bombardier C, Griffin A, Bell RS Development of a measure of physical function for patients with bone and soft tissue sarcoma. *Qual Life Res* 1996;5:508-16.

21. Schreiber D, Bell RS, Wunder JS, O'Sullivan B, Turcotte R, Masri BA, Davis AM Evaluating function and health related quality of life in patients treated for extremity soft tissue sarcoma. *Qual Life Res* 2006;15:1439-46.

22. Tunn PU, Pomraenke D, Goerling U, Hohenberger P Functional outcome after endoprosthetic limb-salvage therapy of primary bone tumours--a comparative analysis using the MSTS score, the TESS and the RNL index. *Int Orthop* 2008;32:619-25.

23. Sprague S, Leece P, Bhandari M, Tornetta P, Schemitsch E, Swiontkowski M. Limiting loss to follow-up in a multicenter randomized trial in orthopaedic surgery. *Control Clin Trials* 2003; 24: 719-725.

24. Assmann SF, Pocock SJ, Enos LE, Kasten LE Subgroup analysis and other (mis)uses of baseline data in clinical trials. *Lancet* 2000;355:1064-9.

25. Friedman L The NHLBI model: a 25 year history. *Stat Med* 1993;12:425-31.

26. Slutsky AS, Lavery JV Data safety and monitoring boards. *N Engl J Med* 2004;350:1143-7.

Protocol Summary of Changes

During the conduct of the **P**rophylactic **A**ntibiotic **R**egimens **I**n **T**umor Surger**y** (PARITY) trial, we undertook five amendments to the trial protocol. The table below provides the version numbers and dates, as well as summarizes the corresponding major changes in each amendment.

Version No.	Version Date	Major Changes
1.0	December 20, 2011	Initial Version
2.0	May 16, 2012	▪ Patients administered study questionnaires at baseline. *We previously failed to state this explicitly in the protocol.*<br>▪ Patients no longer administered study questionnaires at 2W and 4W follow-up visits. *We concluded that this would be too burdensome for patients; moreover, quality of life and functional data at these follow-up times would not be meaningful as patients are recovering from surgery and, thus, quite limited.*<br>▪ Inclusion age changed from 16 years of age to 15 years of age. *From a skeletal standpoint, 15-year-old patients are 'mature' and considered 'adults'; therefore, these patients should be included as well.*
2.1	November 20, 2012	▪ Study changed to a superiority design, which resulted in a change in the sample size calculation (435 patients per arm). *After discussion with the PARITY Steering Committee (including the study statistician), it was decided that the study be re-framed as a superiority trial (i.e., five days of post-operative antibiotics is superior to 24 hours of post-operative antibiotics).*<br>▪ Unplanned re-operations rate added as a secondary outcome to be captured as a likely corollary to infection (i.e., the study's primary outcome measure).<br>▪ Randomization changed to only be stratified based on clinical site and location of tumor. *Experts in the field agreed that peri-operative chemotherapy is inconsistent among patients and, thus, not suitable as a stratification criterion.*<br>▪ Patients no longer administered study questionnaires at 9M follow-up visits. *We concluded that this would be too burdensome for patients and provide little additional data when compared to the questionnaires from the 6M and 12M follow-up visits.*<br>▪ Follow-up schedule changed from 2W, **4W**, 3M, 6M, 9M, 12M to 2W, **6W**, 3M, 6M, 9M, 12M. *We concluded that this was better reflective of the current standard practice for this patient population.*<br>▪ Addition of the Musculoskeletal Tumor Society (MSTS)-87 questionnaire to be administered at the same follow-up visits as the other study questionnaires. *We concluded that this questionnaire is also relevant and should be used in addition to the MSTS-93 and Toronto Extremity Salvage Score (TESS) questionnaires.*<br>▪ Randomization was clarified to occur in random block sizes of 2 or 4 based on tumor location. *The femur (block size of 4) is a more common tumor site than the tibia (block size of 2).*

		▪ The pre-operative antibiotic regimen was clarified to state that all patients will receive the pre-operative antibiotic dose within 60 minutes of surgery.<br>▪ The post-operative antibiotic regimens were clarified for patients discharged prior to five days post-surgery. Those allocated to the short arm will continue to receive saline and those allocated to the long arm will continue to receive study antibiotics until discharge. *We concluded that a discharge from hospital prior to five days post-surgery is not likely given the complexity of this type of surgery.*<br>▪ The study unblinding procedures were stipulated on a per-patient basis.<br>▪ Inclusion of individuals who have not reached skeletal maturity. *We concluded that this term 'skeletally mature' is too restrictive and may exclude patients who, though skeletally immature, were still appropriate candidates for this type of surgery and, therefore, this study.*<br>▪ Removal of the redundant inclusion criterion stipulating non-compulsory pre-operative chemotherapy.<br>▪ Clarification of the exclusion criterion regarding allergy to either penicillin or cefazolin. *We concluded that a 'known allergy' should be clarified as 'documented anaphylaxis or angioedema'.*<br>▪ Addition of exclusion criterion for patients with renal insufficiencies (as evidenced by an estimated creatinine clearance (eGRF) of less than 54 mL/min). *Based on the recommendation of an Infectious Diseases specialist, we concluded that these patients should be excluded as they may experience problems with proper drug clearance.*
2.2	February 28, 2013	▪ All references to the trade name 'Ancef' were replaced with the generic medication name 'cefazolin'. *We concluded that the use of the generic name better reflects the international community participating in the study.*<br>▪ Addition of the statement 'or equivalent gram-positive coverage (i.e., cefuroxime)' after every mention of cefazolin as the study antibiotic. *We concluded that the study drug would be revised to include the use of an antibiotic that provides equivalent gram-positive coverage (i.e., cefuroxime) to better reflect the international community looking to participate that may not have access to / approval for cefazolin.*<br>▪ Addition of the statement 'The same antibiotics must be used for ALL patients at each site'. *This sentence was added to ensure standardized procedures across all participating clinical sites.*
3.0	April 25, 2013	▪ Clarification of the sample size calculation, which included the addition of the statement 'The goal is to detect an absolute difference of 5% between our two treatment arms' in order to clarify the absolute difference aimed to be achieved through the study.<br>▪ Sample size calculation error corrected from **960** patients (460 patients per arm) to **920** patients (460 patients per arm).<br>▪ Revision of the statistical plan, which included the removal of the statement 'We have set an upper threshold

		(i.e., margin of superiority) of an absolute difference of 5% to define superiority: up to a 5% higher infection rate with long-term antibiotics will be considered superior to short-term antibiotics.' *The identification of the upper threshold is no longer necessary for the revised statistical plan.*<br>▪ Revision of the statistical methods for the primary outcome.<br>▪ Revision of the statistical plan for the secondary analyses.<br>▪ Addition of patients with soft-tissue sarcomas of the lower extremities which have invaded the bone and require bone resection and endoprosthetic reconstruction of the tibia or femur.
4.0 / 4.1	January 8, 2014	▪ Clarification of the pre-, intra- and post-operative antibiotic regimens to indicate that no antibiotics other than the study antibiotics will be administered.<br>▪ Removal of the statement 'or equivalent gram-positive coverage (i.e., cefuroxime)'. *We concluded that it would be better to split the protocol into two separate current protocols (V4.0 and V4.1) that represent the two possible study antibiotics treatments (cefazolin and cefuroxime, respectively).*
5.0 / 5.1	May 13, 2014	▪ Expansion of the primary outcome from 'deep surgical site infection' to 'surgical site infection'. *We concluded that this change would increase the expected event rate (and study power) without compromising clinical importance.*<br>▪ Revision of the primary outcome measures to include the diagnostic criteria for any surgical site infection (superficial, deep or organ space) in order to reflect the expansion of the primary outcome.<br>▪ Revision of the sample size estimation (300 patients per arm for a total of 600 patients) based on pilot data (pilot event rate of 14%) and the expansion of the primary outcome.<br>▪ Addition of weight-based pediatric doses for the pre-, intra- and post-operative antibiotic regimens to coincide with the reduction of the minimum inclusion age.<br>▪ Addition of the possible risks associated with the study drug. *This addition was requested by Health Canada.*<br>▪ Clarification of the study events that were to be reviewed by the study Adjudication Committee.<br>▪ Revision of the statistical methods for both the primary and secondary analyses.<br>▪ Clarification that there will be no interim analysis given the risk for systematically overestimating treatment effects.<br>▪ Clarification of events that are expected and, therefore, should not be considered adverse events.<br>▪ Clarification of the process for the reporting of serious adverse events.<br>▪ Inclusion age changed from 15 years of age to 12 years of age.
6.0 / 6.1	October 31, 2016	▪ Clarification of the term 'lower extremity' (i.e., femur or tibia).

		▪ Clarification of eligibility for patients who had undergone a prior revision surgery.<br>▪ Addition of patients with oligometastatic bone disease.

The initial protocol (Version 1.0), and all subsequent amendments, was submitted and approved by the Hamilton Integrated Research Ethics Board. The protocol, and all subsequent amendments, was also submitted to Health Canada for review, who had no objections. At participating clinical sites, all necessary regulatory and ethical bodies reviewed and approved the study protocol and its amendments prior to local study initiation.

Statistical Analysis Plan Version 1.0

(Follows)

Statistical Analysis Plan

Version 1.0

Prophylactic **A**ntibiotic **R**egimens **i**n **T**umor Surger**y (PARITY)**:

A Multi-Center Randomized Controlled Study Comparing Alternative Antibiotic Regimens in Patients Undergoing Tumor Resections with Endoprosthetic Replacements

Principal Investigator: Dr. Michelle Ghert

This study is registered on ClinicalTrials.gov
Identification No.: NCT01479283

Document History

Date (DD-MMM-YY)	Drafted / Revised By:	Version No.	Description of Amendments
11-JAN-21	Tricia Schneider, Diane Heels-Ansdell, Lehana Thabane and Michelle Ghert	1.0	Initial version

Purpose

The purpose of this Statistical Analysis Plan (SAP) is to outline the primary statistical analyses for the primary **P**rophylactic **A**ntibiotic **R**egimens **I**n **T**umor Surger**y** (PARITY) trial manuscript. This document includes a review of all data collected, and follows the Journal of the American Medical Association (JAMA) Guidelines for the content of statistical analysis plans in clinical trials[1]. The PARITY Writing Committee will determine which data points will be included in the primary manuscript and supplemental documents. We will adhere to the Consolidated Standards of Reporting Trials (CONSORT) 2010 guideline when reporting the results of the PARITY trial[2]. Additional SAPs will be developed for secondary analyses.

Study Summary

Title	Prophylactic Antibiotic Regimens in Tumor Surgery (PARITY): A Multi-Center Randomized Controlled Study Comparing Alternative Antibiotics Regimens in Patients Undergoing Tumor Resections with Endoprosthetic Replacements
Short Title	PARITY
Methodology	Multi-Center, Blinded, Randomized Trial
Study Duration	December 2012 to March 2021
Study Center(s)	Multi-Center
Primary Study Question	In patients undergoing surgical excision and endoprosthetic reconstruction of the femur or tibia for a tumor, is a long-duration (five days) post-operative antibiotic regimen more effective at decreasing the rate of infection when compared to a short-duration (24 hours) post-operative antibiotic regimen?
Diagnosis and Main Inclusion Criteria	Primary malignant or benign aggressive bone tumors of the femur or tibia, soft-tissue sarcomas of the lower extremity which have invaded the femur or tibia, or oligometastatic bone disease of the femur or tibia that requires surgical excision and endoprosthetic reconstruction.
Hypothesis	We hypothesize that the long-duration (five days) post-operative antibiotic regimen will result in lower rates of surgical site infections.
Sample Size	600
Study Product, Dose, Route, Regimen	Intravenous cephalosporin antibiotic (cefazolin or cefuroxime) for 24 hours or five days.
Length of Follow-Up	1 year

Study Introduction

Limb salvage surgery is the standard of care in the management of sarcoma of the long bones[3–5]. Advances in chemotherapeutic regimens and imaging techniques allow for wide resection and functional reconstruction in 95% of patients. The most common type of long-bone reconstruction involves the use of a tumor prosthesis, or endoprostheses. Due to the complexity and length of surgical resection and reconstruction, as well as the immunocompromised nature of patients treated with chemotherapy, the risk for infection remains high[6,7]. Deep infection following endoprosthetic

reconstruction is a devastating complication that requires staged revision surgery and long-term intravenous antibiotics. The risk for subsequent infection remains high, as does the risk for ultimate amputation[6,7]. However, the most effective antibiotic regimen in preventing post-operative deep infections remains controversial, and the current state of practice varies widely, particularly with respect to antibiotic duration[8]. Moreover, patients' quality-of-life and function following infection are dramatically impacted, as are health care costs[9,10]. Strategies to optimize prevention of infection and quality-of-life, while mitigating health care costs are needed.

The **P**rophylactic **A**ntibiotic **R**egimens **i**n **T**umor Surger**y** (PARITY) trial is an ongoing international, multi-center, randomized controlled trial using a parallel two-arm design[11]. Six-hundred participants 12 years of age or older undergoing surgical excision and endoprosthetic reconstruction of a lower extremity primary bone tumor across North America, South America, Europe, Australia, Africa and Asia will be randomized to receive either short (24 hours) or long (five days) duration post-operative antibiotics. Allocation is concealed using a centralized and automated 24-hour computerized randomization platform that allows for internet-based randomization. Randomization is stratified by tumor location (i.e., femur or tibia) and clinical site in randomly permuted blocks of two and four. The primary outcome of the study is the development of a surgical site infection (SSI), guided by the Centers for Disease Control and Prevention's (CDC) National Healthcare Safety Network reporting criteria[12]. Secondary outcomes include the development of antibiotic-related complications (i.e., gastrointestinal infections, fungal infections, etc.), unplanned re-operations, oncologic outcomes, mortality, and patient functional outcomes and quality-of-life at one year. Participants are regularly monitored post-operatively by the treating surgeon at the two-week, six-week, three-month, six-month, nine-month and one-year follow-up visits. SSIs, antibiotic-related complications, re-operations and mortality will be reviewed by an Adjudication Committee. Data analysts and Adjudication Committee members are blinded to treatment allocation.

Primary Endpoint

The primary study endpoint is the development of a SSI following the initial surgery to treat a tumor of the femur or tibia. SSIs were classified according to the criteria established by the CDC, which defines a SSI as an infection occurring within the 30 days following the operative procedure or within one year if an implant is in place and the infection appears to be related to the operative procedure[12]. The SSI can involve any part of the body, but excludes the skin incision, fascia, or muscle layers, that is opened or manipulated during the operative procedure. The participant must also present with at least one of the following:

- purulent drainage from the superficial / deep / organ space incision;
- organisms isolated from an aseptically obtained culture of fluid or tissue from the superficial / deep / organ space incision;
- superficial / deep / organ space incision that is deliberately opened by a surgeon, attending physician or other designee and is culture positive or not cultured *and* the participant has at least one of the following signs or symptoms: pain or tenderness; localized swelling; redness; or heat; or
- diagnosis of a superficial / deep / organ space incisional SSI by a surgeon or attending physician.

Secondary Endpoints

The secondary endpoints include participants' functional outcome and quality-of-life, rate of re-operation, antibiotic-related complications, oncologic recurrence and / or metastases, and all-cause mortality. Questionnaires, namely the Musculoskeletal Tumor Society (MSTS) functional scores (1987 and 1993 versions) and the Toronto Extremity Salvage Score (TESS), will be used to assess both functional outcome and quality-of-life prior to surgery, as well as at the three-month, six-month, and one-year follow-up visits. The MSTS-87, MSTS-93 and TESS surveys are commonly accepted functional scoring systems in orthopaedic oncology literature[13–15].

Analysis Plan

Overview

All outcome analyses will be performed using the intention-to-treat (ITT) approach. The primary analysis will compare the treatment groups on the SSI outcome and the secondary analysis will compare the treatment groups on the following outcomes at follow-up: antibiotic-related complications, unplanned re-operations, oncologic outcomes, all-cause mortality and patient functional outcomes and quality-of-life. The secondary comparison will be conducted in accordance with best practice guidelines for secondary analyses. For all models, the results will be expressed as hazards ratios [HRs] for time-to-event outcomes and mean difference for continuous outcomes, with corresponding two-sided 95% confidence intervals and associated p-values. All statistical tests will be performed using two-sided tests at the 0.05 level of significance. Analyses of secondary outcomes are exploratory in nature and, therefore, alpha values will not be adjusted for multiple testing. P-values will be reported to three decimal places with values less than 0.001 reported as < 0.001. All analyses will be performed using SAS 9.4 (Cary, North Carolina, USA).

Blinded Analyses

The primary analyses for the primary and secondary endpoints will first be completed using only blinded treatment groups (i.e., antibiotic duration X and Y). Interpretations for the effect of antibiotic duration will be documented during a blinded review of the data based upon blinded X versus Y post-operative antibiotic duration[16]. We will unblind the results by breaking the randomization code following the documentation of the interpretations. These agreed upon interpretations will guide the discussion section of the subsequent definitive trial manuscript.

Presentation of Data

Screening and Enrolment

The number of patients screened, included and excluded will be presented in a flow diagram (**Figure 1**). The figure will include the number of patients who were eligible, ineligible and randomly assigned to the two treatment groups. It will also include the number of participants who were lost-to-follow-up over the course of the study. The number of patients excluded by reason will also be summarized in the flow diagram (**Figure 1**).

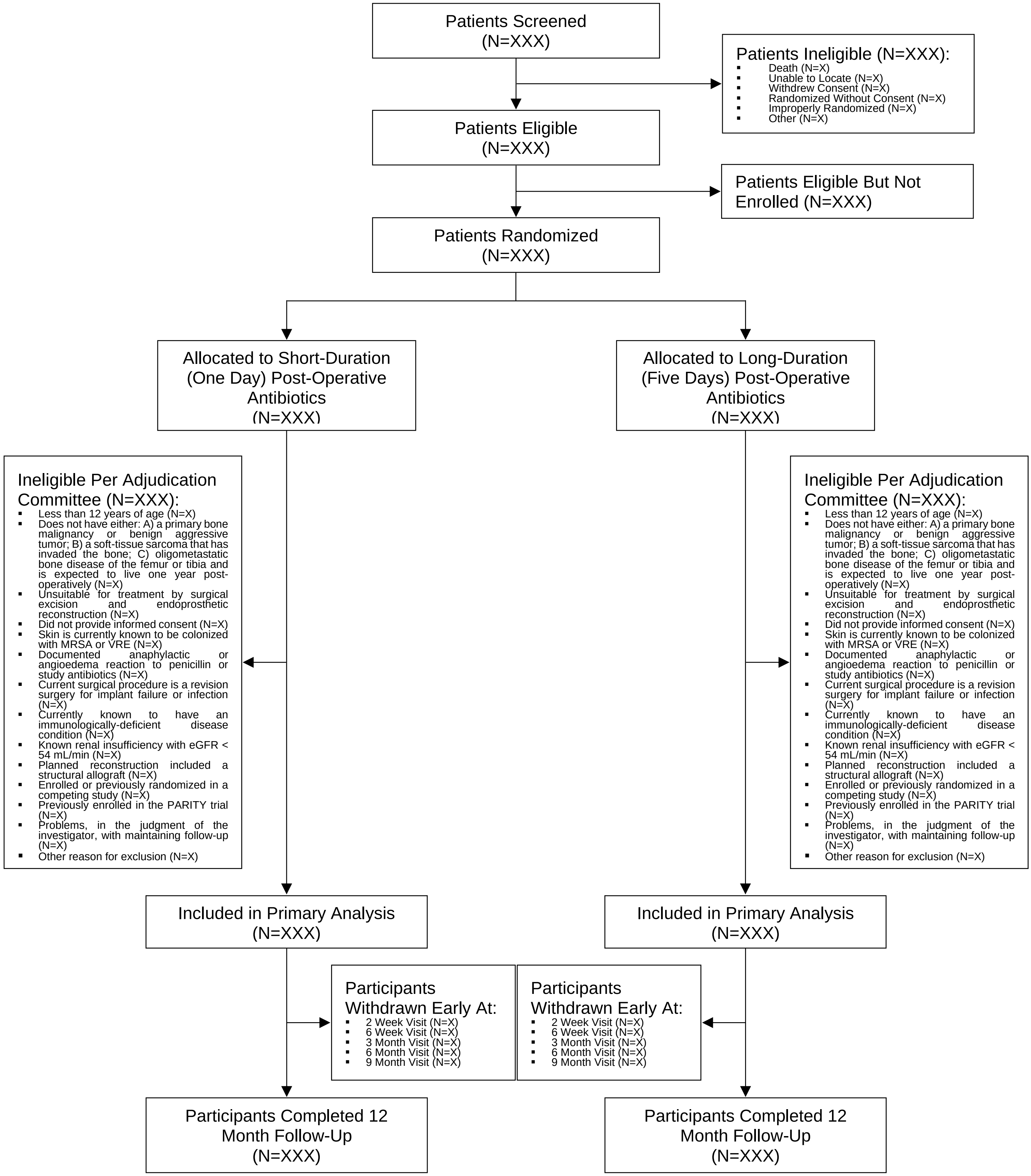

Figure 1: Screening and Enrolment Flow Diagram

Participant Demographics and Baseline Characteristics

Participant demographics and baseline characteristics will be presented by each treatment group (**Table 1**). Continuous data will be presented with means and standard deviations (SDs), or medians and first and third quartiles (Q1, Q3) for skewed data, and categorical data will be presented as frequencies and proportions.

Table 1: Participant Demographics and Baseline Details

Characteristic	Relevant Variable (Case Report Form & Question No.)	Treatment X N = XXX	Treatment Y N = XXX
**Age**, mean (SD)<br>[years]	Date of Birth (Form 2.1, Q2)<br>Date of Surgery (Form 2.1, Q4)		
**Gender**, n (%)<br>Male<br>Female	Form 4.1, Q2		
**Ethnicity**, n (%)<br>White/Caucasian<br>Black<br>Native<br>Asian<br>Hispanic<br>Other (Specify)	Form 4.1, Q3		
**Pre-Diagnosis Employment**, n (%)<br>Employed<br>Not Employed<br>Retired<br>Student<br>Homemaker<br>Doctor's Advice/Disability<br>Unemployed	Form 4.2, Q13		
**Other Known Malignancies at Baseline**, n (%)<br>No<br>Yes	Form 4.1, Q6		
**Systemic Metastases at Baseline**, n (%)<br>No<br>Yes<br>Pulmonary<br>Skeletal<br>Other Viscera (Specify)<br>Other (Specify)	Form 4.1, Q7		
**Other Cancer Treatment Modalities at Baseline**, n (%)<br>No<br>Yes<br>Pre-Operative Chemotherapy<br>Pre-Operative Radiation<br>Other (Specify)	Form 4.3, Q15		
**Smoking Status**, n (%)<br>Current Smoker<br>Former Smoker<br>Non-Smoker	Form 4.2, Q10		
**Alcohol Consumption**, n (%)<br>No	Form 4.2, Q11		

Yes			
**Recreational IV Drug Use**, n (%)<br>No<br>Yes (Specify)	Form 4.2, Q12		
**Diabetes Status**, n (%)<br>No<br>Yes<br>Insulin Dependent (Type I)<br>Non-Insulin Dependent (Type II)	Form 4.2, Q9		
**Medication Use at Baseline**, n (%)<br>None<br>NSAIDS<br>Analgesics: Opioids<br>Anti-Hypertension Medications<br>General Cardiac Medications<br>Pulmonary Medications<br>Osteoporosis Medications<br>Antibiotics	Form 4.3, Q14		

Tumor Characteristics

Participant tumor characteristics will be presented by each treatment group (**Table 2**). Tumor characteristics will be presented as frequencies and proportions.

Table 2: Tumor Details

Characteristic	Relevant Variable (Case Report Form & Question No.)	Treatment X N = XXX	Treatment Y N = XXX
**Location of Tumor**, n (%)<br>Femur<br>Tibia	Form 2.1, Q3		
**Location in Bone**, n (%)<br>Proximal<br>Mid-Shaft<br>Distal<br>Other (Specify)	Form 4.1, Q5		
**Maximum Size**, mean (SD) [centimeters]	Form 4.1, Q6		
**No. of Compartments**, n (%)<br>0<br>1<br>2<br>3<br>4	Form 4.1, Q8		
**Type of Biopsy Performed**, n (%)<br>None<br>Open<br>Fine Needle Aspiration<br>Core Needle	Form 5.1, Q2		
**Type of Tumor**, n (%)<br>Bone Sarcoma<br>Soft-Tissue Sarcoma<br>Metastatic Bone Disease	Form 7.1, Q3		
**Overall Margins**, n (%)<br>Negative<br>Microscopically Positive	Form 7.1, Q7		

Grossly Positive			

Surgical and Peri-Operative Management Details

Participant surgical details will be presented by each treatment group (**Table 3**). Continuous data will be presented with means and standard deviations (SDs), or medians with (Q1, Q3) if data are skewed, and categorical data will be presented as frequencies and proportions.

Table 3: Surgical and Peri-Operative Management Details

Characteristic	Relevant Variable (Case Report Form & Question No.)	Treatment X N = XXX	Treatment Y N = XXX
Surgical Details			
**Length of Procedure**, mean (SD) [minutes]	Form 6.1, Q7		
**Type of Skin Sterilization**, n (%)<br>Iodine<br>Alcohol<br>Chlorohexidine	Form 6.1, Q8		
**Length of Incision**, mean (SD) [centimeters]	Form 6.1, Q9		
**Laminar Flow**, n (%)<br>No<br>Yes	Form 6.1, Q10		
**Spacesuit Worn**, n (%)<br>No<br>Yes	Form 6.1, Q11		
**Tourniquet Used**, n (%)<br>No<br>Yes	Form 6.3, Q23		
**Type of Resection**, n (%)<br>Intra-Articular<br>Extra-Articular	Form 6.3, Q22		
**Length of Bone Resected**, n (%)<br>< 5 cm<br>5-10 cm<br>> 10 cm	Form 6.3, Q25		
**Skin Excised**, n (%)<br>None<br>Small (< 5 cm^2)<br>Moderate (5-10 cm^2)<br>Large (> 10 cm^2)	Form 6.2, Q16		
**Muscle Excised**, n (%)<br>None<br>Small (< 50 cm^3)<br>Moderate (50-100 cm^3)<br>Large (> 100 cm^3)	Form 6.2, Q17		
**Fascial Tissue Excised**, n (%)<br>None<br>Small (< 1 cm^2)<br>Moderate (1-5 cm^2)<br>Large (> 5 cm^2)	Form 6.2, Q18		
**Type of Fixation**, n (%)<br>Press-Fit<br>Cement<br>With Antibiotic (Specify)	Form 6.2, Q14		

Without Antibiotic<br>Cerclage<br>Wire<br>Cable<br>Synthetic			
**Bone Grafting Performed**, n (%)<br>No<br>Yes<br>Synthetic Bone Graft<br>Autograft<br>Cortical<br>Cancellous<br>Vascularized Cancellous<br>Allograft<br>Cortical<br>Cancellous	Form 6.2, Q15		
**Vascular Reconstruction**, n (%)<br>No<br>Yes<br>< 5 cm<br>5-10 cm<br>> 10 cm	Form 6.3, Q26		
**Intra-Operative Thromboprophylaxis**, n (%)<br>No<br>Yes<br>IV Heparin<br>Tranexamic Acid<br>Other (Specify)	Form 6.2, Q19		
**Antibiotic or Silver-Coated Prosthesis**, n (%)<br>No<br>Yes<br>Antibiotic (Specify)<br>Silver	Form 6.2, Q13		
**Antibiotic Impregnated Sponge or Antibiotic Powder Implanted**, n (%)<br>No<br>Yes<br>Gentamicin<br>Tobramycin<br>Cefazolin<br>Vancomycin<br>Other (Specify)	Form 6.2, Q20		
**Irrigation Performed at End of Procedure**, n (%)<br>No<br>Yes, Pulsed Irrigation<br>Yes, Antibiotics in Irrigation	Form 6.3, Q22		
**Mode of Skin Closure**, n (%)<br>Primary Closure<br>Local Fasciocutaneous Flap<br>Local Muscle Flap and Split Thickness Skin Graft<br>Free Flap	Form 6.3, Q27		
Peri-Operative Management Details			

**Post-Operative Thromboprophylaxis**, n (%)<br>No<br>Yes<br>Coumadin<br>Heparin<br>Fractionated Heparin<br>Oral	Form 8.1, Section A, Q3		
**Suction Drain**, n (%)<br>No<br>Yes<br>Duration	Form 8.1, Section A, Q4		
**Urinary Catheter**, n (%)<br>No<br>Yes<br>Duration	Form 8.1, Section A, Q5		
**No. of Patients in Hospital Room**, n (%)<br>1<br>2<br>3<br>4<br>> 4	Form 8.1, Section A, Q7		
**Time to First Post-Operative Wound Dressing Change**, mean (SD)<br>[days]	Date of Surgery (Form 6.1, Q4)<br>Date of First Post-Operative Dressing Change (Form 8.1, Section A, Q8)		
**Negative-Pressure Wound Therapy (Wound Vac)**, n (%)<br>No<br>Yes<br>Duration	Form 8.3, Section C, Q1		
**Length of Post-Operative Hospital Stay**, mean (SD)<br>[days]	Date of Surgery (Form 6.1, Q4)<br>Date of Discharge (Form 8.2, Section B, Q1)		
**Discharge Location**, n (%)<br>Home<br>Rehabilitation Facility<br>Other Hospital<br>Other (Specify)	Form 8.2, Section B, Q2		

Prophylactic Antibiotic Administration

Participant prophylactic antibiotic administration details will be presented by each treatment group (**Table 4**). Data will be presented as frequencies and proportions.

Table 4: Prophylactic Antibiotic Administration Details

Characteristic	Relevant Variable (Case Report Form & Question No.)	Treatment X<br>N = XXX	Treatment Y<br>N = XXX
**Pre-Operative Study Antibiotic Administered Per Protocol**, n (%)<br>No	Form 6.1, Q2		

Yes			
**Additional Pre-Operative Prophylactic Antibiotic(s) Administered**, n (%)<br>No<br>Yes	Form 6.1, Q3		
**Intra-Operative Study Antibiotic Administered Per Protocol**, n (%)<br>No<br>Yes	Form 8.1, Section A, Q1		
**Additional Intra-Operative Prophylactic Antibiotic(s) Administered**, n (%)<br>No<br>Yes	Form 8.1, Section A, Q2		
**Post-Operative Study Antibiotic/Placebo Administered Per Protocol**, n (%)<br>No<br>Yes	Form 8.1, Section A, Q12		
**Additional Post-Operative Prophylactic Antibiotic(s) Administered**, n (%)<br>No<br>Yes	Form 8.1, Section A, Q13		

Primary Outcome Analysis

The primary analysis will be a Cox proportional hazards analysis with time from surgery to the SSI as the primary outcome. Post-operative prophylactic antibiotic duration (treatment group [24 hours versus five days]) will be the independent variable, and the Cox regression will also include tumor location (femur or tibia) and clinical site as stratification variables. Participants who did not experience the primary endpoint will be censored at 12 months or the time of last visit.

The proportional hazards assumption of the Cox model will be assessed by examining Schoenfeld residuals. If an independent variable does not meet the assumption of proportional hazards, we will modify the model to allow the hazard ratio (HR) to differ throughout the study period guided by the observed data.

Results will be reported as HRs with the corresponding 95% confidence interval (CI) and associated p-values. Kaplan-Meier curves will be constructed for the two randomized treatment groups. For each treatment group, we will also report superficial SSI, deep SSI and organ space SSI. The results of the primary analysis will be presented in **Table 5**.

Table 5: Primary Outcome

Primary Endpoint	Relevant Variable (Case Report Form & Question No.)	Treatment X N = XXX n (%)	Treatment Y N = XXX n (%)	Hazard Ratio (95% CI)	P-Value
**Any Surgical Site Infection**	Adjudicated Data				
Superficial Incisional	Adjudicated Data				
Deep Incisional	Adjudicated Data				
Organ/Space	Adjudicated Data				

Sensitivity Analysis

We will conduct a competing risks analysis that accounts for death and amputation as competing risks. We will also perform sensitivity analyses for centre-effects where we will redo the primary analysis without including clinical site in the model. We will also look for prognostic imbalances between the two treatment groups based on the following key variables known to be risk factors for a SSI: total operative time, tumor location, diabetes status, chemotherapy regimen and radiation treatment. We will complete adjusted analyses to address any possible baseline imbalance between groups. Sensitivity analyses will be performed for the primary outcome only.

Sub-Group Analysis

At the onset of the PARITY trial, we identified two important sub-groups, which will be reported according to standard guidelines[17]. As we near the end of the trial, prior to unblinding, we have identified a further three important sub-groups (sex, age and peri-operative chemotherapy). We will add a main effect for the sub-group variable and the treatment by sub-group interaction to our primary model described above to assess whether the magnitude of the treatment effect is significantly different between sub-groups. This will be repeated separately for each sub-group variable. We will perform the following sub-group analyses with the primary endpoint as the outcome (**Table 6**):

- Tumor Type – the type of tumor will be classified as follows: bone sarcoma, soft-tissue sarcoma or oligometastatic bone disease. We hypothesize that there will be no difference in infection rates between the tumor types irrespective of prophylactic antibiotic duration.
- Tumor Location – the location of the tumor will be classified as follows: femur or tibia (we will not include the stratification variable of tumor location in this analysis). We hypothesize that a longer duration (five days) of antibiotics will be more effective relative to a shorter duration (24 hours) in tibial reconstructions than in femoral reconstructions.
- Sex – sex will be classified as follows: male or female. We hypothesize that there will be no difference between the sexes with regards to the association between prophylactic antibiotic duration and infection rates.
- Age – age will be classified as follows: pediatric and young adults (12 – 30 years of age) or older adults (≥31 years of age). We hypothesize that a longer duration (five days) of antibiotics will be more effective relative to a shorter duration (24 hours) in the older adult population than in the pediatric and young adult population.
- Peri-Operative Chemotherapy – peri-operative chemotherapy will be classified as follows: no chemotherapy versus chemotherapy (neoadjuvant or adjuvant or a combination of the two). We hypothesize that a longer duration (five days) of antibiotics will be more effective relative to a shorter duration (24 hours) in patients who received chemotherapy than in those who did not receive chemotherapy.

Table 6: Sub-Group Analyses Factors

Characteristic	Relevant Variable (Case Report Form & Question No.)	Treatment X		Treatment Y			
		N = XXX	n (%)	N = XXX	n (%)	Hazard Ratio (95% CI)	P-Value for the Interaction
**Tumor Type**<br>Bone Sarcoma	Form 7.1, Q3						

Soft-Tissue Sarcoma<br>Oligometastatic Bone Disease							
**Tumor Location**<br>Tibia<br>Femur	Form 2.1, Q3						
**Sex**<br>Male<br>Female	Form 4.1, Q2						
**Age**<br>12 – 30 Years<br>≥31 Years	Date of Birth (Form 2.1, Q2)<br>Date of Randomization (Form 2.1, Q4)						
**Peri-Operative Chemotherapy**<br>Yes<br>No	Neoadjuvant Chemotherapy (Form 4.3, Q15)<br>Adjuvant Chemotherapy (Form 9.3, Q19)						

Rather than pre-specifying a threshold p-value for making a sub-group claim, we will use the approach suggested by Sun et al to consider the plausibility of any possible sub-group effects[18]. If a plausible sub-group effect is found, we will further explore the impact of the sub-group on the secondary outcomes. However, due to an inadequate sample size and power to conduct the sub-group analyses, these results will be used solely for the generation of hypotheses for further investigations.

Interim Analysis

No interim analyses are planned due to our desire to avoid spuriously inflated estimates of treatment effects[19,20]. The PARITY Data and Safety Monitoring Board (DSMB) regularly meets to monitor the study data for participant safety.

Secondary Outcome Analyses

Functional and Quality-of-Life Outcomes

The Musculoskeletal Tumor Society 1987 (MSTS-87) score is a standardized scoring system that is completed by an individual on the treatment team and measures physical function after treatment for a musculoskeletal tumor. The lower extremity portion of the system assigns numerical values (0-5) for each of the following seven categories: motion, pain, stability, deformity, muscular strength, functional activity and emotional status. A numerical score and percent rating are calculated to allow for the comparison of results. The score is summed out of a maximum of 35 and a higher score is associated with better physical function.

The Musculoskeletal Tumor Society 1993 (MSTS-93) score is a standardized scoring system that is completed by an individual on the treatment team (preferably the orthopaedic oncologist) and measures functional outcome after treatment for a musculoskeletal tumor. The lower extremity portion of the system assigns numerical values (0-5) for each of the following six categories: pain, function, emotional acceptance, support, walking ability and gait. A numerical score and percent rating are calculated to

allow for the comparison of results. The score is summed out of a maximum of 25 and a higher score is associated with better physical function.

The Toronto Extremity Salvage Score (TESS) survey is a validated, patient-reported evaluation tool that was developed to assess physical function and quality-of-life in patients that have undergone limb salvage surgery for tumors of the extremities. The lower extremity portion of the survey contains 30 questions that are framed to ask about the difficulty experienced by the patient in performing each of the activities over the previous week. Difficulty is rated on a five-point Likert scale ranging from ‘not at all difficult’ to ‘impossible to do’. A numerical score is calculated to allow for the comparison of results. The score is summed out of a maximum of 150 and a higher score is associated with better functional outcomes and quality-of-life.

The MSTS-87, MSTS-93 and TESS surveys are completed at the one-year follow-up visit. We will only include scores in our analyses if the questionnaires are completed within an acceptable timeframe based on the designated visit due to concerns with recall. These acceptable window for the one-year follow-up visit is as follows:

Visit	Acceptable Window
1 Year	≥ 12 Months

The functional outcome surveys were also completed at the baseline visit to reflect their quality-of-life and function prior to surgery. These baseline scores will be used as adjustment variables in each model.

We will estimate the effect of post-operative prophylactic antibiotic duration on one-year patient functional outcomes (MSTS-87 and MSTS-93 scores) and quality-of-life (TESS survey) (**Table 7**). To do so, we will use multiple linear regression models that include the following independent variables: randomized treatment group, tumor location (femur versus tibia), clinical site and baseline score. The results will be reported as mean differences with 95% CIs. We hypothesize that a longer duration (five days) of post-operative prophylactic antibiotics will result in improved patient functional outcomes. We will use multiple imputation to address missing data in the functional and quality-of-life outcomes should the amount of missing data be considerable but not too substantial. Convention dictates that if more than five but less than 40 percent of data is missing, the use of multiple imputation is appropriate and warranted[21].

Table 7: Functional and Quality-of-Life Outcomes

Endpoint	Relevant Variable (Case Report Form & Question No.)	Treatment X N = XXX Mean (SD)	Treatment Y N = XXX Mean (SD)	Mean Difference (95% CI)	P-Value
**Functional and Quality-of-Life Outcomes**					
MSTS-87	Forms 19.1 – 19.6				
MSTS-93	Form 20.1				
TESS	Form 21.1 – 21.5				

Antibiotic-Related Complications

We will also estimate the effect of post-operative prophylactic antibiotic duration on the rates of antibiotic-related complications using the Cox proportional model (**Table 8**). We hypothesize that a longer duration (five days) of post-operative prophylactic antibiotics will result in greater antibiotic-related complications. We will only perform Cox regressions for individual antibiotic-related complications if there are enough events. Should there be an insufficient number of events, we will summarize by treatment group and report using descriptive statistics (frequencies and proportions).

Table 8: Antibiotic-Related Complications

Endpoint	Relevant Variable (Case Report Form & Question No.)	Treatment X N = XXX n (%)	Treatment Y N = XXX n (%)	Hazard Ratio (95% CI)	P-Value
**Any Antibiotic-Related Complication**	Adjudicated Data				
Stomach Cramps	Adjudicated Data				
Nausea / Vomiting	Adjudicated Data				
Oral Candidiasis	Adjudicated Data				
Unusual Bleeding / Bruising	Adjudicated Data				
Difficulty Breathing	Adjudicated Data				
Sore Mouth / Throat	Adjudicated Data				
Allergic Reaction (Itching, Drug Fever, Skin Rash, Anaphylaxis)	Adjudicated Data				
Anemia / Low Blood Counts	Adjudicated Data				
Skin Reaction	Adjudicated Data				
Diarrhea	Adjudicated Data				
Liver Toxicity	Adjudicated Data				
Kidney Toxicity	Adjudicated Data				
Clostridium difficile Associated Colitis	Adjudicated Data				
Toxic Megacolon	Adjudicated Data				
Opportunistic Fungal Infection	Adjudicated Data				
Indwelling-Catheter Related Sepsis	Adjudicated Data				
Other Antibiotic-Related Event	Adjudicated Data				

Unplanned Re-Operations, Oncologic Events, and Mortality

Finally, we will also explore the effect of post-operative prophylactic antibiotic duration on the rates of unplanned re-operations, oncologic events and all-cause mortality using the Cox proportional model (**Tables 9**, **10** and **11**). We will only perform Cox regressions for individual types of re-operations and individual oncologic events if there are enough events. Should there be an insufficient number of events, we will summarize by treatment group and report using descriptive statistics (frequencies and proportions).

Table 9: Unplanned Re-Operations

Endpoint	Relevant Variable (Case Report Form & Question No.)	Treatment X N = XXX n (%)	Treatment Y N = XXX n (%)	Hazard Ratio (95% CI)	P-Value
**Any Unplanned Re-Operation**	Adjudicated Data				
Implant Revision	Adjudicated Data				
Irrigation and Debridement	Adjudicated Data				
Wound Flap	Adjudicated Data				
Skin Graft	Adjudicated Data				
Bone Graft	Adjudicated Data				
Implant Exchange	Adjudicated Data				
Extensor Mechanism Reconstruction	Adjudicated Data				
Repeat Tumor Excision	Adjudicated Data				
Antibiotic Spacer Insertion	Adjudicated Data				
Patellar Reconstruction	Adjudicated Data				
Abductor Reconstruction	Adjudicated Data				
Rotationplasty	Adjudicated Data				
Amputation	Adjudicated Data				
Other Unplanned Re-Operation	Adjudicated Data				

Table 10: Oncologic Events

Endpoint	Relevant Variable (Case Report Form & Question No.)	Treatment X N = XXX n (%)	Treatment Y N = XXX n (%)	Hazard Ratio (95% CI)	P-Value
**Any Oncologic Event**	Form 13.1, Q2				
Local Recurrence	Form 13.1, Q2				
Distant Metastases	Form 13.1, Q2				

Table 11: Mortality

Endpoint	Relevant Variable (Case Report Form & Question No.)	Treatment X N = XXX n (%)	Treatment Y N = XXX n (%)	Hazard Ratio (95% CI)	P-Value
**Mortality Due To Any Cause**	Adjudicated Data				
Mortality Due To Disease Progression	Adjudicated Data				

References

1. Gamble C, Krishan A, Stocken D, et al. Guidelines for the Content of Statistical Analysis Plans in Clinical Trials. *JAMA*. 2017;318(23):2337-2343. doi:10.1001/jama.2017.18556

2. Moher D, Hopewell S, Schulz KF, et al. CONSORT 2010 Explanation and Elaboration: updated guidelines for reporting parallel group randomised trials. *BMJ*. 2010;340:c869. doi:10.1136/bmj.c869

3. Ilyas I, Younge D, Pant R, Moreau P. Limb salvage for proximal tibial tumours using a modular prosthesis. *Int Orthop*. 2000;24(4):208-211. doi:10.1007/s002640000158

4. Reddy KIA, Wafa H, Gaston CL, et al. Does amputation offer any survival benefit over limb salvage in osteosarcoma patients with poor chemonecrosis and close margins? *Bone Jt J*. 2015;97-B(1):115-120. doi:10.1302/0301-620X.97B1.33924

5. Ruggieri P, Mavrogenis AF, Mercuri M. Quality of life following limb-salvage surgery for bone sarcomas. *Expert Rev Pharmacoecon Outcomes Res*. 2011;11(1):59-73. doi:10.1586/erp.10.91

6. Jeys L, Grimer R. The Long-Term Risks of Infection and Amputation with Limb Salvage Surgery Using Endoprostheses. In: Tunn P-U, ed. *Treatment of Bone and Soft Tissue Sarcomas*. Recent Results in Cancer Research. Springer Berlin Heidelberg; 2009:75-84. doi:10.1007/978-3-540-77960-5_7

7. Jeys LM, Grimer RJ, Carter SR, Tillman RM. Risk of amputation following limb salvage surgery with endoprosthetic replacement, in a consecutive series of 1261 patients. *Int Orthop*. 2003;27(3):160-163. doi:10.1007/s00264-003-0429-x

8. Hasan K, Racano A, Deheshi B, et al. Prophylactic antibiotic regimens in tumor surgery (PARITY) survey. *BMC Musculoskelet Disord*. 2012;13:91. doi:10.1186/1471-2474-13-91

9. Akahane T, Shimizu T, Isobe K, Yoshimura Y, Fujioka F, Kato H. Evaluation of postoperative general quality of life for patients with osteosarcoma around the knee joint. *J Pediatr Orthop B*. 2007;16(4):269-272. doi:10.1097/BPB.0b013e3280925670

10. Gutowski CJ, Zmistowski BM, Clyde CT, Parvizi J. The economics of using prophylactic antibiotic-loaded bone cement in total knee replacement. *Bone Jt J*. 2014;96-B(1):65-69. doi:10.1302/0301-620X.96B1.31428

11. Ghert M, Deheshi B, Holt G, et al. Prophylactic antibiotic regimens in tumour surgery (PARITY): protocol for a multicentre randomised controlled study. *BMJ Open*. 2012;2(6). doi:10.1136/bmjopen-2012-002197

12. 9 Surgical Site Infection (SSI) Event. Published online 2019:34.

13. Tunn PU, Pomraenke D, Goerling U, Hohenberger P. Functional outcome after endoprosthetic limb-salvage therapy of primary bone tumours--a comparative analysis using the MSTS score, the TESS and the RNL index. *Int Orthop*. 2008;32(5):619-625. doi:10.1007/s00264-007-0388-8

14. Davis AM, Wright JG, Williams JI, Bombardier C, Griffin A, Bell RS. Development of a measure of physical function for patients with bone and soft tissue sarcoma. *Qual Life Res Int J Qual Life Asp Treat Care Rehabil*. 1996;5(5):508-516.

15. Schreiber D, Bell RS, Wunder JS, et al. Evaluating function and health related quality of life in patients treated for extremity soft tissue sarcoma. *Qual Life Res Int J Qual Life Asp Treat Care Rehabil*. 2006;15(9):1439-1446. doi:10.1007/s11136-006-0001-4

16. Järvinen TLN, Sihvonen R, Bhandari M, et al. Blinded interpretation of study results can feasibly and effectively diminish interpretation bias. *J Clin Epidemiol*. 2014;67(7):769-772. doi:10.1016/j.jclinepi.2013.11.011

17. Wang R, Lagakos SW, Ware JH, Hunter DJ, Drazen JM. Statistics in medicine--reporting of subgroup analyses in clinical trials. *N Engl J Med*. 2007;357(21):2189-2194. doi:10.1056/NEJMsr077003

18. Sun X, Briel M, Walter SD, Guyatt GH. Is a subgroup effect believable? Updating criteria to evaluate the credibility of subgroup analyses. *BMJ*. 2010;340:c117. doi:10.1136/bmj.c117

19. Briel M, Lane M, Montori VM, et al. Stopping randomized trials early for benefit: a protocol of the Study Of Trial Policy Of Interim Truncation-2 (STOPIT-2). *Trials*. 2009;10:49. doi:10.1186/1745-6215-10-49

20. Montori VM, Devereaux PJ, Adhikari NKJ, et al. Randomized trials stopped early for benefit: a systematic review. *JAMA*. 2005;294(17):2203-2209. doi:10.1001/jama.294.17.2203

21. Jakobsen JC, Gluud C, Wetterslev J, Winkel P. When and how should multiple imputation be used for handling missing data in randomised clinical trials – a practical guide with flowcharts. *BMC Med Res Methodol*. 2017;17(1):162. doi:10.1186/s12874-017-0442-1
