# Supplementary material for: Comparison of Prophylactic Intravenous Antibiotic Regimens After Endoprosthetic Reconstruction for Lower Extremity Bone Tumors: A Randomized Clinical Trial
Source: JAMA Oncol. 2022 Jan 6;8(3):345–53. doi: 10.1001/jamaoncol.2021.6628 (PMC8739829; doi:10.1001/jamaoncol.2021.6628)
Supplement: Supplement 2. — eAppendix. Supplemental Methods eTable 1. Reasons for Exclusion Prior to Randomization eTable 2. Reasons for Exclusion After Adjudication Committee Review eTable 3. Patient Demographics and Baseline Details eTable 4. Tumor Details eTable 5. Surgical and Perioperative Management Details eTable 6. Prophylactic Antibiotic Administration Details eTable 7. Sensitivity Analyses eTable 8. Subgroup Analyses eTable 9. Study Outcomes by Treatment Group (Primary and Secondary) eTable 10. Functional and Quality of Life Outcomes by Treatment Group [file jamaoncol-e216628-s002.pdf]

Supplemental Online Content

The Prophylactic Antibiotic Regimens in Tumor Surgery (PARITY) Investigators. Comparison of prophylactic intravenous antibiotic regimens after endoprosthetic reconstruction for lower extremity bone tumors. *JAMA Oncol*. Published online January 6, 2022. doi:10.1001/jamaoncol.2021.6628

eAppendix. Supplemental Methods

eTable 1. Reasons for Exclusion Prior to Randomization

eTable 2. Reasons for Exclusion After Adjudication Committee Review

eTable 3. Patient Demographics and Baseline Details

eTable 4. Tumor Details

eTable 5. Surgical and Perioperative Management Details

eTable 6. Prophylactic Antibiotic Administration Details

eTable 7. Sensitivity Analyses

eTable 8. Subgroup Analyses

eTable 9. Study Outcomes by Treatment Group (Primary and Secondary)

eTable 10. Functional and Quality of Life Outcomes by Treatment Group

This supplemental material has been provided by the authors to give readers additional information about their work.

© 2022 The PARITY Investigators. *JAMA Oncology*.

eAppendix. Supplemental Methods

1.0 PARITY Investigators

Study Management & Committees

The Global Methods Center at McMaster University coordinated the trial. The Global Methods Center was responsible for the set-up of the trial randomization system, the set-up and maintenance of the study database, data validation, data analyses and clinical site coordination. The Steering Committee designed the trial, assisted with the development of the statistical analysis plan, and vouch for the completeness and accuracy of the data and analyses. The first author (M.G.), the Chair of the Writing Committee, and second author (P.S.) wrote the first draft of the manuscript. The remaining members of the Writing Committee provided important intellectual content and critically revised the manuscript. The Writing Committee assumes full responsibility for the overall content and integrity of the manuscript.

Writing Committee: Michelle Ghert, MD (Chair, McMaster University); Patricia Schneider, BSc (McMaster University); Gordon Guyatt, MD (McMaster University); Lehana Thabane, PhD (McMaster University); Roberto Vélez, MD, PhD (Vall d'Hebron Institut de Recerca); Timothy O'Shea, MD, MPH (McMaster University); R. Lor Randall, MD (University of California, Davis); Robert Turcotte, MD (McGill University); David Wilson, MD, MASc (McMaster University); Jay S. Wunder, MD (University of Toronto); André Mathias Baptista, MD, PhD (Universidade de São Paulo); Edward Y. Cheng, MD (University of Minnesota); Yee-Cheen Doung, MD (Oregon Health and Science University); Peter C. Ferguson, MD (University of Toronto); Victoria Giglio, MSc (McMaster University); James Hayden, MD, PhD (Oregon Health and Science University); Diane Heels-Ansdell, MSc (McMaster University); Shah Alam Khan, MS (Ortho) (All India Institute of Medical Sciences); Venkatesan Sampath Kumar, MS (Ortho) (All India Institute of Medical Sciences); Paula McKay, BSc (McMaster University); Benjamin Miller, MD, MS (University of Iowa); Michiel van de Sande, MD, PhD (Universiteit Leiden); Juan P. Zumárraga, MD, MSc (Universidad San Francisco de Quito); Mohit Bhandari, MD, PhD (McMaster University)

Global Methods Center: Michelle Ghert, MD [Principal Investigator]; Patricia Schneider, BSc [Research Manager]; Victoria Giglio, MSc, Paula McKay, BSc, Andrew Duong, MSc, Nathan Evaniew, MD, PhD, Dana Ghanem, BSc, Callum MacLeay, BSc, Kim Madden, PhD, Antonella Racano, DO, Taryn Scott, MSW, MSc, Marilyn Swinton, MSc [Project Management]; Nicole Simunovic, MSc [Grants Administration]; Sheila Sprague, PhD [Research Methodologist]; Diane Heels-Ansdell, MSc [Statistical Analysis]; Lisa Buckingham, BSc [Data Management] (McMaster University)

European Coordinating Center / Legal Authorized Representative: Roberto Vélez, MD, PhD [Principal Investigator / Legal Authorized Representative], Alba Lopez Fernandez, PhD, Olga Sánchez-Maroto Carrizo, MS [Project Management] (Vall d'Hebron Institut de Recerca)

Steering Committee: Michelle Ghert, MD (Chair, McMaster University), Mohit Bhandari, MD, PhD (Co-Chair, McMaster University); Benjamin Deheshi, MD, MSc (McMaster University); Gordon Guyatt, MD (McMaster University); Ginger Holt, MD (Vanderbilt University); Timothy O'Shea, MD, MPH (McMaster University); R. Lor Randall, MD (University of California, Davis); Lehana Thabane, PhD (McMaster University); Roberto Vélez, MD, PhD (Vall d'Hebron Institut de Recerca); Jay S. Wunder, MD (University of Toronto)

Central Adjudication Committee: Michelle Ghert, MD (Chair, McMaster University); Timothy O'Shea, MD (McMaster University); R. Lor Randall, MD (University of California, Davis); Robert Turcotte, MD (McGill University); David Wilson, MD, MASc (McMaster University)

Data and Safety Monitoring Board: Peter Rose, MD (Chair, Mayo Clinic); Brian Brigman, MD (Duke University); Eleanor Pullenayegum, PhD (The Hospital for Sick Children)

© 2022 The PARITY Investigators. *JAMA Oncology*.

Participating Clinical Sites

The participating clinical sites are listed as follows: Canada, the United States and International. Within each region, the order of participating clinical sites is based on patient enrolment, in the order of highest to lowest enrolment.

Canada

Mount Sinai Hospital (Toronto, ON) – Peter C. Ferguson, MD (PI), Jay S. Wunder, MD [Site Investigators]; Anthony M. Griffin, MSc [Research Coordination]; Gagan Grewal, BSc, R.PhT, Andrew Han, BScPhm, Ioanna Mantas, BScPhm, ACPR, RPh, Andrew Wylie, BScPhm, ACPR, PharmD [Pharmacy]

McGill University Health Centre (Montreal, QC) – Robert Turcotte, MD (PI), Krista Goulding, MD, MPH [Site Investigators]; Nicole Andersen, BSc, Olivier Bouchereau, MSc, Firas Dandachli, MD, MSc, Mireille Dessureault, MSc, Steven Salomon, MSc, Nathalie Ste-Marie, MSc [Research Coordination]; Ariane Lessard, PharmD, Gilbert Matte, PharmD [Pharmacy]

Juravinski Hospital and Cancer Centre (Hamilton, ON) – Michelle Ghert, MD (PI), Benjamin Deheshi, MD, MSc, David Wilson, MD, MASc [Site Investigators]; Zoe Bond, BSc, Nathan Evaniew, MD, PhD, Dana Ghanem, BSc, Victoria Giglio, MSc, Bo Xuan Lin, BSc, Callum MacLeay, MSc, Antonella Racano, DO, Patricia Schneider, BSc [Research Coordination]; Maya Biljan, RPhT, Rita Chan, BScPharm, MSc, Deanna Cosentino, RPhT, Diane Lourenco, RPhT, Brittany Marriott, RPhT, Gita Sobhi, RPh [Pharmacy]

CIUSSS de l'Est-de-l'Île-de-Montréal – Hôpital Maisonneuve-Rosemont (Montreal, QC) – Marc Isler, MD (PI), Sophie Mottard, MD [Site Investigators]; Janie Barry, MSc, Hugo Saint-Yves, MSc, Marysa Bétournay [Research Coordination]; Marceline Quach, BPharm, MSc, Helen Assayag, BPharm, Karine Daoust, BPharm, Kristine Goyette, BPharm, Denis Projean, BPharm, PhD, Millie Lum, BPharm, Ariane Lessard BPharm, Maude Bachand-Fournier, BPharm [Pharmacy]

CHU de Québec – Université Laval (Québec, QC) – Norbert Dion, MD (PI), Annie Arteau, MD [Site Investigators]; Sylvie Turmel, RN [Research Coordination]; Anne Bertrand, MSc, Manon D'Amours, PTA, Lucie Dallaire, MSc, Nancy Gagnon, MSc, Lucie Gosselin, MSc, Gladys Grenier, PTA, Véronique Labbé, MSc, Tuong-Vi Tran, MSc, PhD [Pharmacy]

Vancouver General Hospital (Vancouver, BC) – Paul Clarkson, MBChB, MSc (PI) [Site Investigator]; Lisa Kondo, BScN, RN, Baohua Wang, PhD [Research Coordination]; Judy Yip, RPh [Pharmacy]

The Ottawa Hospital (Ottawa, ON) – Joel Werier, MD (PI), Hesham Abdelbary, MD, MSc [Site Investigators]; Yusra Kassim, MD, PhD, Heather Cosgrove, BA, Kimberly Paquin, BA [Research Coordination]; Anne-Marie Dugal, RPhT, Susan Fetzer, RPhT, Wendy Aikens, RPhT [Pharmacy]

Foothills Medical Centre (Calgary, AB) – Shannon Puloski, MD (PI), Michael Monument, MD, MSc [Site Investigators]; Kimberly Carcary, MSc, Olesja Hazenbiller, MSc, Kayla Kashluba, MSc, Jimena Rodriguez, MSc [Research Coordination]; Candice Cameron, BA, BSP, ACPR [Pharmacy]

United States

Oregon Health & Science University Hospital (Portland, OR) – Yee-Cheen Doung, MD (PI), Kenneth Gundle, MD, James Hayden, MD, PhD [Site Investigators]; Christopher Hart, MD, David Jenkins, BA, Rebecca I. Wetzel, BS [Research Coordination]; Krista Wolf, PharmD, Brooke Bernard, PharmD, Sara Blefgen, RPh [Pharmacy]

Huntsman Cancer Institute (Salt Lake City, UT) – Kevin Jones, MD (PI; 2018 – 2020), R. Lor Randall, MD (PI; 2013 – 2018), John Groundland, MD, MS [Site Investigators]; Susie Crabtree, AS, Jacqueline Hart, AS, Sara Shaw, BS [Research Coordination]; Rian Davis, PharmD, Winter Redd, PharmD, Susan Sorenson, PharmD [Pharmacy]

Holden Comprehensive Cancer Center (Iowa City, IA) – Benjamin Miller, MD, MS (PI), Mohammed Milhem, MBBS, Jill Kain, MSN, ARNP [Site Investigators]; Marian Andersen, MA, CCRP, Kathryn Hillburn, RN, BSN, Jennifer Larson, AAS, CMA, Nancy McCurdy, RN, Alyssa Pratt, MS, CCRP, Mary Schall, BSN [Research Coordination]; Theresa Hobbs BSPharm, RPh, Kristine Johnson, BSPharm, RPh, Joanna Nohr, PharmD, Wendi Slaughter, PharmD, RPh [Pharmacy]

© 2022 The PARITY Investigators. *JAMA Oncology*.

Vanderbilt University Medical Center (Nashville, TN) – Ginger Holt, MD (PI), Jennifer Halpern, MD, Herbert Schwartz, MD [Site Investigators]; Julie Daniels, CCRP, Eden Schafer, MPH [Research Coordination]; M. Shane Moore, PharmD [Pharmacy]

Memorial Sloan Kettering Cancer Center (New York, NY) – John H. Healey, MD (PI) [Site Investigators]; Kaity Chang, MBA, Linda Chen, MS, Olivera Douvelis, BA, Jesse Galle, BA, Marissa Mezzancello, MPH, MS, Yoely Tavarez, BA [Research Coordination]; Brian Del Corral, PharmD, Sabrina Lopez, PharmD, Gerry O'Neill, PharmD [Pharmacy]

The Rothman Institute at Thomas Jefferson University Hospital (Philadelphia, PA) – John Abraham, MD (PI), Scot Brown, MD [Site Investigators]; Meghan Angelos, Keenan Sobol, BS, John Strony, BS [Research Coordination]; Braden Rall, PharmD, BCPS, BCOP, Melissa Furio, PharmD, Linda Sailor, PharmD, Rania Sadaka, PharmD, Lauren Karel, PharmD, BCPS [Pharmacy]

Montefiore Medical Center (Bronx, NY) – David Geller, MD (PI), Bang Hoang, MD [Site Investigators]; Janet Tingling, AA, AS, BS, MS, MBA, PhD [Research Coordination]; Clemencia Solorzano, PharmD, RPh [Pharmacy]

University of California, San Francisco Medical Center (San Francisco, CA) – Rosanna Wustrack, MD (PI), Richard O'Donnell, MD, Melissa Zimel, MD [Site Investigators]; Veronica Andaya, BA, Adrianna Carrasco, BS [Research Coordination]; Shirley Chen, PharmD, Diana Ng, PharmD, Yelena Koplowicz, PharmD [Pharmacy]

University of Florida Health Shands Hospital (Gainesville, FL) – André Spiguel, MD (PI), Chung Ming Chan, MD, Charles Parker Gibbs, MD, Mark Scarborough, MD, MaryBeth Horodyski, EdD, LAT, ATC, FNATA [Site Investigators]; Johanna Carmona, LPN, Alana Jackson, MS, Aimee Struk, Med, MBA, LAT, ATC [Research Coordination]; Susan Beltz, PharmD, Justin C. Giaquinta, PharmD, Melissa Johnson, PharmD [Pharmacy]

University of Minnesota (Minneapolis, MN) – Edward Y. Cheng, MD (PI) [Site Investigator]; Julie Agel, MA, ATC [Research Coordination]; Theresa Christiansen, RPh, Derek LaBar, PharmD, Darlette Luke, RPh [Pharmacy]

Stanford University Health Care (Palo Alto, CA) – Raffi Avedian, MD (PI) [Site Investigators]; Linda Jordan, PA-C, Deborah Kenney, MS, OTR [Research Coordination]; Steven Chinn, PharmD, Martha Hamilton, PharmD, Scott Mayeda, PharmD [Pharmacy]

Johns Hopkins Hospital (Baltimore, MD) – Carol Morris, MD, MS (PI), Adam Levin, MD [Site Investigators]; Kari Albery, PA-C, Jennifer Giordano, CRNP, Vaishali Laljani [Research Coordination]; Anne Delisa, PharmD [Pharmacy]

The Cleveland Clinic (Cleveland, OH) – Nathan Mesko, MD (PI), Lukas Nystrom, MD [Site Investigators]; Matthew Rerko, Heather Keaney, MPH [Research Coordination]; Rachael Yim, PharmD, MPH, John Petrich MS, RPh [Pharmacy]

Boston Children's Hospital (Boston, MA) – Megan E. Anderson, MD (PI), Mark C. Gebhardt, MD [Site Investigators]; Benjamin Allar, MD, Michael Greenberg, BS, Manahil Naqvi, MS, Ellis Prather, MBA, Emily Rademacher, BS, Jodie Shea, BS [Research Coordination]; James Bennett, PharmD, Stacey Albuquerque, BS, PharmD, Michael Giarrusso, PharmD [Pharmacy]

MedStar Georgetown Cancer Institute at Franklin Square (Baltimore, MD) – Albert J. Aboulafia, MD, MBA (PI), Matthew T. Wallace, MD, MBA [Site Investigators]; Sally Brown, RN, BSN, MGA, OCN, Janice Fowler, Jean Flack, RN, BSN, OCN, CCRC [Research Coordination]; Rick Battersby, RPh, Chad Taylor, PharmD [Pharmacy]

Saint Louis University (St. Louis, MO) – David Greenberg, MD (PI) [Site Investigators]; Sarah Dawson, RN, BSN [Research Coordination]; Adam Riebeling, PharmD, Anna Schmidt, PharmD, BCPS [Pharmacy]

Dartmouth-Hitchcock Medical Center (Lebanon, NH) – Eric Henderson, MD (PI) [Site Investigators]; Peter DePalo Sr, BS, CCRP, CPhT, Lisa Mack, RN, Christine Neely-Jones, RN, Crystallee Newton, BA, CCRC, Daniel Ressler,

© 2022 The PARITY Investigators. *JAMA Oncology*.

BA, Holly Symonds, CCRC [Research Coordination]; Iryna Gardner, CPhT, Douglas Parr, PharmD, Victoria Poisson, CPhT, David Rozolsky, PharmD, Patrick Teune, PharmD [Pharmacy]

Massachusetts General Hospital (Boston, MA) – Joseph Schwab, MD (PI), Santiago A. Lozano-Calderon, MD, PhD [Site Investigators]; Jonathan Baker, BS, Emily Ann Berner, BS, Gi Hye Im, BA, Jason Kim, BS, Christine Park, BS, Rishabh Phukan, BS, Zachary Wright, BS, Sarah Yeates, BS [Research Coordination]; Lalit Joshi, RPh [Pharmacy]

State University of New York Upstate Orthopedics (Syracuse, NY) – Timothy Damron, MD (PI) [Site Investigators]; Tina Craig, CCRP [Research Coordination]; Melissa Reale [Pharmacy]

Albany Medical Center (Albany, NY) – Matthew R. DiCaprio, MD (PI), Bradford A. Palmer, RPA-C [Site Investigators]; Toni Schaeffer, PharmD, Elena Cioppa, RPh, MS [Pharmacy]

Froedtert Hospital (Milwaukee, WI) – John C. Neilson, MD (PI), David M. King, MD, Adam N. Wooldridge, MD, MPH [Site Investigators]; Karen C. Gonzalez, MS, CCRP, Marie Ellestad, CCRP [Research Coordination]; Kate Lewis, PharmD, BCPS, Tom Nelson, PharmD, RPh [Pharmacy]

Emory University Orthopedics and Spine Center (Atlanta, GA) – Nickolas Reimer, MD (PI), David Monson, MD, Shervin Oskouei, MD [Site Investigators]; Christina Lomba, MS, CCRC, Lauren Glenney, MHSc, CCRP [Research Coordination]; Susan Rogers, RPh [Pharmacy]

Long Island Jewish Medical Center [Northwell Health] (New Hyde Park, NY) – Howard Goodman, MD (PI) [Site Investigators]; Marlena McGill, MPH, Peter Olivares, BSc, Francesca Petruccelli, BA [Research Coordination]; Uzma Afzal, PharmD, Zina Faynblat, RPh, Elizabeth Mathew, RPh [Pharmacy]

Sinai Hospital of Baltimore [Lifebridge Health] (Baltimore, MD) – Albert Aboulafia, MD, MPH (PI), Matthew T. Wallace, MD [Site Investigators]; Wanda Bell-Farrell, RN, MS, CCRP, Judith Bosley, RN, BSN, Corilynn Hughes, RN, BSN, OCN, Ukeme Ikiddeh-Barnes, RN, Ashley Jones, BS, Melissa Loomis, CCRP, Alexis Solis, BS, Christine Wade, BA [Research Coordination]; Stephanie Friedman, PharmD, Chukwuemeka N. Nzelibe, PharmD [Pharmacy]

Beth Israel Deaconess Medical Center (Boston, MA) – Megan E. Anderson, MD (PI), Mark C. Gebhardt, MD [Site Investigators]; Katiri Wagner, BS [Research Coordination]; Hina A. Jolin, PharmD, Heena Patel, RPh [Pharmacy]

Cincinnati Children's Hospital (Cincinnati, OH) – Joel Sorger, MD (PI) [Site Investigators]; Nichole Leitsinger, BS, CCRP [Research Coordination]; Krista Carpenter, APRN Denise LaGory, RPh [Pharmacy]

University of California, Davis Medical Center (Sacramento, CA) – Steven Thorpe, MD (PI), R. Lor Randall, MD [Site Investigators]; Shari Lynn Nichols, CCRP, ADN [Research Coordination]; Patrick Febre, PharmD, Jacob Monares, CPhT, Kimmai Nguyen, PharmD, Nadir Sarwary, CPhT, Peter Trovitch, PharmD [Pharmacy]

University of California, Los Angeles Medical Center (Los Angeles, CA) – Nicholas Bernthal, MD (PI), Jeffrey Eckardt, MD, Francis Hornicek, MD, PhD [Site Investigators]; Stephen Zoller, MD, Gloria Kiel [Research Coordination]; Jason Madamba, PharmD, BCPS, Christina Shin, PharmD [Pharmacy]

Hartford Hospital (Hartford, CT) – Adam Lindsay, MD (PI) [Site Investigators]; Jamie Fish-Fuhrmann, BS [Research Coordination]

Maimonides Medical Center (New York, NY) – Howard Goodman, MD (PI) [Site Investigators]; Maya Culbertson, MS [Research Coordination]; Patricia Caruso-Prendergast, MS, PharmD, BCPS, Emily Garling, PharmD [Pharmacy]

University of Arkansas for Medical Sciences (Little Rock, AR) – Richard Nicholas, MD (PI), Corey Montgomery, MD [Site Investigators]; J. Aaron Holley, BS, Rachel Jones, MSc, Melissa McAdoo, BSN, Daisy Wade, BA [Research Coordination]; Mindy Caid, BS, Amy Crisp, PharmD, Jennifer Roberts, PharmD [Pharmacy]

UConn Health (Farmington, CT) – Adam Lindsay, MD (PI: 2018 – 2020), Tessa Balach, MD (PI: 2014 – 2017) [Site Investigators]; Mark Cote, PT, DPT, MS, Kathleen Coyle, RN, BSN, MPH, Kelly Rushlow, BA [Research Coordination]; Ruth LaCasse, RPh [Pharmacy]

© 2022 The PARITY Investigators. *JAMA Oncology*.

University of Maryland Medical Center (Baltimore, MD) – Daniel Lerman, MD (PI) [Site Investigators]; Andrea Howe, BS [Research Coordination]; Prashant Patel, PharmD, Andrew Phan, PharmD, Shinyi Telscher, PharmD, CCRP [Pharmacy]

University of Pittsburgh Medical Center (Pittsburgh, PA) – Kurt Weiss, MD (PI), Mark Goodman, MD [Site Investigators]; Alma Heyl, CCRC, LAS [Research Coordination]; Chris Korenoski, PharmD, Chris Ann Yeschke, PharmD [Pharmacy]

Wexner Medical Center (Columbus, OH) – Thomas Scharschmidt, MD (PI), Joel Mayerson, MD [Site Investigators]; Martha Crist, RN [Research Coordination]; Hallie Barr, PharmD, BCOP [Pharmacy]

International

All India Institute of Medical Sciences (New Delhi, India) – Shah Alam Khan, MS (Ortho) (PI), Venkatesan Sampath Kumar, MS (Ortho) [Site Investigators]; Abhinav Agarwal, MS (Ortho) Roshan Banjara, MS (Ortho), Sanjay Oli, BA (Sociology) [Research Coordination]

Instituto de Ortopedia e Traumatologia da Universidade de São Paulo (São Paulo, Brazil) – André Mathias Baptista, MD, PhD (PI), Olavo Pires de Camargo, MD, PhD, Juan Pablo Zumárraga, MD, MSc, PhD [Site Investigators]; Juliana Freitar, RN, Ismael Agomes, RN [Pharmacy]

Leiden University Medical Center (Leiden, the Netherlands) – P.D. Sander Dijkstra, MD, PhD (PI), Michiel van de Sande, MD, PhD (Co-PI) [Site Investigators]; Philip Sanders, MD, Sarah Bosma, MD [Research Coordination]; Marieke Afra Toi, PharmD [Pharmacy]

Hospital Universitario Austral (Buenos Aires, Argentina) – Marcos Galli Serra, MD (PI), Walter Parizzia, MD [Site Investigators]; Gabriela Marinsalta, BIOCH, Angela Podrzaj [Research Coordination]; Mariana Foa Torres, RN [Pharmacy]

Hospital Vall d'Hebron (Barcelona, Spain) – Roberto Vélez, MD, PhD (PI), Manuel Pérez, MD [Site Investigators]; Alba Lopez Fernandez, PhD [Research Coordination]; Lourdes Girona Brumós, PharmD, Pilar Suñé, PharmD [Pharmacy]

School of Clinical Medicine, University of KwaZulu-Natal/Grey's Hospital (Pietermaritzburg, South Africa) – Reitze Rodseth, MD, PhD (PI), Leonard Marais, MD, PhD (Co-PI), Luan Nieuwoudt, MD, Chantal Rajah, MD [Site Investigators]; Simphiwe Gumede [Research Coordination]

Medical University Graz (Graz, Austria) – Andreas Leithner, MD (PI), Marko Bergovec, MD [Site Investigators]; Andrea Fink, MSc [Research Coordination]; Carina Halb, Mag [Pharmacy]

Children's Cancer Hospital Egypt (Cairo, Egypt) – Ahmed El Ghoneimy, MD (PI) [Site Investigators]; Dina Elgalaly, BPharm, Nehal Kamal, BPharm [Pharmacy]

Hospital de Clínicas de Porto Alegre (Porto Alegre, Brazil) – Ricardo Becker, MD, MSc, PhD (PI), Bruno Pereira Antunes, MD, MSc, Carlos Roberto Galia MD, MSc, PhD [Site Investigators]; Julie F. Cerutti Santos, RN, MSc [Research Coordination]; Daniel Fasolo, BPharm, MSc, PhD [Pharmacy]

Singapore General Hospital (Singapore, Singapore) – Mann Hong Tan, MBBS (Singapore), FRCS (Edinburgh), FRCS (Glasgow), FAMS (Orthopaedic Surgery) (PI), Suraya Zainul Abidin, MBBS BSc (Hons), MMed (Ortho), FRCS (Edinburgh) [Site Investigators]; Lai Ye, CHEANG, MPharm [Pharmacy]

Royal Adelaide Hospital (Adelaide, Australia) – Mark Clayer, MD, MSc, MBBS (PI), Jakub Jagiello, MBBS, David Morris, MD [Site Investigators]; Yee Chai, BPharm, Steven Duong, BPharm, Tran Nguyen, BPharm, Peter Slobodian, BPharm, MClinPharm [Pharmacy]

University Medical Center Groningen (Groningen, the Netherlands) – Paul Jutte, MD, PhD (PI) [Site Investigators]; Marlanka Zuur, PhD [Pharmacy]

© 2022 The PARITY Investigators. *JAMA Oncology*.

2.0 Eligibility Criteria

Patients who satisfied all the inclusion criteria and did not meet any of the exclusion criteria below were to be included in the study.

Inclusion Criteria

1. Males and females 12 years of age or older;
2. Primary bone malignancies or benign aggressive tumors of the femur or tibia, soft-tissue sarcomas which have invaded the femur or tibia, or oligometastatic bone disease of the femur or tibia in a patient expected to live at least one year post-operatively[†];
3. Treatment by excision and endoprosthetic reconstruction of femur or tibia[*]; and
4. Provision of informed consent.

[†]*During the transition from the vanguard to the definitive phase of the trial, the Steering Committee expanded eligibility to also include patients with oligometastatic bone disease with expected survival of at least one year due to the similarities between these patient populations and in order to increase the pace of recruitment;*
[*]*Expandable prostheses acceptable.*

Exclusion Criteria

1. Current known methicillin-resistant *Staphylococcus aureus* (MRSA) or vancomycin-resistant *Enterococcus* (VRE) skin colonization[†];
2. Documented anaphylaxis or angioedema to penicillin or the study antibiotics (cefazolin or cefuroxime);
3. Current surgical procedure is a revision surgery for implant failure or infection[*];
4. Prior local infection within the surgical field of the limb[*];
5. Current known immunologically-deficient disease conditions (not including recent chemotherapy)[‡];
6. Known renal insufficiency with estimated creatinine clearance (eGFR) of less than 54 mL/min;
7. Reconstruction to include a structural allograft;
8. Likely problems, in the judgement of the investigator, with maintaining follow-up;
9. Enrolled or previously randomized in a competing study; or
10. Current weight less than or equal to 45 kg[ʃ].

[†]*Unable to safely randomize antibiotics in these patients;*
[*]*Higher risk of infection (versus baseline) in patients undergoing revision or with prior infection;*
[‡]*Acquired immunodeficiency conditions (i.e., HIV or prior splenectomy) or inherited immunodeficiency diseases (i.e., Agammaglobulinemia or Severe Combined Immunodeficiency Disorder);*
[ʃ]*For clinical sites using cefuroxime only.*

© 2022 The PARITY Investigators. *JAMA Oncology*.

3.0 Trial Interventions & Standardization of Peri-Operative Care

Given the inherent variability in prophylactic antibiotic practice patterns among orthopaedic oncologists, it was important to ensure that surgeons adhered as closely as possible to the study protocol. As such, the prophylactic antibiotics and peri-operative regimens were standardized.

Selection of Study Antibiotics and Regimens

The ideal prophylactic antibiotic should have activity against the anticipated pathogens, be able to achieve tissue concentrations in excess of the inhibitory concentration of the bacteria at the time of surgery and have negligible toxicity.[1] In preparation for this study, an expert panel of six orthopaedic oncologists and three infectious diseases specialists were consulted. The choice of study antibiotics was based on coverage of the most common pathogens associated with orthopaedic surgical site infections, as well as survey data of participating surgeons.[2,3] Despite the fact that over ten percent of PARITY survey respondents indicated that they prescribe an aminoglycoside or vancomycin in combination with a first-generation cephalosporin, the infectious diseases experts agreed that sufficient antimicrobial coverage is provided by cephalosporins, and that additional gram-negative coverage was not warranted as cephalosporins are a group of broad-spectrum antibiotics that have been shown to be effective against both gram-positive and gram-negative organisms.[3–5] The choice of five-days as the 'experimental' treatment arm was based on consensus among the infectious diseases specialists engaged in preparation for this trial, who agreed that any longer would significantly increase the risk for resistant organisms and would not provide further benefit.

During the transition from the vanguard to the definitive phase of the trial, the Steering Committee consulted with the expert panel of infectious diseases specialists, who agreed that the addition of a comparable antibiotic for clinical sites interested in participating that were not authorized to use cefazolin was warranted. Although subtle differences do exist within the cephalosporin group of antibiotics, many provide similar coverage against pathogens and the majority have similar half-lives of one and a half to two hours, thereby necessitating repeat administration at six-to-eight-hour intervals.[4,5] Therefore, cefuroxime, a second-generation cephalosporin, was selected as the most comparable antibiotic with respect to antimicrobial coverage, half-life and toxicity.

Standardization of Peri-Operative Antibiotic Regimens

Pre-Operative Antibiotic Regimen

All adult patients received 2g of intravenous cefazolin or 1.5g of intravenous cefuroxime pre-operatively within 60 minutes of the procedure. Pediatric patients (less than 18 years of age) received a weight-based dose of intravenous cefazolin that was based on 100mg/kg/day (with a maximum single dose of 2g), or intravenous cefuroxime that was based on 50mg/kg/day (with a maximum single dose of 1.5g), pre-operatively within 60 minutes of the procedure. No other antibiotics were pre-operatively administered within 60 minutes of the procedure.

Intra-Operative Antibiotic Regimen

All adult patients received 2g of intravenous cefazolin or 1.5g of intravenous cefuroxime every three to four hours during surgery. Pediatric patients (less than 18 years of age) received weight-based doses of intravenous cefazolin that were based on 100mg/kg/day (with a maximum single dose of 2g), or intravenous cefuroxime that were based on 50mg/kg/day (with a maximum single dose of 1.5g), every three to four hours during surgery. No other antibiotics were intra-operatively administered.

Trial Interventions

Included patients were randomly allocated to receive either a one- or five-day regimen of post-operative prophylactic intravenous antibiotics. Patients began their randomly allocated post-operative prophylactic intravenous antibiotic regimen within eight hours after skin closure.

One-Day Regimen

All adult patients received 2g of open-label intravenous cefazolin or 1.5g of open-label intravenous cefuroxime every eight hours for 24 hours followed by blinded intravenous saline (i.e., placebo) every eight hours for four additional days or until hospital discharge if acute care stay was less than five days. Pediatric patients (less than 18 years of age) received weight-based doses of open-label intravenous cefazolin that were based on 100mg/kg/day (with a maximum single dose of 2g), or open-label intravenous cefuroxime that were based on 50mg/kg/day (with a maximum single dose of 1.5g), every eight hours for 24 hours followed by blinded intravenous saline every eight hours for four additional days or until hospital discharge if acute care stay was less than five days.

© 2022 The PARITY Investigators. *JAMA Oncology*.

Five-Day Regimen

All adult patients received 2g of open-label intravenous cefazolin or 1.5g of open-label intravenous cefuroxime every eight hours for 24 hours followed by 2g of blinded intravenous cefazolin or 1.5g of blinded intravenous cefuroxime every eight hours for four additional days or until hospital discharge if acute care stay was less than five days. Pediatric patients (less than 18 years of age) received weight-based doses of open-label intravenous cefazolin that were based on 100mg/kg/day (with a maximum single dose of 2g), or open-label intravenous cefuroxime that were based on 50mg/kg/day (with a maximum single dose of 1.5g), every eight hours for 24 hours followed by weight-based doses of blinded intravenous cefazolin that were based on 100mg/kg/day (with a maximum single dose of 2g), or blinded intravenous cefuroxime that were based on 50mg/kg/day (with a maximum single dose of 1.5g), every eight hours for four additional days or until hospital discharge if acute care stay was less than five days.

Other Care

Given the inherent variability in practice patterns among orthopaedic oncologists, it was important to ensure that this study was as pragmatic as possible while still ensuring that surgeons adhered as closely as possible to the study protocol. Therefore, due to a lack of evidence definitively favouring a particular antibiotic regimen, key aspects of peri-operative care were recorded but not standardized.

Pre-Operative Care

The following pre-operative factors were recorded but not standardized:

1. Absolute neutrophil count prior to surgery; and
2. Neoadjuvant chemotherapy and radiotherapy administration.

Intra-Operative Care

The following intra-operative care factors were recorded but not standardized:

1. Antibiotic- or silver-coated implant use;
2. Antibiotic beads and/or antibiotic osteobiologics use;
3. Betadine soak use;
4. Fixation type;
5. Implant type;
6. Individual who performed the majority of surgery;
7. Irrigation use;
8. Laminar flow use;
9. Mode of skin closure;
10. Skin sterilization type;
11. Spacesuit use;
12. Suction drain use;
13. Thromboprophylaxis use; and
14. Tourniquet use.

Surgical Approach

Surgical excision and endoprosthetic reconstruction were performed according to the standard practice of the participating orthopaedic oncologists. This generally involved a wide extensile surgical exposure, isolation and protection of major neurovascular structures, resection of the segment of bone affected by the tumor with a two-to-three-centimeter bone margin, and a soft-tissue margin dictated by the amount of available tissue that could safely be resected from both oncological and functional standpoints. To ensure both feasibility and generalizability, we did not standardize the implants. Endoprostheses were implanted according to the manufacturer specific implant guides. Soft-tissue reconstruction may or may not have required tissue transfer based on the original extent of the tumor and required soft-tissue excision to establish wide oncological margins.

Post-Operative Care

Following the completion of their randomly allocated regimen of post-operative prophylactic intravenous antibiotics, patients could, at the surgeon's discretion, be continued on either intravenous or oral antibiotics.

The following post-operative care factors were recorded but not standardized:

1. Additional antibiotic administration;
2. Adjuvant chemotherapy and radiotherapy administration;
3. Duration until first wound dressing change;

© 2022 The PARITY Investigators. *JAMA Oncology*.

4. Negative pressure wound therapy (i.e., wound vacs) use (including duration);
5. Number of patients in hospital room;
6. Suction drain use (including duration);
7. Urinary catheter use (including duration); and
8. Thromboprophylaxis use.

© 2022 The PARITY Investigators. *JAMA Oncology*.

4.0 Investigational Pharmacy Procedures

The following information has been excerpted from the PARITY Pharmacy Manual, which documents the study-specific procedures for the local investigational pharmacies.

Randomization

Randomization was centralized through an internet-based, computer-generated randomization platform (www.randomize.net) that concealed allocation and utilized randomly permuted blocks of two or four. Only the investigational pharmacy at each clinical site had access to the randomization system, and an unblinded member of each investigational pharmacy team performed the peri-operative randomization.

Investigational Products

Inventory Management

Clinical sites used their own inventory to prepare the study antibiotics or placebo. In certain situations, the investigational products were segregated from the regular pharmacy inventory to prevent the unblinding of local research and clinical personnel, such as through the local institution's electronic medical record system.

Preparation

The preparation of the study antibiotics or placebo were conducted as per the relevant manufacturers' labels.

Study Antibiotics

The preparation of the study antibiotics was conducted as per local procedures and the relevant manufacturer's label. This generally involved using sterile techniques to add the appropriate diluents to the cefazolin vial for reconstitution according to the directions on the relevant Product Monograph. The contents of the vial were then swirled to allow the particles to dissolve. The appropriate dose of solution was then withdrawn and injected into a sodium chloride 0.9% 50mL intravenous bag. Alternatively, pre-mixed antibiotic bags could be used with no further need for manipulation.

Placebo

A sodium chloride 0.9% 50mL intravenous bag with no further manipulation was used as the placebo for patients randomized to the one-day regimen of post-operative prophylactic antibiotics.

Storage

The storage of the study antibiotics or placebo were conducted as per local procedures and the relevant manufacturers' labels. In general, reconstituted cefazolin and cefuroxime for injection or infusion could be stored for 24 hours at a controlled room temperature between 15-30°C, or for 72 hours under refrigeration (2 – 8°C), when protected from light.

Administration

The administration of the study antibiotics or placebo were conducted as per local procedures and the relevant manufacturers' labels. This typically involved the investigational products being administered over 30 minutes (15 – 60 minutes).

Blinding

To ensure the complete blinding of patients and caregivers, an unblinded member of the local investigational pharmacy prepared and shrouded using an opaque bag, or reconstituted in identical intravenous fluid bags, the randomly allocated study antibiotic or placebo solutions depending on the inventory available at each clinical site. The study antibiotics or placebo were properly labeled in accordance with local guidelines and all applicable regulations to ensure the safe administration and use while still maintaining adequate blinding.

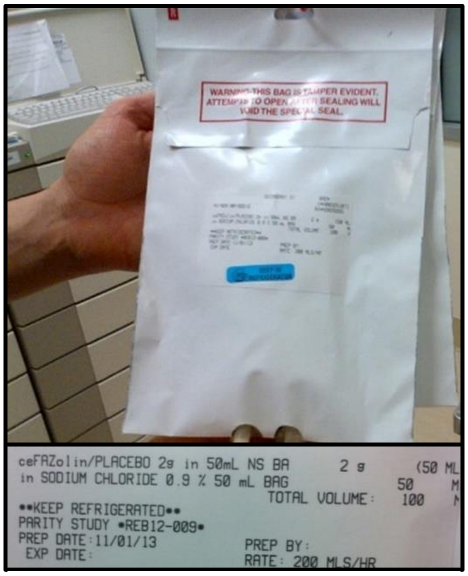

Example of Blinding of Study Antibiotics/Placebo

Medical Emergency Management

In the event of a medical emergency or infection that directly affects the health status of a study patient within the first five days post-surgery, the

following steps were taken by the local study team to ensure that no local study personnel were unblinded to the patient's treatment allocation:

1. The blinded study antibiotics or placebo were stopped;
2. The surgeon proceeded with treatment as per his/her usual standard practice; and
3. The discontinuation of the study antibiotics or placebo was documented on the relevant data collection forms.

The patient continued to be followed in the study under the intention-to-treat principle.

Unblinding

Unblinding requests were only to be considered if a patient:

1. Had an allergic reaction, and the surgeon needs to know the specific antibiotic regimen in order to inform treatment; or
2. Needed to be started on a drug that had the potential to interfere or interact with the study antibiotic.

In the event that one of the aforementioned situations occurred, the following steps were taken by the local study team to ensure that only the surgeon, as well as individuals directly involved in the patient's care, were unblinded to the patient's treatment allocation:

1. The local research personnel contacted the unblinded member of the Methods Center team and provided details of the medical emergency;
2. The unblinded member of the Methods Center team then contacted the study Principal Investigator to obtain permission to unblind the local investigator at the clinical site requesting the unblinding;
3. The Principal Investigator then reviewed the circumstances of the situation and approved the request; and
4. Once approved, the unblinded member of the Methods Center team contacted, via telephone, the local investigator to provide him/her with the patient's treatment allocation.

© 2022 The PARITY Investigators. *JAMA Oncology*.

5.0 PARITY Process Overview

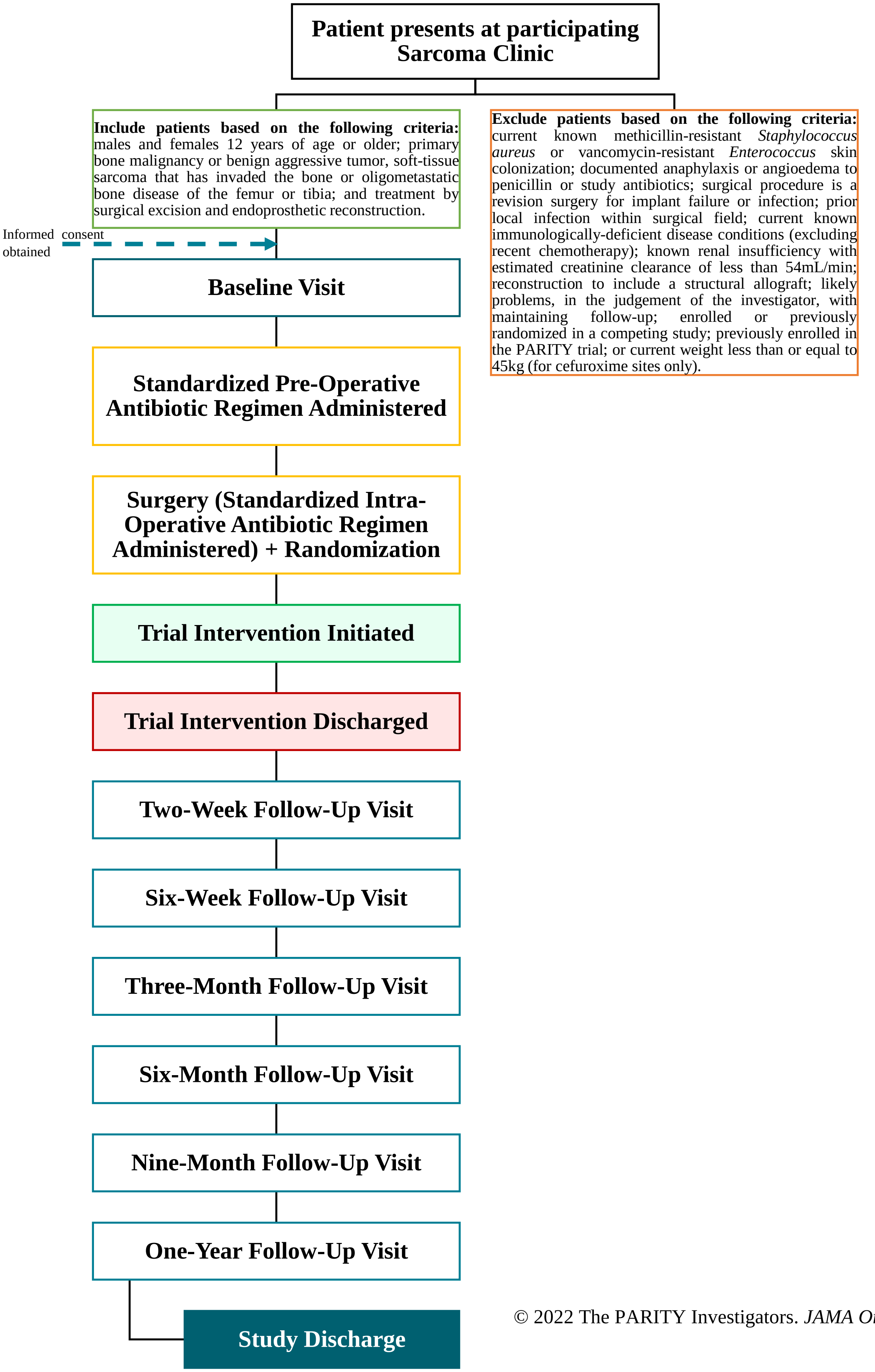

© 2022 The PARITY Investigators. *JAMA Oncology*.

Patients who presented to participating sarcoma clinics were screened for eligibility. Those who were eligible were approached to participate and informed consent was obtained. Baseline data was then obtained from consenting patients. On the day of surgery, the standardized pre- and intra-operative doses of prophylactic intravenous antibiotics were administered, limb salvage surgery was performed, and surgical data was collected. During the peri-operative period (immediately pre-operatively, intra-operatively or within 24 hours post-operatively), included patients were randomly allocated to one of the two trial interventions (a one- or five-day regimen of post-operative prophylactic intravenous antibiotics). The blinded trial intervention was discontinued on post-operative day five or at hospital discharge if acute hospital stay was less than five days. Included patients were assessed and monitored regularly for the primary outcome by their treating surgeon at two and six weeks, and three, six and nine months, and one year post-operatively. Other outcomes assessed at each study visit included antibiotic-related complications, adverse events, serious adverse events, re-operations, complications of wound healing, tumor recurrence or metastasis, and mortality. The Musculoskeletal Tumor Society (MSTS)-1987 and 1993 scores, as well as the Toronto Extremity Salvage Score (TESS), were completed prior to surgery, and at the three- and six-month, and one-year follow-up visits.

Timepoint	Assessment Procedures	Data Collection
Screening	In Person (Hospital/Clinic)	▪ Screening Form
Baseline Visit	In Person (Hospital/Clinic)	▪ Baseline Characteristics Form;<br>▪ Tumor Characteristics Form;<br>▪ MSTS-87 and MSTS-93 Questionnaires (clinician-administered); and<br>▪ TESS Questionnaire (self-administered)
Surgery and Peri-Operative Period	Not Applicable	▪ Randomization Form<br>▪ Surgical Report Form;<br>▪ Surgical Pathology Report Form;<br>▪ Peri-Operative Form; and<br>▪ Antibiotics Log
Two-Week Follow-Up Visit (1 to 3 weeks)	In Person (Hospital/Clinic) or Telephone	▪ Follow-Up Form*
Six-Week Follow-Up Visit (4 to 8 weeks)	In Person (Hospital/Clinic) or Telephone	▪ Follow-Up Form*
Three-Month Follow-Up Visit (2 to 4 months)	In Person (Hospital/Clinic) or Telephone	▪ Follow-Up Form*;<br>▪ MSTS-87 and MSTS-93 Questionnaires (clinician-administered); and<br>▪ TESS Questionnaire (self-administered)
Six-Month Follow-Up Visit (5 to 7 months)	In Person (Hospital/Clinic) or Telephone	▪ Follow-Up Form*;<br>▪ MSTS-87 and MSTS-93 Questionnaires (clinician-administered); and<br>▪ TESS Questionnaire (self-administered)
Nine-Month Follow-Up Visit (8 to 11 months)	In Person (Hospital/Clinic) or Telephone	▪ Follow-Up Form*
One-Year Follow-Up Visit (≥ 12 months)	In Person (Hospital/Clinic) or Telephone	▪ Follow-Up Form*;<br>▪ MSTS-87 and MSTS-93 Questionnaires (clinician-administered); and<br>▪ TESS Questionnaire (self-administered)

**In addition to the standardized Follow-Up Form that was to be completed at every study follow-up visit, the following other case report forms were completed as necessary: Tumor Site Infection Form, Protocol Deviation Form, Surgical Report Form: Re-Operations, Adverse Event Form, Antibiotics Log, Cultures Form.*

© 2022 The PARITY Investigators. *JAMA Oncology*.

6.0 Outcome Definitions

The primary outcome was the development of a surgical site infection within one year of the date of the initial limb-salvage surgery. Secondary outcomes included antibiotic-related complications, unplanned re-operations, death and oncologic and functional outcomes within one year after the initial limb-salvage surgery.

Primary Outcome

Surgical site infections (primary outcome) were classified according to the following definitions and criteria established by the Centers for Disease Control and Prevention.[6]

Classification	Definition/Criteria
Superficial incisional surgical site infection	An infection that occurs within the 30 days following the operative procedure and the infection involves only the skin or subcutaneous tissue of the incision. At least one of the following must also be present:<br>1. Purulent drainage, with or without laboratory confirmation, from the superficial incision;<br>2. Organisms isolated from an aseptically obtained culture of fluid or tissue from the superficial incision;<br>3. At least one of the following signs or symptoms of infection: pain/tenderness, localized swelling, redness or heat; or<br>4. Diagnosis of a superficial incisional surgical site infection by the surgeon or attending physician.
Deep incisional surgical site infection	An infection that occurs within the 30 days following the operative procedure or within one year if an implant is in place, the infection appears to be related to the operation, and the infection involves the deep soft tissue (e.g., fascial and muscle layers) of the incision. At least one of the following must also be present:<br>1. Purulent drainage from the deep incision but not from the organ space component of the surgical site;<br>2. A deep incision spontaneously dehisces or is deliberately opened by the surgeon when the patient has at least one of the following signs or symptoms: fever (>38ºC), localized pain or tenderness, unless the site culture is negative;<br>3. An abscess or other evidence of infection involving the deep incision that is found on direct examination, during reoperation, or by histopathologic or radiologic examination; or<br>4. Diagnosis of a deep incisional surgical site infection by a surgeon or attending physician.
Organ space surgical site infection	An infection that occurs within the 30 days following the operative procedure or within one year if an implant is in place, the infection appears to be related to the operation, and the infection involves any part of the anatomy (e.g., organs or spaces), other than the incision, which was opened or manipulated during the operation. At least one of the following must also be present:<br>1. Purulent drainage from a drain that is placed through a stab wound into organ space;<br>2. Organisms isolated from an aseptically obtained culture of fluid or tissue in the organ space;<br>3. An abscess or other evidence of infection involving the organ space that is found on direct examination, during reoperation, or by histopathologic or radiologic examination; or<br>4. Diagnosis of an organ space surgical site infection by a surgeon or attending physician.

© 2022 The PARITY Investigators. *JAMA Oncology*.

Secondary Outcomes

Outcome	Definition
Antibiotic-Related Complications	Possible antibiotic-related complications diagnosed by physicians at clinical sites, including: *Clostridioides difficile* associated colitis, toxic megacolon, opportunistic fungal infection, indwelling catheter-related sepsis, seizures, stomach cramps, nausea or vomiting, oral candidiasis, vaginal thrush, unusual bleeding or bruising, difficulty breathing, sore mouth or throat, allergic reaction, anemia and/or low blood counts, skin reaction, diarrhea, liver toxicity, kidney toxicity or other (specify).
Unplanned Re-Operations	Any unplanned re-operations at the surgical site after the initial limb-salvage surgery, including (but not limited to): abductor reconstruction, amputation, antibiotic spacer insertion, bone graft, extensor mechanism reconstruction, fasciotomy, implant exchange, implant revision, irrigation and debridement, patellar resurfacing, repeat tumor excision, rotationplasty, skin graft, wound flap or other (specify).<br><br>Secondary procedures that were planned at the onset of the initial limb-salvage surgery were not considered study events.
Oncologic Events	Reported oncologic events, including local recurrence or distant metastases, as diagnosed by physicians at clinical sites.
Mortality	Reported deaths if they occurred within one year of the initial surgery.
Functional Outcomes	Functional outcomes were measured using physician-administered and patient-administered questionnaires, which were completed at baseline as well as the three- and six-month, and one-year, follow-up visits.<br><br>The Musculoskeletal Tumor Society (MSTS)-87 score is a standardized scoring system that is completed by an individual on the treatment team (preferably the treating surgeon) and measures physical function after treatment for a musculoskeletal tumor across seven domains: motion, pain, stability, deformity, muscular strength, functional activity and emotional acceptance. Each domain consists of seven questions and is scored separately from zero (lowest) to five (highest), for a maximum total of 35. Therefore, a higher score indicates better function.<br><br>The MSTS-93 score is a standardized scoring system that is also completed by an individual on the treatment team and measures functional outcome after treatment for a musculoskeletal tumor across six domains: pain, function, emotional acceptance, support, walking ability and gait. Each domain consists of six questions and is scored separately from zero (lowest) to five (highest), for a maximum total of 30. This is then converted into a percentage, with a higher score indicating better function.<br><br>The Toronto Extremity Salvage Score (TESS) is a self-administered evaluation tool that was developed to assess physical function and quality-of-life in patients that have undergone limb salvage surgery for tumors of the extremities. The lower extremity portion of the survey contains 30 questions that are framed to ask about the difficulty experienced by the patient in performing each activity over the previous week. Each question uses a Likert scale, scored from one to five and consisting of the following responses: impossible (1); extremely difficult (2); moderately difficult (3); a little bit difficult (4); or not at all difficult (5). The maximum total of 150 is then converted into a score out of 100. Therefore, a higher score indicates better function.

© 2022 The PARITY Investigators. *JAMA Oncology*.

7.0 Adjudication

The following information has been excerpted from the PARITY Adjudication Charter, which documents the responsibilities of the Central Adjudication Committee and the adjudication processes for the PARITY trial.

Overview

The Adjudication Committee was comprised of the study Principal Investigator, who served as the committee's Chair, an infectious diseases specialist and three other orthopaedic oncologists. Each patient requiring adjudication was reviewed by the Adjudication Committee Chair, the infectious diseases specialist and at least one other member who specialized in orthopaedic oncology. As a result, all instances of possible antibiotic-related complications and surgical site infections were independently reviewed by the infectious diseases specialist, and all additional surgical procedures were independently reviewed by at least two orthopaedic oncologists.

Adjudication took place only after each patient had completed his/her final study visit (One-Year or Early Withdrawal Follow-Up Visit). To do so, the Adjudication Committee reviewed all relevant and available clinical notes, digital images/photographs, post-initial surgery radiographs and case report forms. The Adjudication Committee was blinded to treatment allocation. The committee was also blinded to the participating clinical site as all site identifiers were removed, including the participating institution and surgeon's name and study site identification number.

Case Eligibility

The Adjudication Committee confirmed case eligibility only for those randomized patients whose eligibility was in doubt. To minimize random error, the Adjudication Committee blindly adjudicated trial eligibility based on data available just before or shortly after randomization. Patients that did not meet all inclusion criteria, or met one of the exclusion criteria, were deemed ineligible.

Surgical Site Infections

The Adjudication Committee adjudicated all reported possible surgical site infections that occurred within one year of the initial surgery to determine if they were study events. Surgical site infections were classified according to the criteria established by the Centers for Disease Control and Prevention (see Appendix S2.4 above). The following clinical events were reviewed as possible surgical site infections:

- Surgical site infection (superficial incisional, deep incisional and organ space);
- Aseptic loosening;
- Cellulitis;
- Sepsis; and
- Wound healing problems (including wound dehiscence and wound necrosis).

Antibiotic-Related Complications

The Adjudication Committee adjudicated all reported possible antibiotic-related complications that occurred within one year of the initial surgery to determine if they were study events. The following clinical events were reviewed as possible antibiotic-related complications:

- Allergic reaction;
- Anemia and/or low blood counts;
- *Clostridioides difficile* associated colitis;
- Diarrhea;
- Difficulty breathing;
- Indwelling catheter-related sepsis;
- Kidney toxicity;
- Liver toxicity;
- Nausea or vomiting;
- Opportunistic fungal infection;
- Oral candidiasis;
- Seizures;
- Skin reaction;
- Sore mouth or throat;
- Stomach cramps;
- Toxic megacolon;
- Unusual bleeding or bruising; and
- Vaginal thrush.

© 2022 The PARITY Investigators. *JAMA Oncology*.

The Adjudication Committee also reached a consensus on the relation of the complication to the study antibiotics.

Unplanned Re-Operations

The Adjudication Committee adjudicated all reported additional surgical procedures that occurred within one year of initial surgery at the initial surgical site to determine if they were study events. Planned additional surgeries were not considered study events. For those patients who had multiple unplanned additional surgeries for one indication, each unplanned revision surgery was considered a study event. Any unplanned revision surgery after the initial limb salvage surgery that was intended to treat or manage at least one of the clinical events was considered a study event:

- Abductor failure;
- Aseptic loosening;
- Compartment syndrome;
- Extensor mechanism failure;
- Hardware failure;
- Hematoma;
- Implant malpositioning;
- Joint effusion;
- Joint instability or dislocation;
- Leg lengthening (including leg length discrepancy after initial surgery);
- Local recurrence;
- Neuropathic pain;
- Patellar degeneration;
- Peri-prosthetic fracture;
- Positive margin after initial surgery;
- Soft-tissue coverage;
- Stiffness/limited range of motion (including arthrofibrosis or contracture);
- Surgical site infection (surgical incisional)
- Surgical site infection (deep incisional or organ space);
- Traumatic wound opening;
- Vascular compromise; and
- Wound healing problem (such as wound dehiscence or wound necrosis).

Any unplanned surgery to implant the tumor endoprosthesis after the initial surgery was aborted due to patient instability were also considered study events.

Mortality

The Adjudication Committee adjudicated mortality, as necessary, following a patient’s early withdrawal to confirm the cause of death.

© 2022 The PARITY Investigators. *JAMA Oncology*.

8.0 Interpretation of Blinded Data

Purpose

The purpose of this document is to outline the interpretations of the blinded study data of the primary analyses for the primary outcome, sub-group, secondary outcomes and sensitivity analyses for the Prophylactic Antibiotic Regimens In Tumor Surgery (PARITY) trial. Blinded data interpretation may decrease the frequency of misleading data interpretations. Details of the analyses conducted can be found in the PARITY Statistical Analysis Plan.[7]

Here, we present alternative interpretations of blinded preliminary results from the PARITY trial. These data are representative from June 22, 2021 as below, and includes 604 patients in the primary analysis.

	Number of Patients		
	**Total**	**Group A**	**Group B**
Total Number of Enrolled Patients	611	312	299
**Total Included in Primary Analyses**	604	311	293

Hypothesis

We hypothesize that a five-day regimen of post-operative prophylactic intravenous antibiotics will result in fewer surgical site infections at one year.

Rationale for Hypothesized Direction

In a meta-analysis of data from retrospective studies, the surgical site infection rate following endoprosthetic reconstruction of the lower extremity in 4,838 patients was 10% (95% confidence interval [CI], 8% - 11%).[8] The pooled retrospective data suggested that long-term antibiotic prophylaxis decreases the risk of deep surgical site infection (8% versus 13%).

Primary Outcome Analysis

Outcome	Group A n=311	Group B n=293	Total n=604	Hazard Ratio (95% CI)	P-value
Any surgical site infection	52 (16.7)	44 (15.0)	96 (15.9)	1.08 (0.71, 1.62)	0.730
Superficial incisional	12 (3.9)	13 (4.4)	25 (4.1)		
Deep incisional	8 (2.6)	3 (1.0)	11 (1.8)		
Organ space	34 (10.9)	28 (9.6)	62 (10.3)	1.03 (0.62, 1.71)	0.916

Blinded Interpretations

Results Fail to Establish Any Difference Between Post-Operative Antibiotic Treatment Groups

If Group A is the Longer Duration of Post-Operative Prophylactic Antibiotics: The findings of this trial fail to demonstrate **a convincing difference** between the two treatment groups in the risk of developing a surgical site infection. The findings of this trial do not, however, exclude an important difference between the two treatment groups favoring either the short or long antibiotic duration group.

If Group B is the Longer Duration of Post-Operative Prophylactic Antibiotics: The findings of this trial fail to demonstrate **a convincing difference** between the two treatment groups in the risk of developing a surgical site infection. The findings of this trial do not, however, exclude an important difference between the two treatment groups and, in particular, do not exclude an important benefit for the long duration of post-operative prophylactic antibiotics.

Subgroup Analyses

At the onset of the PARITY trial, we identified two important subgroups (tumor type and tumor location). As we neared the end of the trial, prior to unblinding, we identified a further three important subgroups (sex, age and pre-operative chemotherapy). We added a main effect for the subgroup variable and the treatment by subgroup interaction to our primary analysis model to assess whether the magnitude of the treatment effect was significantly different between subgroups. This was repeated separately for each subgroup variable. We performed these subgroup analyses with the primary endpoint as the outcome. If a statistically significant subgroup effect was found, we further explored the impact of the subgroup on the secondary outcomes.

Subgroup	Hypothesis	Rationale for Hypothesized Direction	If Hypothesis Not Supported
Tumor Type	We hypothesize that there will be no difference between	The risk of developing a surgical site infection is not	Possible bias existed in previous studies with

© 2022 The PARITY Investigators. *JAMA Oncology*.

	the tumor types regarding the association between prophylactic antibiotic duration and risk of surgical site infection.	known to be different between tumor types.[9]	uncontrolled groups that directed the rationale for the hypothesis.
Tumor Location	We hypothesize that a five-day regimen of prophylactic antibiotics will be more effective relative to a one-day regimen in tibial reconstructions than in femoral reconstructions.	Tibial reconstruction is a known risk factor for developing a surgical site infection after limb salvage surgery, likely owing to issues with soft-tissue coverage after reconstructions at this anatomical location.[9–11]	Other factors may play a role in the increased surgical site infection rates after tibial reconstructions, such as soft-tissue coverage or (possibly) tumor size.
Sex	We hypothesize that there will be no difference between the sexes regarding the association between prophylactic antibiotic duration and risk for surgical site infection.	Sarcoma does not have a sex predilection and the risk of developing a surgical site infection is not known to be different across sexes.[9]	Possible bias existed in previous studies with uncontrolled groups that directed the rationale for the hypothesis.
Age	We hypothesize that a five-day regimen of prophylactic antibiotics will be more effective relative to a one-day regimen in the older adult population than in the pediatric and young adult population.	There is some evidence demonstrating that advanced age is a risk factor for developing a surgical site infection after limb salvage surgery, likely owing to decreased function of the immune system.[12]	Other factors may play a role in the increased surgical site infection rates in the older adult population, such as comorbidities and slower healing rates.
Pre-Operative Chemotherapy	We hypothesize that a five-day regimen of prophylactic antibiotics will be more effective relative to a one-day regimen in patients who received pre-operative chemotherapy than in those who did not receive pre-operative chemotherapy.	There is some evidence demonstrating that the administration of pre-operative chemotherapy is a risk factor for developing a surgical site infection after limb salvage surgery, likely owing to its immunosuppressive properties.[13,14]	Other factors may play a role in the increased surgical site infection rates in patients treated with pre-operative chemotherapy, such as bone marrow suppression and prolonged hospital stays.

© 2022 The PARITY Investigators. *JAMA Oncology*.

9.0 Supplementary Tables

eTable 1: Reasons for Exclusion Prior to Randomization

Eligibility Criterion	Total<br>n=252
Less than 12 years of age, n(%)	14 (5.6%)
Does not have either: A) a primary bone malignancy or benign aggressive tumor of the femur or tibia; B) a soft-tissue sarcoma that has invaded the bone of the femur or tibia; or C) oligometastatic bone disease of the femur or tibia and is expected to live one year post-operatively, n(%)	23 (9.1%)
Unsuitable for treatment by surgical excision and endoprosthetic reconstruction, n(%)	32 (12.7%)
Did not provide informed consent, n(%)	100 (39.7%)
Skin is currently known to be colonized with MRSA or VRE, n(%)	1 (0.4%)
Documented anaphylactic or angioedema reaction to penicillin or study antibiotics, n(%)	7 (2.7%)
Planned surgical procedure is a revision surgery for implant failure or infection, n(%)	20 (7.9%)
Prior local infection within the surgical field, n(%)	10 (4.0%)
Currently known to have an immunologically-deficient disease condition, n(%)	7 (2.8%)
Known renal insufficiency with an eGRF < 54 mL/min, n(%)	5 (2.0%)
Planned reconstruction to include a structural allograft, n(%)	9 (3.6%)
Enrolled or previously randomized in a competing study, n(%)	0 (0.0%)
Previously enrolled in the PARITY trial, n(%)	1 (0.4%)
Problems, in the judgment of the investigator, with maintaining follow-up, n(%)	20 (7.9%)
Any other reason for exclusion [surgeon discretion], n(%)	3 (1.2%)

MRSA = Methicillin-resistant *Staphylococcus aureus*; VRE = Vancomycin-resistant *Enterococcus*; eGFR = Estimated glomerular filtration rate

© 2022 The PARITY Investigators. *JAMA Oncology*.

eTable 2: Reasons for Exclusion After Adjudication Committee Review

Eligibility Criterion	Five-Day Regimen	One-Day Regimen
Less than 12 years of age	0	0
Does not have either: A) a primary bone malignancy or benign aggressive tumor of the femur or tibia; B) a soft-tissue sarcoma that has invaded the bone of the femur or tibia; or C) oligometastatic bone disease of the femur or tibia and is expected to live one year post-operatively	0	0
Unsuitable for treatment by surgical excision and endoprosthetic reconstruction	1	1
Did not provide informed consent	2	0
Skin is currently known to be colonized with MRSA or VRE	1	0
Documented anaphylactic or angioedema reaction to penicillin or study antibiotics	0	0
Planned surgical procedure is a revision surgery for implant failure or infection	0	0
Prior local infection within the surgical field	1	0
Currently known to have an immunologically-deficient disease condition	1	0
Known renal insufficiency with an eGRF < 54 mL/min	0	0
Planned reconstruction to include a structural allograft	0	0
Enrolled or previously randomized in a competing study	0	0
Previously enrolled in the PARITY trial	0	0
Problems, in the judgment of the investigator, with maintaining follow-up	0	0
Any other reason for exclusion [surgeon discretion]	0	0

MRSA = Methicillin-resistant *Staphylococcus aureus*; VRE = Vancomycin-resistant *Enterococcus*; eGFR = Estimated glomerular filtration rate

© 2022 The PARITY Investigators. *JAMA Oncology*.

eTable 3: Patient Demographics and Baseline Details

Characteristic	Five-Day Regimen n=293	One-Day Regimen n=311	Total n=604
Age in years, mean (SD)	42.6 (21.7)	39.9 (22.0)	41.2 (21.9)
Females, n (%)	115 (39.2)	128 (41.2)	243 (40.2)
Ethnicity, n (%)	n=293	n=309	n=602
Native	4 (1.4)	11 (3.6)	15 (2.5)
Asian	53 (18.1)	60 (19.4)	113 (18.8)
Black	21 (7.2)	22 (7.1)	43 (7.1)
Hispanic	14 (4.8)	20 (6.5)	34 (5.6)
White	194 (66.2)	190 (61.5)	384 (63.8)
Other	7 (2.4)	6 (1.9)	13 (2.2)
Employed pre-diagnosis, n (%)	n=290	n=310	n=600
Yes	128 (44.1)	111 (35.8)	239 (39.8)
No	162 (55.9)	199 (64.2)	361 (60.2)
Retired	47 (16.2)	54 (17.4)	101 (16.8)
Doctor's advice/disability	6 (2.1)	8 (2.6)	14 (2.3)
Unemployed	6 (2.1)	18 (5.8)	24 (4.0)
Homemaker	10 (3.4)	12 (3.9)	22 (3.7)
Student	90 (31.0)	107 (34.5)	197 (32.8)
Other	1 (0.3)	0 (0.0)	1 (0.2)
Unknown	2 (0.7)	0 (0.0)	2 (0.3)
Other known malignancies, n (%)	1 (0.3)	6 (1.9)	7 (1.2)
Systemic metastases, n (%)			
No	244 (83.3)	255 (82.0)	499 (82.6)
Yes	49 (16.7)	56 (18.0)	105 (17.4)
Pulmonary	25 (8.5)	29 (9.3)	54 (8.9)
Skeletal	33 (11.3)	32 (10.3)	65 (10.8)
Other viscera	2 (0.7)	7 (2.3)	9 (1.5)
Other	3 (1.0)	6 (1.9)	9 (1.5)
Other cancer treatment modalities, n (%)			
No	157 (53.6)	138 (44.4)	295 (48.8)
Yes	136 (46.4)	173 (55.6)	309 (51.2)
Pre-operative chemotherapy	129 (44.0)	161 (51.8)	290 (48.0)
Pre-operative radiation	10 (3.4)	12 (3.9)	22 (3.6)
Other	7 (2.4)	7 (2.3)	14 (2.3)
Smoking status, n (%)			
Never smoked	211 (72.0)	239 (76.8)	450 (74.5)
Current smoker	34 (11.6)	26 (8.4)	60 (9.9)
Former smoker	48 (16.4)	46 (14.8)	94 (15.6)
Alcohol use, n (%)			
No	194 (66.2)	222 (71.4)	416 (68.9)
Yes	99 (33.8)	89 (28.6)	188 (31.1)
Recreational IV drug use, n (%)			
No	291 (99.3)	311 (100.0)	602 (99.7)
Yes	2 (0.7)	0 (0.0)	2 (0.3)
Diabetic, n (%)			
No	273 (93.2)	287 (92.3)	560 (92.7)
Yes	20 (6.8)	24 (7.7)	44 (7.3)
Insulin-dependent	4 (1.4)	6 (1.9)	10 (1.7)
Not insulin-dependent	16 (5.5)	18 (5.8)	34 (5.6)
Medication use, n (%)			
NSAIDs	128 (43.7)	137 (44.1)	265 (43.9)
Opioids	94 (32.1)	76 (24.4)	170 (28.1)

© 2022 The PARITY Investigators. *JAMA Oncology*.

Characteristic	Five-Day Regimen n=293	One-Day Regimen n=311	Total n=604
Anti-hypertension medications	39 (13.3)	42 (13.5)	81 (13.4)
Cardiac medications	21 (7.2)	19 (6.1)	40 (6.6)
Pulmonary medications	6 (2.0)	7 (2.3)	13 (2.2)
Osteoporosis medications	3 (1.0)	5 (1.6)	8 (1.3)
Antibiotics	4 (1.4)	7 (2.3)	11 (1.8)
None of the above	95 (32.4)	117 (37.6)	212 (35.1)
Neutropenic at time of surgery*, n (%)	n=275	n=286	n=561
No	231 (84.0)	234 (81.8)	465 (82.9)
Yes	44 (16.0)	52 (18.2)	96 (17.1)

SD = Standard deviation

*Absolute neutrophil count ≤1500/mm^3

© 2022 The PARITY Investigators. *JAMA Oncology*.

eTable 4: Tumor Details

Characteristic	Five-Day Regimen n=293	One-Day Regimen n=311	Total n=604
Location of tumor, n (%)			
Tibia	53 (18.1)	55 (17.7)	108 (17.9)
Femur	240 (81.9)	256 (82.3)	496 (82.1)
Location in bone, n (%)			
Proximal	135 (46.1)	134 (43.1)	269 (44.5)
Mid-shaft	26 (8.9)	14 (4.5)	40 (6.6)
Distal	165 (56.3)	178 (57.2)	343 (56.8)
Other	3 (1.0)	1 (0.3)	4 (0.7)
Maximum dimension in centimeters,	n=204	n=227	n=431
mean (SD)			
median (Q1-Q3)	9.5 (6.0)	10.1 (5.4)	9.8 (5.7)
total range	8 (5.2-11.85)	9.5 (6.3-13)	9 (6-12.7)
	0.7-43.4	1-34	0.7-43.4
Number of compartments, n (%)	n=204	n=227	n=431
0	3 (1.5)	2 (0.9)	5 (1.2)
1	67 (32.8)	72 (31.7)	139 (32.3)
2	79 (38.7)	82 (36.1)	161 (37.4)
3	40 (19.6)	53 (23.3)	93 (21.6)
4	15 (7.4)	18 (7.9)	33 (7.7)
Type of biopsy performed, n (%)	n=292	n=310	n=602
Open	104 (35.6)	117 (37.7)	221 (36.7)
Fine-needle aspiration	3 (1.0)	3 (1.0)	6 (1.0)
Core needle	164 (56.2)	161 (51.9)	325 (54.0)
None	21 (7.2)	29 (9.4)	50 (8.3)
Type of tumor, n (%)			
Bone sarcoma	237 (80.9)	249 (80.1)	486 (80.5)
Soft tissue sarcoma	28 (9.6)	34 (10.9)	62 (10.3)
Oligometastatic bone disease	28 (9.6)	28 (9.0)	56 (9.3)
Overall margins, n (%)	n=290	n=308	n=598
Negative	264 (91.0)	283 (91.9)	547 (91.5)
Microscopically positive	17 (5.9)	16 (5.2)	33 (5.5)
Grossly positive	9 (3.1)	9 (2.9)	18 (3.0)

SD = Standard deviation; Q1-Q3 = Quartile 1 to quartile 3

© 2022 The PARITY Investigators. *JAMA Oncology*.

eTable 5: Surgical and Peri-Operative Management Details

Characteristic	Five-Day Regimen n=293	One-Day Regimen n=311	Total n=604
Surgical Details			
Length of procedure in minutes, median (Q1-Q3)	270 (206-377)	270 (200-377)	270 (205-377)
Type of skin sterilization, n (%)			
Iodine	72 (24.6)	80 (25.7)	152 (25.2)
Alcohol	82 (28.0)	92 (29.6)	174 (28.8)
Chlorhexidine	239 (81.6)	251 (80.7)	490 (81.1)
Length of incision in centimeters, mean (SD)	n=284<br>31.4 (11.1)	n=299<br>29.7 (9.8)	n=583<br>30.5 (10.5)
Laminar flow, n (%)			
Yes	113 (38.6)	121 (38.9)	234 (38.7)
No	180 (61.4)	190 (61.1)	370 (61.3)
Spacesuit worn, n (%)			
Yes	114 (38.9)	129 (41.5)	243 (40.2)
No	179 (61.1)	182 (58.5)	361 (59.8)
Tourniquet used, n (%)	n=292	n=311	n=603
Yes	112 (38.4)	118 (37.9)	230 (38.1)
No	180 (61.6)	193 (62.1)	373 (61.9)
Type of resection, n (%)	n=292	n=309	n=601
Intra-articular	251 (86.0)	249 (80.6)	500 (83.2)
Extra-articular	41 (14.0)	60 (19.4)	101 (16.8)
Length of bone resected, n (%)	n=293	n=310	n=603
< 5 cm	4 (1.4)	6 (1.9)	10 (1.7)
5 – 10 cm	30 (10.2)	32 (10.3)	62 (10.3)
> 10 cm	259 (88.4)	272 (87.7)	531 (88.1)
Skin excised, n (%)	n=292	n=309	n=601
None	115 (39.4)	138 (44.7)	253 (42.1)
Small amount	130 (44.5)	123 (39.8)	253 (42.1)
Moderate amount	34 (11.6)	30 (9.7)	64 (10.6)
Large amount	13 (4.5)	18 (5.8)	31 (5.2)
Muscle excised, n (%)	n=291	n=308	n=599
None	30 (10.3)	34 (11.0)	64 (10.7)
Small amount	138 (47.4)	148 (48.1)	286 (47.7)
Moderate amount	76 (26.1)	80 (26.0)	156 (26.0)
Large amount	47 (16.2)	46 (14.9)	93 (15.5)
Fascial tissue excised, n (%)	n=292	n=309	n=601
None	77 (26.4)	73 (23.6)	150 (25.0)
Small amount	88 (30.1)	94 (30.4)	182 (30.3)
Moderate amount	73 (25.0)	86 (27.8)	159 (26.5)
Large amount	54 (18.5)	56 (18.1)	110 (18.3)
Type of fixation, n (%)	n=293	n=309	n=602
Press-fit	95 (32.4)	110 (35.6)	205 (34.1)
Cement	234 (79.9)	234 (75.7)	468 (77.7)
With Antibiotic	178 (60.8)	169 (54.7)	347 (57.6)
Without Antibiotic	56 (19.1)	64 (20.7)	120 (19.9)
Missing	0 (0.0)	1 (0.3)	1 (0.2)
Cerclage	22 (7.5)	25 (8.1)	47 (7.8)
Wire	13 (4.4)	10 (3.2)	23 (3.8)
Cable	8 (2.7)	14 (4.5)	22 (3.7)
Synthetic	1 (0.3)	1 (0.3)	2 (0.3)
Bone grafting performed, n (%)	n=292	n=311	n=603
No	280 (95.9)	296 (95.2)	576 (95.5)

© 2022 The PARITY Investigators. *JAMA Oncology*.

Characteristic	Five-Day Regimen<br>n=293	One-Day Regimen<br>n=311	Total<br>n=604
Yes	12 (4.1)	15 (4.8)	27 (4.5)
Synthetic bone graft	0 (0.0)	0 (0.0)	0 (0.0)
Autograft	11 (3.8)	12 (3.9)	23 (3.8)
Cortical	0 (0.0)	0 (0.0)	0 (0.0)
Cancellous	11 (3.8)	11 (3.5)	22 (3.6)
Vascularized Cortical	0 (0.0)	1 (0.3)	1 (0.2)
Allograft	1 (0.3)	3 (1.0)	4 (0.7)
Cortical	0 (0.0)	1 (0.3)	1 (0.2)
Cancellous	1 (0.3)	2 (0.6)	3 (0.5)
Vascular reconstruction, n (%)	n=292	n=311	n=603
No	287 (98.3)	308 (99.0)	595 (98.7)
Yes	5 (1.7)	3 (1.0)	8 (1.3)
<5 cm	2 (0.7)	1 (0.3)	3 (0.5)
5-10 cm	2 (0.7)	1 (0.3)	3 (0.5)
>10 cm	0 (0.0)	1 (0.3)	1 (0.2)
Missing	1 (0.3)	0 (0.0)	1 (0.2)
Intra-operative thromboprophylaxis, n (%)			
No	203 (69.3)	207 (66.6)	410 (67.9)
Yes	90 (30.7)	104 (33.4)	194 (32.1)
IV heparin	2 (0.7)	5 (1.6)	7 (1.2)
Tranexamic acid	77 (26.3)	88 (28.3)	165 (27.3)
Other	11 (3.8)	11 (3.5)	22 (3.6)
Antibiotic or silver-coated prosthesis, n (%)	n=292	n=311	n=603
No	276 (94.5)	295 (94.9)	571 (94.7)
Yes	16 (5.5)	16 (5.1)	32 (5.3)
Antibiotic	6 (2.1)	6 (1.9)	12 (2.0)
Silver	10 (3.4)	10 (3.2)	20 (3.3)
Antibiotic impregnated sponge or antibiotic powder implanted, n (%)	n=291	n=311	n=602
No			
Yes	230 (79.0)	248 (79.7)	478 (79.4)
Gentamicin	61 (21.0)	63 (20.3)	124 (20.6)
Tobramycin	6 (2.1)	5 (1.6)	11 (1.8)
Cefazolin	0 (0.0)	2 (0.6)	2 (0.3)
Vancomycin	0 (0.0)	1 (0.3)	1 (0.2)
Other	55 (18.9)	55 (17.7)	110 (18.3)
	1 (0.3)	0 (0.0)	1 (0.2)
Irrigation performed at end of procedure, n (%)	n=292	n=310	n=602
No	5 (1.7)	5 (1.6)	10 (1.7)
Yes	287 (98.3)	305 (98.4)	592 (98.3)
Pulsed irrigation			
Yes	245 (83.9)	258 (83.2)	503 (83.6)
No	40 (13.7)	46 (14.8)	86 (14.3)
Missing	2 (0.7)	1 (0.3)	3 (0.5)
Antibiotics in irrigation			
Yes	29 (9.9)	40 (12.9)	69 (11.5)
No	255 (87.3)	262 (84.5)	517 (85.9)
Missing	3 (1.0)	3 (1.0)	6 (1.0)
Mode of skin closure, n (%)			
Primary closure	265 (90.4)	286 (92.0)	551 (91.2)

© 2022 The PARITY Investigators. *JAMA Oncology*.

Characteristic	Five-Day Regimen n=293	One-Day Regimen n=311	Total n=604
Local muscle flap and split thickness skin graft	32 (10.9)	31 (10.0)	63 (10.4)
Local fasciocutaneous flap	7 (2.4)	5 (1.6)	12 (2.0)
Free flap	4 (1.4)	5 (1.6)	9 (1.5)
Peri-Operative Management Details			
Post-operative thromboprophylaxis, n (%)			
No	89 (30.4)	86 (27.7)	175 (29.0)
Yes	204 (69.6)	225 (72.3)	429 (71.0)
Coumadin	5 (1.7)	8 (2.6)	13 (2.2)
Fractionated heparin	137 (46.8)	153 (49.2)	290 (48.0)
Heparin	27 (9.2)	27 (8.7)	54 (8.9)
Oral	35 (11.9)	37 (11.9)	72 (11.9)
Suction drain, n (%)	n=293	n=310	n=603
No	63 (21.5)	74 (23.9)	137 (22.7)
Yes	230 (78.5)	236 (76.1)	466 (77.3)
Suction drain duration in days, median (Q1-Q3)	n=227<br>4 (3-5)	n=234<br>4 (3-6)	n=461<br>4 (3-5)
Urinary catheter, n (%)			
No	26 (8.9)	26 (8.4)	52 (8.6)
Yes	267 (91.1)	285 (91.6)	552 (91.4)
Urinary catheter duration in days, median (Q1-Q3)	n=265<br>2 (1-4)	n=284<br>2 (1-3.5)	n=549<br>2 (1-4)
Number of patients in hospital room, n (%)	n=285	n=306	n=591
1	109 (38.2)	132 (43.1)	241 (40.8)
2	88 (30.9)	90 (29.4)	178 (30.1)
3	11 (3.9)	10 (3.3)	21 (3.6)
4	35 (12.3)	29 (9.5)	64 (10.8)
> 4	42 (14.7)	45 (14.7)	87 (14.7)
Days to first post-operative wound dressing change, median (Q1-Q3)	n=285<br>3 (2-5)	n=303<br>3 (2-5)	n=588<br>3 (2-5)
Negative pressure wound therapy (wound vac), n (%)	n=292	n=311	n=603
No	244 (83.6)	276 (88.7)	520 (86.2)
Yes	48 (16.4)	35 (11.3)	83 (13.8)
Duration of wound vac in days, median (Q1-Q3)	n=48<br>6 (5-8.5)	n=35<br>5 (4-7)	n=83<br>6 (4-8)
Length of post-operative hospital stay in days, median (Q1-Q3)	n=292<br>6 (5-9)	n=311<br>6 (4-8)	n=603<br>6 (5-8)
Discharge location	n=292	n=311	n=603
Died	2 (0.7)	4 (1.3)	6 (1.0)
Home	222 (76.0)	247 (79.4)	469 (77.8)
Rehabilitation facility	52 (17.8)	45 (14.5)	97 (16.1)
Other hospital	13 (4.5)	11 (3.5)	24 (4.0)
Other	3 (1.0)	4 (1.3)	7 (1.2)
Adjuvant chemotherapy, n (%)	n=288	n=309	n=597
No	131 (45.5)	129 (41.7)	260 (43.6)
Yes	157 (54.5)	180 (58.3)	337 (56.4)

Q1-Q3 = Quartile 1 to quartile 3; SD = Standard deviation

© 2022 The PARITY Investigators. *JAMA Oncology*.

eTable 6: Prophylactic Antibiotic Administration Details

Detail	Five-Day Regimen n=293	One-Day Regimen n=311
Pre-operative study antibiotic administered per protocol, n (%)		
Yes	276 (94.2)	299 (96.1)
No	17 (5.8)	12 (3.9)
Additional pre-operative prophylactic antibiotics administered, n (%)		
Yes	11 (3.8)	11 (3.5)
No	282 (96.2)	300 (96.5)
Intra-operative study antibiotic administered per protocol, n (%)		
Yes	274 (93.5)	304 (97.7)
No	19 (6.5)	7 (2.3)
Additional intra-operative prophylactic antibiotics administered, n (%)		
Yes	5 (1.7)	1 (0.3)
No	288 (98.3)	310 (99.7)
Post-operative study antibiotic administered per protocol, n (%)		
Yes	180 (61.4)	188 (60.5)
No	113 (38.6)	123 (39.5)
Additional post-operative prophylactic antibiotics administered, n (%)	n=293	n=310
Yes	45 (15.4)	52 (16.8)
No	248 (84.6)	258 (83.2)
All study antibiotics administered per protocol, n (%)		
Yes	170 (58.0)	183 (58.8)
No	123 (42.0)	128 (41.2)

© 2022 The PARITY Investigators. *JAMA Oncology*.

eTable 7: Sensitivity Analyses

Outcome	Five-Day Regimen n=293	One-Day Regimen n=311	Hazard Ratio (95% CI)	P-value
Competing Risks Analysis*				
Any surgical site infection (primary outcome)	44 (15.0)	52 (16.7)	0.92 (0.62, 1.35)	0.654
Seventy-seven patients died and 20 had an amputation, for a total of 90 with either amputation or death. Twenty-eight of these had a surgical site infection prior to amputation/death. Therefore, 62 (eight amputations and 58 deaths) are competing events.				
Center-Effects†				
Any surgical site infection (primary outcome)	44 (15.0)	52 (16.7)	0.92 (0.62, 1.38)	0.696
Adjusted Analyses‡				
Any surgical site infection (primary outcome)	44 (15.0)	52 (16.7)	0.94 (0.62, 1.42)	0.763

CI = Confidence interval

*Death and amputation as competing risks

†Primary analysis but clinical site not included in the model.

‡Primary analysis plus the following included as independent variables in the model: total operative time, tumor location§, diabetes status, pre-operative chemotherapy and pre-operative radiation.

§Cox regression not stratified by tumor location (femur/tibia) as it is included as an independent variable.

© 2022 The PARITY Investigators. *JAMA Oncology*.

eTable 8: Subgroup Analyses

These subgroup analyses were performed by including the subgroup factor as an independent variable in our Cox proportional hazards regression model along with an interaction term between it and randomized treatment group. Separate models were performed for each subgroup variable. All models also include clinical site and tumor location (femur/tibia), similar to the primary analysis.

	**Five-Day Regimen**	**One-Day Regimen**	**Hazard Ratio (95% CI)**	**P-value for the interaction term**
Tumor Type				
Bone tumor	38/237 (16.0)	43/249 (17.3)	1.02 (0.65, 1.61)	0.537
Soft tissue sarcoma	5/28 (17.9)	7/34 (20.6)	0.49 (0.14, 1.66)	
Oligometastatic bone disease	1/28 (3.6)	2/28 (7.1)	0.73 (0.06, 8.27)	
Tumor Location*				
Tibia	11/53 (20.8)	10/55 (18.2)	1.18 (0.50, 2.80)	0.563
Femur	33/240 (13.8)	42/256 (16.4)	0.88 (0.56, 1.40)	
Sex				
Male	25/178 (14.0)	32/183 (17.5)	0.75 (0.44, 1.29)	0.167
Female	19/115 (16.5)	20/128 (15.6)	1.38 (0.72, 2.66)	
Age				
<31 years	19/118 (16.1)	23/138 (16.7)	1.11 (0.59, 2.09)	0.479
≥31 years	25/175 (14.3)	29/173 (16.8)	0.82 (0.47, 1.41)	
Pre-Operative Chemotherapy				
No	28/164 (17.1)	22/150 (14.7)	1.18 (0.67, 2.10)	0.234
Yes	16/129 (12.4)	30/161 (18.6)	0.70 (0.37, 1.32)	

CI = Confidence interval

*Cox regression for tumor location not stratified by femur/tibia.

© 2022 The PARITY Investigators. *JAMA Oncology*.

eTable 9: Study Outcomes by Treatment Group (Primary and Secondary)

Study Endpoint	Five-Day Regimen n=293	One-Day Regimen n=311	Hazard Ratio (95% CI)	P-value*
Primary Outcome				
**Any surgical site infection**	44 (15.0)	52 (16.7)	0.93 (0.62, 1.40)	0.730
Superficial incisional	13 (4.4)	12 (3.9)		
Deep incisional	3 (1.0)	8 (2.6)		
Organ/space	28 (9.6)	34 (10.9)	0.97 (0.59, 1.62)	0.916
Secondary Outcomes				
**Any antibiotic-related complications**	15 (5.1)	5 (1.6)	3.24 (1.17, 8.98)	0.024
Clostridioides difficile associated colitis	11 (3.8)	4 (1.3)		
Opportunistic fungal infection	0 (0.0)	1 (0.3)		
Oral candidiasis	1 (0.3)	0 (0.0)		
Diarrhea (unrelated to *Clostridioides difficile*) that required intervention	3 (1.0)	0 (0.0)		
**Any unplanned re-operation**	75 (25.6)	80 (25.7)	1.06 (0.77, 1.46)	0.722
Implant revision	21 (7.2)	14 (4.5)	1.89 (0.94, 3.80)	0.075
Irrigation and debridement	48 (16.4)	48 (15.4)	1.10 (0.73, 1.66)	0.641
Wound flap	8 (2.7)	5 (1.6)		
Skin graft	4 (1.4)	3 (1.0)		
Implant exchange	18 (6.1)	20 (6.4)	1.01 (0.53, 1.93)	0.968
Extensor mechanism reconstruction	3 (1.0)	5 (1.6)		
Repeat tumor excision	6 (2.0)	7 (2.3)		
Antibiotic spacer insertion	7 (2.4)	5 (1.6)		
Patellar resurfacing	0 (0.0)	1 (0.3)		
Abductor reconstruction	1 (0.3)	0 (0.0)		
Fasciotomy	2 (0.7)	0 (0.0)		
Amputation	8 (2.7)	12 (3.9)		
Other	33 (11.3)	27 (8.7)		
**Any oncologic events**	85 (29.0)	89 (28.6)	1.02 (0.75, 1.39)	0.895
Local recurrence	15 (5.1)	22 (7.1)	0.78 (0.40, 1.51)	0.456
Distant metastases	69 (23.5)	79 (25.4)	0.90 (0.65, 1.25)	0.527
Other oncologic event	7 (2.4)	8 (2.6)		
**All-cause mortality**	37 (12.6)	40 (12.9)	1.01 (0.64, 1.58)	0.982
Death due to disease progression	29 (9.9)	29 (9.3)	1.08 (0.64, 1.81)	0.778

© 2022 The PARITY Investigators. *JAMA Oncology*.

eTable 10: Functional and Quality of Life Outcomes by Treatment Group

	Five-Day Regimen n=250	One-Day Regimen n=264	Mean Difference† (95% CI)	P-value
Musculoskeletal Tumor Society-87 Questionnaire				
No. of completed cases	202	211	-0.49 (-1.67, 0.69)	0.411
Mean (SD)	27.3 (5.9)	27.8 (6.4)		
Median (Q1-Q3)	29 (25-31)	29 (25-33)		
Total range	3-35	2-35		
Musculoskeletal Tumor Society-93 Questionnaire				
No. of completed cases	196	204	-1.89 (-5.74, 1.97)	0.337
Mean (SD)	77.5 (18.6)	79.8 (21.3)		
Median (Q1-Q3)	80.0 (66.7-93.3)	86.7 (70.0-96.7)		
Total range	26.7-100.0	6.7-100.0		
Toronto Extremity Salvage Score Questionnaire				
No. of completed cases	195	214	0.10 (-3.30, 3.49)	0.956
Mean (SD)	80.6 (17.9)	81.5 (18.2)		
Median (Q1-Q3)	83.7 (73.1-94.0)	86.6 (72.1-95.0)		
Total range	6.7-100.0	2.3-100.0		

CI = Confidence interval; SD = standard deviation; Q1-Q3 = Quartile 1 to quartile 3
*Patients who did have an amputation and did not die within one-year post-surgery.
†Multiple imputation performed. Results from the linear regression model that includes treatment group, tumor location, clinical site and baseline score as independent variables. Mean difference is presented as Five-Day Regimen minus One-Day Regimen.

© 2022 The PARITY Investigators. *JAMA Oncology*.

References

1. Woods RK, Dellinger EP. Current Guidelines for Antibiotic Prophylaxis of Surgical Wounds. *Am Fam Physician*. 1998;57(11):2731.

2. de Beer J, Petruccelli D, Rotstein C, Weening B, Royston K, Winemaker M. Antibiotic prophylaxis for total joint replacement surgery: results of a survey of Canadian orthopedic surgeons. *Can J Surg J Can Chir*. 2009;52(6):E229-234.

3. Hasan K, Racano A, Deheshi B, et al. Prophylactic antibiotic regimens in tumor surgery (PARITY) survey. *BMC Musculoskelet Disord*. 2012;13:91. doi:10.1186/1471-2474-13-91

4. Marshall WF, Blair JE. The Cephalosporins. *Mayo Clin Proc*. 1999;74(2):187-195. doi:10.4065/74.2.187

5. Bui T, Preuss CV. Cephalosporins. In: *StatPearls*. StatPearls Publishing; 2021. Accessed July 29, 2021. http://www.ncbi.nlm.nih.gov/books/NBK551517/

6. Horan TC, Gaynes RP, Martone WJ, Jarvis WR, Emori TG. CDC Definitions of Nosocomial Surgical Site Infections, 1992: A Modification of CDC Definitions of Surgical Wound Infections. *Infect Control Hosp Epidemiol*. 1992;13(10):606-608. doi:10.2307/30148464

7. Schneider P, Heels-Ansdell D, Thabane L, et al. Prophylactic Antibiotic Regimens In Tumor Surgery (PARITY): a multi-center randomized controlled study comparing alternative antibiotic regimens in patients undergoing tumor resections with endoprosthetic replacements—a statistical analysis plan. *Trials*. 2021;22(1):223. doi:10.1186/s13063-021-05147-2

8. Racano A, Pazionis T, Farrokhyar F, Deheshi B, Ghert M. High Infection Rate Outcomes in Long-bone Tumor Surgery with Endoprosthetic Reconstruction in Adults: A Systematic Review. *Clin Orthop*. 2013;471(6):2017-2027. doi:10.1007/s11999-013-2842-9

9. Jeys LM, Grimer RJ, Carter SR, Tillman RM. Periprosthetic infection in patients treated for an orthopaedic oncological condition. *J Bone Joint Surg Am*. 2005;87(4):842-849. doi:10.2106/JBJS.C.01222

10. Jeys L, Grimer R. The Long-Term Risks of Infection and Amputation with Limb Salvage Surgery Using Endoprostheses. In: Tunn P-U, ed. *Treatment of Bone and Soft Tissue Sarcomas*. Recent Results in Cancer Research. Springer Berlin Heidelberg; 2009:75-84. doi:10.1007/978-3-540-77960-5_7

11. Langit MB, Miwa S, Yamamoto N, et al. Risk Factors for Postoperative Deep Infection After Malignant Bone Tumor Surgery of the Extremities. *Anticancer Res*. 2020;40(6):3551-3557. doi:10.21873/anticanres.14344

12. Severyns M, Briand S, Waast D, Touchais S, Hamel A, Gouin F. Postoperative infections after limb-sparing surgery for primary bone tumors of the pelvis: Incidence, characterization and functional impact. *Surg Oncol*. 2017;26(2):171-177. doi:10.1016/j.suronc.2017.03.005

13. Miwa S, Shirai T, Yamamoto N, et al. Risk factors for surgical site infection after malignant bone tumor resection and reconstruction. *BMC Cancer*. 2019;19(1):33. doi:10.1186/s12885-019-5270-8

14. Anatone AJ, Danford NC, Jang ES, Smartt A, Konigsberg M, Tyler WK. Risk Factors for Surgical Site Infection in Orthopaedic Oncology. *J Am Acad Orthop Surg*. 2020;28(20):e923-e928. doi:10.5435/JAAOS-D-19-00582

© 2022 The PARITY Investigators. *JAMA Oncology*.
