# Supplementary material for: Comparison of Prophylactic Intravenous Antibiotic Regimens After Endoprosthetic Reconstruction for Lower Extremity Bone Tumors: A Randomized Clinical Trial
Source: JAMA Oncol. 2022 Jan 6;8(3):345–53. doi: 10.1001/jamaoncol.2021.6628 (PMC8739829; doi:10.1001/jamaoncol.2021.6628)
Supplement: Supplement 3. — Nonauthor Collaborators. The PARITY Investigators [file jamaoncol-e216628-s003.pdf]

Supplemental Online Content: Nonauthor Collaborators

*Indicates required information. Only first name, last name, and suffix will appear in PubMed.

## *Group Name(s): The PARITY Investigators

| *First Name and Middle Initial(s) | *Last Name | *Suffix (eg, Jr, III) | Academic Degrees | Institution | Location (city, state/province, country) | Role or Contribution, eg, chair, principal investigator | Group (if more than 1 Group listed in the byline) and/or Subgroup (eg, Steering Committee) |
|---|---|---|---|---|---|---|---|
| Michelle | Ghert | | MD, FRCSC | McMaster University | Hamilton, Ontario, Canada | Chair (Steering Committee, Central Adjudication Committee), Principal Investigator (Juravinski Hospital and Cancer Centre) | Global Methods Center, Steering Committee, Central Adjudication Committee |
| Patricia | Schneider | | BSc | McMaster University | Hamilton, Ontario, Canada | Methods Center Research Personnel | Global Methods Center |
| Victoria | Giglio | | MSc | McMaster University | Hamilton, Ontario, Canada | Methods Center Research Personnel | Global Methods Center |
| Paula | McKay | | BSc | McMaster University | Hamilton, Ontario, Canada | Methods Center Research Personnel | Global Methods Center |
| Andrew | Duong | | MSc | McMaster University | Hamilton, Ontario, Canada | Methods Center Research Personnel | Global Methods Center |
| Nathan | Evaniew | | MD, PhD, FRCSC | McMaster University | Hamilton, Ontario, Canada | Methods Center Research Personnel | Global Methods Center |
| Dana | Ghanem | | BSc | McMaster University | Hamilton, Ontario, Canada | Methods Center Research Personnel | Global Methods Center |

Supplemental Online Content: Nonauthor Collaborators

*Indicates required information. Only first name, last name, and suffix will appear in PubMed.

| ***First Name and Middle Initial(s)** | ***Last Name** | ***Suffix (eg, Jr, III)** | Academic Degrees | Institution | Location (city, state/province, country) | Role or Contribution, eg, chair, principal investigator | Group (if more than 1 Group listed in the byline) and/or Subgroup (eg, Steering Committee) |
|---|---|---|---|---|---|---|---|
| Callum | MacLeay | | BSc | McMaster University | Hamilton, Ontario, Canada | Methods Center Research Personnel | Global Methods Center |
| Kim | Madden | | PhD | McMaster University | Hamilton, Ontario, Canada | Methods Center Research Personnel | Global Methods Center |
| Antonella | Racano | | DO | McMaster University | Hamilton, Ontario, Canada | Methods Center Research Personnel | Global Methods Center |
| Taryn | Scott | | MSW, MSc | McMaster University | Hamilton, Ontario, Canada | Methods Center Research Personnel | Global Methods Center |
| Marilyn | Swinton | | MSc | McMaster University | Hamilton, Ontario, Canada | Methods Center Research Personnel | Global Methods Center |
| Nicole | Simunovic | | MSc | McMaster University | Hamilton, Ontario, Canada | Methods Center Research Personnel | Global Methods Center |
| Sheila | Sprague | | PhD | McMaster University | Hamilton, Ontario, Canada | Methods Center Research Personnel | Global Methods Center |
| Diane | Heels-Ansdell | | MSc | McMaster University | Hamilton, Ontario, Canada | Methods Center Research Personnel | Global Methods Center |
| Lisa | Buckingham | | BSc | McMaster University | Hamilton, Ontario, Canada | Methods Center Research Personnel | Global Methods Center |

*Indicates required information. Only first name, last name, and suffix will appear in PubMed.

| ***First Name and Middle Initial(s)** | ***Last Name** | ***Suffix (eg, Jr, III)** | Academic Degrees | Institution | Location (city, state/province, country) | Role or Contribution, eg, chair, principal investigator | Group (if more than 1 Group listed in the byline) and/or Subgroup (eg, Steering Committee) |
|---|---|---|---|---|---|---|---|
| Roberto | Vélez | | MD, PhD | Vall d'Hebron Institut de Recerca | Barcelona, Spain | Site Principal Investigator (Hospital Vall d'Hebron), Legal Authorized Representative | European Coordinating Center, Steering Committee |
| Alba Lopez | Fernandez | | PhD | Vall d'Hebron Institut de Recerca, Hospital Vall d'Hebron | Barcelona, Spain | Site Research Personnel (Hospital Vall d'Hebron) | European Coordinating Center |
| Olga Sánchez-Maroto | Carrizo | | MS | Vall d'Hebron Institut de Recerca, Hospital Vall d'Hebron | Barcelona, Spain | European Coordinating Center Research Personnel | European Coordinating Center |
| Mohit | Bhandari | | MD, PhD, FRCSC | McMaster University | Hamilton, Ontario, Canada | Co-Chair (Steering Committee) | Steering Committee |
| Benjamin | Deheshi | | MD, MSc, FRCSC | McMaster University | Hamilton, Ontario, Canada | Site Co-investigator (Juravinski Hospital and Cancer Centre) | Steering Committee |
| Gordon | Guyatt | | MD | McMaster University | Hamilton, Ontario, Canada | | Steering Committee |
| Ginger | Holt | | MD | Vanderbilt University | Nashville, Tennessee, U.S.A. | Site Principal Investigator (Vanderbilt University Medical Center) | Steering Committee |
| Timothy | O'Shea | | MD, MPH | McMaster University | Hamilton, Ontario, Canada | | Steering Committee, Central Adjudication Committee |

Supplemental Online Content: Nonauthor Collaborators

*Indicates required information. Only first name, last name, and suffix will appear in PubMed.

| *First Name and Middle Initial(s) | *Last Name | *Suffix (eg, Jr, III) | Academic Degrees | Institution | Location (city, state/province, country) | Role or Contribution, eg, chair, principal investigator | Group (if more than 1 Group listed in the byline) and/or Subgroup (eg, Steering Committee) |
|---|---|---|---|---|---|---|---|
| R. Lor | Randall | | MD | University of California, Davis | Davis, California, U.S.A. | Site Principal Investigator (Huntsman Cancer Institute), Site Co-Investigator (University of California, Davis Medical Center) | Steering Committee, Central Adjudication Committee |
| Lehana | Thabane | | PhD | McMaster University | Hamilton, Ontario, Canada | | Steering Committee |
| Jay S. | Wunder | | MD, FRCSC | University of Toronto | Toronto, Ontario, Canada | Site Co-investigator (Mount Sinai Hospital) | Steering Committee |
| Robert | Turcotte | | MD, FRCSC | McGill University | Montreal, Québec, Canada | Site Principal Investigator (McGill University Health Centre) | Central Adjudication Committee |
| David | Wilson | | MD, MASc, FRCSC | McMaster University | Hamilton, Ontario, Canada | Site Co-investigator (Juravinski Hospital and Cancer Centre) | Central Adjudication Committee |
| Peter | Rose | | MD | Mayo Clinic | Rochester, Minnesota, U.S.A. | Chair (Data and Safety Monitoring Board) | Data and Safety Monitoring Board |
| Brian | Brigman | | MD | Duke University | Durham, North Carolina, U.S.A. | | Data and Safety Monitoring Board |
| Eleanor | Pullenayegum | | PhD | The Hospital for Sick Children | Toronto, Ontario, Canada | | Data and Safety Monitoring Board |
| Peter C. | Ferguson | | MD, FRCSC | Mount Sinai Hospital | Toronto, Ontario, Canada | Site Principal Investigator | |

*Indicates required information. Only first name, last name, and suffix will appear in PubMed.

| ***First Name and Middle Initial(s)** | ***Last Name** | ***Suffix (eg, Jr, III)** | Academic Degrees | Institution | Location (city, state/province, country) | Role or Contribution, eg, chair, principal investigator | Group (if more than 1 Group listed in the byline) and/or Subgroup (eg, Steering Committee) |
|---|---|---|---|---|---|---|---|
| Anthony M. | Griffin | | MSc | Mount Sinai Hospital | Toronto, Ontario, Canada | Site Research Personnel | |
| Gagan | Grewal | | BSc, RPhT | Mount Sinai Hospital | Toronto, Ontario, Canada | Site Research Pharmacy Personnel | |
| Andrew | Han | | BScPhm | Mount Sinai Hospital | Toronto, Ontario, Canada | Site Research Pharmacy Personnel | |
| Ionna | Mantas | | BScPhm, ACPR, RPh | Mount Sinai Hospital | Toronto, Ontario, Canada | Site Research Pharmacy Personnel | |
| Andrew | Wylie | | BScPhm, ACPR, PharmD | Mount Sinai Hospital | Toronto, Ontario, Canada | Site Research Pharmacy Personnel | |
| Krista | Goulding | | MD, MPH, FRCSC | McGill University Health Centre | Montreal, Québec, Canada | Site Co-investigator | |
| Nicole | Andersen | | BSc | McGill University Health Centre | Montreal, Québec, Canada | Site Research Personnel | |
| Olivier | Bouchereau | | MSc | McGill University Health Centre | Montreal, Québec, Canada | Site Research Personnel | |
| Firas | Dandachli | | MD, MSc | McGill University Health Centre | Montreal, Québec, Canada | Site Research Personnel | |
| Mireille | Dessureault | | MSc | McGill University Health Centre | Montreal, Québec, Canada | Site Research Personnel | |
| Steven | Salomon | | MSc | McGill University Health Centre | Montreal, Québec, Canada | Site Research Personnel | |
| Nathalie | Ste-Marie | | MSc | McGill University Health Centre | Montreal, Québec, Canada | Site Research Personnel | |

Supplemental Online Content: Nonauthor Collaborators

*Indicates required information. Only first name, last name, and suffix will appear in PubMed.

| ***First Name and Middle Initial(s)** | ***Last Name** | ***Suffix (eg, Jr, III)** | Academic Degrees | Institution | Location (city, state/province, country) | Role or Contribution, eg, chair, principal investigator | Group (if more than 1 Group listed in the byline) and/or Subgroup (eg, Steering Committee) |
|---|---|---|---|---|---|---|---|
| Ariane | Lessard | | PharmD | McGill University Health Centre, CIUSSS de l'Est-de-l'Île-de-Montréal – Hôpital Maisonneuve-Rosemont | Montreal, Québec, Canada | Site Research Pharmacy Personnel (McGill University Health Centre, CIUSSS de l'Est-de-l'Île-de-Montréal – Hôpital Maisonneuve-Rosemont) | |
| Gilbert | Matte | | PharmD | McGill University Health Centre | Montreal, Québec, Canada | Site Research Pharmacy Personnel | |
| Zoe | Bond | | BSc | Juravinski Hospital and Cancer Centre | Hamilton, Ontario, Canada | Site Research Personnel | |
| Bo Xuan | Lin | | BSc | Juravinski Hospital and Cancer Centre | Hamilton, Ontario, Canada | Site Research Personnel | |
| Maya | Biljan | | RPhT | Juravinski Hospital and Cancer Centre | Hamilton, Ontario, Canada | Site Research Pharmacy Personnel | |
| Rita | Chan | | BScPharm, MSc | Juravinski Hospital and Cancer Centre | Hamilton, Ontario, Canada | Site Research Pharmacy Personnel | |
| Deanna | Cosentino | | RPhT | Juravinski Hospital and Cancer Centre | Hamilton, Ontario, Canada | Site Research Pharmacy Personnel | |
| Diane | Lourenco | | RPhT | Juravinski Hospital and Cancer Centre | Hamilton, Ontario, Canada | Site Research Pharmacy Personnel | |
| Brittany | Marriott | | RPhT | Juravinski Hospital and Cancer Centre | Hamilton, Ontario, Canada | Site Research Pharmacy Personnel | |

Supplemental Online Content: Nonauthor Collaborators

*Indicates required information. Only first name, last name, and suffix will appear in PubMed.

| *First Name and Middle Initial(s) | *Last Name | *Suffix (eg, Jr, III) | Academic Degrees | Institution | Location (city, state/province, country) | Role or Contribution, eg, chair, principal investigator | Group (if more than 1 Group listed in the byline) and/or Subgroup (eg, Steering Committee) |
|---|---|---|---|---|---|---|---|
| Gita | Sobhi | | RPh | Juravinski Hospital and Cancer Centre | Hamilton, Ontario, Canada | Site Research Pharmacy Personnel | |
| Marc | Isler | | MD, FRCSC | CIUSSS de l'Est-de-l'Île-de-Montréal – Hôpital Maisonneuve-Rosemont | Montreal, Québec, Canada | Site Principal Investigator | |
| Sophie | Mottard | | MD, FRCSC | CIUSSS de l'Est-de-l'Île-de-Montréal – Hôpital Maisonneuve-Rosemont | Montreal, Québec, Canada | Site Co-investigator | |
| Janie | Barry | | MSc | CIUSSS de l'Est-de-l'Île-de-Montréal – Hôpital Maisonneuve-Rosemont | Montreal, Québec, Canada | Site Research Personnel | |
| Hugo | Saint-Yves | | MSc | CIUSSS de l'Est-de-l'Île-de-Montréal – Hôpital Maisonneuve-Rosemont | Montreal, Québec, Canada | Site Research Personnel | |
| Marysa | Bétournay | | | CIUSSS de l'Est-de-l'Île-de-Montréal – Hôpital Maisonneuve-Rosemont | Montreal, Québec, Canada | Site Research Personnel | |
| Marceline | Quach | | BPharm, MSc | CIUSSS de l'Est-de-l'Île-de-Montréal – Hôpital Maisonneuve-Rosemont | Montreal, Québec, Canada | Site Research Pharmacy Personnel | |
| Helen | Assayag | | BPharm | CIUSSS de l'Est-de-l'Île-de-Montréal – Hôpital Maisonneuve-Rosemont | Montreal, Québec, Canada | Site Research Pharmacy Personnel | |
| Karine | Daoust | | BPharm | CIUSSS de l'Est-de-l'Île-de-Montréal – Hôpital Maisonneuve-Rosemont | Montreal, Québec, Canada | Site Research Pharmacy Personnel | |
| Kristine | Goyette | | BPharm | CIUSSS de l'Est-de-l'Île-de-Montréal – Hôpital Maisonneuve-Rosemont | Montreal, Québec, Canada | Site Research Pharmacy Personnel | |

*Indicates required information. Only first name, last name, and suffix will appear in PubMed.

| *First Name and Middle Initial(s) | *Last Name | *Suffix (eg, Jr, III) | Academic Degrees | Institution | Location (city, state/province, country) | Role or Contribution, eg, chair, principal investigator | Group (if more than 1 Group listed in the byline) and/or Subgroup (eg, Steering Committee) |
|---|---|---|---|---|---|---|---|
| Denis | Projean | | BPharm, PhD | CIUSSS de l'Est-de-l'Île-de-Montréal – Hôpital Maisonneuve-Rosemont | Montreal, Québec, Canada | Site Research Pharmacy Personnel | |
| Millie | Lum | | BPharm | CIUSSS de l'Est-de-l'Île-de-Montréal – Hôpital Maisonneuve-Rosemont | Montreal, Québec, Canada | Site Research Pharmacy Personnel | |
| Maude | Bachand-Fournier | | BPharm | CIUSSS de l'Est-de-l'Île-de-Montréal – Hôpital Maisonneuve-Rosemont | Montreal, Québec, Canada | Site Research Pharmacy Personnel | |
| Norbert | Dion | | MD, FRCSC | CHU de Québec – Université Laval | Québec, Québec, Canada | Site Principal Investigator | |
| Annie | Arteau | | MD, FRCSC | CHU de Québec – Université Laval | Québec, Québec, Canada | Site Co-investigator | |
| Sylvie | Turmel | | RN | CHU de Québec – Université Laval | Québec, Québec, Canada | Site Research Personnel | |
| Anne | Bertrand | | MSc | CHU de Québec – Université Laval | Québec, Québec, Canada | Site Research Pharmacy Personnel | |
| Manon | D'Amours | | PTA | CHU de Québec – Université Laval | Québec, Québec, Canada | Site Research Pharmacy Personnel | |
| Lucie | Dallaire | | MSc | CHU de Québec – Université Laval | Québec, Québec, Canada | Site Research Pharmacy Personnel | |
| Nancy | Gagnon | | MSc | CHU de Québec – Université Laval | Québec, Québec, Canada | Site Research Pharmacy Personnel | |
| Lucie | Gosselin | | MSc | CHU de Québec – Université Laval | Québec, Québec, Canada | Site Research Pharmacy Personnel | |

Supplemental Online Content: Nonauthor Collaborators

*Indicates required information. Only first name, last name, and suffix will appear in PubMed.

| *First Name and Middle Initial(s) | *Last Name | *Suffix (eg, Jr, III) | Academic Degrees | Institution | Location (city, state/province, country) | Role or Contribution, eg, chair, principal investigator | Group (if more than 1 Group listed in the byline) and/or Subgroup (eg, Steering Committee) |
|---|---|---|---|---|---|---|---|
| Gladys | Grenier | | PTA | CHU de Québec – Université Laval | Québec, Québec, Canada | Site Research Pharmacy Personnel | |
| Véronique | Labbé | | MSc | CHU de Québec – Université Laval | Québec, Québec, Canada | Site Research Pharmacy Personnel | |
| Tuong-Vi | Tran | | MSc, PhD | CHU de Québec – Université Laval | Québec, Québec, Canada | Site Research Pharmacy Personnel | |
| Paul | Clarkson | | MBChB, MSc | Vancouver General Hospital | Vancouver, British Columbia, Canada | Site Principal Investigator | |
| Lisa | Kondo | | BScN, RN | Vancouver General Hospital | Vancouver, British Columbia, Canada | Site Research Personnel | |
| Baohua | Wang | | PhD | Vancouver General Hospital | Vancouver, British Columbia, Canada | Site Research Personnel | |
| Judy | Yip | | RPh | Vancouver General Hospital | Vancouver, British Columbia, Canada | Site Research Pharmacy Personnel | |
| Joel | Werier | | MD, FRCSC | The Ottawa Hospital | Ottawa, Ontario, Canada | Site Principal Investigator | |
| Hesham | Abdelbary | | MD, MSc, FRCSC | The Ottawa Hospital | Ottawa, Ontario, Canada | Site Co-investigator | |
| Yusra | Kassim | | MD, PhD | The Ottawa Hospital | Ottawa, Ontario, Canada | Site Research Personnel | |
| Heather | Cosgrove | | BA | The Ottawa Hospital | Ottawa, Ontario, Canada | Site Research Personnel | |
| Kimberly | Paquin | | BA | The Ottawa Hospital | Ottawa, Ontario, Canada | Site Research Personnel | |
| Anne-Marie | Dugal | | RPhT | The Ottawa Hospital | Ottawa, Ontario, Canada | Site Research Pharmacy Personnel | |

*Indicates required information. Only first name, last name, and suffix will appear in PubMed.

| *First Name and Middle Initial(s) | *Last Name | *Suffix (eg, Jr, III) | Academic Degrees | Institution | Location (city, state/province, country) | Role or Contribution, eg, chair, principal investigator | Group (if more than 1 Group listed in the byline) and/or Subgroup (eg, Steering Committee) |
|---|---|---|---|---|---|---|---|
| Susan | Fetzer | | RPhT | The Ottawa Hospital | Ottawa, Ontario, Canada | Site Research Pharmacy Personnel | |
| Wendy | Aikens | | RPhT | The Ottawa Hospital | Ottawa, Ontario, Canada | Site Research Pharmacy Personnel | |
| Shannon | Puloski | | MD, FRCSC | Foothills Medical Centre | Calgary, Alberta, Canada | Site Principal Investigator | |
| Michael | Monument | | MD, MSc, FRCSC | Foothills Medical Centre | Calgary, Alberta, Canada | Site Co-investigator | |
| Kimberly | Carcary | | MSc | Foothills Medical Centre | Calgary, Alberta, Canada | Site Research Personnel | |
| Olesja | Hazenbiller | | MSc | Foothills Medical Centre | Calgary, Alberta, Canada | Site Research Personnel | |
| Kayla | Kashluba | | MSc | Foothills Medical Centre | Calgary, Alberta, Canada | Site Research Personnel | |
| Jimena | Rodriguez | | MSc | Foothills Medical Centre | Calgary, Alberta, Canada | Site Research Personnel | |
| Candice | Cameron | | BA, BSP, ACPR | Foothills Medical Centre | Calgary, Alberta, Canada | Site Research Pharmacy Personnel | |
| Yee-Cheen | Doung | | MD | Oregon Health & Science University Hospital | Portland, Oregon, U.S.A. | Site Principal Investigator | |
| Kenneth | Gundle | | MD | Oregon Health & Science University Hospital | Portland, Oregon, U.S.A. | Site Co-investigator | |
| James | Hayden | | MD, PhD | Oregon Health & Science University Hospital | Portland, Oregon, U.S.A. | Site Co-investigator | |
| Christopher | Hart | | MD | Oregon Health & Science University Hospital | Portland, Oregon, U.S.A. | Site Research Personnel | |
| David | Jenkins | | BA | Oregon Health & Science University Hospital | Portland, Oregon, U.S.A. | Site Research Personnel | |

Supplemental Online Content: Nonauthor Collaborators

*Indicates required information. Only first name, last name, and suffix will appear in PubMed.

| ***First Name and Middle Initial(s)** | ***Last Name** | ***Suffix (eg, Jr, III)** | Academic Degrees | Institution | Location (city, state/province, country) | Role or Contribution, eg, chair, principal investigator | Group (if more than 1 Group listed in the byline) and/or Subgroup (eg, Steering Committee) |
|---|---|---|---|---|---|---|---|
| Rebecca I. | Wetzel | | BS | Oregon Health & Science University Hospital | Portland, Oregon, U.S.A. | Site Research Personnel | |
| Krista | Wolf | | PharmD | Oregon Health & Science University Hospital | Portland, Oregon, U.S.A. | Site Research Pharmacy Personnel | |
| Brooke | Bernard | | PharmD | Oregon Health & Science University Hospital | Portland, Oregon, U.S.A. | Site Research Pharmacy Personnel | |
| Sara | Blefgen | | RPh | Oregon Health & Science University Hospital | Portland, Oregon, U.S.A. | Site Research Pharmacy Personnel | |
| Kevin | Jones | | MD | Huntsman Cancer Institute | Salt Lake City, Utah, U.S.A. | Site Principal Investigator | |
| John | Groundland | | MD, MS | Huntsman Cancer Institute | Salt Lake City, Utah, U.S.A. | Site Co-investigator | |
| Susie | Crabtree | | AS | Huntsman Cancer Institute | Salt Lake City, Utah, U.S.A. | Site Research Personnel | |
| Jacqueline | Hart | | AS | Huntsman Cancer Institute | Salt Lake City, Utah, U.S.A. | Site Research Personnel | |
| Sara | Shaw | | BS | Huntsman Cancer Institute | Salt Lake City, Utah, U.S.A. | Site Research Personnel | |
| Rian | Davis | | PharmD | Huntsman Cancer Institute | Salt Lake City, Utah, U.S.A. | Site Research Pharmacy Personnel | |
| Winter | Redd | | PharmD | Huntsman Cancer Institute | Salt Lake City, Utah, U.S.A. | Site Research Pharmacy Personnel | |
| Susan | Sorenson | | PharmD | Huntsman Cancer Institute | Salt Lake City, Utah, U.S.A. | Site Research Pharmacy Personnel | |

Supplemental Online Content: Nonauthor Collaborators

*Indicates required information. Only first name, last name, and suffix will appear in PubMed.

| ***First Name and Middle Initial(s)** | ***Last Name** | ***Suffix (eg, Jr, III)** | Academic Degrees | Institution | Location (city, state/province, country) | Role or Contribution, eg, chair, principal investigator | Group (if more than 1 Group listed in the byline) and/or Subgroup (eg, Steering Committee) |
|---|---|---|---|---|---|---|---|
| Benjamin | Miller | | MD, MS | Holden Comprehensive Cancer Center | Iowa City, Iowa, U.S.A. | Site Principal Investigator | |
| Mohammed | Milhem | | MBBS | Holden Comprehensive Cancer Center | Iowa City, Iowa, U.S.A. | Site Co-investigator | |
| Jill | Kain | | MSN, ARNP | Holden Comprehensive Cancer Center | Iowa City, Iowa, U.S.A. | Site Co-investigator | |
| Marian | Andersen | | MA, CCRP | Holden Comprehensive Cancer Center | Iowa City, Iowa, U.S.A. | Site Research Personnel | |
| Kathryn | Hillburn | | RN, BSN | Holden Comprehensive Cancer Center | Iowa City, Iowa, U.S.A. | Site Research Personnel | |
| Jennifer | Larson | | AAS, CMA | Holden Comprehensive Cancer Center | Iowa City, Iowa, U.S.A. | Site Research Personnel | |
| Nancy | McCurdy | | RN | Holden Comprehensive Cancer Center | Iowa City, Iowa, U.S.A. | Site Research Personnel | |
| Alyssa | Pratt | | MS, CCRP | Holden Comprehensive Cancer Center | Iowa City, Iowa, U.S.A. | Site Research Personnel | |
| Mary | Schall | | BSN | Holden Comprehensive Cancer Center | Iowa City, Iowa, U.S.A. | Site Research Personnel | |
| Theresa | Hobbs | | BSPharm, RPh | Holden Comprehensive Cancer Center | Iowa City, Iowa, U.S.A. | Site Research Pharmacy Personnel | |
| Kristine | Johnson | | BSPharm, RPh | Holden Comprehensive Cancer Center | Iowa City, Iowa, U.S.A. | Site Research Pharmacy Personnel | |
| Joanna | Nohr | | PharmD | Holden Comprehensive Cancer Center | Iowa City, Iowa, U.S.A. | Site Research Pharmacy Personnel | |
| Wendi | Slaughter | | PharmD, RPh | Holden Comprehensive Cancer Center | Iowa City, Iowa, U.S.A. | Site Research Pharmacy Personnel | |

*Indicates required information. Only first name, last name, and suffix will appear in PubMed.

| *First Name and Middle Initial(s) | *Last Name | *Suffix (eg, Jr, III) | Academic Degrees | Institution | Location (city, state/province, country) | Role or Contribution, eg, chair, principal investigator | Group (if more than 1 Group listed in the byline) and/or Subgroup (eg, Steering Committee) |
|---|---|---|---|---|---|---|---|
| Jennifer | Halpern | | MD | Vanderbilt University Medical Center | Nashville, Tennessee, U.S.A. | Site Co-investigator | |
| Herbert | Schwartz | | MD | Vanderbilt University Medical Center | Nashville, Tennessee, U.S.A. | Site Co-investigator | |
| Julie | Daniels | | CCRP | Vanderbilt University Medical Center | Nashville, Tennessee, U.S.A. | Site Research Personnel | |
| Eden | Schafer | | MPH | Vanderbilt University Medical Center | Nashville, Tennessee, U.S.A. | Site Research Personnel | |
| M. Shane | Moore | | PharmD | Vanderbilt University Medical Center | Nashville, Tennessee, U.S.A. | Site Research Pharmacy Personnel | |
| John H. | Healey | | MD | Memorial Sloan Kettering Cancer Center | New York City, New York, U.S.A. | Site Principal Investigator | |
| Kaity | Chang | | MBA | Memorial Sloan Kettering Cancer Center | New York City, New York, U.S.A. | Site Research Personnel | |
| Linda | Chen | | MS | Memorial Sloan Kettering Cancer Center | New York City, New York, U.S.A. | Site Research Personnel | |
| Olivera | Douvelis | | BA | Memorial Sloan Kettering Cancer Center | New York City, New York, U.S.A. | Site Research Personnel | |
| Jesse | Galle | | BA | Memorial Sloan Kettering Cancer Center | New York City, New York, U.S.A. | Site Research Personnel | |
| Marissa | Mezzancello | | MPH, MS | Memorial Sloan Kettering Cancer Center | New York City, New York, U.S.A. | Site Research Personnel | |
| Yoely | Tavarez | | BA | Memorial Sloan Kettering Cancer Center | New York City, New York, U.S.A. | Site Research Personnel | |
| Brian | Del Corral | | PharmD | Memorial Sloan Kettering Cancer Center | New York City, New York, U.S.A. | Site Research Pharmacy Personnel | |
| Sabrina | Lopez | | PharmD | Memorial Sloan Kettering Cancer Center | New York City, New York, U.S.A. | Site Research Pharmacy Personnel | |

*Indicates required information. Only first name, last name, and suffix will appear in PubMed.

| *First Name and Middle Initial(s) | *Last Name | *Suffix (eg, Jr, III) | Academic Degrees | Institution | Location (city, state/province, country) | Role or Contribution, eg, chair, principal investigator | Group (if more than 1 Group listed in the byline) and/or Subgroup (eg, Steering Committee) |
|---|---|---|---|---|---|---|---|
| Gerry | O'Neill | | PharmD | Memorial Sloan Kettering Cancer Center | New York City, New York, U.S.A. | Site Research Pharmacy Personnel | |
| John | Abraham | | MD | The Rothman Institute at Thomas Jefferson University Hospital | Philadelphia, Pennsylvania, U.S.A. | Site Principal Investigator | |
| Scot | Brown | | MD | The Rothman Institute at Thomas Jefferson University Hospital | Philadelphia, Pennsylvania, U.S.A. | Site Co-investigator | |
| Meghan | Angelos | | | The Rothman Institute at Thomas Jefferson University Hospital | Philadelphia, Pennsylvania, U.S.A. | Site Research Personnel | |
| Keenan | Sobol | | BS | The Rothman Institute at Thomas Jefferson University Hospital | Philadelphia, Pennsylvania, U.S.A. | Site Research Personnel | |
| John | Strony | | BS | The Rothman Institute at Thomas Jefferson University Hospital | Philadelphia, Pennsylvania, U.S.A. | Site Research Personnel | |
| Braden | Rall | | PharmD, BCPS, BCOP | The Rothman Institute at Thomas Jefferson University Hospital | Philadelphia, Pennsylvania, U.S.A. | Site Research Pharmacy Personnel | |
| Melissa | Furio | | PharmD | The Rothman Institute at Thomas Jefferson University Hospital | Philadelphia, Pennsylvania, U.S.A. | Site Research Pharmacy Personnel | |
| Linda | Sailor | | PharmD | The Rothman Institute at Thomas Jefferson University Hospital | Philadelphia, Pennsylvania, U.S.A. | Site Research Pharmacy Personnel | |
| Rania | Sadaka | | PharmD | The Rothman Institute at Thomas Jefferson University Hospital | Philadelphia, Pennsylvania, U.S.A. | Site Research Pharmacy Personnel | |

Supplemental Online Content: Nonauthor Collaborators

*Indicates required information. Only first name, last name, and suffix will appear in PubMed.

| ***First Name and Middle Initial(s)** | ***Last Name** | ***Suffix (eg, Jr, III)** | Academic Degrees | Institution | Location (city, state/province, country) | Role or Contribution, eg, chair, principal investigator | Group (if more than 1 Group listed in the byline) and/or Subgroup (eg, Steering Committee) |
|---|---|---|---|---|---|---|---|
| Lauren | Karel | | PharmD, BCPS | The Rothman Institute at Thomas Jefferson University Hospital | Philadelphia, Pennsylvania, U.S.A. | Site Research Pharmacy Personnel | |
| David | Geller | | MD | Montefiore Medical Center | Bronx, New York, U.S.A. | Site Principal Investigator | |
| Bang | Hoang | | MD | Montefiore Medical Center | Bronx, New York, U.S.A. | Site Co-investigator | |
| Janet | Tingling | | AA, AS, BS, MS, MBA, PhD | Montefiore Medical Center | Bronx, New York, U.S.A. | Site Research Personnel | |
| Clemencia | Solorzano | | PharmD, RPh | Montefiore Medical Center | Bronx, New York, U.S.A. | Site Research Pharmacy Personnel | |
| Rosanna | Wustrack | | MD | University of California, San Francisco Medical Center | San Francisco, California, U.S.A. | Site Principal Investigator | |
| Richard | O'Donnell | | MD | University of California, San Francisco Medical Center | San Francisco, California, U.S.A. | Site Co-investigator | |
| Melissa | Zimel | | MD | University of California, San Francisco Medical Center | San Francisco, California, U.S.A. | Site Co-investigator | |
| Veronica | Andaya | | BA | University of California, San Francisco Medical Center | San Francisco, California, U.S.A. | Site Research Personnel | |
| Adrianna | Carrasco | | BS | University of California, San Francisco Medical Center | San Francisco, California, U.S.A. | Site Research Personnel | |
| Shirley | Chen | | PharmD | University of California, San Francisco Medical Center | San Francisco, California, U.S.A. | Site Research Pharmacy Personnel | |
| Diana | Ng | | PharmD | University of California, San Francisco Medical Center | San Francisco, California, U.S.A. | Site Research Pharmacy Personnel | |

Supplemental Online Content: Nonauthor Collaborators

*Indicates required information. Only first name, last name, and suffix will appear in PubMed.

| ***First Name and Middle Initial(s)** | ***Last Name** | ***Suffix (eg, Jr, III)** | Academic Degrees | Institution | Location (city, state/province, country) | Role or Contribution, eg, chair, principal investigator | Group (if more than 1 Group listed in the byline) and/or Subgroup (eg, Steering Committee) |
|---|---|---|---|---|---|---|---|
| Yelena | Koplowicz | | PharmD | University of California, San Francisco Medical Center | San Francisco, California, U.S.A. | Site Research Pharmacy Personnel | |
| André | Spiguel | | MD | University of Florida Health Shands Hospital | Gainesville, Florida, U.S.A. | Site Principal Investigator | |
| Chung Ming | Chan | | MD | University of Florida Health Shands Hospital | Gainesville, Florida, U.S.A. | Site Co-investigator | |
| Charles Parker | Gibbs | | MD | University of Florida Health Shands Hospital | Gainesville, Florida, U.S.A. | Site Co-investigator | |
| Mark | Scarborough | | MD | University of Florida Health Shands Hospital | Gainesville, Florida, U.S.A. | Site Co-investigator | |
| MaryBeth | Horodyski | | EdD, LAT, ATC, FNATA | University of Florida Health Shands Hospital | Gainesville, Florida, U.S.A. | Site Co-investigator | |
| Johanna | Carmona | | LPN | University of Florida Health Shands Hospital | Gainesville, Florida, U.S.A. | Site Research Personnel | |
| Alana | Jackson | | MS | University of Florida Health Shands Hospital | Gainesville, Florida, U.S.A. | Site Research Personnel | |
| Aimee | Struk | | Med, MBA, LAT, ATC | University of Florida Health Shands Hospital | Gainesville, Florida, U.S.A. | Site Research Personnel | |
| Susan | Beltz | | PharmD | University of Florida Health Shands Hospital | Gainesville, Florida, U.S.A. | Site Research Pharmacy Personnel | |
| Justin C. | Giaquinta | | PharmD | University of Florida Health Shands Hospital | Gainesville, Florida, U.S.A. | Site Research Pharmacy Personnel | |
| Melissa | Johnson | | PharmD | University of Florida Health Shands Hospital | Gainesville, Florida, U.S.A. | Site Research Pharmacy Personnel | |

Supplemental Online Content: Nonauthor Collaborators

*Indicates required information. Only first name, last name, and suffix will appear in PubMed.

| *First Name and Middle Initial(s) | *Last Name | *Suffix (eg, Jr, III) | Academic Degrees | Institution | Location (city, state/province, country) | Role or Contribution, eg, chair, principal investigator | Group (if more than 1 Group listed in the byline) and/or Subgroup (eg, Steering Committee) |
|---|---|---|---|---|---|---|---|
| Edward Y. | Cheng | | MD | University of Minnesota | Minneapolis, Minnesota, U.S.A. | Site Principal Investigator | |
| Julie | Agel | | MA, ATC | University of Minnesota | Minneapolis, Minnesota, U.S.A. | Site Research Personnel | |
| Theresa | Christiansen | | RPh | University of Minnesota | Minneapolis, Minnesota, U.S.A. | Site Research Pharmacy Personnel | |
| Derek | LaBar | | PharmD | University of Minnesota | Minneapolis, Minnesota, U.S.A. | Site Research Pharmacy Personnel | |
| Darlette | Luke | | RPh | University of Minnesota | Minneapolis, Minnesota, U.S.A. | Site Research Pharmacy Personnel | |
| Raffi | Avedian | | MD | Stanford University Health Care | Palo Alto, California, U.S.A. | Site Principal Investigator | |
| Linda | Jordan | | PA-C | Stanford University Health Care | Palo Alto, California, U.S.A. | Site Research Personnel | |
| Deborah | Kenney | | MS, OTR | Stanford University Health Care | Palo Alto, California, U.S.A. | Site Research Personnel | |
| Steven | Chinn | | PharmD | Stanford University Health Care | Palo Alto, California, U.S.A. | Site Research Pharmacy Personnel | |
| Martha | Hamilton | | PharmD | Stanford University Health Care | Palo Alto, California, U.S.A. | Site Research Pharmacy Personnel | |
| Scott | Mayeda | | PharmD | Stanford University Health Care | Palo Alto, California, U.S.A. | Site Research Pharmacy Personnel | |
| Carol | Morris | | MD, MS | Johns Hopkins Hospital | Baltimore, Maryland, U.S.A. | Site Principal Investigator | |

Supplemental Online Content: Nonauthor Collaborators

*Indicates required information. Only first name, last name, and suffix will appear in PubMed.

| *First Name and Middle Initial(s) | *Last Name | *Suffix (eg, Jr, III) | Academic Degrees | Institution | Location (city, state/province, country) | Role or Contribution, eg, chair, principal investigator | Group (if more than 1 Group listed in the byline) and/or Subgroup (eg, Steering Committee) |
|---|---|---|---|---|---|---|---|
| Adam | Levin | | MD | Johns Hopkins Hospital | Baltimore, Maryland, U.S.A. | Site Co-investigator | |
| Kari | Albery | | PA-C | Johns Hopkins Hospital | Baltimore, Maryland, U.S.A. | Site Research Personnel | |
| Jennifer | Giordano | | CRNP | Johns Hopkins Hospital | Baltimore, Maryland, U.S.A. | Site Research Personnel | |
| Vaishali | Laljani | | | Johns Hopkins Hospital | Baltimore, Maryland, U.S.A. | Site Research Personnel | |
| Anne | Delisa | | PharmD | Johns Hopkins Hospital | Baltimore, Maryland, U.S.A. | Site Research Pharmacy Personnel | |
| Nathan | Mesko | | MD | The Cleveland Clinic | Cleveland, Ohio, U.S.A. | Site Principal Investigator | |
| Lukas | Nystrom | | MD | The Cleveland Clinic | Cleveland, Ohio, U.S.A. | Site Co-investigator | |
| Matthew | Rerko | | | The Cleveland Clinic | Cleveland, Ohio, U.S.A. | Site Research Personnel | |
| Heather | Keaney | | MPH | The Cleveland Clinic | Cleveland, Ohio, U.S.A. | Site Research Personnel | |
| Rachael | Yim | | PharmD, MPH | The Cleveland Clinic | Cleveland, Ohio, U.S.A. | Site Research Pharmacy Personnel | |
| John | Petrich | | MS, RPh | The Cleveland Clinic | Cleveland, Ohio, U.S.A. | Site Research Pharmacy Personnel | |
| Megan E. | Anderson | | MD | Boston Children's Hospital | Boston, Massachusetts, U.S.A. | Site Principal Investigator (Boston Children's Hospital, Beth Israel Deaconess Medical Center) | |

*Indicates required information. Only first name, last name, and suffix will appear in PubMed.

| *First Name and Middle Initial(s) | *Last Name | *Suffix (eg, Jr, III) | Academic Degrees | Institution | Location (city, state/province, country) | Role or Contribution, eg, chair, principal investigator | Group (if more than 1 Group listed in the byline) and/or Subgroup (eg, Steering Committee) |
|---|---|---|---|---|---|---|---|
| Mark C. | Gebhardt | | MD | Boston Children's Hospital | Boston, Massachusetts, U.S.A. | Site Co-investigator (Boston Children's Hospital, Beth Israel Deaconess Medical Center) | |
| Benjamin | Allar | | MD | Boston Children's Hospital | Boston, Massachusetts, U.S.A. | Site Research Personnel | |
| Michael | Greenberg | | BS | Boston Children's Hospital | Boston, Massachusetts, U.S.A. | Site Research Personnel | |
| Manahil | Naqvi | | MS | Boston Children's Hospital | Boston, Massachusetts, U.S.A. | Site Research Personnel | |
| Ellis | Prather | | MBA | Boston Children's Hospital | Boston, Massachusetts, U.S.A. | Site Research Personnel | |
| Emily | Rademacher | | BS | Boston Children's Hospital | Boston, Massachusetts, U.S.A. | Site Research Personnel | |
| Jodie | Shea | | BS | Boston Children's Hospital | Boston, Massachusetts, U.S.A. | Site Research Personnel | |
| James | Bennett | | PharmD | Boston Children's Hospital | Boston, Massachusetts, U.S.A. | Site Research Pharmacy Personnel | |
| Stacey | Albuquerque | | BS, PharmD | Boston Children's Hospital | Boston, Massachusetts, U.S.A. | Site Research Pharmacy Personnel | |
| Michael | Giarrusso | | PharmD | Boston Children's Hospital | Boston, Massachusetts, U.S.A. | Site Research Pharmacy Personnel | |

Supplemental Online Content: Nonauthor Collaborators

*Indicates required information. Only first name, last name, and suffix will appear in PubMed.

| *First Name and Middle Initial(s) | *Last Name | *Suffix (eg, Jr, III) | Academic Degrees | Institution | Location (city, state/province, country) | Role or Contribution, eg, chair, principal investigator | Group (if more than 1 Group listed in the byline) and/or Subgroup (eg, Steering Committee) |
|---|---|---|---|---|---|---|---|
| Albert J. | Aboulafia | | MD, MBA | MedStar Georgetown Cancer Institute at Franklin Square | Baltimore, Maryland, U.S.A. | Site Principal Investigator (MedStar Georgetown Cancer Institute at Franklin Square, Sinai Hospital of Baltimore [Lifebridge Health]) | |
| Matthew T. | Wallace | | MD, MBA | MedStar Georgetown Cancer Institute at Franklin Square | Baltimore, Maryland, U.S.A. | Site Co-investigator (MedStar Georgetown Cancer Institute at Franklin Square, Sinai Hospital of Baltimore [Lifebridge Health]) | |
| Sally | Brown | | RN, BSN, MGA, OCN | MedStar Georgetown Cancer Institute at Franklin Square | Baltimore, Maryland, U.S.A. | Site Research Personnel | |
| Janice | Fowler | | | MedStar Georgetown Cancer Institute at Franklin Square | Baltimore, Maryland, U.S.A. | Site Research Personnel | |
| Jean | Flack | | RN, BSN, OCN, CCRC | MedStar Georgetown Cancer Institute at Franklin Square | Baltimore, Maryland, U.S.A. | Site Research Personnel | |
| Rick | Battersby | | RPh | MedStar Georgetown Cancer Institute at Franklin Square | Baltimore, Maryland, U.S.A. | Site Research Pharmacy Personnel | |

*Indicates required information. Only first name, last name, and suffix will appear in PubMed.

| *First Name and Middle Initial(s) | *Last Name | *Suffix (eg, Jr, III) | Academic Degrees | Institution | Location (city, state/province, country) | Role or Contribution, eg, chair, principal investigator | Group (if more than 1 Group listed in the byline) and/or Subgroup (eg, Steering Committee) |
|---|---|---|---|---|---|---|---|
| Chad | Taylor | | PharmD | MedStar Georgetown Cancer Institute at Franklin Square | Baltimore, Maryland, U.S.A. | Site Research Pharmacy Personnel | |
| David | Greenberg | | MD | Saint Louis University | St. Louis, Missouri, U.S.A. | Site Principal Investigator | |
| Sarah | Dawson | | RN, BSN | Saint Louis University | St. Louis, Missouri, U.S.A. | Site Research Personnel | |
| Adam | Riebeling | | PharmD | Saint Louis University | St. Louis, Missouri, U.S.A. | Site Research Pharmacy Personnel | |
| Anna | Schmidt | | PharmD | Saint Louis University | St. Louis, Missouri, U.S.A. | Site Research Pharmacy Personnel | |
| Eric | Henderson | | MD | Dartmouth-Hitchcock Medical Center | Lebanon, New Hampshire, U.S.A. | Site Principal Investigator | |
| Peter | DePalo | Sr | BS, CCRP, CPhT | Dartmouth-Hitchcock Medical Center | Lebanon, New Hampshire, U.S.A. | Site Research Personnel | |
| Lisa | Mack | | RN | Dartmouth-Hitchcock Medical Center | Lebanon, New Hampshire, U.S.A. | Site Research Personnel | |
| Christine | Neely-Jones | | RN | Dartmouth-Hitchcock Medical Center | Lebanon, New Hampshire, U.S.A. | Site Research Personnel | |
| Crystallee | Newton | | BA, CCRC | Dartmouth-Hitchcock Medical Center | Lebanon, New Hampshire, U.S.A. | Site Research Personnel | |
| Daniel | Ressler | | BA | Dartmouth-Hitchcock Medical Center | Lebanon, New Hampshire, U.S.A. | Site Research Personnel | |
| Holly | Symonds | | CCRC | Dartmouth-Hitchcock Medical Center | Lebanon, New Hampshire, U.S.A. | Site Research Personnel | |
| Iryna | Gardner | | CPhT | Dartmouth-Hitchcock Medical Center | Lebanon, New Hampshire, U.S.A. | Site Research Pharmacy Personnel | |

Supplemental Online Content: Nonauthor Collaborators

*Indicates required information. Only first name, last name, and suffix will appear in PubMed.

| ***First Name and Middle Initial(s)** | ***Last Name** | ***Suffix (eg, Jr, III)** | Academic Degrees | Institution | Location (city, state/province, country) | Role or Contribution, eg, chair, principal investigator | Group (if more than 1 Group listed in the byline) and/or Subgroup (eg, Steering Committee) |
|---|---|---|---|---|---|---|---|
| Douglas | Parr | | PharmD | Dartmouth-Hitchcock Medical Center | Lebanon, New Hampshire, U.S.A. | Site Research Pharmacy Personnel | |
| Victoria | Poisson | | CPhT | Dartmouth-Hitchcock Medical Center | Lebanon, New Hampshire, U.S.A. | Site Research Pharmacy Personnel | |
| David | Rozolsky | | PharmD | Dartmouth-Hitchcock Medical Center | Lebanon, New Hampshire, U.S.A. | Site Research Pharmacy Personnel | |
| Patrick | Teune | | PharmD | Dartmouth-Hitchcock Medical Center | Lebanon, New Hampshire, U.S.A. | Site Research Pharmacy Personnel | |
| Joseph | Schwab | | MD | Massachusetts General Hospital | Boston, Massachusetts, U.S.A. | Site Principal Investigator | |
| Santiago A. | Lozano-Calderon | | MD, PhD | Massachusetts General Hospital | Boston, Massachusetts, U.S.A. | Site Co-investigator | |
| Jonathan | Baker | | BS | Massachusetts General Hospital | Boston, Massachusetts, U.S.A. | Site Research Personnel | |
| Emily Ann | Berner | | BS | Massachusetts General Hospital | Boston, Massachusetts, U.S.A. | Site Research Personnel | |
| Gi Hye | Im | | BA | Massachusetts General Hospital | Boston, Massachusetts, U.S.A. | Site Research Personnel | |
| Jason | Kim | | BS | Massachusetts General Hospital | Boston, Massachusetts, U.S.A. | Site Research Personnel | |
| Christine | Park | | BS | Massachusetts General Hospital | Boston, Massachusetts, U.S.A. | Site Research Personnel | |
| Rishabh | Phukan | | BS | Massachusetts General Hospital | Boston, Massachusetts, U.S.A. | Site Research Personnel | |
| Zachary | Wright | | BS | Massachusetts General Hospital | Boston, Massachusetts, U.S.A. | Site Research Personnel | |

Supplemental Online Content: Nonauthor Collaborators

*Indicates required information. Only first name, last name, and suffix will appear in PubMed.

| ***First Name and Middle Initial(s)** | ***Last Name** | ***Suffix (eg, Jr, III)** | Academic Degrees | Institution | Location (city, state/province, country) | Role or Contribution, eg, chair, principal investigator | Group (if more than 1 Group listed in the byline) and/or Subgroup (eg, Steering Committee) |
|---|---|---|---|---|---|---|---|
| Sarah | Yeates | | BS | Massachusetts General Hospital | Boston, Massachusetts, U.S.A. | Site Research Personnel | |
| Lalit | Joshi | | RPh | Massachusetts General Hospital | Boston, Massachusetts, U.S.A. | Site Research Pharmacy Personnel | |
| Timothy | Damron | | MD | State University of New York Upstate Orthopedics | Syracuse, New York, U.S.A. | Site Principal Investigator | |
| Tina | Craig | | CCRP | State University of New York Upstate Orthopedics | Syracuse, New York, U.S.A. | Site Research Personnel | |
| Melissa | Reale | | | State University of New York Upstate Orthopedics | Syracuse, New York, U.S.A. | Site Research Pharmacy Personnel | |
| Matthew R. | DiCaprio | | MD | Albany Medical Center | Albany, New York, U.S.A. | Site Principal Investigator | |
| Bradford A. | Palmer | | RPA-C | Albany Medical Center | Albany, New York, U.S.A. | Site Co-investigator | |
| Toni | Schaeffer | | PharmD | Albany Medical Center | Albany, New York, U.S.A. | Site Research Pharmacy Personnel | |
| Elena | Cioppa | | RPh, MS | Albany Medical Center | Albany, New York, U.S.A. | Site Research Pharmacy Personnel | |
| John C. | Neilson | | MD | Froedtert Hospital | Milwaukee, Wisconsin, U.S.A. | Site Principal Investigator | |
| David M. | King | | MD | Froedtert Hospital | Milwaukee, Wisconsin, U.S.A. | Site Co-investigator | |
| Adam N. | Wooldridge | | MD, MPH | Froedtert Hospital | Milwaukee, Wisconsin, U.S.A. | Site Co-investigator | |
| Karen C. | Gonzalez | | MS, CCRP | Froedtert Hospital | Milwaukee, Wisconsin, U.S.A. | Site Research Personnel | |

Supplemental Online Content: Nonauthor Collaborators

*Indicates required information. Only first name, last name, and suffix will appear in PubMed.

| ***First Name and Middle Initial(s)** | ***Last Name** | ***Suffix (eg, Jr, III)** | Academic Degrees | Institution | Location (city, state/province, country) | Role or Contribution, eg, chair, principal investigator | Group (if more than 1 Group listed in the byline) and/or Subgroup (eg, Steering Committee) |
|---|---|---|---|---|---|---|---|
| Marie | Ellestad | | CCRP | Froedtert Hospital | Milwaukee, Wisconsin, U.S.A. | Site Research Personnel | |
| Kate | Lewis | | PharmD, BCPS | Froedtert Hospital | Milwaukee, Wisconsin, U.S.A. | Site Research Pharmacy Personnel | |
| Tom | Nelson | | PharmD, RPh | Froedtert Hospital | Milwaukee, Wisconsin, U.S.A. | Site Research Pharmacy Personnel | |
| Nickolas | Reimer | | MD | Emory University Orthopedics and Spine Center | Atlanta, Georgia, U.S.A. | Site Principal Investigator | |
| David | Monson | | MD | Emory University Orthopedics and Spine Center | Atlanta, Georgia, U.S.A. | Site Co-investigator | |
| Shervin | Oskouei | | MD | Emory University Orthopedics and Spine Center | Atlanta, Georgia, U.S.A. | Site Co-investigator | |
| Christina | Lomba | | MS, CCRC | Emory University Orthopedics and Spine Center | Atlanta, Georgia, U.S.A. | Site Research Personnel | |
| Lauren | Glenney | | MHSc, CCRP | Emory University Orthopedics and Spine Center | Atlanta, Georgia, U.S.A. | Site Research Personnel | |
| Susan | Rogers | | RPh | Emory University Orthopedics and Spine Center | Atlanta, Georgia, U.S.A. | Site Research Pharmacy Personnel | |
| Howard | Goodman | | MD | Long Island Jewish Medical Center [Northwell Health] | New Hyde Park, New York, U.S.A. | Site Principal Investigator (Long Island Jewish Medical Center [Northwell Health], Maimonides Medical Center) | |
| Marlena | McGill | | MPH | Long Island Jewish Medical Center [Northwell Health] | New Hyde Park, New York, U.S.A. | Site Research Personnel | |

Supplemental Online Content: Nonauthor Collaborators

*Indicates required information. Only first name, last name, and suffix will appear in PubMed.

| *First Name and Middle Initial(s) | *Last Name | *Suffix (eg, Jr, III) | Academic Degrees | Institution | Location (city, state/province, country) | Role or Contribution, eg, chair, principal investigator | Group (if more than 1 Group listed in the byline) and/or Subgroup (eg, Steering Committee) |
|---|---|---|---|---|---|---|---|
| Peter | Olivares | | BSc | Long Island Jewish Medical Center [Northwell Health] | New Hyde Park, New York, U.S.A. | Site Research Personnel | |
| Francesca | Petruccelli | | BA | Long Island Jewish Medical Center [Northwell Health] | New Hyde Park, New York, U.S.A. | Site Research Personnel | |
| Uzma | Afzal | | PharmD | Long Island Jewish Medical Center [Northwell Health] | New Hyde Park, New York, U.S.A. | Site Research Pharmacy Personnel | |
| Zina | Faynblat | | RPh | Long Island Jewish Medical Center [Northwell Health] | New Hyde Park, New York, U.S.A. | Site Research Pharmacy Personnel | |
| Elizabeth | Mathew | | RPh | Long Island Jewish Medical Center [Northwell Health] | New Hyde Park, New York, U.S.A. | Site Research Pharmacy Personnel | |
| Wanda | Bell-Farrell | | RN, MS, CCRP | Sinai Hospital of Baltimore [Lifebridge Health] | Baltimore, Maryland, U.S.A. | Site Research Personnel | |
| Judith | Bosley | | RN, BSN | Sinai Hospital of Baltimore [Lifebridge Health] | Baltimore, Maryland, U.S.A. | Site Research Personnel | |
| Corilynn | Hughes | | RN, BSN, OCN | Sinai Hospital of Baltimore [Lifebridge Health] | Baltimore, Maryland, U.S.A. | Site Research Personnel | |
| Ukeme | Ikiddeh-Barnes | | RN | Sinai Hospital of Baltimore [Lifebridge Health] | Baltimore, Maryland, U.S.A. | Site Research Personnel | |
| Ashley | Jones | | BS | Sinai Hospital of Baltimore [Lifebridge Health] | Baltimore, Maryland, U.S.A. | Site Research Personnel | |
| Melissa | Loomis | | CCRP | Sinai Hospital of Baltimore [Lifebridge Health] | Baltimore, Maryland, U.S.A. | Site Research Personnel | |
| Alexis | Solis | | BS | Sinai Hospital of Baltimore [Lifebridge Health] | Baltimore, Maryland, U.S.A. | Site Research Personnel | |
| Christine | Wade | | BA | Sinai Hospital of Baltimore [Lifebridge Health] | Baltimore, Maryland, U.S.A. | Site Research Personnel | |

Supplemental Online Content: Nonauthor Collaborators

*Indicates required information. Only first name, last name, and suffix will appear in PubMed.

| *First Name and Middle Initial(s) | *Last Name | *Suffix (eg, Jr, III) | Academic Degrees | Institution | Location (city, state/province, country) | Role or Contribution, eg, chair, principal investigator | Group (if more than 1 Group listed in the byline) and/or Subgroup (eg, Steering Committee) |
|---|---|---|---|---|---|---|---|
| Stephanie | Friedman | | PharmD | Sinai Hospital of Baltimore [Lifebridge Health] | Baltimore, Maryland, U.S.A. | Site Research Pharmacy Personnel | |
| Chukwuemeka N. | Nzelibe | | PharmD | Sinai Hospital of Baltimore [Lifebridge Health] | Baltimore, Maryland, U.S.A. | Site Research Pharmacy Personnel | |
| Katiri | Wagner | | BS | Beth Israel Deaconess Medical Center | Boston, Massachusetts, U.S.A. | Site Research Personnel | |
| Hina A. | Jolin | | PharmD | Beth Israel Deaconess Medical Center | Boston, Massachusetts, U.S.A. | Site Research Pharmacy Personnel | |
| Heena | Patel | | RPh | Beth Israel Deaconess Medical Center | Boston, Massachusetts, U.S.A. | Site Research Pharmacy Personnel | |
| Joel | Sorger | | MD | Cincinnati Children's Hospital | Cincinnati, Ohio, U.S.A. | Site Principal Investigator | |
| Nichole | Leitsinger | | BS, CCRP | Cincinnati Children's Hospital | Cincinnati, Ohio, U.S.A. | Site Research Personnel | |
| Krista | Carpenter | | APRN | Cincinnati Children's Hospital | Cincinnati, Ohio, U.S.A. | Site Research Pharmacy Personnel | |
| Denise | LaGory | | RPh | Cincinnati Children's Hospital | Cincinnati, Ohio, U.S.A. | Site Research Pharmacy Personnel | |
| Steven | Thorpe | | MD | University of California, Davis Medical Center | Sacramento, California, U.S.A. | Site Principal Investigator | |
| Shari Lynn | Nichols | | CCRP, ADN | University of California, Davis Medical Center | Sacramento, California, U.S.A. | Site Research Personnel | |
| Patrick | Febre | | PharmD | University of California, Davis Medical Center | Sacramento, California, U.S.A. | Site Research Pharmacy Personnel | |

*Indicates required information. Only first name, last name, and suffix will appear in PubMed.

| ***First Name and Middle Initial(s)** | ***Last Name** | ***Suffix (eg, Jr, III)** | Academic Degrees | Institution | Location (city, state/province, country) | Role or Contribution, eg, chair, principal investigator | Group (if more than 1 Group listed in the byline) and/or Subgroup (eg, Steering Committee) |
|---|---|---|---|---|---|---|---|
| Jacob | Monares | | CPhT | University of California, Davis Medical Center | Sacramento, California, U.S.A. | Site Research Pharmacy Personnel | |
| Kimmai | Nguyen | | PharmD | University of California, Davis Medical Center | Sacramento, California, U.S.A. | Site Research Pharmacy Personnel | |
| Nadir | Sarwary | | CPhT | University of California, Davis Medical Center | Sacramento, California, U.S.A. | Site Research Pharmacy Personnel | |
| Peter | Trovitch | | PharmD | University of California, Davis Medical Center | Sacramento, California, U.S.A. | Site Research Pharmacy Personnel | |
| Nicholas | Bernthal | | MD | University of California, Los Angeles Medical Center | Los Angeles, California, U.S.A. | Site Principal Investigator | |
| Jeffrey | Eckardt | | MD | University of California, Los Angeles Medical Center | Los Angeles, California, U.S.A. | Site Co-investigator | |
| Francis | Hornicek | | MD, PhD | University of California, Los Angeles Medical Center | Los Angeles, California, U.S.A. | Site Co-investigator | |
| Stephen | Zoller | | MD | University of California, Los Angeles Medical Center | Los Angeles, California, U.S.A. | Site Research Personnel | |
| Gloria | Kiel | | | University of California, Los Angeles Medical Center | Los Angeles, California, U.S.A. | Site Research Personnel | |
| Jason | Madamba | | PharmD, BCPS | University of California, Los Angeles Medical Center | Los Angeles, California, U.S.A. | Site Research Pharmacy Personnel | |
| Christina | Shin | | PharmD | University of California, Los Angeles Medical Center | Los Angeles, California, U.S.A. | Site Research Pharmacy Personnel | |

Supplemental Online Content: Nonauthor Collaborators

*Indicates required information. Only first name, last name, and suffix will appear in PubMed.

| *First Name and Middle Initial(s) | *Last Name | *Suffix (eg, Jr, III) | Academic Degrees | Institution | Location (city, state/province, country) | Role or Contribution, eg, chair, principal investigator | Group (if more than 1 Group listed in the byline) and/or Subgroup (eg, Steering Committee) |
|---|---|---|---|---|---|---|---|
| Adam | Lindsay | | MD | Hartford Hospital | Hartford, Connecticut | Site Principal Investigator (Hartford Hospital, UConn Health) | |
| Jamie | Fish-Fuhrmann | | BS | Hartford Hospital | Hartford, Connecticut | Site Research Personnel | |
| Maya | Culbertson | | MS | Maimonides Medical Center | New York City, New York, U.S.A. | Site Research Personnel | |
| Patricia | Caruso-Prendergast | | MS, PharmD, BCPS | Maimonides Medical Center | New York City, New York, U.S.A. | Site Research Pharmacy Personnel | |
| Emily | Garling | | PharmD | Maimonides Medical Center | New York City, New York, U.S.A. | Site Research Pharmacy Personnel | |
| Richard | Nicholas | | MD | University of Arkansas for Medical Sciences | Little Rock, Arkansas, U.S.A. | Site Principal Investigator | |
| Corey | Montgomery | | MD | University of Arkansas for Medical Sciences | Little Rock, Arkansas, U.S.A. | Site Co-investigator | |
| J. Aaron | Holley | | BS | University of Arkansas for Medical Sciences | Little Rock, Arkansas, U.S.A. | Site Research Personnel | |
| Rachel | Jones | | MSc | University of Arkansas for Medical Sciences | Little Rock, Arkansas, U.S.A. | Site Research Personnel | |
| Melissa | McAdoo | | BSN | University of Arkansas for Medical Sciences | Little Rock, Arkansas, U.S.A. | Site Research Personnel | |
| Daisy | Wade | | BA | University of Arkansas for Medical Sciences | Little Rock, Arkansas, U.S.A. | Site Research Personnel | |
| Mindy | Caid | | BS | University of Arkansas for Medical Sciences | Little Rock, Arkansas, U.S.A. | Site Research Pharmacy Personnel | |

Supplemental Online Content: Nonauthor Collaborators

*Indicates required information. Only first name, last name, and suffix will appear in PubMed.

| ***First Name and Middle Initial(s)** | ***Last Name** | ***Suffix (eg, Jr, III)** | Academic Degrees | Institution | Location (city, state/province, country) | Role or Contribution, eg, chair, principal investigator | Group (if more than 1 Group listed in the byline) and/or Subgroup (eg, Steering Committee) |
|---|---|---|---|---|---|---|---|
| Amy | Crisp | | PharmD | University of Arkansas for Medical Sciences | Little Rock, Arkansas, U.S.A. | Site Research Pharmacy Personnel | |
| Jennifer | Roberts | | PharmD | University of Arkansas for Medical Sciences | Little Rock, Arkansas, U.S.A. | Site Research Pharmacy Personnel | |
| Tess | Balach | | MD | UConn Health | Farmington, Connecticut, U.S.A. | Site Principal Investigator | |
| Mark | Cote | | PT, DPT, MS | UConn Health | Farmington, Connecticut, U.S.A. | Site Research Personnel | |
| Kathleen | Coyle | | RN, BSN, MPH | UConn Health | Farmington, Connecticut, U.S.A. | Site Research Personnel | |
| Kelly | Rushlow | | BA | UConn Health | Farmington, Connecticut, U.S.A. | Site Research Personnel | |
| Ruth | LaCasse | | RPh | UConn Health | Farmington, Connecticut, U.S.A. | Site Research Pharmacy Personnel | |
| Daniel | Lerman | | MD | University of Maryland Medical Center | Baltimore, Maryland, U.S.A. | Site Principal Investigator | |
| Andrea | Howe | | BS | University of Maryland Medical Center | Baltimore, Maryland, U.S.A. | Site Research Personnel | |
| Prashant | Patel | | PharmD | University of Maryland Medical Center | Baltimore, Maryland, U.S.A. | Site Research Pharmacy Personnel | |
| Andrew | Phan | | PharmD | University of Maryland Medical Center | Baltimore, Maryland, U.S.A. | Site Research Pharmacy Personnel | |
| Shinyi | Telscher | | PharmD, CCRP | University of Maryland Medical Center | Baltimore, Maryland, U.S.A. | Site Research Pharmacy Personnel | |

Supplemental Online Content: Nonauthor Collaborators

*Indicates required information. Only first name, last name, and suffix will appear in PubMed.

| *First Name and Middle Initial(s) | *Last Name | *Suffix (eg, Jr, III) | Academic Degrees | Institution | Location (city, state/province, country) | Role or Contribution, eg, chair, principal investigator | Group (if more than 1 Group listed in the byline) and/or Subgroup (eg, Steering Committee) |
|---|---|---|---|---|---|---|---|
| Kurt | Weiss | | MD | University of Pittsburgh Medical Center | Pittsburgh, Pennsylvania, U.S.A. | Site Principal Investigator | |
| Mark | Goodman | | MD | University of Pittsburgh Medical Center | Pittsburgh, Pennsylvania, U.S.A. | Site Co-investigator | |
| Alma | Heyl | | CCRC, LAS | University of Pittsburgh Medical Center | Pittsburgh, Pennsylvania, U.S.A. | Site Research Personnel | |
| Chris | Korenoski | | PharmD | University of Pittsburgh Medical Center | Pittsburgh, Pennsylvania, U.S.A. | Site Research Pharmacy Personnel | |
| Chris Ann | Yeschke | | PharmD | University of Pittsburgh Medical Center | Pittsburgh, Pennsylvania, U.S.A. | Site Research Pharmacy Personnel | |
| Thomas | Scharschmidt | | MD | Wexner Medical Center | Columbus, Ohio, U.S.A. | Site Principal Investigator | |
| Joel | Mayerson | | MD | Wexner Medical Center | Columbus, Ohio, U.S.A. | Site Co-investigator | |
| Martha | Crist | | RN | Wexner Medical Center | Columbus, Ohio, U.S.A. | Site Research Personnel | |
| Hallie | Barr | | PharmD, BCOP | Wexner Medical Center | Columbus, Ohio, U.S.A. | Site Research Pharmacy Personnel | |
| Shah Alam | Khan | | MS (Ortho) | All India Institute of Medical Sciences | New Delhi, India | Site Principal Investigator | |
| Venkatesan | Sampath Kumar | | MS (Ortho) | All India Institute of Medical Sciences | New Delhi, India | Site Co-investigator | |
| Abhinav | Agarwal | | MS (Ortho) | All India Institute of Medical Sciences | New Delhi, India | Site Research Personnel | |
| Roshan | Banjara | | MS (Ortho) | All India Institute of Medical Sciences | New Delhi, India | Site Research Personnel | |

Supplemental Online Content: Nonauthor Collaborators

*Indicates required information. Only first name, last name, and suffix will appear in PubMed.

| ***First Name and Middle Initial(s)** | ***Last Name** | ***Suffix (eg, Jr, III)** | Academic Degrees | Institution | Location (city, state/province, country) | Role or Contribution, eg, chair, principal investigator | Group (if more than 1 Group listed in the byline) and/or Subgroup (eg, Steering Committee) |
|---|---|---|---|---|---|---|---|
| Sanjay | Oli | | BA (Sociology) | All India Institute of Medical Sciences | New Delhi, India | Site Research Personnel | |
| André Mathias | Baptista | | MD, PhD | Instituto de Ortopedia e Traumatologia da Universidade de São Paulo | São Paulo, Brazil | Site Principal Investigator | |
| Olavo | Pires de Camargo | | MD, PhD | Instituto de Ortopedia e Traumatologia da Universidade de São Paulo | São Paulo, Brazil | Site Co-investigator | |
| Juan Pablo | Zumárraga | | MD, MSc, PhD | Instituto de Ortopedia e Traumatologia da Universidade de São Paulo | São Paulo, Brazil | Site Co-investigator | |
| Juliana | Freitar | | RN | Instituto de Ortopedia e Traumatologia da Universidade de São Paulo | São Paulo, Brazil | Site Research Pharmacy Personnel | |
| Ismael | Agomes | | RN | Instituto de Ortopedia e Traumatologia da Universidade de São Paulo | São Paulo, Brazil | Site Research Pharmacy Personnel | |
| P.D. Sander | Dijkstra | | MD, PhD | Leiden University Medical Center | Leiden, the Netherlands | Site Principal Investigator | |
| Michiel | van de Sande | | MD, PhD | Leiden University Medical Center | Leiden, the Netherlands | Site Co-Principal Investigator | |
| Philip | Sanders | | MD | Leiden University Medical Center | Leiden, the Netherlands | Site Research Personnel | |
| Sarah | Bosma | | MD | Leiden University Medical Center | Leiden, the Netherlands | Site Research Personnel | |
| Marieke Afra | Toi | | PharmD | Leiden University Medical Center | Leiden, the Netherlands | Site Research Pharmacy Personnel | |
| Marcos Galli | Serra | | MD | Hospital Universitario Austral | Buenos Aires, Argentina | Site Principal Investigator | |

Supplemental Online Content: Nonauthor Collaborators

*Indicates required information. Only first name, last name, and suffix will appear in PubMed.

| ***First Name and Middle Initial(s)** | ***Last Name** | ***Suffix (eg, Jr, III)** | Academic Degrees | Institution | Location (city, state/province, country) | Role or Contribution, eg, chair, principal investigator | Group (if more than 1 Group listed in the byline) and/or Subgroup (eg, Steering Committee) |
|---|---|---|---|---|---|---|---|
| Walter | Parizzia | | MD | Hospital Universitario Austral | Buenos Aires, Argentina | Site Co-investigator | |
| Gabriela | Marinsalta | | BIOCH | Hospital Universitario Austral | Buenos Aires, Argentina | Site Research Personnel | |
| Angela | Podrzaj | | | Hospital Universitario Austral | Buenos Aires, Argentina | Site Research Personnel | |
| Mariana Foa | Torres | | RN | Hospital Universitario Austral | Buenos Aires, Argentina | Site Research Pharmacy Personnel | |
| Manuel | Pérez | | MD | Hospital Vall d'Hebron | Barcelona, Spain | Site Co-investigator | |
| Lourdes Girona | Brumós | | PharmD | Hospital Vall d'Hebron | Barcelona, Spain | Site Research Pharmacy Personnel | |
| Pilar | Suñé | | PharmD | Hospital Vall d'Hebron | Barcelona, Spain | Site Research Pharmacy Personnel | |
| Reitze | Rodseth | | MD, PhD | School of Clinical Medicine, University of KwaZulu-Natal/Grey's Hospital | Pietermaritzburg, South Africa | Site Principal Investigator | |
| Leonard | Marais | | MD, PhD | School of Clinical Medicine, University of KwaZulu-Natal/Grey's Hospital | Pietermaritzburg, South Africa | Site Co-Principal Investigator | |
| Luan | Nieuwoudt | | MD | School of Clinical Medicine, University of KwaZulu-Natal/Grey's Hospital | Pietermaritzburg, South Africa | Site Co-investigator | |
| Chantal | Rajah | | MD | School of Clinical Medicine, University of KwaZulu-Natal/Grey's Hospital | Pietermaritzburg, South Africa | Site Co-investigator | |

*Indicates required information. Only first name, last name, and suffix will appear in PubMed.

| ***First Name and Middle Initial(s)** | ***Last Name** | ***Suffix (eg, Jr, III)** | Academic Degrees | Institution | Location (city, state/province, country) | Role or Contribution, eg, chair, principal investigator | Group (if more than 1 Group listed in the byline) and/or Subgroup (eg, Steering Committee) |
|---|---|---|---|---|---|---|---|
| Simphiwe | Gumede | | | School of Clinical Medicine, University of KwaZulu-Natal/Grey's Hospital | Pietermaritzburg, South Africa | Site Research Personnel | |
| Andreas | Leithner | | MD | Medical University Graz | Graz, Austria | Site Principal Investigator | |
| Marko | Bergovec | | MD | Medical University Graz | Graz, Austria | Site Co-investigator | |
| Andrea | Fink | | MSc | Medical University Graz | Graz, Austria | Site Research Personnel | |
| Carina | Halb | | Mag | Medical University Graz | Graz, Austria | Site Research Pharmacy Personnel | |
| Ahmed | El Ghoneimy | | MD | Children's Cancer Hospital Egypt | Cairo, Egypt | Site Principal Investigator | |
| Dina | Elgalaly | | BPharm | Children's Cancer Hospital Egypt | Cairo, Egypt | Site Research Pharmacy Personnel | |
| Nehal | Kamal | | BPharm | Children's Cancer Hospital Egypt | Cairo, Egypt | Site Research Pharmacy Personnel | |
| Ricardo | Becker | | MD, MSc, PhD | Hospital de Clínicas de Porto Alegre | Porto Alegre, Brazil | Site Principal Investigator | |
| Bruno Pereira | Antunes | | MD, MSc | Hospital de Clínicas de Porto Alegre | Porto Alegre, Brazil | Site Co-investigator | |
| Carlos Roberto | Galia | | MD, MSc, PhD | Hospital de Clínicas de Porto Alegre | Porto Alegre, Brazil | Site Co-investigator | |
| Julie F. Cerutti | Santos | | RN, MSc | Hospital de Clínicas de Porto Alegre | Porto Alegre, Brazil | Site Research Personnel | |
| Daniel | Fasolo | | BPharm, MSc, PhD | Hospital de Clínicas de Porto Alegre | Porto Alegre, Brazil | Site Research Pharmacy Personnel | |

Supplemental Online Content: Nonauthor Collaborators

*Indicates required information. Only first name, last name, and suffix will appear in PubMed.

| ***First Name and Middle Initial(s)** | ***Last Name** | ***Suffix (eg, Jr, III)** | Academic Degrees | Institution | Location (city, state/province, country) | Role or Contribution, eg, chair, principal investigator | Group (if more than 1 Group listed in the byline) and/or Subgroup (eg, Steering Committee) |
|---|---|---|---|---|---|---|---|
| Mann Hong | Tan | | MBBS (Singapore), FRCS (Edinburgh), FRCS (Glasgow), FAMS (Orthopaedic Surgery) | Singapore General Hospital | Singapore, Singapore | Site Principal Investigator | |
| Suraya | Zainul Abidin | | MBBS BSc (Hons), MMed (Ortho), FRCS (Edinburgh) | Singapore General Hospital | Singapore, Singapore | Site Co-investigator | |
| Lai Ye | Cheang | | MPharm | Singapore General Hospital | Singapore, Singapore | Site Research Pharmacy Personnel | |
| Mark | Clayer | | MD, MSc, MBBS | Royal Adelaide Hospital | Adelaide, Australia | Site Principal Investigator | |
| Jakub | Jagiello | | MBBS | Royal Adelaide Hospital | Adelaide, Australia | Site Co-investigator | |
| David | Morris | | MD | Royal Adelaide Hospital | Adelaide, Australia | Site Co-investigator | |
| Yee | Chai | | BPharm | Royal Adelaide Hospital | Adelaide, Australia | Site Research Pharmacy Personnel | |

Supplemental Online Content: Nonauthor Collaborators

*Indicates required information. Only first name, last name, and suffix will appear in PubMed.

| ***First Name and Middle Initial(s)** | ***Last Name** | ***Suffix (eg, Jr, III)** | Academic Degrees | Institution | Location (city, state/province, country) | Role or Contribution, eg, chair, principal investigator | Group (if more than 1 Group listed in the byline) and/or Subgroup (eg, Steering Committee) |
|---|---|---|---|---|---|---|---|
| Steven | Duong | | BPharm | Royal Adelaide Hospital | Adelaide, Australia | Site Research Pharmacy Personnel | |
| Tran | Nguyen | | BPharm | Royal Adelaide Hospital | Adelaide, Australia | Site Research Pharmacy Personnel | |
| Peter | Slobodian | | BPharm, MClinPharm | Royal Adelaide Hospital | Adelaide, Australia | Site Research Pharmacy Personnel | |
| Paul | Jutte | | MD, PhD | University Medical Center Groningen | Groningen, the Netherlands | Site Principal Investigator | |
| Marlanka | Zuur | | PhD | University Medical Center Groningen | Groningen, the Netherlands | Site Research Pharmacy Personnel | |
