# Supplementary material for: Comparison of Prophylactic Intravenous Antibiotic Regimens After Endoprosthetic Reconstruction for Lower Extremity Bone Tumors: A Randomized Clinical Trial
Source: JAMA Oncol. 2022 Jan 6;8(3):345–53. doi: 10.1001/jamaoncol.2021.6628 (PMC8739829; doi:10.1001/jamaoncol.2021.6628)
Supplement: Supplement 4. — Data Sharing Statement [file jamaoncol-e216628-s004.pdf]

Data Sharing Statement

Ghert. Comparison of Prophylactic Intravenous Antibiotic Regimens After Endoprosthetic Reconstruction for Lower Extremity Bone Tumors. *JAMA Oncol*. Published January 06, 2022. doi:10.1001/jamaoncol.2021.6628

Data

Data available: Yes
Data types: Deidentified participant data
How to access data: A written request for access to the data must be submitted to the corresponding author via email at: ghertm@mcmaster.ca.
When available: beginning date: 01-06-2023

Supporting Documents

Document types: Statistical/analytic code, Informed consent form
How to access documents: A written request for access to the statistical/analytic code and Informed Consent Form must be submitted to the corresponding author via email at: ghertm@mcmaster.ca.
When available: beginning date: 01-06-2023

Additional Information

Who can access the data: Data will be made available to anyone requesting the data.
Types of analyses: Data will be made available for any purpose.
Mechanisms of data availability: Data will be made available to anyone requesting the data with a signed data access agreement.
Any additional restrictions: Data will only be made available one year after publication date or January 6, 2023, whichever comes last.
